# Global analysis of gene expression changes during retinoic acid-induced growth arrest and differentiation of melanoma: comparison to differentially expressed genes in melanocytes vs melanoma

**DOI:** 10.1186/1471-2164-9-478

**Published:** 2008-10-11

**Authors:** Mary Estler, Goran Boskovic, James Denvir, Sarah Miles, Donald A Primerano, Richard M Niles

**Affiliations:** 1 Department of Biochemistry and Microbiology, Joan C. Edwards School of Medicine, Marshall University, One John Marshall Drive – BBSC, Huntington, WV 25755, USA

## Abstract

**Background:**

The incidence of malignant melanoma has significantly increased over the last decade. Some of these malignancies are susceptible to the growth inhibitory and pro-differentiating effects of all-*trans*-retinoic acid (RA). The molecular changes responsible for the biological activity of RA in melanoma are not well understood.

**Results:**

In an analysis of sequential global gene expression changes during a 4–48 h RA treatment of B16 mouse melanoma cells, we found that RA increased the expression of 757 genes and decreased the expression of 737 genes. We also compared the gene expression profile (no RA treatment) between non-malignant melan-a mouse melanocytes and B16 melanoma cells. Using the same statistical test, we found 1495 genes whose expression was significantly higher in melan-a than in B16 cells and 2054 genes whose expression was significantly lower in melan-a than in B16 cells. By intersecting these two gene sets, we discovered a common set of 233 genes whose RNA levels were significantly different between B16 and melan-a cells and whose expression was altered by RA treatment. Within this set, RA treatment altered the expression of 203 (87%) genes toward the melan-a expression level. In addition, hierarchical clustering showed that after 48 h of RA treatment expression of the 203 genes was more closely related to the melan-a gene set than any other RA treatment time point. Functional analysis of the 203 gene set indicated that RA decreased expression of mRNAs that encode proteins involved in cell division/cell cycle, DNA replication, recombination and repair, and transcription regulation. Conversely, it stimulated genes involved in cell-cell signaling, cell adhesion and cell differentiation/embryonic development. Pathway analysis of the 203 gene set revealed four major hubs of connectivity: CDC2, CHEK1, CDC45L and MCM6.

**Conclusion:**

Our analysis of common genes in the 48 h RA-treatment of B16 melanoma cells and untreated B16 vs. melan-a data set show that RA "normalized" the expression of genes involved in energy metabolism, DNA replication, DNA repair and differentiation. These results are compatible with the known growth inhibitory and pro-differentiating effects of RA. Pathway analysis suggests that CDC2, CHEK1, CDC45L and MCM6 are key players in mediating the biological activity of RA in B16 melanoma cells.

## Background

Malignant melanoma is one of the fastest rising cancers in the US population and its yearly incidence is estimated at 60,000 [1]. If it is diagnosed early and is restricted to the epidermis, surgical resection can yield high cure rates. However, once the melanoma invades through the basement membrane into the dermis, surgical resection is less successful and there is no effective radio/chemotherapy for patients with metastatic melanoma.

Vitamin A (retinol) is an essential nutrient that is required for night vision, reproduction, embryogenesis and differentiation [2]. Retinol is metabolized by cells into a number of compounds, the most biologically active being all-*trans*-retinoic acid (RA). The biological changes induced by RA are mediated by binding and activation of nuclear receptors [3,4]. There are three subtypes of retinoic acid receptors (RAR); RAR-α, β and γ, which bind RA. Another vitamin A metabolite, 9-cis RA, also binds these receptors but in addition serves as the ligand for closely related nuclear receptors, the retinoid × receptors (RXRs) [5,6]. Analogous to the RARs, there are three RXR subtypes, α, β and γ [7]. RXRs form heterodimers with the RARs as well as with several other members of the nuclear hormone receptor family, such as Peroxisome Proliferator Activated Receptor (PPAR) [8], vitamin D3 [9] and thyroid hormone receptors [10]. The RXR:RAR heterodimer appears to be the physiologically relevant dimer for stimulating target gene expression [11]. This receptor heterodimer binds to a DNA element in the 5' flanking region of target genes and usually consists of a direct repeat of the sequence 5'-(A/G)G(G/T)TCA-3' separated by 5 bp [12,13]. These receptors are also regulated by co-repressors and co-activators. Several of the co-activators contain histone acetyltransferase activity, while co-repressor complexes contain histone deacetylase activity, suggesting that localized chromatin remodeling modulates receptor activity [14].

RA inhibits the proliferation and stimulates the differentiation of many different tumor cell lines, including murine and human melanoma cells [15]. We have previously used B16 mouse melanoma as a model for our retinoid studies. This cell line is very responsive to the growth inhibitory and pro-differentiating effects of retinoic acid [16]. The B16 cell line also has major deletions in the *p16*^*INK*4*a *^and *p19*^*QRF*^genes with consequent loss of expression of these proteins, but it lacks an activating mutation in the *BRAF *gene [17]. Loss of *p16*^*INK*4*a *^and *p19*^*ORF *^contributes to melanomagenesis by overcoming senescence [18]. In addition, activating *BRAF *mutations have been found in up to 60% of sporadic human melanomas [19]. We have found that our strain of B16 melanoma has an activating N-ras mutation (Niles et al., unpublished). Thus B16 cells have a number of the genetic/protein changes found in human melanoma cell lines and clinical melanoma specimens.

Melan-a cells are nonmalignant mouse melanocytes derived from C57BL/6 mice (the same origin as B16 melanoma) that have been partially immortalized. They have diploid chromosomes and do not form tumors in syngeneic or athymic mice [20]. Therefore melan-a cells serve as a good model to compare gene expression profiles to B16 melanoma cells with the aim of identifying genes whose altered expression might contribute to malignancy. In the study reported here, we used DNA microarrays to compare gene expression patterns between B16 and melan-a cells. We also used DNA microarrays to identify time-dependent changes in gene expression during RA-induced growth arrest and differentiation of B16 melanoma cells. From these two data sets, we then performed an analysis to identify a unique gene set, whose expression was altered in B16 compared to melan-a and whose expression was restored toward the melan-a level in RA-treated B16 melanoma cells.

## Results

### Time-dependent regulation of gene expression by RA

Our previous studies [21] have determined the amount of time required for RA to induce significant growth arrest and differentiation of B16 melanoma cells to be 48 – 72 h. Using DNA microarrays, we profiled the series of changes in gene expression that occurred in RA-treated cells prior to and coincident with these phenotypic changes. Table 1 shows the number of genes significantly up-regulated or down-regulated at 4, 10, 24, and 48 h of RA treatment in B16 melanoma cells. The statistical analysis of microarrays (SAM) method [22] was used to determine significance in all microarray studies reported in this communication. Only 14 genes were differentially regulated at the 4 hour time point. This set includes two genes, *RARβ *and retinal short chain dehydrogenase 3 (*DHRS3*), whose expression has previously been shown to be induced by RA [23,24]. The number of RA-regulated genes increases over time, with the largest increase between the 24 h and 48 h time points. The number of upregulated genes exceeds the downregulated genes at all time points except the 48 h time point. A few genes (*RARβ *and *DHRS3*) were up- or down-regulated during the entire time of retinoic acid treatment, but more commonly, genes exhibited altered expression for one or two time points and then their expression returns to control levels. The identity of these RA-regulated genes can be found in Additional file 1.

**Table 1:** Numbers of genes significantly differentially expressed relative to untreated B16 cells^a^

	Retinoic Acid Treatment				melan-a vs. B16
		
	4 hour	10 hour	24 hour	48 hour	
Upregulated	13	25	151	701	1495
Downregulated	1	3	3	734	2054
Total	14	28	154	1435	3549

^a ^Significance was determined by Significance Analysis of Microarrays (SAM) with median false discovery rate of 10%, and a minimum fold change of 1.5.

### Expression profiling of untreated melan-a nonmalignant mouse melanocytes vs. B16 mouse melanoma cells

Melan-a and B16 cells are both derived from the C57BL/6 strain of mouse. The melan-a cells are partially immortalized, but they are diploid, lack transformed cell phenotypes and do not form tumors in syngeneic mice [20]. A comparison of global gene expression between these two melanocyte/melanoma cell lines has not been reported. Our analyses show that 1495 genes had expression levels significantly higher in melan-a vs. B16 cells, i.e. their expression was downregulated in the malignant melanoma cells relative to the non-malignant cells (Table 1). In addition, there were 2054 genes whose expression levels were significantly lower in melan-a cells vs. B16 cells, i.e. their expression was upregulated in the malignant melanoma cells. The identity of these differentially regulated genes can be found in Additional file 2.

### Gene expression changes induced by RA treatment of B16 cells that mimic expression in Melan-a cells: the *intersecting gene set*

We combined data sets from RA-treated B16 melanoma cells and untreated melan-a vs. B16 data sets in order to discover common genes that were significantly changed in both sets (Table 2). There were 71 genes that were upregulated in melan-a compared to B16 cells and whose expression was upregulated by RA treatment of B16 cells at one or more time point. There were however a small number of genes (16) whose expression was downregulated in B16 compared to melan-a and whose expression was also decreased by RA treatment of B16 cells. Likewise, there were 132 genes whose expression was downregulated in melan-a cells compared to B16 cells and whose expression was decreased by RA treatment of B16 cells. Again, a small number of genes (14) had increased expression in B16 vs. melan-a and treatment of B16 cells with RA further enhanced their expression. Overall, the majority of genes in this combined data set (203 out of 233) were those whose expression was restored toward the melan-a (non-malignant melanocyte) level when B16 melanoma cells were treated with RA for 48 h. Three of the 203 genes (*DHRS3*, calcium/calmodulin-dependent protein kinase II inhibitor 1 (*CAMK2N1*) and *RIKEN cDNA 3110001A13*) were upregulated by RA at 4 h. The identities of these 203 genes, their fold changes at 48 h of RA treatment and their relative expression in melan-a vs. B16 cells are shown in Additional file 3.

**Table 2:** Numbers of genes in the intersections of gene sets generated by the two experiments

	Over-expressed in melan-a v B16	Under-expressed in melan-a v B16	Total
Upregulated by RA treatment	71	14	85
Downregulated by RA treatment	16	132	151
Total	87	149	233

In both the experiment comparing gene expression in RA-treated B16 cells to that in untreated B16 cells, and the experiment comparing gene expression in melan-a cells to that in B16 cells, significance is determined by SAM with median false discovery rate of 10%, and a minimum fold change of 1.5. Table entries in bold represent genes for which treatment by RA changes the expression in B16 cells towards that in melan-a cells.

We determined if members of the 203 gene set were enriched in known biological processes listed in the Gene Ontology database by using the Fisher Exact Test in Ariadne Genomics Pathway Studio. Additional file 4 provides a list of these processes ordered by statistical significance. The five biological processes which were most significant are cell division, cell cycle, cell cycle regulation, DNA replication and mitosis. In these processes, all genes were expressed higher in B16 than in melan-a cells. Examination of members within the processes reveals gene products that are clearly involved in promoting the cell division and DNA replication. For example, in the DNA replication set, 12 of the 13 genes encode proteins known to function directly in DNA synthesis or nucleotide metabolism (TOP2A, MCM2, MCM4, MCM6, CDC6, POLA, POLE, CDC45L, DUT, RNASEH2A, RPA1 and ZRF1). The identity of each gene within the five processes is given in Additional file 5. Several significant biological processes (protein phosphorylation, proteolysis, apoptosis induction cell-cell signaling) contained genes that were overexpressed in melan-a cells. These findings are consistent with the more rapid doubling of B16 (16 hours in log phase) in contrast to melan-a cells (28 hours in log phase). The total number of genes in both columns is greater than 203 because several genes are present in more than one process.

We confirmed the authenticity of the microarray-measured expression changes for seven genes within the 203 gene set by quantitative real time polymerase chain reaction (qRT-PCR) (Table 3). All gene expression changes except for the *PLAT *melan-a vs. B16 ratio were verified by these independent analyses. Protein Kinase C alpha (PKCα), not listed here, but a member of the 203 gene set, has previously been verified by us using both Northern and Western analysis [25]. It is interesting to note that in most instances, the reported degree of difference in gene expression between melan-a vs. B16 or RA treatment at 48 h is greater by qRT-PCR analysis than by microarray analysis. Thus the microarray technology tends to underestimate changes in gene expression between the comparison groups. This phenomenon has been previously reported by Yuen et al. [26].

**Table 3:** Validation of microarray-based mRNA levels by qRT-PCR

		**Microarray**		**qRT-PCR**	
		
**Gene**	**Accession Number**	**B16 48 h RA vs. control**	**melan-a vs. B16**	**B16 48 h RA vs. control**	**melan-a vs. B16**
*BRCA2*	NM_009765	0.30	0.36	0.301	0.0938
*CDC2A*	NM_007659	0.40	0.22	0.298	0.0311
*TOP2A*	NM_011623	0.50	0.11	0.226	0.00668
*TINAGL*	NM_023476	3.70	4.00	1.81	5.64
*PLAT*	NM_008872	2.10	3.33	3.15	1.43*
*DHRS3*	NM_011303	4.60	2.70	4.89	133
*FGF1*	NM_010197	2.20	2.27	3.53	2.35

All qRT-PCR values were significant by Bonferroni-corrected (n = 14) T-test with p < 0.01 with the exception of starred (*) value.

### Clustering and pathway analysis

**Figure 1:**
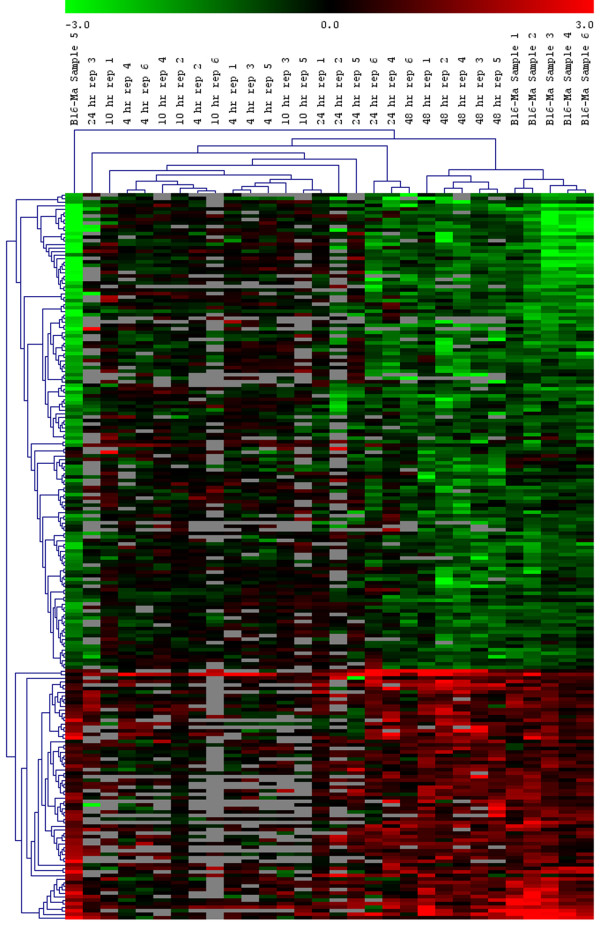
**Hierarchical clustering of the 203 genes for which RA treatment of B16 cells reverts the expression towards that of melan-a cells**. Average linkage was used with a Euclidean metric. The green-red color scale refers to log base two expression ratios with expression in untreated B16 cells as the denominator in all cases. Grey cells represent values which failed to pass the minimum expression level filter.

Hierarchical clustering of the 203 genes over all 30 arrays (six replicates each of the RA-treated cells at 4 h, 10 h, 24 h, and 48 h, and six replicates of the melan-a cells compared to untreated B16 cells) is shown in Fig. 1. We observed that the 4 and 10 hours arrays tend to cluster together, indicating that there is little overall expression difference between these two time points. However, the 4 hour arrays and 10 hour arrays considered as a single set form a tight cluster, indicating that the expression at the 4- and 10-hour time points is significantly different from later time points. The majority of the 48 h arrays form a single cluster, as do the majority of the arrays comparing melan-a expression to B16 expression. Furthermore, the expression at the 48 h time point for these 203 genes is closer to the expression in melan-a cells than to any of the RA-treated cells at other time points. The arrays at 24 h do not form a cluster, indicating that there is much greater variation in expression at this time point than at the other time points or in the melan-a expression profile.

**Figure 2:**
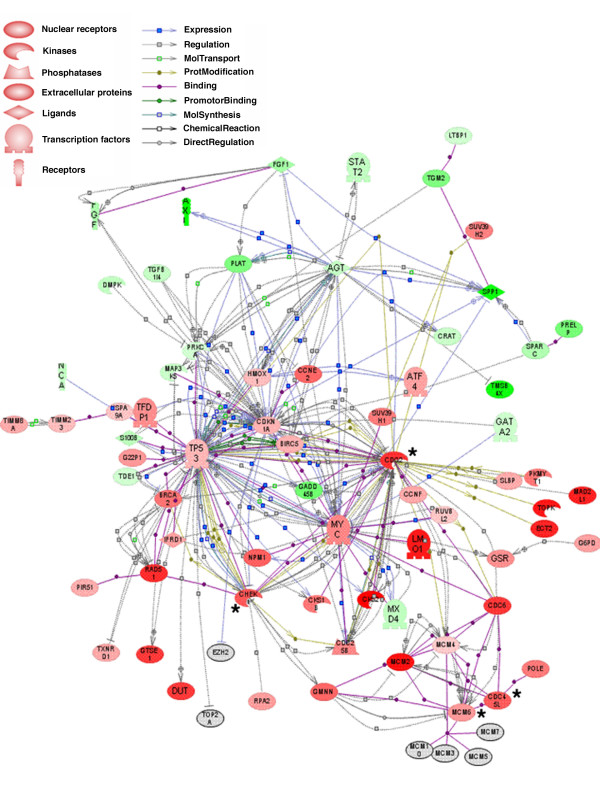
**Pathway Studio Analysis of 203 Gene Set**. Out of 203 genes, 74 genes were found to be involved in direct interactions. Protein hubs which have most significant interactions (CDC2, CHEK1, CDC45L and MCM6) are marked with an asterisk (*). Although there are relatively large numbers of interactions stemming from TP53, P21 and CMYC, these proteins were not among the most statistically significant hubs. Green color indicates a gene is upregulated in melan-a relative to B16 whereas red color indicates that a gene is downregulated in melan-a relative to B16. Color intensity reflects the expression ratio.

Lastly we used Pathway Studio v5.0 (Ariadne Genomics) program to identify and illustrate molecular connections between the proteins encoded by the 203 gene set. This program searches through the ResNet database for all known interactions between genes/proteins such as physical interaction, regulation of expression and protein modification (e.g. phosphorylation) and expresses the result in graphical form (Fig. 2). We calculated the statistical significance of the number of interactions, accounting for the number of known interactions in the Pathway Studio database. Those with Holm-Bonferroni adjusted *p *< 0.05 are shown in Table 4. Based on the most significant *p*-values, we identified four major "hubs" for connectivity; cell division cycle 2 protein (CDC2), checkpoint kinase 1(CHEK1), CDC45 cell division cycle 45-like (CDC45L) and minichromosome maintenance deficient protein 6 (MCM6). Three hubs with relatively high numbers of interactions (p53 protein (TP53), cyclin-dependent kinase inhibitor 1A protein (CDKN1A/p21/WAF1/CIP1) and CMYC protein) also met our criteria for statistical significance but with lower *p*-values.

**Table 4:** Number of interactions among genes in the 203 gene set

Gene	Number of Interactions	Adjusted p-value
*CDC2*	17	1.25 × 10^-08^

*CHEK1*	9	1.05 × 10^-07^

*CDC45L*	5	1.03 × 10^-06^

*MCM6*	5	1.98 × 10^-05^

*P21*	13	2.66 × 10^-05^

*MCM4*	5	6.96 × 10^-05^

*CDC6*	6	1.70 × 10^-04^

*GADD45B*	4	3.44 × 10^-04^

*CMYC*	13	5.55 × 10^-04^

*PLAT*	6	6.17 × 10^-04^

*FGF1*	5	1.50 × 10^-03^

*CDC25B*	4	3.11 × 10^-03^

*MCM2*	4	8.50 × 10^-03^

*STAT2*	11	0.0119

*BRCA2*	7	0.0334

*SPA9A*	2	0.0361

*P53*	19	0.0366

*TIMM23*	2	0.0367

*GMNN*	3	0.0407

*AGT*	10	0.0436

Genes listed are those which have a statistically significant number of interactions according to Fisher's Exact Test with Holm-Bonferroni adjusted *p*-value < 0.05.

## Discussion

RA has the ability to induce differentiation and/or growth arrest in a variety of cancer cells [15]. However, the genes and pathways that mediate the biological effects of RA have not been fully elucidated. B16 mouse melanoma cells are very sensitive to RA treatment and respond by undergoing growth arrest and differentiation [16]. In this study we used mouse DNA microarrays to determine the time-dependent changes in gene expression in control vs. RA-treated B16 mouse melanoma cells. Our results show a small number of changes, mostly increases, in gene expression at early time points (4–10 h).

There was a major increase in the number of genes whose expression changed at 48 h of RA treatment and at this time point genes that were downregulated outnumbered those that were upregulated. To our knowledge, this is the first reported study of time-dependent changes in gene expression in RA-treated melanoma cells. Other studies have examined time-dependent gene expression changes in human embryonal carcinoma cells [27], F9 teratocarcinoma cells [28], and mouse skin [29] treated with RA. Similar to our results, a much larger number of genes had altered expression at 24–48 h of RA treatment. Comparison of the identity of RA-regulated genes in these studies with those reported here reveal some commonality of genes such as *RAR-β2*, *PKCα *and insulin-like growth factor binding protein 6, but also a number of different gene expression changes. Possible explanations for these differences are analysis of different cell types, use of different microarray platforms and different methods of statistical analysis.

In the hierarchical clustering analysis (Fig. 1) the 4 and 10 h RA-treated B16 cell-gene expression changes formed a tight cluster as do the 48 h RA-treated B16 cell gene changes. However, the changes in gene expression in 24 h RA-treated B16 cells did not form a cluster. This implies more variability in gene expression around this time of RA treatment, possibly because there are dramatic changes in gene expression around this time point.

The second microarray data set is a comparison between gene expression in untreated B16 mouse melanoma cells and melan-a mouse melanocytes. Both of these cell lines are derived from the same C57BL/6 inbred mouse strain. The melan-a cells have a longer replicative life-span than primary mouse melanocytes before they enter senescence [20]. Significant differences in expression are found in genes encoding cell cycle regulatory proteins, DNA metabolism, and DNA repair enzymes. There have been a number of studies comparing gene expression profiles in human melanocytes to human melanoma cell lines [30,31] and human nevus tissue to various stages of human melanoma tumors from patients [32,33], but very little information is available comparing expression profiles in mouse melanocytes with mouse melanoma cells. Despite the species difference, a number of genes which had altered expression in human melanocytes vs. human melanoma cells, such as *CDC2*, *C-MYC*, DNA repair enzymes and differentiation/embryonic markers, were found to be altered in the expression profiles between melan-a and B16 cells. Our findings suggest that B16 melanoma cells serve as a valid model for studying human melanoma

We sought to determine whether there were common genes between the microarray data set generated from RA treatment of B16 melanoma cells and the microarray data set comparing the gene expression of melan-a to B16 cells. We found that there were 233 genes in common between these two data sets. Within this group, the large majority, 203 genes, (87%) had their expression altered by RA treatment of B16 melanoma cells toward the levels found in melan-a cells. In general, RA treatment decreases the expression of genes involved in cell division/cell cycle, DNA replication and repair, and transcription regulation. It also decreases expression of the genes involved in DNA recombination and protein folding. In contrast, RA increases the expression of genes that regulate cell-cell signaling, cell adhesion and cell differentiation and development. Considering that RA inhibits cell replication and stimulates differentiation, these RA-induced changes in gene expression reflect the reprogramming of the B16 cells toward the melan-a (normal) phenotype. The reversion is not complete since some genes whose expression is decreased in B16 melanoma cells relative to melan-a cells (e.g. cell adhesion and extracellular matrix proteins) are not regulated by RA. Finally, we note that only three genes within the 203 gene set were differentially regulated by RA at 4 h. (1) *DHRS3*, a member of SDR family, encodes an enzyme which catalyzes the reduction of all *trans*-retinal to all-*trans*-retinol in the presence of NADPH and is highly expressed in the retina [34]. (2) Mouse *CAMK2Nl *mRNA is induced 2.2 fold by RA at 4 h and is downregulated 3.8 fold in B16 relative to melan-a cells. This protein exhibits strong similarity (97.44%) to the human CAMKIINα which has been shown to inhibit cell cycle progression in S phase in human colon adenocarcinoma cells [35]. (3) Although RIKEN *cDNA 3110001A13 *had significant homology to human, chimp, macaque and rat cDNAs (Blast search results not shown), no function has been ascribed to its predicted protein.

To determine the pathway(s) that mediate the effect of RA inhibition of cell proliferation and stimulation of differentiation, we used Pathway Studio v5.0 software to discover relationships between the proteins encoded by the 203 gene set. We found 74 genes (36%) whose encoded proteins have known connections either through physical interaction, regulation of gene expression or protein modification (e.g. phosphorylation). These interactions clustered around four major "hub" proteins: CDC2, CHEK1, CDC45L and MCM6. We note that even though these four proteins had small numbers of partners, by using the Fisher Exact Test we found that these hubs were statistically enriched for molecular interactions. In addition, three other proteins p53, p21 and CMYC appear to be important since they have large numbers of known interactions. Zhang and Rosdahl [36] found that RA treatment of human melanoma cells increased the expression of p53, while Vertuani et al. [37] observed p53 to be increased in RA-treated human uveal melanoma cells. Likewise there are numerous reports that RA alters the expression of p21^WAF-1/CIP-1 ^in a variety of tumor cells [38,39]. Gompel [40] found that RA decreases the expression and activity of CDK1 (CDC2) and there are a variety of studies showing that RA inhibits the expression of C-MYC [41,42]. In contrast, no evidence has been published that RA regulates CHEK1 gene expression. CHEK1 is involved in stopping cells from progressing through the cell cycle while DNA damage is repaired. Increased expression of this gene is frequently associated with resistance to chemotherapy [43]. A search of the PubMed database revealed no publications linking retinoic acid to the regulation of MCM6 or CDC45L. Further analysis of the time-dependent changes in RA-induced gene expression is needed to define the sequence of pathways that lead to RNA expression levels found in the 203 gene set.

## Conclusion

Our study has shown that RA regulates the expression of 4.8% of the total known mouse genes in B16 mouse melanoma cells over the course of 48 h of treatment. Alteration of the expression of these genes is associated with RA-induced growth arrest and differentiation of B16 mouse melanoma cells. We also found that ~13.7% of the total known mouse genes were differentially expressed in melan-a mouse melanocytes vs. B16 mouse melanoma cells. In comparing these two data sets, we found 233 common genes. Within this set, 203 genes in B16 melanoma cells had their RNA expression "normalized" toward the melan-a level by treatment with RA. Pathway analysis of the proteins encoded by this 203 data set revealed that 32% are involved in common interactions. The major hubs of connectivity are centered around CDC2, CHEK1, CDC45L and MCM6. It is likely that these four genes and their encoded proteins play a major role in RA-induced growth arrest and differentiation of B16 mouse melanoma cells.

## Methods

### Cell culture

B16 mouse melanoma cells obtained from the American Type Culture Collection were grown in DMEM (Invitrogen, Carlsbad, CA), supplemented with 10% bovine calf serum (Hyclone Laboratories, Logan, UT) at 37°C in a 5% CO_2_/95% air humidified atmosphere. Cells were treated for 4, 10, 24, and 48 h with 10 μM RA (Fluka Chemical Corp., Ronkonkoma, NY). Control cells received the solubilization vehicle, DMSO. All manipulations involving RA were conducted under subdued lighting or yellow lights in order to minimize photo-oxidation. Melan-a cells (non-malignant mouse melanocytes) were a generous gift from Dr. Dorothy Bennett (St. George's Hospital, London, England). They were grown in RPMI 1640 medium with 5% fetal bovine serum supplemented with 200 nM phorbol 12-myristate 13-acetate plus antibiotics.

### RNA extraction and DNA microarray analysis

Control and RA-treated B16 cells were harvested at 4, 10, 24, and 48 h of treatment. Melan-a and B16 cells were seeded at appropriate cell numbers so that at harvest (72 h after seeding) they would be at approximately the same degree of confluence on the tissue culture dishes. RNA was extracted using TRI Reagent (Sigma Chemical Co., St. Louis, MO) according to the manufacturer's suggested protocol. RNA quality was assessed by electrophoretic analysis on an Agilent Model 2100 Bioanalyzer (Santa Clara, CA). All RNA samples used for expression profiling had RNA Integrity Numbers greater than 9.

MWG Biotech (Ebersberg, Germany) Mouse 30K arrays were used to identify genes in B16 melanoma whose expression was altered by treatment with 10 μM RA for the various time periods using a balanced block design. All treatments were repeated 6 times to provide biological replicates for the microarray analysis. Labeled cDNAs were prepared from total RNA using the Invitrogen SuperScript II direct cDNA labeling system in the presence of either Cyanine-3 (Cy3) dCTP or Cyanine-5 (Cy5) dCTP (Perkin Elmer, Waltham, MA). For each time point, three of the arrays were hybridized with Cy3 labeled cDNA from the untreated cells and Cy5 labeled cDNA from treated cells. The remaining three arrays were hybridized with the labeling reversed to eliminate the effects of differential dye incorporation. The amounts of dye incorporated into cDNA were measured on a NanoDrop spectrophotometer (ThermoFisher Scientific, Pittsburgh, PA). Equimolar amounts of Cy3 and Cy5 labeled cDNAs were combined and added to MWG hybridization solution. This solution was heated for 3 min. at 95°C, cooled on ice for 3 min., and added to the microarray slide. Hybridizations were carried out for 16 h at 42°C on a GeneTAC hybridization station (Genomic Solutions, Ann Arbor, MI). Slides were washed on the automated hybridization station according to a Genomic Solutions protocol. Slides were scanned on a PerkinElmer ScanArray Express Microarray Scanner.

For the melan-a vs. B16 melanoma expression profiles, we employed the same balanced block design with a dye swap using six biological replicates. Equimolar amounts of the Cy3 and Cy5 labeled cDNAs were loaded onto Agilent Whole Mouse Genome Arrays, and hybridized for 17 h at 60°C using a MAUI hybridization system (BioMicroSystems, Salt Lake City, UT). The microarray slides were then washed using Agilent Gene Expression Wash Buffer and scanned using a Perkin Elmer ScanArray Express scanner.

### Statistical Analysis of Microarray Data

Feature intensities for the RA time course were extracted from the scanned image using PerkinElmer ScanArray software with the default lowess normalization settings; for melan-a vs. B16 comparisons, intensities were extracted using ImaGene software (BioDiscovery, El Segundo, CA). All extracted data were exported to Microsoft Excel (Microsoft Corporation, Redmond, WA) as tab delimited files. For each feature, a low intensity filter was applied in which data were only included if the total background-subtracted intensity of the two channels was greater than 400. The log base 2 of the expression ratios of RA-treated samples to untreated samples or the log base 2 of the expression ratios of melan-a samples to B16 samples were computed, and assembled into a single tab-delimited file for each comparison. Each file was imported to the Multiple Experiment Viewer (MeV) v4.0 [44] to perform statistical analysis.

Calculated log ratios were compared for significant deviation from zero using one-class Significance Analysis of Microarrays (SAM) [22]. This analysis was performed independently at each time point for the RA treatment experiments and for the melan-a vs. B16 experiment. In each case, only probes for which at least three of the six replicates passed the low intensity filter were included in the analysis. SAM was performed with the maximum number of unique permutations available, and delta values were chosen to give a median False Discovery Rate of 10%. All other parameters were set to the MeV defaults. Features found to be statistically significant by this analysis were subsequently filtered for a minimum fold change greater than 1.5. In both array platforms, a number of genes are represented by multiple probes. Additionally, a number of identical probes are represented as multiple features on the array. For genes which were determined to be significantly differentially expressed and which were represented by multiple features on the array, we report the feature with the largest fold change. In the melan-a vs. B16 comparison, three genes represented by multiple features were reported to be significantly differentially expressed with some features showing overexpression in melan-a and some showing underexpression in melan-a. These genes, which were not reported as significantly differentially expressed at any time point in the RA treatment comparison, were eliminated from the results as being internally inconsistent.

We compiled a set of all genes differentially regulated by RA at one or more time points and intersected this set with those genes differentially expressed in the melan-a vs. B16 cell experiment. We restricted this intersection to those genes which were upregulated by RA and overexpressed in melan-a cells OR which were downregulated by RA and underexpressed in melan-a cells. Hierarchical clustering was performed on the resulting gene set using MeV. Both gene clusters and sample clusters were computed using the Euclidean metric and average linkage. Microarray data may be accessed at the NCBI Gene Expression Omnibus (GEO) database (accession # GSE11588).

### Independent verification of gene expression changes

We selected seven genes from the 203 gene set to validate the microarray results. Total RNA was extracted from untreated and 48 h RA-treated B16 cells and melan-a cells as described above. qRT-PCR assays were used to measure expression of the selected genes. For qRT-PCR, total RNA was reverse transcribed into cDNA with the Advantage RT-for-PCR system (Clontech, Mountain View, CA) in the presence of random primers. The real time amplifications were carried out using a TaqMan Universal PCR Mastermix (ABI, Foster City, CA) and TaqMan probes specific for *BRCA2*, *CDC2A*, *TINAGL*, *PLAT*, *TOP2A*, *DHRS3 *and *FGF1*. All amplifications were performed on an ABI Model 7000 Sequence Detection System with the following cycles: 95°C for 10 min, and 40 cycles of 95°C for 15 s and 60°C for 1 min. Each assay was performed in triplicate and resulting qRT-PCR data was analyzed used a modification of the ΔΔCt method that accounts for variations in primer efficiency [45]. 18S rRNA was used as the reference gene for all normalizations. *p *values were computed for the RA-treated and melan-a ΔCt values compared by T test to the untreated B16 ΔCt values and corrected by Bonferroni correction for multiple hypothesis testing.

### Pathway analysis

In order to identify molecular interactions among the protein products of 203 genes, we entered the expression data from these genes into Pathway Studio software [Rockville, MD] and set the analysis for the identification of direct interactions within the gene set. We used Fisher's Exact Test as implemented by Pathway Studio to determine the *p*-value associated with the biological processes, and subsequently adjusted for multiple hypothesis testing using the Holm-Bonferroni method [46] with an overall type-1 error rate of 0.05. In order to determine statistical significance of the "hubs" in the graphical output, we directly implemented Fisher's Exact Test with the same parameters using custom-written Java code. The Holm-Bonferroni adjusted *p*-values reported for the hubs indicate the significance level for the number of connections to other genes in our set, accounting for the number of known connections in the Pathway Studio database.

## Competing interests

The authors declare that they have no competing interests.

## Authors' contributions

ME conducted the time-course treatment of B16 melanoma cell with RA, and did the classification of RA-regulated genes for biological function. GB conducted all of the microarray experiments, carried out the data extraction and performed the pathway interaction analysis. JD was responsible for the statistical analysis of significance of all gene expression changes on the microarrays, as well as the hierarchical clustering. SM performed all the experiments using qRT-PCR to confirm the microarray results for the seven selected genes. DP had overall responsibility for the execution of the microarray assays and statistical analyses of the data. RMN wrote the manuscript and was responsible for the overall design of the project and data interpretation. All authors read and approved the final manuscript.

## Supplementary Material

Additional file 1 **List of genes whose expression is significantly regulated by RA treatment at one or more time point.** Statistical significance is determined by SAM with a false discovery rate of 10%, and a minimum fold change of 1.5. Average fold changes across six replicates are reported for significant changes only. Gene IDs (first column) are linked to search pages at the National Center for Biotechnology Information. Click here for file

Additional file 2 **List of genes whose expression is significantly different between the melan-a and B16 cell lines.** Statistical significance is determined by SAM with a false discovery rate of 10% and a minimum fold change of 1.5. Average fold changes across six replicates are reported. Gene IDs (first column) are linked to search pages at the National Center for Biotechnology Information. Click here for file

Additional file 3 **Members of the 203 gene set, fold change at 48 h of RA treatment and relative expression in melan-a vs.** B16 cells. Click here for file

Additional file 4 Classification of 203 Gene Set Members According to Gene Ontology Biological Processes. Processes were ranked by p-value which is the Holm-Bonferroni adjusted probability of a random set of 203 genes containing at least the stated representation in the Pathway Studio database. The number of members within each process that are expressed lower in melan-a, higher in melan-a and the total number of members are provided in columns 2, 3 and 4 respectively. Click here for file

Additional file 5 **Members of 203 gene set which are components of the major biological processes.** Using Pathway Studio, we identified biological processes which were enriched for members of the 203 gene set. Members of the five processes that are most significantly enriched according to Fisher's Exact Test are given. Click here for file
