# Supplementary material for: Global analysis of gene expression changes during retinoic acid-induced growth arrest and differentiation of melanoma: comparison to differentially expressed genes in melanocytes vs melanoma
Source: BMC Genomics. 2008 Oct 11;9:478. doi: 10.1186/1471-2164-9-478 (PMC2572629; doi:10.1186/1471-2164-9-478)
Supplement: Additional file 1 — List of genes whose expression is significantly regulated by RA treatment at one or more time point. Statistical significance is determined by SAM with a false discovery rate of 10%, and a minimum fold change of 1.5. Average fold changes across six replicates are reported for significant changes only. Gene IDs (first column) are linked to search pages at the National Center for Biotechnology Information. [file 1471-2164-9-478-S1.htm]

Supplemental Table 1.


Additional file 1. List of genes whose expression is
significantly regulated by RA treatment at one or more time point. Statistical
significance was determined by SAM with a false discovery rate of 10%, and a
minimum fold change of 1.5. Average fold changes across six replicates are
reported for significant changes only. Gene IDs (first column) are linked to
search pages at the National Center for
Biotechnology Information.

| ID | Description | 4 hr fold change | 10 hr fold change | 24 hr fold change | 48 hr fold change |
| --- | --- | --- | --- | --- | --- |
| NM\_031185 | a kinase (prka) anchor protein (gravin) 12; akap12 | - | - | 8.56 | 21.86 |
| AB020886 | ssecks | - | - | 8.22 | 13.28 |
| ENSMUSG00000026256 | ENSMUST00000027474 inward rectifier potassium channel potassium channel; inwardly rectifying; subfamily j; member inward rectifier k+ channel [source:ensembl\_protein\_families;acc:ensmusf00000000160]; inward rectifier potassium channel potassium channel; i | - | - | 10.80 | 11.03 |
| AK019535 | data source:sptr; source key:q9h9y1; evidence:iss homolog to cdna flj12476 fis; clone nt2rm1000978 putative | - | - | - | 10.06 |
| NM\_011303 | retinal short-chain dehydrogenase/reductase 1; rsdr1-pending | 7.94 | 2.97 | 5.63 | 4.64 |
| BC010972 | similar to retinal short-chain dehydrogenase/reductase 1 | 7.86 | 3.52 | 6.20 | 5.20 |
| NM\_019919 | latent transforming growth factor beta binding protein 1; ltbp1 | - | - | 3.23 | 7.52 |
| NM\_008131 | glutamine synthetase; glns | - | - | 2.78 | 7.04 |
| ENSMUSG00000037582 | ENSMUST00000042029 nck associated protein 5 nap 5 [source:ensembl\_protein\_families;acc:ensmusf00000006826]; nck associated protein 5 nap 5 | - | - | 4.16 | 6.89 |
| AK010084 | homolog to kiaa0830 protein (fragment) | - | - | - | 6.80 |
| NM\_019861 | cathepsin f; ctsf | - | - | - | 6.50 |
| AF280604 | latent transforming growth factor beta binding protein-1s precursor | - | - | 4.15 | 6.24 |
| NM\_028889 | riken cdna 4931430i01; 4931430i01rik | - | - | 5.35 | 5.74 |
| BC021496 | atpase; na+k+ transporting; alpha 1 polypeptide | - | - | 4.73 | 5.73 |
| NM\_130859 | bimp1; ai449026 | - | - | 3.36 | 5.70 |
| NM\_011782 | a disintegrin-like and metalloprotease (reprolysin type) with thrombospondin type 1 motif; 5 (aggrecanase-2); adamts5 | - | - | - | 4.91 |
| NM\_011101 | protein kinase c; alpha; prkca | - | - | 3.54 | 4.87 |
| ENSMUSG00000017491 | ENSMUST00000037779 retinoic acid receptor beta (rar-beta). [source:swissprot;acc:p22605]; retinoic acid receptor rar | - | - | 4.06 | 4.83 |
| ENSMUSG00000023902 | ENSMUST00000024693 zinc finger protein [source:ensembl\_protein\_families;acc:ensmusf00000000001]; zinc finger protein | - | - | - | 4.72 |
| NM\_008486 | alanyl (membrane) aminopeptidase; anpep | 2.77 | 3.60 | 4.66 | 4.65 |
| NM\_026672 | riken cdna 0610005a07; 0610005a07rik | - | - | 4.66 | 2.40 |
| ENSMUSG00000029943 | ENSMUST00000032005 unknown | - | - | - | 4.63 |
| NM\_010357 | glutathione-s-transferase; alpha 4; gsta4 | - | - | - | 4.34 |
| AY073618 | olfactory receptor mor170-8 | - | - | 1.96 | 4.29 |
| ENSMUSG00000038332 | ENSMUST00000041438 sestrin 1 (p53-regulated protein pa26). [source:swissprot;acc:p58006]; sestrin | - | - | 3.78 | 4.29 |
| AB006361 | prostaglandin d synthetase | - | - | - | 4.27 |
| NM\_022995 | nedd4 ww binding# protein 4; n4wbp4-pending | - | 1.96 | 2.23 | 4.26 |
| NM\_030258 | hypothetical protein; mgc:7035; bc003323 | - | - | 4.19 | 2.02 |
| ENSMUSG00000042207 | ENSMUST00000047714 unknown | - | - | 4.18 | 2.51 |
| NM\_011599 | transducin-like enhancer of split 1; homolog of drosophila e(spl); tle1 | - | - | 4.17 | 2.11 |
| ENSMUSG00000030441 | ENSMUST00000036975 zinc finger protein [source:ensembl\_protein\_families;acc:ensmusf00000000001]; zinc finger protein | - | - | 2.63 | 4.13 |
| BC006800 | similar to phosphoprotein regulated by mitogenic pathways | - | - | 2.09 | 4.08 |
| AK013580 | similar to mrna; complete cds; clone:1-82 | - | - | 2.31 | 4.05 |
| BC025088 | unknown (protein for image:3964696) | - | 1.88 | 3.11 | 4.00 |
| ENSMUSG00000034614 | ENSMUST00000045153 ambiguous [source:ensembl\_protein\_families;acc:ensmusf00000009513]; ambiguous | - | - | - | 3.98 |
| ENSMUSG00000026939 | ENSMUST00000028308 unknown | - | - | 3.86 | 3.97 |
| NM\_021278 | thymosin; beta 4; x chromosome; tmsb4x | - | - | - | 3.96 |
| BC014284 | unknown (protein for image:3666877) | - | - | 3.15 | 3.93 |
| AY073227 | olfactory receptor mor114-4 | - | - | - | 3.92 |
| ENSMUSG00000018166 | ENSMUST00000026423 receptor protein tyrosine kinase erbb ec 2.7.1.112 [source:ensembl\_protein\_families;acc:ensmusf00000000300]; receptor protein tyrosine kinase erbb ec 2.7.1.112 | - | - | - | 3.85 |
| ENSMUSG00000030522 | ENSMUST00000032736 unknown | - | 1.75 | 2.90 | 3.85 |
| ENSMUSG00000030185 | ENSMUST00000032300 zinc finger protein [source:ensembl\_protein\_families;acc:ensmusf00000000001]; zinc finger protein | - | - | 3.46 | 3.81 |
| AK005535 | homolog to cdna flj20327 fis; clone hep10012 | - | - | 2.85 | 3.79 |
| NM\_009699 | aquaporin 2; aqp2 | - | - | 3.73 | 3.39 |
| AY073819 | olfactory receptor mor245-21 | - | - | 3.73 | 2.95 |
| NM\_023476 | lipocalin 7; lcn7 | - | - | 3.70 | - |
| ENSMUSG00000032664 | ENSMUST00000040586 transporting atpase alpha chain ec pump atpase [source:ensembl\_protein\_families;acc:ensmusf00000000134]; transporting atpase alpha chain ec pump atpase | - | - | 3.66 | - |
| AF455111 | ataxin-7; sca7 | - | 2.22 | 3.66 | 2.82 |
| AK006300 | homolog to ba108l7.8 (novel protein (translation of cdna flj10512 (em:ak001374))) | - | - | - | 3.64 |
| NM\_009923 | cyclic nucleotide phosphodiesterase 1; cnp1 | - | - | - | 3.62 |
| NM\_013616 | olfactory receptor 64; olfr64 | - | - | - | 3.60 |
| AJ237586 | hypothetical protein | - | - | 2.39 | 3.58 |
| AK014532 | kelch motif containing protein | - | - | 2.14 | 3.48 |
| AY073671 | olfactory receptor mor218-3 | - | - | 2.73 | 3.47 |
| S56660 | retinoic acid nuclear receptor isoform beta 2; retinoic acid nuclear receptor isoform > | 3.47 | 2.60 | 2.53 | 3.23 |
| NM\_009263 | secreted phosphoprotein 1; spp1 | - | - | 3.00 | 3.46 |
| AY073036 | olfactory receptor mor32-3 | - | - | 2.10 | 3.46 |
| NM\_020599 | retinaldehyde-binding protein 1; rlbp1 | - | - | - | 3.46 |
| AK008738 | cdna clone homolog to plu-1 protein | - | - | 3.46 | 2.43 |
| ENSMUSG00000037147 | ENSMUST00000039827 fragile x mental retardation 2 protein fmr 2 fmr2p ox19 [source:ensembl\_protein\_families;acc:ensmusf00000006942]; fragile x mental retardation 2 protein fmr 2 fmr2p ox19 | - | - | 3.44 | 2.43 |
| ENSMUSG00000035735 | ENSMUST00000039327 protein [source:ensembl\_protein\_families;acc:ensmusf00000007986]; protein | - | - | - | 3.41 |
| BC024955 | unknown (protein for mgc:37148) | - | - | - | 3.40 |
| AY072976 | olfactory receptor mor8-3 | - | - | - | 3.36 |
| NM\_016894 | receptor-activity modifying protein 1; ramp1 | - | - | - | 3.36 |
| S82796 | carnitine palmitoyltransferase i; cpt i | - | - | - | 3.33 |
| NM\_053146 | protocadherin beta 21; pcdhb21 | - | - | - | 3.32 |
| NM\_023056 | lr8 protein; 1810009m01rik | - | - | 1.96 | 3.32 |
| NM\_133732 | riken cdna 4931406c07; 4931406c07rik | - | - | 3.32 | 3.18 |
| AK007352 | cdna clone hypothetical protein | 2.22 | 2.15 | - | 3.31 |
| BC025037 | unknown (protein for image:4953771) | - | - | 2.79 | 3.30 |
| NM\_008735 | nuclear receptor interacting protein 1; nrip1 | - | 2.10 | 3.29 | - |
| AK007905 | tbc domain containing protein data source:pfam; source key:pf00566; evidence:iss putative | - | - | - | 3.29 |
| X16998 | protein sequence is in conflict with the conceptual translation | - | - | - | 3.28 |
| NM\_009324 | t-box 2; tbx2 | 2.15 | 2.28 | 3.11 | 3.25 |
| NM\_007709 | cbp/p300-interacting transactivator with glu/asp-rich carboxy-terminal domain 1; cited1 | - | - | 2.62 | 3.24 |
| NM\_009115 | s100 protein; beta polypeptide; neural; s100b | - | - | - | 3.23 |
| NM\_011481 | src-related kinase lacking c-terminal regulatory tyrosine and n-terminal myristylation sites; srms | - | - | 3.22 | 2.08 |
| NM\_013722 | synapsin 3; syn3 | - | - | 3.15 | - |
| AY073209 | olfactory receptor mor267-2 | - | - | - | 3.13 |
| AF071001 | phr1 | - | - | - | 3.07 |
| NM\_018812 | protein inhibitor of activated stat3; pias3 | - | - | - | 3.07 |
| NM\_030255 | hypothetical protein; mgc:7002; bc003314 | - | - | - | 3.05 |
| AB027197 | sialyltransferase | - | - | 3.05 | 2.28 |
| ENSMUSG00000022714 | ENSMUST00000023399 4 aminobutyrate aminotransferase; mitochondrial precursor ec 2.6.1.19 gamma amino n butyrate transaminase gaba transaminase gaba aminotransferase gaba [source:ensembl\_protein\_families;acc:ensmusf00000003292]; 4 aminobutyrate aminotransf | - | - | 2.49 | 3.03 |
| U36575 | t cell transcription factor nfat1 isoform b | 3.02 | - | - | - |
| ENSMUSG00000030163 | ENSMUST00000032266 c-type (calcium dependent; carbohydrate recognition domain) lectin; superfamily member 12. [source:refseq;acc:nm\_020008]; beta glucan receptor isoform | - | - | 3.00 | - |
| AK002546 | homolog to hepatocellular carcinoma-associated antigen 112 | - | - | 2.83 | 3.00 |
| BC008695 | unknown (protein for image:3707880) | - | 1.89 | 3.00 | 2.75 |
| AK013035 | hypothetical protein | - | - | - | 3.00 |
| ENSMUSG00000042473 | ENSMUST00000046267 unknown | - | - | 2.13 | 2.99 |
| L24755 | bone morphogenetic protein; bmp-1 | - | - | - | 2.98 |
| ENSMUSG00000020085 | ENSMUST00000020282 unknown | 1.76 | 2.06 | 2.98 | 2.19 |
| M16472 | proteolipid protein variant dm-20 | - | - | 2.97 | 2.60 |
| NM\_009754 | bcl2 interacting mediator of cell death; bcl2l11 | - | - | - | 2.94 |
| NM\_007845 | defensin related cryptdin; related sequence 10; defcr-rs10 | - | - | - | 2.93 |
| AL354805 | dm544j17.1 (novel protein similar to rat rhogap); dm544j17.1 | - | - | - | 2.92 |
| M13138 | gag-myb protein | - | - | - | 2.92 |
| ENSMUSG00000042214 | ENSMUST00000047823 unknown | - | - | 1.72 | 2.92 |
| ENSMUSG00000027476 | ENSMUST00000028978 f4 4 novel protein similar to nucleolar protein 4 nol4 [source:ensembl\_protein\_families;acc:ensmusf00000011493]; f4 4 novel protein similar to nucleolar protein 4 nol4 | - | - | - | 2.91 |
| ENSMUSG00000039157 | ENSMUST00000048375 unknown | - | - | - | 2.91 |
| ENSMUSG00000038375 | ENSMUST00000043237 n3 1 similar to collagen [source:ensembl\_protein\_families;acc:ensmusf00000012800]; n3 1 similar to collagen | - | - | - | 2.91 |
| AK005168 | homolog to intestinal membrane a4 protein (differentiation-dependent protein a4) (proteolipid protein 2) | - | - | - | 2.89 |
| M14222 | mouse preprocathepsin b | - | - | - | 2.89 |
| NM\_053141 | protocadherin beta 16; pcdhb16 | - | - | - | 2.89 |
| ENSMUSG00000034588 | ENSMUST00000038915 unknown | - | - | 2.88 | - |
| NM\_011862 | protein kinase c and casein kinase substrate in neurons 2; pacsin2 | - | 1.52 | - | 2.87 |
| AF189772 | gamma adducin | 1.53 | 2.87 | - | - |
| AK015239 | btb/poz domain containing protein | - | - | 2.86 | - |
| NM\_018733 | sodium channel; voltage-gated; type i; alpha polypeptide; scn1a | - | - | - | 2.85 |
| ENSMUSG00000039879 | ENSMUST00000037879 homolog of drosophila headcase dj225e12 1 homolog of drosophila [source:ensembl\_protein\_families;acc:ensmusf00000011404]; homolog of drosophila headcase dj225e12 1 homolog of drosophila | - | - | - | 2.84 |
| BC003808 | similar to testin | - | - | - | 2.84 |
| NM\_019909 | mhc (a.ca/j(h-2k-f) class i antigen; loc56628 | - | - | 2.84 | 1.69 |
| BC026896 | unknown (protein for image:3982506) | - | - | 2.82 | - |
| AK004692 | sorbitol dehydrogenase 1 | - | - | 2.82 | - |
| BC020028 | similar to hypothetical protein flj13204 | - | - | - | 2.82 |
| ENSMUSG00000028300 | ENSMUST00000047686 ambiguous [source:ensembl\_protein\_families;acc:ensmusf00000008408]; ambiguous | - | - | - | 2.82 |
| NM\_009950 | casp2 and ripk1 domain containing adaptor with death domain; cradd | - | - | 2.80 | - |
| BC006779 | unknown (protein for image:3589116) | - | - | 2.51 | 2.79 |
| BC004727 | similar to loss of heterozygosity; 11; chromosomal region 2; gene a | - | - | - | 2.79 |
| AK019677 | synj2 | - | - | - | 2.79 |
| NM\_011337 | small inducible cytokine a3; scya3 | - | - | - | 2.79 |
| AF012926 | fibroblast growth factor-1 | - | 1.84 | 2.11 | 2.79 |
| NM\_011340 | serine (or cysteine) proteinase inhibitor; clade f (alpha-2 antiplasmin; pigment epithelium derived factor); member 1; serpinf1 | - | - | 2.79 | 1.71 |
| BC008155 | unknown (protein for mgc:6974) | - | - | - | 2.78 |
| AK010477 | homolog to dna polymerase delta smallest subunit p12 | - | - | - | 2.78 |
| U49952 | yrk | - | - | 2.76 | 2.41 |
| ENSMUSG00000020598 | ENSMUST00000020942 precursor [source:ensembl\_protein\_families;acc:ensmusf00000000276]; precursor | - | - | - | 2.76 |
| AF114382 | four and half lim domain protein 3; fhl3 | - | - | 2.76 | - |
| ENSMUSG00000038178 | ENSMUST00000042570 unknown | - | - | 2.18 | 2.75 |
| NM\_011595 | tissue inhibitor of metalloproteinase 3; timp3 | - | - | - | 2.74 |
| NM\_009223 | stannin; snn | - | - | - | 2.74 |
| ENSMUSG00000042687 | ENSMUST00000043424 adamts ec 3.4.24.- a disintegrin and metalloproteinase with thrombospondin motifs adam ts adam [source:ensembl\_protein\_families;acc:ensmusf00000000447]; adamts ec 3.4.24.- a disintegrin and metalloproteinase with thrombospondin motifs a | - | - | - | 2.73 |
| ENSMUSG00000023857 | ENSMUST00000024633 protein [source:ensembl\_protein\_families;acc:ensmusf00000012195]; protein | - | - | 2.73 | 2.01 |
| NM\_009155 | selenoprotein p; plasma; 1; sepp1 | - | - | - | 2.72 |
| NM\_013490 | choline kinase; chk | - | - | 2.12 | 2.71 |
| ENSMUSG00000034704 | ENSMUST00000045979 protocadherin precursor pcdh [source:ensembl\_protein\_families;acc:ensmusf00000000021]; protocadherin precursor pcdh | - | - | 2.71 | 2.15 |
| ENSMUSG00000041471 | ENSMUST00000035262 ambiguous [source:ensembl\_protein\_families;acc:ensmusf00000007895]; ambiguous | - | - | - | 2.69 |
| AF215669 | regulator of g-protein signaling 3s | - | - | - | 2.69 |
| ENSMUSG00000034074 | ENSMUST00000037202 protein kinase [source:ensembl\_protein\_families;acc:ensmusf00000000126]; protein kinase | - | - | - | 2.69 |
| ENSMUSG00000040296 | ENSMUST00000037896 rna [source:ensembl\_protein\_families;acc:ensmusf00000005662]; rna | - | - | - | 2.69 |
| L37058 | gag | - | - | - | 2.69 |
| AK014548 | homolog to putative ras inhibitor (fragment) | 2.69 | - | 1.93 | 1.95 |
| ENSMUSG00000001630 | ENSMUST00000037319 myotonin protein kinase ec 2.7.1.- myotonic dystrophy protein kinase mdpk dm kinase dmk dmpk mt [source:ensembl\_protein\_families;acc:ensmusf00000000307]; myotonin protein kinase ec 2.7.1.- myotonic dystrophy protein kinase mdpk dm kinas | - | - | - | 2.68 |
| AK003542 | homolog to exchange factor for arf6 | - | - | - | 2.68 |
| ENSMUSG00000038694 | ENSMUST00000049334 unknown | - | - | - | 2.68 |
| NM\_009366 | transforming growth factor beta 1 induced transcript 4; tgfb1i4 | - | - | - | 2.67 |
| BC003903 | similar to glutathione s-transferase theta 1 | - | - | 2.15 | 2.66 |
| NM\_015825 | sh3-binding domain glutamic acid-rich protein; sh3bgr | - | - | - | 2.66 |
| ENSMUSG00000028709 | ENSMUST00000035267 unknown | - | - | - | 2.66 |
| AF288694 | ube1l | - | - | - | 2.65 |
| AK006096 | hypothetical protein | - | - | - | 2.65 |
| NM\_007798 | cathepsin b; ctsb | - | - | 2.65 | 2.60 |
| NM\_019676 | phospholipase c; delta; plcd | - | - | 2.64 | 2.48 |
| AF416923 | brown fat inducible thioesterase 2 | - | - | - | 2.63 |
| NM\_008321 | inhibitor of dna binding 3; idb3 | - | - | - | 2.63 |
| NM\_023128 | paralemmin; palm | - | - | 2.61 | - |
| BC011344 | unknown (protein for mgc:5739) | - | - | - | 2.60 |
| AJ307670 | suppression of tumorigenicity 5; st5 | - | - | - | 2.59 |
| ENSMUSG00000034840 | ENSMUST00000036534 unknown | - | - | - | 2.59 |
| AJ344065 | hypothetical protein; tes | - | - | 2.59 | 2.59 |
| BC016208 | similar to hypothetical protein flj22237 | - | - | - | 2.58 |
| BC011490 | similar to cholinergic receptor; nicotinic; alpha polypeptide 2 (neuronal) | - | - | - | 2.57 |
| NM\_009646 | autoimmune regulator (autoimmune polyendocrinopathy candidiasis ectodermal dystrophy); aire | - | - | - | 2.57 |
| AK005954 | hypothetical protein | - | - | - | 2.56 |
| NM\_019819 | dual specificity phosphatase 14; dusp14 | - | - | - | 2.56 |
| NM\_010260 | guanylate nucleotide binding protein 2; gbp2 | - | - | - | 2.56 |
| AK003346 | data source:sptr; source key:q9h3t5; evidence:iss homolog to mob1 protein (hypothetical 25.1 kda protein) putative | - | - | - | 2.56 |
| NM\_010476 | hydroxysteroid 17-beta dehydrogenase 7; hsd17b7 | - | - | 2.55 | - |
| NM\_011770 | zinc finger protein; subfamily 1a; 2 (helios); znfn1a2 | - | - | - | 2.54 |
| NM\_018809 | pancreas specific transcription factor; 1a; ptf1a | - | - | - | 2.54 |
| AF022858 | neuropilin-2(b5) | - | 1.62 | 2.51 | 2.54 |
| M14674 | myelin proteolipid | - | - | - | 2.54 |
| NM\_024479 | hypothetical protein; mgc: 8159; bc002286 | - | - | 2.54 | - |
| AK013666 | homolog to intestinal membrane a4 protein (differentiation-dependent protein a4) (proteolipid protein 2) | - | - | - | 2.52 |
| NM\_053262 | hydroxysteroid 17-beta dehydrogenase 11; hsd17b11 | - | - | - | 2.52 |
| NM\_030259 | hypothetical protein; mgc:7036; bc003324 | - | - | 2.52 | 1.99 |
| NM\_007899 | extracellular matrix protein 1; ecm1 | - | - | - | 2.51 |
| NM\_026415 | riken cdna 2310002j15; 2310002j15rik | - | - | - | 2.50 |
| NM\_015784 | osteoblast specific factor 2 (fasciclin i-like); osf2-pending | - | - | - | 2.50 |
| BC016215 | similar to hypothetical protein flj13710 | - | - | - | 2.50 |
| AK006661 | hypothetical protein | - | - | - | 2.49 |
| AK011324 | hypothetical protein | - | - | 2.48 | 2.27 |
| AK007582 | evidence:nas hypothetical protein putative | - | - | 2.47 | - |
| BC003974 | cdna clone; unknown | - | - | 1.94 | 2.46 |
| ENSMUSG00000034168 | ENSMUST00000038433 unknown | - | - | 2.45 | 2.40 |
| ENSMUSG00000042522 | ENSMUST00000041903 60s ribosomal protein l7a surfeit locus protein [source:ensembl\_protein\_families;acc:ensmusf00000000361]; 60s ribosomal protein l7a surfeit locus protein | - | - | - | 2.45 |
| NM\_021344 | tescalcin; tesc-pending | - | - | - | 2.45 |
| ENSMUSG00000038428 | ENSMUST00000044359 unknown | - | - | - | 2.43 |
| NM\_030697 | riken cdna 0610013d04 gene; 0610013d04rik | - | - | - | 2.43 |
| NM\_009177 | sialyltransferase 4; siat4a | - | - | - | 2.43 |
| NM\_021318 | activator of crem in testis; act-pending | - | - | - | 2.43 |
| BC016192 | similar to bicaudal d (drosophila) homolog 1 | - | - | - | 2.42 |
| ENSMUSG00000030397 | ENSMUST00000003646 protein kinase [source:ensembl\_protein\_families;acc:ensmusf00000000126]; protein kinase | - | - | - | 2.42 |
| ENSMUSG00000020061 | ENSMUST00000005246 myosin binding protein c; type mybp c c protein; muscle [source:ensembl\_protein\_families;acc:ensmusf00000000570]; myosin binding protein c; type mybp c c protein; muscle | - | - | - | 2.41 |
| AK007703 | homolog to atp-binding cassette; sub-family a; member 3 (atp-binding cassette transporter 3) (atp-binding cassette 3) (abc-c transporter) | - | - | - | 2.41 |
| AF487348 | down syndrome cell adhesion molecule-like protein; dscaml1 | - | - | - | 2.40 |
| AF029694 | extracellular matrix protein; ecm1 | - | - | - | 2.40 |
| ENSMUSG00000034372 | ENSMUST00000045088 k20d4. [source:sptrembl;acc:q9epx3]; unknown | - | - | - | 2.39 |
| ENSMUSG00000024763 | ENSMUST00000037251 ambiguous [source:ensembl\_protein\_families;acc:ensmusf00000000614]; ambiguous | - | - | - | 2.39 |
| AK003407 | data source:sptr; source key:p26376; evidence:iss homolog to rat interferon-inducible protein putative | - | - | 2.39 | 2.18 |
| AK018926 | hypothetical protein | - | - | - | 2.39 |
| BC025917 | similar to inositol 1;3;4-triphosphate 5/6 kinase | - | - | 1.89 | 2.39 |
| BC004808 | similar to signal transducer and activator of transcription 1 | - | - | - | 2.38 |
| ENSMUSG00000036345 | ENSMUST00000040346 unknown | - | - | - | 2.38 |
| ENSMUSG00000025477 | ENSMUST00000026550 type i inositol 1;4;5 trisphosphate 5 phosphatase ec 3.1.3.56 [source:ensembl\_protein\_families;acc:ensmusf00000006164]; type i inositol 1;4;5 trisphosphate 5 phosphatase ec 3.1.3.56 | - | - | - | 2.37 |
| BC018397 | similar to cellular retinoic acid-binding protein 2 | - | - | 2.37 | - |
| NM\_007484 | ras homolog 9 (rhoc); arhc | - | - | - | 2.37 |
| AF167552 | taj-alpha long | - | - | - | 2.37 |
| BC019749 | unknown (protein for image:5134560) | - | - | - | 2.37 |
| X15052 | neural cell adhesion molecule ncam-180 | - | - | - | 2.37 |
| ENSMUSG00000003710 | ENSMUST00000003807 cytokine receptor-like factor 1; cytokine receptor like molecule 3. [source:refseq;acc:nm\_018827]; cytokine | - | - | - | 2.36 |
| ENSMUSG00000035916 | ENSMUST00000043930 protein tyrosine phosphatase; non receptor type ec 3.1.3.48 protein tyrosine phosphatase [source:ensembl\_protein\_families;acc:ensmusf00000000067]; protein tyrosine phosphatase; non receptor type ec 3.1.3.48 protein tyrosine phosphatase | - | - | - | 2.36 |
| NM\_013697 | transthyretin; ttr | - | - | - | 2.36 |
| ENSMUSG00000036175 | ENSMUST00000041411 ig kappa chain v region [source:ensembl\_protein\_families;acc:ensmusf00000000033]; ig kappa chain v region | - | - | 2.35 | 1.75 |
| AK005274 | homolog to hydroxyacylglutathione hydrolase (ec 3.1.2.6) (glyoxalase ii) (glx ii) | - | - | - | 2.35 |
| NM\_009642 | angiotensin ii receptor; type i; agtrap | - | - | 2.26 | 2.35 |
| ENSMUSG00000036792 | ENSMUST00000047413 ambiguous [source:ensembl\_protein\_families;acc:ensmusf00000007913]; ambiguous | - | - | - | 2.35 |
| BC006873 | similar to hypothetical protein flj20174 | - | - | 2.34 | - |
| NM\_009543 | zinc finger protein 103; zfp103 | - | - | - | 2.34 |
| NM\_133978 | expressed sequence ai481279; ai481279 | - | - | 2.27 | 2.34 |
| AK002382 | ptgds | - | - | - | 2.33 |
| ENSMUSG00000036475 | ENSMUST00000038771 ambiguous [source:ensembl\_protein\_families;acc:ensmusf00000009770]; ambiguous | - | - | - | 2.32 |
| NM\_013866 | hematopoietic zinc finger; hzf-pending | - | - | - | 2.32 |
| NM\_021534 | pxmp4 | - | - | - | 2.31 |
| NM\_008171 | glutamate receptor; ionotropic; nmda2b (epsilon 2); grin2b | - | - | - | 2.31 |
| NM\_030714 | deltex3; dtx3 | - | - | - | 2.31 |
| NM\_026233 | riken cdna 4933434i20; 4933434i20rik | - | - | - | 2.30 |
| BC019446 | unknown (protein for image:4023951) | - | - | - | 2.29 |
| BC025850 | similar to wap four-disulfide core domain 1 | - | - | - | 2.29 |
| NM\_008655 | growth arrest and dna-damage-inducible 45 beta; gadd45b | - | - | - | 2.29 |
| BC011431 | similar to hemk homolog 7kb | - | - | 2.29 | - |
| AK002477 | homolog to plasmolipin | - | - | - | 2.29 |
| NM\_016718 | ninjurin 2; ninj2 | - | - | - | 2.29 |
| ENSMUSG00000037572 | ENSMUST00000043788 acidic nucleoplasmic dna binding protein 1 and [source:ensembl\_protein\_families;acc:ensmusf00000009348]; acidic nucleoplasmic dna binding protein 1 and | - | - | 2.29 | - |
| D14859 | drosophila vasa homologue | - | - | - | 2.29 |
| ENSMUSG00000042003 | ENSMUST00000025108 unknown | - | - | - | 2.28 |
| U33323 | corticotropin-releasing hormone-binding protein | - | - | - | 2.28 |
| NM\_018804 | synaptotagmin 11; syt11 | - | - | 2.28 | 2.03 |
| NM\_009134 | sodium channel; voltage-gated; type x; alpha polypeptide; scn10a | - | - | 2.27 | 1.72 |
| M55512\_2 | long orf | - | - | - | 2.27 |
| NM\_010638 | kruppel-like factor 9; klf9 | - | - | 2.26 | - |
| NM\_009242 | secreted acidic cysteine rich glycoprotein; sparc | - | - | - | 2.26 |
| AB047978 | calmin alpha | - | - | 2.26 | - |
| ENSMUSG00000040430 | ENSMUST00000040371 retinal degeneration b [source:ensembl\_protein\_families;acc:ensmusf00000019497]; retinal degeneration b | - | - | - | 2.26 |
| AB030442 | cas and hef1 associated signal transducer; chat | - | - | - | 2.26 |
| ENSMUSG00000041969 | ENSMUST00000048394 unknown | - | - | - | 2.26 |
| ENSMUSG00000037043 | ENSMUST00000048939 unknown | - | - | - | 2.25 |
| AF101053 | phr1 isoform 2 | - | - | - | 2.24 |
| NM\_019789 | calsenilin; presenilin-binding protein; ef hand transcription factor; csen | - | - | - | 2.24 |
| ENSMUSG00000036046 | ENSMUST00000047144 protein [source:ensembl\_protein\_families;acc:ensmusf00000011190]; protein | - | - | - | 2.24 |
| ENSMUSG00000033048 | ENSMUST00000040296 unknown | - | - | - | 2.23 |
| ENSMUSG00000036365 | ENSMUST00000038258 ambiguous [source:ensembl\_protein\_families;acc:ensmusf00000012349]; ambiguous | - | - | - | 2.23 |
| NM\_010591 | jun oncogene; jun | - | - | - | 2.23 |
| NM\_008116 | gamma-glutamyl transpeptidase; ggtp | - | - | - | 2.22 |
| BC026867 | similar to kiaa1145 protein | - | - | - | 2.22 |
| NM\_021272 | brain lipid binding protein; fabp7 | - | - | - | 2.22 |
| AK014174 | chk | - | - | - | 2.22 |
| BC025508 | unknown (protein for mgc:38064) | - | - | - | 2.22 |
| NM\_021356 | growth factor receptor bound protein 2-associated protein 1; gab1 | - | - | - | 2.22 |
| AY057384 | neuromedin u receptor type 2 | - | - | - | 2.21 |
| NM\_009602 | acetylcholine receptor beta 2 neural; chrnb2 | - | - | - | 2.21 |
| AY073606 | olfactory receptor mor208-2 | - | - | - | 2.21 |
| NM\_009964 | crystallin; alpha 2; cryab | - | - | - | 2.21 |
| BC025429 | similar to riken cdna 2810049g06 gene | - | - | - | 2.20 |
| NM\_021897 | thymus expressed acidic protein; teap-pending | - | - | - | 2.20 |
| ENSMUSG00000026314 | ENSMUST00000027546 cadherin [source:ensembl\_protein\_families;acc:ensmusf00000000213]; cadherin | - | - | 2.01 | 2.20 |
| NM\_134030 | expressed sequence ai849362; ai849362 | - | - | - | 2.20 |
| ENSMUSG00000034163 | ENSMUST00000036044 protein [source:ensembl\_protein\_families;acc:ensmusf00000011113]; protein | - | - | - | 2.20 |
| ENSMUSG00000030375 | ENSMUST00000006179 homeobox protein meis3 (meis1-related protein 2). [source:swissprot;acc:p97368]; homeobox protein | - | - | - | 2.20 |
| NM\_010197 | fibroblast growth factor 1; fgf1 | - | - | - | 2.20 |
| M11301 | polymerase | - | - | - | 2.20 |
| NM\_008874 | phospholipase c; beta 3; plcb3 | - | - | - | 2.20 |
| NM\_016765 | dimethylarginine dimethylaminohydrolase 2; ddah2 | - | - | - | 2.20 |
| AK009634 | homolog to human dj1009e24.7 | - | - | 2.20 | - |
| AK007393 | homolog to hypothetical 77.3 kda protein (kiaa1536 protein) | - | - | - | 2.19 |
| AK016525 | unclassifiable | - | - | - | 2.18 |
| NM\_021878 | jumonji protein; jmj | - | - | - | 2.18 |
| ENSMUSG00000033618 | ENSMUST00000042065 mitogen activated protein kinase kinase kinase 12 ec 2.7.1.37 leucine zipper protein kinase [source:ensembl\_protein\_families;acc:ensmusf00000001928]; mitogen activated protein kinase kinase kinase 12 ec 2.7.1.37 leucine zipper protein k | - | - | - | 2.17 |
| ENSMUSG00000040851 | ENSMUST00000038378 bm282d4 3 novel protein isoform [source:ensembl\_protein\_families;acc:ensmusf00000005694]; bm282d4 3 novel protein isoform | - | - | - | 2.17 |
| NM\_134147 | expressed sequence ai604841; ai604841 | - | - | - | 2.17 |
| NM\_009367 | transforming growth factor; beta 2; tgfb2 | - | - | - | 2.17 |
| NM\_010753 | max dimerization protein 4; mad4 | - | - | 2.17 | 1.96 |
| BC006874 | similar to cg7083 gene product | - | - | 2.17 | - |
| NM\_008492 | lactate dehydrogenase 2; b chain; ldh2 | - | - | - | 2.17 |
| BC024581 | similar to hras-like suppressor 3 | - | - | 1.87 | 2.16 |
| NM\_023395 | riken cdna 2310058a03; 2310058a03rik | - | - | - | 2.16 |
| ENSMUSG00000029552 | ENSMUST00000031528 testin (tes1/tes2). [source:swissprot;acc:p47226]; testin | - | - | - | 2.16 |
| AK010756 | homolog to dj930l11.1 (similar to kiaa0397) (fragment) | - | - | - | 2.15 |
| NM\_009459 | ubiquitin-conjugating enzyme e2h; ube2h | - | - | - | 2.15 |
| NM\_054077 | proline arginine-rich end leucine-rich repeat; prelp | - | - | - | 2.15 |
| L47240 | erbb3 | - | - | - | 2.15 |
| NM\_008453 | kruppel-like factor 3 (basic); klf3 | - | - | - | 2.15 |
| U11548 | tfiii-associated transactivator factor p17 | - | - | - | 2.14 |
| AC002397\_17 | c3f | - | - | 2.14 | 2.06 |
| BC004802 | aldolase 3; c isoform | - | - | 2.14 | 1.74 |
| NM\_009373 | transglutaminase 2; c polypeptide; tgm2 | - | - | - | 2.14 |
| NM\_010952 | ornithine decarboxylase antizyme 2; oaz2 | - | - | - | 2.14 |
| ENSMUSG00000022393 | ENSMUST00000023028 unknown | - | - | - | 2.13 |
| NM\_012032 | tumor differentially expressed 1; tde1 | - | - | - | 2.13 |
| NM\_134097 | expressed sequence aw105885; aw105885 | - | - | - | 2.13 |
| NM\_011994 | atp-binding cassette; sub-family d; member 2; abcd2 | - | - | - | 2.13 |
| BC016574 | similar to hypothetical protein flj20156 | - | - | - | 2.13 |
| BC002238 | similar to hypothetical protein | - | - | - | 2.13 |
| U34361 | laf-4 | - | - | - | 2.12 |
| ENSMUSG00000039471 | ENSMUST00000047810 son [source:ensembl\_protein\_families;acc:ensmusf00000001802]; son | - | - | 2.12 | - |
| AK007339 | homolog to transcription initiation factor tfiid 18 kda subunit (tafii-18) (tafii18) | - | - | - | 2.11 |
| BC021354 | similar to double cortin and calcium/calmodulin-dependent protein kinase-like 1 | - | - | 2.11 | 1.75 |
| NM\_009238 | sry-box containing gene 4; sox4 | - | - | - | 2.11 |
| NM\_133769 | riken cdna 6430511d02; 6430511d02rik | - | - | - | 2.11 |
| NM\_016811 | diacylglycerol kinase; alpha (80 kda); dagk1 | - | - | - | 2.11 |
| AJ245720 | suppressor of actin mutations; sac1 | - | - | - | 2.10 |
| ENSMUSG00000024187 | ENSMUST00000025021 unknown | - | - | - | 2.10 |
| AF107832 | aminopeptidase | - | - | - | 2.10 |
| AF017055 | pigment epithelium-derived factor | - | - | 2.10 | 1.91 |
| NM\_030706 | tripartite motif protein trim2; trim2 | - | - | - | 2.10 |
| AK010939 | homolog to heat shock factor binding protein 1 | - | - | 2.10 | - |
| ENSMUSG00000039642 | ENSMUST00000036466 unknown | - | - | - | 2.10 |
| ENSMUSG00000040470 | ENSMUST00000046232 unknown | - | - | - | 2.10 |
| ENSMUSG00000042418 | ENSMUST00000045588 olfactory receptor [source:ensembl\_protein\_families;acc:ensmusf00000000099]; olfactory receptor | - | - | - | 2.09 |
| NM\_019709 | membrane-bound transcription factor protease; site 1; mbtps1 | - | - | 2.09 | - |
| NM\_018882 | serpentine receptor (secretin receptor superfamily member with s; gpr56 | - | - | - | 2.09 |
| ENSMUSG00000024415 | ENSMUST00000025280 transcription initiation factor tfiid kda subunit tafii [source:ensembl\_protein\_families;acc:ensmusf00000003248]; transcription initiation factor tfiid kda subunit tafii | - | - | 2.09 | 1.75 |
| AJ245936 | glucose transporter; glutx1 | - | - | 2.09 | - |
| AJ006993 | laminin alpha 5 chain; lama5 | - | - | - | 2.08 |
| NM\_021292 | ellis van creveld gene homolog (human); evc | - | - | - | 2.08 |
| NM\_007801 | cathepsin h; ctsh | - | - | - | 2.08 |
| BC026838 | similar to hematopoietic pbx-interacting protein | - | - | - | 2.08 |
| NM\_134112 | expressed sequence aw553000; aw553000 | - | - | - | 2.08 |
| ENSMUSG00000041889 | ENSMUST00000041240 unknown | - | - | - | 2.07 |
| NM\_018752 | melastatin 1; mlsn1 | - | - | - | 2.07 |
| U90029 | bicaudal-d | - | - | - | 2.07 |
| AF041857 | synaptojanin 2 isoform beta | - | - | - | 2.07 |
| ENSMUSG00000001786 | ENSMUST00000001837 f box only protein [source:ensembl\_protein\_families;acc:ensmusf00000008869]; f box only protein | - | - | - | 2.07 |
| ENSMUSG00000011486 | ENSMUST00000040183 grave's disease carrier protein gdc mitochondrial solute carrier protein [source:ensembl\_protein\_families;acc:ensmusf00000000752]; grave's disease carrier protein gdc mitochondrial solute carrier protein | - | - | - | 2.07 |
| NM\_007993 | fibrillin 1; fbn1 | - | - | - | 2.07 |
| NM\_008154 | g-protein coupled receptor 3; gpcr3 | - | - | 2.07 | 1.88 |
| NM\_008769 | ornithine transcarbamylase; otc | - | - | - | 2.07 |
| NM\_030889 | vps10 domain receptor protein sorcs 2; sorcs2-pending | - | - | - | 2.06 |
| S37484 | angiotensin ii receptor isoform 1a; angiotensin ii receptor isoform 1a; at-1a receptor | - | - | - | 2.06 |
| AJ288055 | bicaudal d protein; bicd1 | - | - | 2.06 | - |
| AF332055 | cyclic nucleotide phophodiesterase 1; cnp1 | - | - | - | 2.06 |
| NM\_023719 | thioredoxin interacting protein; txnip | - | - | - | 2.06 |
| NM\_008238 | hnf-3/forkhead homolog 11; foxn1 | - | - | - | 2.06 |
| NM\_020520 | carnitine/acylcarnitine translocase; slc25a20 | - | - | - | 2.06 |
| ENSMUSG00000027652 | ENSMUST00000046274 unknown | - | - | - | 2.05 |
| AK014635 | similar to ultra-high sulphur keratin | - | - | 1.92 | 2.05 |
| NM\_011153 | g substrate; gsbs-pending | - | - | - | 2.05 |
| AK003665 | homolog to erg2 protein | - | - | - | 2.05 |
| ENSMUSG00000038024 | ENSMUST00000045512 unknown | - | - | - | 2.05 |
| NM\_033474 | armadillo repeat gene deleted in velo-cardio-facial syndrome; arvcf | - | - | - | 2.05 |
| AF305427 | camp-dependent protein kinase regulatory subunit | - | - | - | 2.04 |
| ENSMUSG00000036575 | ENSMUST00000021006 unknown | - | - | - | 2.04 |
| NM\_008627 | myeloid ecotropic viral integration site-related gene 2; mrg2 | - | - | - | 2.04 |
| NM\_010077 | dopamine receptor 2; drd2 | - | - | - | 2.04 |
| AF059175 | egf-like growth factor receptor erbb3 extracellular domain | - | - | - | 2.04 |
| AK019914 | cdna clone homolog to breast cancer suppressor candidate 1 | - | - | - | 2.04 |
| NM\_021384 | viral hemorrhagic septicemia virus(vhsv) induced gene 1; vig1-pending | - | - | - | 2.04 |
| NM\_025760 | riken cdna 4933428i03; 4933428i03rik | - | - | - | 2.04 |
| ENSMUSG00000020507 | ENSMUST00000020814 olfactory receptor [source:ensembl\_protein\_families;acc:ensmusf00000000032]; olfactory receptor | - | - | - | 2.04 |
| NM\_053078 | neuronal protein 3.1; d0h4s114 | - | - | - | 2.03 |
| NM\_133704 | riken cdna 1810005c06; 1810005c06rik | - | - | - | 2.03 |
| AF449483 | aggrecanase-1 | - | - | - | 2.03 |
| NM\_009306 | synaptotagmin 1; syt1 | - | - | - | 2.03 |
| AF032460 | biml | - | - | - | 2.03 |
| NM\_010501 | interferon-induced protein with tetratricopeptide repeats 3; ifit3 | - | - | - | 2.02 |
| NM\_016757 | ww domain binding protein 1; wbp1 | - | - | - | 2.02 |
| AK016813 | cdna clone hypothetical protein | - | - | - | 2.02 |
| BC026372 | hypothetical protein mgc10924 similar to nedd4 ww-binding protein 5 | - | - | - | 2.02 |
| ENSMUSG00000042331 | ENSMUST00000038350 unknown | - | - | - | 2.02 |
| NM\_008613 | meiosis-specific nuclear structural protein 1; mns1 | - | - | - | 2.02 |
| NM\_130895 | adenosine deaminase; rna-specific; b1; adarb1 | - | - | - | 2.02 |
| BC005470 | similar to ubiquitin specific protease 11 | - | - | - | 2.02 |
| ENSMUSG00000022469 | ENSMUST00000023109 guanine nucleotide exchange factor [source:ensembl\_protein\_families;acc:ensmusf00000001194]; guanine nucleotide exchange factor | - | - | - | 2.02 |
| NM\_011150 | peptidylprolyl isomerase c-associated protein; ppicap | - | - | - | 2.01 |
| ENSMUSG00000032592 | ENSMUST00000035212 unknown | - | - | 1.89 | 2.01 |
| AJ278123 | mus musculus partial putative synaptopodin orf1; orf1 | - | - | - | 2.01 |
| ENSMUSG00000036067 | ENSMUST00000045734 solute carrier family 2; facilitated glucose transporter; member 8 glucose transporter type 8 glucose transporter type [source:ensembl\_protein\_families;acc:ensmusf00000019421]; solute carrier family 2; facilitated glucose transporter; m | - | - | - | 2.01 |
| BC018551 | similar to sry-box containing gene 10 | - | - | - | 2.01 |
| M35603\_2 | hox-3.1 protein | - | - | - | 2.01 |
| AK008822 | homolog to cgi-60 protein | - | - | - | 2.01 |
| NM\_013653 | small inducible cytokine a5; scya5 | - | - | - | 2.00 |
| NM\_008964 | prostaglandin e receptor 2 (subtype ep2); ptger2 | - | - | - | 2.00 |
| NM\_008052 | deltex 1 homolog (drosophila); dtx1 | - | - | 2.00 | - |
| AK020784 | unclassifiable | - | - | 2.00 | - |
| NM\_030261 | hypothetical protein; mgc:7182; bc003348 | - | - | 2.00 | - |
| AF483506 | neuropilin-2(a17) | - | - | - | 1.99 |
| AB053955 | kinesin superfamily protein 26b; kif26b | - | - | - | 1.99 |
| AL583887 | bm121m7.1 (novel protein (ortholog of human tubulin tyrosine ligase-like 1 (ttll1)); isoform 1); bm121m7.1 | - | - | - | 1.99 |
| AF133279 | b-cell leukemia/lymphoma x-gamma; bclx | - | - | - | 1.99 |
| D83144 | six3a | - | - | - | 1.99 |
| S60315 | myotonic dystrophy kinase; dmr-b15 | - | - | 1.99 | 1.84 |
| BC019991 | similar to oculospanin | - | - | 1.99 | - |
| S67218 | transferrin | - | - | - | 1.98 |
| ENSMUSG00000026632 | ENSMUST00000027945 unknown | - | - | - | 1.98 |
| NM\_010437 | human immunodeficiency virus type i enhancer binding protein 2; hivep2 | - | - | - | 1.98 |
| BC023182 | unknown (protein for mgc:37030) | - | - | 1.97 | 1.73 |
| AF368462 | histamine h2 receptor | - | - | - | 1.97 |
| AK015324 | hypothetical protein | - | - | - | 1.97 |
| BC010596 | map1 light chain 3-like protein 1 | - | - | - | 1.97 |
| NM\_011043 | protocadherin 10; pcdh10 | - | - | - | 1.97 |
| X95345 | phospholipase c beta 3 | - | - | - | 1.97 |
| ENSMUSG00000037445 | ENSMUST00000038542 ambiguous [source:ensembl\_protein\_families;acc:ensmusf00000006584]; ambiguous | - | - | - | 1.97 |
| X67282 | biliary glycoprotein; bgpg | - | - | - | 1.97 |
| NM\_011976 | sema domain; immunoglobulin domain (ig); transmembrane domain (tm) and short cytoplasmic domain; (semaphorin) 4g; sema4g | - | - | - | 1.97 |
| ENSMUSG00000036686 | ENSMUST00000040122 unknown | - | - | - | 1.97 |
| AK009137 | homolog to kiaa1434 protein (fragment) | - | - | - | 1.97 |
| NM\_027722 | riken cdna 4933436c10; 4933436c10rik | - | - | - | 1.97 |
| AK014839 | cdna clone homolog to hypothetical 88.2 kda protein | - | - | - | 1.96 |
| NM\_009089 | rna polymerase ii 1; rpo2-1 | - | - | - | 1.96 |
| NM\_023517 | tumor necrosis factor (ligand) superfamily; member 13; tnfsf13 | - | - | - | 1.96 |
| ENSMUSG00000026593 | ENSMUST00000027885 precursor [source:ensembl\_protein\_families;acc:ensmusf00000000228]; precursor | - | - | - | 1.96 |
| NM\_008872 | plasminogen activator; tissue; plat | - | - | - | 1.96 |
| NM\_020573 | oxysterol binding protein-like 1a; osbpl1a | - | - | - | 1.96 |
| NM\_033041 | hairy and enhancer of split 7 (drosophila); hes7 | - | - | - | 1.96 |
| ENSMUSG00000007237 | ENSMUST00000007381 unknown | - | - | - | 1.95 |
| AK010201 | homolog to yippee protein; a drosophila gene encoding a putative zinc binding protein | - | - | - | 1.95 |
| BC004064 | unknown (protein for image:3590584) | - | - | - | 1.95 |
| AK014390 | cdna clone beta-site app cleaving enzyme | - | - | 1.95 | - |
| ENSMUSG00000036880 | ENSMUST00000041053 3 ketoacyl coa thiolase; mitochondrial ec 2.3.1.16 beta ketothiolase acetyl coa acyltransferase mitochondrial 3 oxoacyl coa [source:ensembl\_protein\_families;acc:ensmusf00000003952]; 3 ketoacyl coa thiolase; mitochondrial ec 2.3.1.16 bet | - | - | - | 1.95 |
| M12374 | mouse ig germline kappa-chain 'recombining sequence' rs; rs orf2 bp 588-293 first start codon is located at base 427.; putative | - | - | - | 1.95 |
| NM\_010382 | histocompatibility 2; class ii antigen e beta; h2-eb1 | - | 1.62 | 1.95 | 1.94 |
| NM\_010354 | gelsolin; gsn | - | - | - | 1.94 |
| NM\_023239 | mage-g1 protein; 5730494g16rik | - | - | - | 1.94 |
| NM\_021715 | carbohydrate (n-acetylglucosamino) sulfotransferase 7; chst7 | - | - | - | 1.94 |
| BC023461 | unknown (protein for mgc:32434) | - | - | - | 1.94 |
| AK005842 | hypothetical protein | - | - | - | 1.94 |
| AK014127 | homolog to hypothetical 31.5 kda protein (dj1119d9.3) | - | - | - | 1.94 |
| ENSMUSG00000034574 | ENSMUST00000045448 unknown | - | - | - | 1.93 |
| ENSMUSG00000038175 | ENSMUST00000038275 similar to myosin regulatory light chain interacting protein. [source:sptrembl;acc:q91z47]; myosin regulatory light chain interacting | - | - | 1.93 | 1.90 |
| NM\_020276 | nasal embryonic lhrh factor; nelf | - | - | - | 1.93 |
| BC027204 | similar to riken cdna 4921517l17 gene | - | - | - | 1.93 |
| BC003954 | similar to riken cdna 1810054n16 gene | - | - | - | 1.93 |
| BC017540 | similar to kiaa0721 protein | - | - | - | 1.93 |
| NM\_013535 | c10; grcc10 | - | - | - | 1.93 |
| BC006945 | similar to dna polymerase alpha 2; 68 kda | - | - | - | 1.92 |
| NM\_024477 | hypothetical protein; mgc:7623; bc002262 | - | - | - | 1.92 |
| X06328 | ncam-140 (partial) (320 aa) (1 is 3rd base in codon) | - | - | 1.92 | 1.78 |
| BC003374 | similar to jumonji | - | - | - | 1.92 |
| NM\_025504 | riken cdna 2310004l02; 2310004l02rik | - | - | 1.92 | - |
| AK010429 | zinc finger; c3hc4 type (ring finger) containing protein | - | - | - | 1.91 |
| NM\_008937 | prospero-related homeobox 1; prox1 | - | - | - | 1.91 |
| AF100699 | mhc class i antigen qa1; h2-t23 | - | - | - | 1.91 |
| AK012136 | cited4 | - | - | 1.91 | - |
| NM\_025785 | f-box only protein 25; fbxo25 | - | - | - | 1.91 |
| ENSMUSG00000038538 | ENSMUST00000039127 unknown | - | - | - | 1.91 |
| NM\_007688 | cofilin 2; muscle; cfl2 | - | - | - | 1.91 |
| NM\_010807 | marcks-like protein; mlp | - | - | - | 1.91 |
| BC006583 | similar to hypothetical protein dkfzp434g156 | - | - | - | 1.91 |
| NM\_008590 | mesoderm specific transcript; mest | - | - | - | 1.91 |
| BC006045 | unknown (protein for image:3590207) | - | - | - | 1.91 |
| AK020041 | homolog to hypothetical 18.5 kda protein | - | - | - | 1.90 |
| NM\_015732 | axin2 | - | - | - | 1.90 |
| ENSMUSG00000014782 | ENSMUST00000014927 unknown | - | - | - | 1.90 |
| NM\_015749 | transcobalamin 2; tcn2 | - | - | - | 1.90 |
| NM\_009136 | scrapie responsive gene 1; scrg1 | - | - | - | 1.90 |
| ENSMUSG00000025006 | ENSMUST00000025971 sh3 domain protein 5; c-cbl-associated protein; ponsin. [source:refseq;acc:nm\_009166]; vinexin sh3 containing molecule 1 scam | - | - | - | 1.90 |
| ENSMUSG00000038855 | ENSMUST00000038674 1d myo inositol trisphosphate 3 kinase ec 2.7.1.127 inositol 1;4;5 trisphosphate 3 kinase ip3k ip3 3 [source:ensembl\_protein\_families;acc:ensmusf00000001420]; 1d myo inositol trisphosphate 3 kinase ec 2.7.1.127 inositol 1;4;5 trisphosph | - | - | - | 1.89 |
| ENSMUSG00000042744 | ENSMUST00000042614 unknown | - | - | - | 1.89 |
| BC013565 | unknown (protein for image:3591967) | - | - | - | 1.89 |
| ENSMUSG00000041842 | ENSMUST00000044301 unknown | - | - | - | 1.89 |
| AK009886 | homolog to cdna flj20014 fis; clone adse00113 | - | - | - | 1.89 |
| ENSMUSG00000035577 | ENSMUST00000035353 gag [source:ensembl\_protein\_families;acc:ensmusf00000005390]; gag | - | - | - | 1.89 |
| M26005 | gag protein | - | - | - | 1.89 |
| NM\_028375 | riken cdna 2900027g03; 2900027g03rik | - | - | - | 1.89 |
| X03796 | aldolase c (aa 1-227) | - | - | - | 1.89 |
| Y09588 | serotonin 4 receptor; 5-ht4 | - | - | - | 1.89 |
| AK016881 | hypothetical protein | - | - | - | 1.89 |
| ENSMUSG00000037327 | ENSMUST00000039563 zonadhesin [source:ensembl\_protein\_families;acc:ensmusf00000000626]; zonadhesin | - | - | - | 1.89 |
| ENSMUSG00000034680 | ENSMUST00000038155 ambiguous [source:ensembl\_protein\_families;acc:ensmusf00000006315]; ambiguous | - | - | - | 1.88 |
| NM\_008580 | mitogen activated protein kinase kinase kinase 5; map3k5 | - | - | - | 1.88 |
| NM\_008360 | interleukin 18; il18 | - | - | - | 1.88 |
| ENSMUSG00000042506 | ENSMUST00000041683 ubiquitin carboxyl terminal hydrolase 22 ec 3.1.2.15 ubiquitin thiolesterase 22 ubiquitin specific processing protease 22 deubiquitinating enzyme 22 [source:ensembl\_protein\_families;acc:ensmusf00000002537]; ubiquitin carboxyl terminal h | - | - | - | 1.88 |
| BC021457 | similar to sarcolemma associated protein | - | - | - | 1.88 |
| NM\_011302 | retinoschisis 1 homolog (human); rs1h | - | - | - | 1.88 |
| ENSMUSG00000042035 | ENSMUST00000044817 g protein coupled receptor [source:ensembl\_protein\_families;acc:ensmusf00000002011]; g protein coupled receptor | - | - | 1.88 | 1.73 |
| AK011566 | homolog to alpha-fodrin | - | - | - | 1.87 |
| NM\_007378 | atp-binding cassette; sub-family a; member 4; abca4 | - | - | - | 1.87 |
| BC021614 | unknown (protein for mgc:37914) | - | - | - | 1.87 |
| NM\_025933 | riken cdna 2010110m21; 2010110m21rik | - | - | - | 1.87 |
| BC002144 | similar to hypothetical protein | - | - | - | 1.87 |
| AF040095 | inositol polyphosphate 5-phosphatase ii splice variant; inpp5p | - | - | 1.87 | 1.86 |
| ENSMUSG00000035504 | ENSMUST00000040081 polyposis locus protein 1-like 1 (tb2 protein-like 1). [source:sptrembl;acc:q9jm62]; polyposis locus protein 1 tb2 | - | - | - | 1.87 |
| ENSMUSG00000040721 | ENSMUST00000036328 protein [source:ensembl\_protein\_families;acc:ensmusf00000013606]; protein | - | - | - | 1.87 |
| BC027053 | similar to neural f box protein nfb42 | - | - | 1.87 | 1.70 |
| ENSMUSG00000020132 | ENSMUST00000020343 ras related protein rab [source:ensembl\_protein\_families;acc:ensmusf00000000025]; ras related protein rab | - | - | 1.87 | - |
| AJ319726 | neurotrophin receptor interacting factor 2 | - | - | - | 1.86 |
| ENSMUSG00000034793 | ENSMUST00000036998 glucose 6 phosphatase ec 3.1.3.9 g6pase g 6 [source:ensembl\_protein\_families;acc:ensmusf00000001361]; glucose 6 phosphatase ec 3.1.3.9 g6pase g 6 | - | - | - | 1.86 |
| ENSMUSG00000031904 | ENSMUST00000034378 large neutral amino acids transporter small subunit l type amino acid transporter [source:ensembl\_protein\_families;acc:ensmusf00000000417]; large neutral amino acids transporter small subunit l type amino acid transporter | - | 1.86 | - | - |
| NM\_008862 | protein kinase inhibitor; alpha; pkia | - | - | - | 1.86 |
| U89408 | glucose-6-phosphate isomerase | - | - | - | 1.86 |
| NM\_010764 | mannosidase 2; alpha b1; man2b1 | - | - | 1.86 | 1.74 |
| AK012810 | homolog to b-myc transforming protein (fragment) | - | 1.86 | - | - |
| AF131205\_3 | neuronal apoptosis inhibitory protein-rs6; naip-rs6 | - | - | - | 1.85 |
| NM\_010338 | g protein-coupled receptor 37; gpr37 | - | - | - | 1.85 |
| AY036887 | mll3-like protein | - | - | - | 1.85 |
| NM\_010045 | duffy blood group; dfy | - | - | - | 1.85 |
| NM\_013689 | cytoplasmic tyrosine kinase; dscr28c related (drosophila); tec | - | - | - | 1.85 |
| AY073839 | olfactory receptor mor108-3 | - | - | - | 1.85 |
| NM\_011309 | s100 calcium binding protein a1; s100a1 | - | - | - | 1.85 |
| BC011310 | unknown (protein for mgc:12077) | - | - | - | 1.85 |
| NM\_009147 | sec23a (s. cerevisiae); sec23a | - | - | - | 1.85 |
| S71861 | cd40 ligand | - | - | - | 1.85 |
| NM\_018832 | pdz domain containing; x chromosome; pdzx | - | - | - | 1.85 |
| NM\_053110 | glycoprotein (transmembrane) nmb; gpnmb | - | - | 1.85 | 1.70 |
| ENSMUSG00000042321 | ENSMUST00000038231 unknown | - | - | - | 1.84 |
| NM\_025730 | riken cdna 4921513o20; 4921513o20rik | - | - | 1.84 | - |
| NM\_021530 | solute carrier family 4 (anion exchanger); member 8; slc4a8 | - | - | 1.84 | 1.72 |
| AY073719 | olfactory receptor mor178-1 | - | - | - | 1.84 |
| NM\_133716 | riken cdna 1810031k02; 1810031k02rik | - | - | 1.84 | - |
| AB011543 | dna-pkcs | - | - | - | 1.84 |
| S53716 | protein tyrosine kinase; tec | - | - | - | 1.84 |
| M36654\_76-828 | homeobox protein hox-2.6 | - | - | - | 1.83 |
| NM\_008905 | protein tyrosine phosphatase; receptor-type; f interacting protein; binding protein 2; ppfibp2 | - | - | - | 1.83 |
| AB010355 | mszf50 | - | - | - | 1.83 |
| AK021076 | homolog to cdna flj11728 fis; clone hemba1005382 | - | - | - | 1.83 |
| ENSMUSG00000030859 | ENSMUST00000033147 unknown | - | - | - | 1.83 |
| ENSMUSG00000024971 | ENSMUST00000025925 unknown | - | - | - | 1.83 |
| AF092734 | growth/differentiation factor 11 | - | - | - | 1.83 |
| AK020734 | hypothetical protein | - | - | - | 1.83 |
| NM\_008609 | matrix metalloproteinase 15; mmp15 | - | - | - | 1.83 |
| BC006842 | unknown (protein for image:3598550) | - | - | - | 1.83 |
| NM\_013832 | ras protein activator like 1 (gap1 like); rasal1 | - | - | - | 1.83 |
| NM\_008836 | per-hexamer repeat gene 5; phxr5 | - | - | - | 1.83 |
| NM\_018827 | cytokine receptor-like factor 1; crlf1 | - | - | - | 1.83 |
| AK004577 | cdna clone cd97 antigen | - | - | 1.83 | - |
| NM\_031251 | cystinosis; nephropathic; ctns | - | - | - | 1.82 |
| NM\_007826 | dachshund (drosophila); dach1 | - | - | - | 1.82 |
| ENSMUSG00000022789 | ENSMUST00000023477 dynamin [source:ensembl\_protein\_families;acc:ensmusf00000000335]; dynamin | - | - | - | 1.82 |
| BC026853 | similar to hmg-box containing protein 1 | - | - | 1.79 | 1.81 |
| ENSMUSG00000030380 | ENSMUST00000045409 zinc finger protein 98; myeloid zinc finger protein-2; zinc finger protein 121; myeloid-specific retinoic acid-responsive zinc finger protein; myeloid zinc finger protein 1; myeloid zinc finger protein 2. [source:refseq;acc:nm\_016793]; | - | - | 1.81 | - |
| BC019649 | unknown (protein for image:3994416) | - | - | - | 1.81 |
| BC025587 | unknown (protein for mgc:38259) | - | - | - | 1.81 |
| NM\_007561 | bone morphogenic protein receptor; type ii (serine/threonine kinase); bmpr2 | - | - | - | 1.81 |
| NM\_009720 | atx1 (antioxidant protein 1; yeast) homolog 1; atox1 | - | - | - | 1.81 |
| ENSMUSG00000035143 | ENSMUST00000045158 kelch like protein [source:ensembl\_protein\_families;acc:ensmusf00000000060]; kelch like protein | - | - | - | 1.81 |
| NM\_007599 | capping protein (actin filament); gelsolin-like; capg | - | - | 1.81 | 1.80 |
| NM\_009922 | calponin 1; cnn1 | - | - | - | 1.80 |
| ENSMUSG00000038559 | ENSMUST00000045405 ambiguous [source:ensembl\_protein\_families;acc:ensmusf00000007943]; ambiguous | - | - | - | 1.80 |
| AF353243 | putative wd-repeat protein | - | - | - | 1.80 |
| AK014406 | data source:sptr; source key:q9nu54; evidence:iss homolog to dj12g14.1 (novel cyclophilin type peptidyl-prolyl cis-trans isomerase) (fragment) putative | - | - | - | 1.80 |
| BC022154 | similar to hypothetical protein flj14225 | - | - | - | 1.80 |
| NM\_033072 | hypothetical protein d10wsu93e; d10wsu93e | - | - | - | 1.80 |
| AF426411 | zinc ring finger-containing protein grail | - | - | - | 1.80 |
| BC003986 | unknown (protein for image:3491638) | - | - | - | 1.80 |
| AF100424 | gamma-1 adducin; addl | - | 1.80 | - | - |
| NM\_009162 | secretory granule neuroendocrine protein 1; 7b2 protein; sgne1 | - | - | 1.80 | - |
| ENSMUSG00000032922 | ENSMUST00000036141 zinc finger protein [source:ensembl\_protein\_families;acc:ensmusf00000000001]; zinc finger protein | - | - | - | 1.79 |
| ENSMUSG00000042461 | ENSMUST00000047431 ambiguous [source:ensembl\_protein\_families;acc:ensmusf00000002369]; ambiguous | - | - | - | 1.79 |
| NM\_008410 | integral membrane protein 2b; itm2b | - | - | - | 1.79 |
| NM\_008998 | rab17; member ras oncogene family; rab17 | - | - | - | 1.79 |
| U85993 | unknown | - | - | - | 1.78 |
| NM\_010827 | musculin; msc | - | - | - | 1.78 |
| ENSMUSG00000024322 | ENSMUST00000039083 h-2 class i histocompatibility antigen; k-b alpha chain precursor (h-2k(b)). [source:swissprot;acc:p01901]; h 2 class i histocompatibility antigen; alpha chain | - | - | - | 1.78 |
| NM\_013640 | proteasome (prosomome; macropain) subunit; beta type 10; psmb10 | - | - | - | 1.78 |
| AK015675 | cntfr | - | - | - | 1.78 |
| NM\_026551 | riken cdna 6720485c15; 6720485c15rik | - | - | - | 1.78 |
| NM\_028513 | riken cdna 1700052k15; 1700052k15rik | - | - | - | 1.78 |
| ENSMUSG00000019874 | ENSMUST00000020024 fatty acid-binding protein; brain (b-fabp) (brain lipid-binding protein) (blbp). [source:swissprot;acc:p51880]; binding protein | - | - | - | 1.78 |
| AK003581 | hypothetical protein | - | - | - | 1.77 |
| NM\_021460 | lysosomal acid lipase 1; lip1 | - | - | - | 1.77 |
| NM\_018871 | tyrosine 3-monooxygenase/tryptophan 5-monooxygenase activation protein; gamma polypeptide; ywhag | - | - | - | 1.77 |
| NM\_009213 | sphingomyelin phosphodiesterase 2; neutral; smpd2 | - | - | - | 1.77 |
| NM\_133764 | riken cdna 0610006o14; 0610006o14rik | - | - | - | 1.77 |
| BC006717 | unknown (protein for mgc:12151) | - | - | 1.77 | - |
| ENSMUSG00000038384 | ENSMUST00000045342 unknown | - | - | - | 1.76 |
| ENSMUSG00000037433 | ENSMUST00000047092 unknown | - | - | - | 1.76 |
| NM\_008676 | next to the brca1; nbr1 | - | - | - | 1.76 |
| S78219 | tif1; nuclear protein tif1 | - | - | - | 1.76 |
| AF153350 | metalloprotease disintegrin; adam28 | - | - | - | 1.76 |
| BC020532 | similar to camp-regulated guanine nucleotide exchange factor i (camp-gefi) | - | - | - | 1.76 |
| ENSMUSG00000038587 | ENSMUST00000045739 unknown | - | - | - | 1.75 |
| ENSMUSG00000039369 | ENSMUST00000046789 zinc finger protein [source:ensembl\_protein\_families;acc:ensmusf00000019372]; zinc finger protein | - | - | - | 1.75 |
| AK020187 | cdna clone dna segment; human est 478828 | 1.51 | - | - | 1.75 |
| NM\_008010 | fibroblast growth factor receptor 3; fgfr3 | - | - | - | 1.75 |
| BC020024 | heterochromatin protein 2; binding protein 3 | - | - | - | 1.75 |
| AK010713 | cdna clone kruppel-like factor 3 (basic) | - | - | - | 1.75 |
| NM\_015762 | thioredoxin reductase 1; txnrd1 | - | 1.75 | - | - |
| ENSMUSG00000034415 | ENSMUST00000036906 ambiguous [source:ensembl\_protein\_families;acc:ensmusf00000002263]; ambiguous | - | - | - | 1.74 |
| ENSMUSG00000005501 | ENSMUST00000040783 ambiguous [source:ensembl\_protein\_families;acc:ensmusf00000008518]; ambiguous | - | - | - | 1.74 |
| AY073170 | olfactory receptor mor225-1 | - | - | - | 1.74 |
| U73200 | p116rip | - | - | - | 1.74 |
| AF020311 | proline-rich protein 7 | - | - | - | 1.74 |
| NM\_019831 | zinc finger protein 261; zfp261 | - | - | - | 1.74 |
| ENSMUSG00000025064 | ENSMUST00000048628 procollagen; type xvii; alpha 1. [source:refseq;acc:nm\_007732]; bullous pemphigoid | - | - | - | 1.74 |
| NM\_009265 | small proline-rich protein 1b; sprr1b | - | - | - | 1.74 |
| ENSMUSG00000021385 | ENSMUST00000021817 unknown | - | - | - | 1.73 |
| ENSMUSG00000037455 | ENSMUST00000041635 dj55c23 6 novel protein; isoform [source:ensembl\_protein\_families;acc:ensmusf00000009146]; dj55c23 6 novel protein; isoform | - | - | - | 1.73 |
| NM\_009792 | calcium/calmodulin-dependent protein kinase ii alpha; camk2a | - | - | - | 1.73 |
| AK015916 | hypothetical protein | - | - | - | 1.73 |
| ENSMUSG00000020894 | ENSMUST00000021273 vesicle-associated membrane protein 2 (vamp-2) (synaptobrevin 2). [source:swissprot;acc:q64357]; vesicle associated membrane protein vamp synaptobrevin | - | - | - | 1.73 |
| NM\_021894 | calpain 12; capn12 | - | - | - | 1.73 |
| BC006957 | similar to htgn29 protein | - | - | - | 1.73 |
| K02591 | homeo peptide | - | - | - | 1.73 |
| AK015854 | hypothetical protein | - | - | - | 1.73 |
| AK019030 | crlf1 | - | - | - | 1.73 |
| NM\_007502 | atpase; na+/k+ beta 3 polypeptide; atp1b3 | - | - | - | 1.73 |
| NM\_011050 | programmed cell death 4; pdcd4 | - | - | - | 1.73 |
| ENSMUSG00000032827 | ENSMUST00000035813 neurabin i neural tissue specific f actin binding protein i protein phosphatase 1 regulatory subunit 9a [source:ensembl\_protein\_families;acc:ensmusf00000002430]; neurabin i neural tissue specific f actin binding protein i protein phosph | - | - | - | 1.72 |
| NM\_009216 | somatostatin receptor 1; smstr1 | - | - | - | 1.72 |
| BC025603 | unknown (protein for mgc:38041) | - | - | - | 1.72 |
| AK018483 | homolog to splicing factor; proline-and glutamine-rich (polypyrimidine tract- binding protein-associated splicing factor) (ptb-associated splicing factor) (psf) (dna-binding p52/p100 complex; 100 kda subunit) | - | - | - | 1.72 |
| AK019591 | hypothetical protein | - | - | - | 1.72 |
| NM\_011957 | old astrocyte specifically induced substance; oasis-pending | - | - | - | 1.72 |
| AF336850 | cytochrome p450 cyp2j9 | - | - | 1.72 | - |
| AF059029 | calcium/calmodulin-dependent protein kinase ii delta | - | - | - | 1.72 |
| AK016555\_142-417 | calponin homology (ch) domain containing protein | - | - | - | 1.72 |
| D14637 | pebp2a2 protein; pebp2a | - | - | - | 1.71 |
| NM\_016785 | thiopurine methyltransferase; tpmt | - | - | - | 1.71 |
| U84012 | hippocampal amyloid precursor protein | - | - | - | 1.71 |
| AK017419 | hypothetical protein | - | - | - | 1.71 |
| ENSMUSG00000042156 | ENSMUST00000047208 unknown | - | - | - | 1.71 |
| NM\_133648 | solute carrier family 12; member 6; slc12a6 | - | - | - | 1.71 |
| L29278 | acute phase response factor; aprf | - | - | - | 1.71 |
| AK019824 | hypothetical protein | - | - | - | 1.71 |
| ENSMUSG00000033306 | ENSMUST00000038062 zyxin [source:ensembl\_protein\_families;acc:ensmusf00000000795]; zyxin | - | - | - | 1.71 |
| NM\_018810 | makorin; ring finger protein; 1; mkrn1 | - | - | - | 1.71 |
| NM\_033175 | small proline rich-like 1; sprrl1 | - | - | - | 1.71 |
| NM\_013624 | otogelin; otog | - | - | - | 1.70 |
| NM\_008349 | interleukin 10 receptor; beta; il10rb | - | - | - | 1.70 |
| NM\_008515 | leucine rich repeat (in flii) interacting protein 1; lrrfip1 | - | - | - | 1.70 |
| NM\_011074 | pftaire protein kinase 1; pftk1 | - | - | - | 1.70 |
| NM\_022012 | mitogen activated protein kinase kinase kinase 11; map3k11 | - | - | - | 1.70 |
| ENSMUSG00000040128 | ENSMUST00000049357 proline rich [source:ensembl\_protein\_families;acc:ensmusf00000008405]; proline rich | - | - | - | 1.70 |
| NM\_009465 | axl receptor tyrosine kinase; axl | - | - | - | 1.70 |
| AK016654 | evidence:nas hypothetical protein putative | - | - | - | 1.69 |
| AK008529 | cdna clone homolog to phospholipase | - | - | - | 1.69 |
| BC003984 | unknown (protein for image:3491252) | - | - | - | 1.69 |
| NM\_010365 | general transcription factor ii i; gtf2i | - | - | - | 1.69 |
| AK009178 | data source:sptr; source key:q24623; evidence:iss putative related to lethal(3)87df protein (fragment) | - | - | - | 1.69 |
| BC010229 | similar to guanylate nucleotide binding protein 3 | - | - | - | 1.69 |
| AF387674 | neurexin 1 nrxn1+7a; nrxn1 | - | - | - | 1.69 |
| AB035322 | ionized calcium binding adapter molecule 2 (iba2); iba2 | - | - | - | 1.69 |
| NM\_008012 | fibroblast growth factor regulated protein; fgfrp | - | - | - | 1.69 |
| BC027385 | unknown (protein for image:4953078) | - | - | - | 1.69 |
| ENSMUSG00000024220 | ENSMUST00000002318 zinc finger protein [source:ensembl\_protein\_families;acc:ensmusf00000019372]; zinc finger protein | - | - | - | 1.69 |
| AB049357\_11 | nadh dehydrogenase subunit 5 | - | - | - | 1.68 |
| NM\_023622 | ralgds-like protein 3; 1300003d20rik | - | - | - | 1.68 |
| BC007179 | similar to kiaa0144 gene product | - | - | - | 1.68 |
| AF108354 | structural glucose phosphate isomerase 1; gpi1s | - | - | - | 1.68 |
| U37485 | cde1-binding protein cdebp; cdebp | - | - | - | 1.68 |
| AF326308 | protocadherin-betao; pcdhb7 | - | - | - | 1.67 |
| NM\_138590 | dna segment; chr 4; wayne state university 132; expressed; d4wsu132e | - | - | - | 1.67 |
| AF345953 | map kinase phosphatase-m b1 isoform | - | - | - | 1.67 |
| NM\_032008 | sarcolemmal-associated protein; slap | - | - | - | 1.67 |
| NM\_007872 | dna methyltransferase 3a; dnmt3a | - | - | - | 1.67 |
| ENSMUSG00000001901 | ENSMUST00000001965 voltage gated potassium channel herg potassium channel; voltage gated subfamily h member 2 ether a go go related [source:ensembl\_protein\_families;acc:ensmusf00000000404]; voltage gated potassium channel herg potassium channel; voltage g | - | - | - | 1.67 |
| NM\_011161 | protein kinase; mitogen activated kinase; 11; p38beta; mapk11 | - | - | - | 1.67 |
| AK010930 | evidence:nas hypothetical protein putative | - | 1.67 | - | - |
| AK020110 | limb-bud and heart | - | - | - | 1.67 |
| AF102527 | olfactory receptor e3 | - | - | - | 1.67 |
| NM\_011893 | sh3-domain binding protein 2; sh3bp2 | - | - | - | 1.67 |
| BC023054 | unknown (protein for image:5357053) | - | - | - | 1.66 |
| AK003282 | homolog to dj697k14.9.1 (novel protein) (unknown) (protein for mgc:2479) | - | - | - | 1.66 |
| ENSMUSG00000024533 | ENSMUST00000025415 unknown | - | - | - | 1.66 |
| AK009454 | homolog to cdna flj13680 fis; clone place2000007; highly similar to homo sapiens kiaa0913 protein | - | - | 1.66 | - |
| ENSMUSG00000022457 | ENSMUST00000023091 homeobox protein [source:ensembl\_protein\_families;acc:ensmusf00000005332]; homeobox protein | - | - | - | 1.66 |
| ENSMUSG00000014164 | ENSMUST00000014308 kelch like protein [source:ensembl\_protein\_families;acc:ensmusf00000000060]; kelch like protein | - | - | - | 1.66 |
| AK003405 | homolog to ada3-like protein | - | - | - | 1.66 |
| ENSMUSG00000040651 | ENSMUST00000048232 unknown | - | - | - | 1.66 |
| NM\_013637 | protamine 1; prm1 | - | - | - | 1.66 |
| Z25469 | protein s | - | - | - | 1.66 |
| ENSMUSG00000029327 | ENSMUST00000043573 ambiguous [source:ensembl\_protein\_families;acc:ensmusf00000004528]; ambiguous | - | - | - | 1.66 |
| ENSMUSG00000033769 | ENSMUST00000046334 unknown | - | - | - | 1.66 |
| ENSMUSG00000040575 | ENSMUST00000048514 ribonuclease [source:ensembl\_protein\_families;acc:ensmusf00000001400]; ribonuclease | - | - | - | 1.66 |
| BC013754 | similar to mitogen activated protein kinase 3 | - | - | - | 1.65 |
| AK018640 | homolog to dj462o23.2 (novel protein) | - | - | - | 1.65 |
| X82288 | protein-tyrosine-phosphatase; ptp nu-3 | - | - | - | 1.65 |
| ENSMUSG00000036482 | ENSMUST00000046926 unknown | - | - | - | 1.65 |
| AK006427 | related to cg3309 protein | - | - | - | 1.65 |
| BC027081 | unknown (protein for image:5358729) | - | - | - | 1.65 |
| AF345952 | map kinase phosphatase-m a2 isoform | - | - | - | 1.65 |
| BC005585 | similar to hydroxyacyl-coenzyme a dehydrogenase/3-ketoacyl-coenzyme a thiolase/enoyl-coenzyme a hydratase (trifunctional protein); beta subunit | - | - | - | 1.65 |
| ENSMUSG00000042093 | ENSMUST00000047848 arylsulfatase b (ec 3.1.6.12) (asb) (n-acetylgalactosamine- 4-sulfatase) (g4s) (fragments). [source:swissprot;acc:p50429]; arylsulfatase b ec 3.1.6.12 asb n acetylgalactosamine 4 sulfatase | - | - | - | 1.65 |
| AK020222 | hypothetical protein | - | - | - | 1.64 |
| ENSMUSG00000035016 | ENSMUST00000038632 gag protein. [source:sptrembl;acc:q61540]; retrovirus related pol polyprotein | - | - | - | 1.64 |
| NM\_007760 | carnitine acetyltransferase; crat | - | - | - | 1.64 |
| AK010826 | vascular endothelial junction-associated molecule (junctional adhesion molecule-3) (2410030g21rik protein) | - | - | - | 1.64 |
| NM\_011494 | serine/threonine kinase 16; stk16 | - | - | - | 1.64 |
| AF205222 | tis11d deletion variant; brf2 | - | - | - | 1.64 |
| ENSMUSG00000019462 | ENSMUST00000019606 neuroligin [source:ensembl\_protein\_families;acc:ensmusf00000001205]; neuroligin | - | - | - | 1.64 |
| NM\_015754 | retinoblastoma binding protein 9; rbbp9 | - | - | - | 1.64 |
| NM\_019963 | signal transducer and activator of transcription 2; stat2 | - | - | - | 1.63 |
| ENSMUSG00000038667 | ENSMUST00000047589 unknown | - | - | - | 1.63 |
| X66177 | hox-2.7 | - | - | - | 1.63 |
| AF303106 | gtrgeo22 | - | - | - | 1.63 |
| NM\_019953 | transmembrane protein 4; tmem4 | - | - | - | 1.62 |
| M12818 | mhc h2-ie-alpha cell surface glycoprotein | - | - | - | 1.62 |
| M74773 | brain beta spectrin; spnb-2 | - | - | - | 1.62 |
| NM\_134248 | t-cell immunoglobulin and mucin domain containing 1; timd1-pending | - | - | - | 1.62 |
| AK012292 | hypothetical protein | - | - | - | 1.62 |
| AK013411 | homolog to bcl-2 related proline-rich protein | - | - | - | 1.62 |
| BC020311 | unknown (protein for image:5041241) | - | - | - | 1.62 |
| AK009297 | wd domain; g-beta repeat containing protein | - | - | - | 1.62 |
| AK007503 | hypothetical protein | - | - | - | 1.62 |
| ENSMUSG00000039572 | ENSMUST00000036034 unknown | - | - | - | 1.62 |
| NM\_007428 | angiotensinogen; agt | - | - | - | 1.62 |
| NM\_011907 | three prime repair exonuclease 2; trex2 | - | - | - | 1.62 |
| NM\_029385 | riken cdna 2310041h06; 2310041h06rik | - | - | - | 1.61 |
| NM\_008289 | hydroxysteroid 11-beta dehydrogenase 2; hsd11b2 | - | - | - | 1.61 |
| NM\_053273 | tweety homolog 2 (drosophila); ttyh2 | - | - | - | 1.61 |
| NM\_008090 | gata binding protein 2; gata2 | - | - | - | 1.61 |
| NM\_009297 | suppressor of ty 6 homolog (s. cerevisiae); supt6h | - | 1.61 | - | - |
| AK005230 | similar to ras-related protein rab-2 | - | - | - | 1.61 |
| NM\_011190 | proteasome (prosome; macropain) 28 subunit; beta; psme2 | - | - | - | 1.61 |
| NM\_025626 | riken cdna 3110001a13; 3110001a13rik | 1.61 | - | - | - |
| BC026742 | unknown (protein for mgc:25845) | - | - | - | 1.60 |
| ENSMUSG00000034496 | ENSMUST00000020842 acetyl coa carboxylase ec 6.4.1.2 acc [includes: biotin carboxylase ec 6.3.4.- 14 [source:ensembl\_protein\_families;acc:ensmusf00000000774]; acetyl coa carboxylase ec 6.4.1.2 acc [includes: biotin carboxylase ec 6.3.4.- 14 | - | - | - | 1.60 |
| AF218253 | vacuolar proton-translocating atpase 100 kda subunit isoform a3 | 1.60 | - | - | - |
| NM\_007765 | collapsin response mediator protein 1; crmp1 | - | - | 1.60 | - |
| NM\_026257 | riken cdna 4930506l07; 4930506l07rik | - | - | - | 1.60 |
| AB051562 | glutamate receptor interacting protein 1b-s; grip1 | - | - | - | 1.60 |
| NM\_009875 | cyclin-dependent kinase inhibitor 1b (p27); cdkn1b | - | - | - | 1.60 |
| NM\_024204 | riken cdna 5430429d21; 5430429d21rik | - | - | - | 1.60 |
| ENSMUSG00000000394 | ENSMUST00000000404 glucagon precursor [contains: glicentin-related polypeptide (grpp); glucagon; glucagon-like peptide 1 (glp1); glucagon-like peptide 2 (glp2)]. [source:swissprot;acc:p55095]; glucagon precursor [contains: glicentin related polypeptide gr | - | - | - | 1.60 |
| AF321301 | fibroblast growth factor receptor 5 gamma precursor; fgfr5 | - | - | - | 1.60 |
| AY034611 | stress-induced protein sip18; sip | - | - | - | 1.59 |
| BC025586 | similar to hypothetical protein flj10743 | - | - | - | 1.59 |
| ENSMUSG00000037652 | ENSMUST00000046624 polyhomeotic [source:ensembl\_protein\_families;acc:ensmusf00000002250]; polyhomeotic | - | - | - | 1.59 |
| D50000 | doc2; mdoc2 | - | - | - | 1.59 |
| NM\_007399 | a disintegrin and metalloprotease domain 10; adam10 | - | - | - | 1.59 |
| AK016573 | homolog to cdna flj13593 fis; clone place1009493 | - | - | - | 1.58 |
| NM\_009636 | ae binding protein 1; aebp1 | - | - | - | 1.58 |
| NM\_134116 | expressed sequence aa960287; aa960287 | - | - | - | 1.58 |
| AY073450 | olfactory receptor mor181-1 | - | - | - | 1.58 |
| NM\_025508 | guanosine monophosphate reductase; gmpr | - | - | - | 1.58 |
| AB030201 | contains transmembrane (tm) region | - | - | - | 1.57 |
| AK005562 | homolog to echinoderm microtubule-associated protein-like emap2 | - | - | - | 1.57 |
| BC008150 | unknown (protein for image:3584069) | - | - | - | 1.57 |
| NM\_009287 | stromal interaction molecule 1; stim1 | - | - | - | 1.57 |
| NM\_028189 | udp-glcnac:betagal beta-1;3-n-acetylglucosaminyltransferase 3; b3gnt3 | - | - | - | 1.57 |
| ENSMUSG00000035933 | ENSMUST00000036862 putative 13 s golgi transport complex 90kd subunit [source:ensembl\_protein\_families;acc:ensmusf00000007841]; putative 13 s golgi transport complex 90kd subunit | - | - | - | 1.57 |
| NM\_011163 | eukaryotic translation initiation factor 2 alpha kinase 2; eif2ak2 | - | - | - | 1.57 |
| ENSMUSG00000035375 | ENSMUST00000047948 cgi 85 [source:ensembl\_protein\_families;acc:ensmusf00000004388]; cgi 85 | - | - | - | 1.56 |
| AJ319753 | putative mc2 protein | - | - | - | 1.56 |
| NM\_010800 | muscle; intestine and stomach expression 1; mist1 | - | - | - | 1.56 |
| D44592 | zinc alpha 2 glycoprotein | - | - | - | 1.56 |
| AF442737 | hypothetical protein rda63 | - | - | - | 1.56 |
| NM\_033584 | protocadherin gamma subfamily a; 1; pcdhga1 | - | - | - | 1.56 |
| NM\_134050 | riken cdna 2310012g06; 2310012g06rik | - | - | - | 1.56 |
| AK009596 | ubap | - | - | - | 1.56 |
| NM\_019801 | open reading frame 18; orf18 | - | - | - | 1.56 |
| NM\_029926 | riken cdna 9330209d03; 9330209d03rik | - | - | - | 1.55 |
| NM\_011027 | purinergic receptor p2x; ligand-gated ion channel; 7; p2rx7 | - | - | - | 1.55 |
| NM\_026252 | riken cdna 4930447d24; 4930447d24rik | - | - | - | 1.55 |
| AK017066 | hypothetical protein | - | - | - | 1.55 |
| ENSMUSG00000020721 | ENSMUST00000021064 unknown | - | - | - | 1.55 |
| X66118\_543-2693 | glutamate receptor subunit glur5-2c | - | - | - | 1.54 |
| AK019490 | sh3 domain containing protein data source:pfam; source key:pf00018; evidence:iss putative | - | - | - | 1.54 |
| BC005738 | unknown (protein for mgc:6860) | - | - | - | 1.54 |
| AB048844 | fertilin alpha; adam1a | - | - | - | 1.54 |
| AY073238 | olfactory receptor mor258-1 | - | - | - | 1.53 |
| AK019821 | hipk2 | - | - | - | 1.52 |
| AF276951 | hmg-box containing protein; hbp2 | - | - | - | 1.52 |
| Z78150 | unknown | - | - | - | 1.51 |
| NM\_080461 | zinc finger protein 358; zfp358 | - | - | - | 1.50 |
| AF232828 | ventral neuron-specific protein 1 nova1; nova1 | - | - | - | 1.50 |
| AK009812 | data source:sptr; source key:q9vtp1; evidence:iss putative related to cg14130 protein | - | 1.50 | - | - |
| NM\_026139 | riken cdna 3230401n03; 3230401n03rik | - | - | - | 0.66 |
| BC014688 | similar to peter pan (drosophila) homolog | - | - | - | 0.66 |
| ENSMUSG00000031296 | ENSMUST00000033654 high mobility group protein hmg [source:ensembl\_protein\_families;acc:ensmusf00000000264]; high mobility group protein hmg | - | - | - | 0.66 |
| BC011285 | unknown (protein for mgc:7004) | - | - | - | 0.66 |
| NM\_134084 | expressed sequence aw457192; aw457192 | - | - | - | 0.66 |
| X61449 | dn38 | - | - | - | 0.66 |
| AK020147 | data source:sptr; source key:q9h5g6; evidence:iss homolog to cdna: flj23459 fis; clone hsi07588 putative | - | - | - | 0.66 |
| NM\_016897 | translocase of inner mitochondrial membrane 23 homolog (yeast); timm23 | - | - | - | 0.65 |
| NM\_019472 | myosin x; myo10 | - | - | - | 0.65 |
| NM\_010344 | glutathione reductase 1; gsr | - | - | - | 0.65 |
| S72304 | lmw g-protein; rah | - | - | - | 0.65 |
| AK012939 | data source:sptr; source key:q9p0p6; evidence:iss homolog to hspc232 putative | - | - | - | 0.65 |
| AK019462 | small nuclear ribonucleoprotein e | - | - | - | 0.65 |
| NM\_026616 | riken cdna 1500026d16; 1500026d16rik | - | - | - | 0.65 |
| BC008121 | similar to glutamate rich wd repeat protein grwd | - | - | - | 0.65 |
| NM\_011605 | thymopoietin; tmpo | - | - | - | 0.64 |
| BC007151 | similar to nucleolar protein 1 (120kd) | - | - | - | 0.64 |
| AK015349 | protein phosphatase 1; regulatory (inhibitor) 2 | - | - | - | 0.64 |
| NM\_053086 | riken cdna 3230402k17; nolc1 | - | - | - | 0.64 |
| NM\_134151 | expressed sequence al024047; al024047 | - | - | - | 0.64 |
| U82270 | breast cancer susceptibility; brca2 | - | - | - | 0.64 |
| BC006917 | similar to metalloprotease 1 (pitrilysin family) | - | - | - | 0.64 |
| ENSMUSG00000024281 | ENSMUST00000025131 mitotic spindle assembly checkpoint protein mad2a mad2 like [source:ensembl\_protein\_families;acc:ensmusf00000006229]; mitotic spindle assembly checkpoint protein mad2a mad2 like | - | - | - | 0.64 |
| NM\_007853 | degenerative spermatocyte homolog (drosophila); degs | - | - | - | 0.64 |
| NM\_008840 | phosphatidylinositol 3-kinase catalytic delta polypeptide; pik3cd | - | - | - | 0.63 |
| NM\_018813 | cleavage and polyadenylation specificity factor 3; cpsf3 | - | - | - | 0.63 |
| AK009199 | homolog to dj616b8.3 (novel gene) (fragment) | - | - | - | 0.63 |
| NM\_009352 | telomeric repeat binding factor 1; terf1 | - | - | - | 0.63 |
| AK013109 | cdna clone homolog to chromosome-associated protein-e | - | - | - | 0.63 |
| BC006859 | unknown (protein for image:3594992) | - | - | - | 0.63 |
| ENSMUSG00000035271 | ENSMUST00000038929 atp dependent rna helicase ddx18 dead box protein 18 myc regulated dead box protein [source:ensembl\_protein\_families;acc:ensmusf00000003967]; atp dependent rna helicase ddx18 dead box protein 18 myc regulated dead box protein | - | - | - | 0.63 |
| AK018609 | homolog to thyroid receptor interacting protein 3 (trip3) (fragment) | - | - | - | 0.63 |
| ENSMUSG00000037557 | ENSMUST00000045305 unknown | - | - | - | 0.63 |
| NM\_023595 | 5031412i06rik | - | - | - | 0.63 |
| ENSMUSG00000037743 | ENSMUST00000037973 nucleoside diphosphate kinase ec 2.7.4.6 ndk ndp kinase [source:ensembl\_protein\_families;acc:ensmusf00000000449]; nucleoside diphosphate kinase ec 2.7.4.6 ndk ndp kinase | - | - | - | 0.63 |
| NM\_023294 | hec protein; 2610020p18rik | - | - | - | 0.63 |
| NM\_009214 | spermine synthase; sms | - | - | - | 0.63 |
| NM\_018799 | eukaryotic translation initiation factor 3; subunit 2 (beta; 36kd); eif3s2 | - | - | - | 0.63 |
| U83633 | sp100 | - | - | - | 0.63 |
| ENSMUSG00000034530 | ENSMUST00000039354 zinc finger protein [source:ensembl\_protein\_families;acc:ensmusf00000000001]; zinc finger protein | - | - | - | 0.63 |
| ENSMUSG00000027823 | ENSMUST00000029405 gmp synthase [glutamine hydrolyzing] ec 6.3.5.2 glutamine amidotransferase gmp [source:ensembl\_protein\_families;acc:ensmusf00000006017]; gmp synthase [glutamine hydrolyzing] ec 6.3.5.2 glutamine amidotransferase gmp | - | - | - | 0.63 |
| BC027154 | unknown (protein for image:4953665) | - | - | - | 0.63 |
| NM\_013556 | hypoxanthine guanine phosphoribosyl transferase; hprt | - | - | - | 0.63 |
| BC025529 | similar to splicing factor; arginine/serine-rich 7 (35kd) | - | - | - | 0.63 |
| NM\_010760 | mago-nashi homolog; proliferation-associated (drosophila); magoh | - | - | - | 0.63 |
| NM\_013614 | ornithine decarboxylase; structural; odc | - | - | - | 0.63 |
| ENSMUSG00000015143 | ENSMUST00000021554 alpha actinin alpha actinin f actin cross linking [source:ensembl\_protein\_families;acc:ensmusf00000000634]; alpha actinin alpha actinin f actin cross linking | - | - | - | 0.63 |
| Y10349 | msirp-alpha1 | - | - | - | 0.63 |
| NM\_013536 | gene rich cluster; c2f gene; grcc2f | - | - | - | 0.62 |
| AK012372 | evidence:nas hypothetical protein putative | - | - | - | 0.62 |
| NM\_025695 | riken cdna 3830418c19; 3830418c19rik | - | - | - | 0.62 |
| BC003444 | unknown (protein for mgc:6873) | - | - | - | 0.62 |
| ENSMUSG00000035217 | ENSMUST00000038279 cdna fis; clone ; weakly similar to rattus norvegicus schlafen 4 slfn 4 [source:ensembl\_protein\_families;acc:ensmusf00000007647]; cdna fis; clone ; weakly similar to rattus norvegicus schlafen 4 slfn 4 | - | - | - | 0.62 |
| BC008995 | similar to riken cdna 2610024n01 gene | - | - | - | 0.62 |
| AF426314 | sh3glb2 | - | - | - | 0.62 |
| AK010433 | homolog to quinone oxidoreductase-like 1 (qoh-1) (4p11) | - | - | - | 0.62 |
| NM\_023479 | elac homolog 2 (e. coli); elac2 | - | - | - | 0.62 |
| AK003292 | data source:sptr; source key:q9ui30; evidence:iss homolog to adrenal gland protein ad-001 (hspc170 protein) (hspc152) putative | - | - | - | 0.62 |
| NM\_009722 | atpase; ca++ transporting; cardiac muscle; slow twitch 2; atp2a2 | - | - | - | 0.62 |
| AK011691 | cdna clone similar to chloride conductance regulatory protein icln i(cln) (chloride channel; nucleotide sensitive 1a) (chloride ion current inducer protein) (clci) | - | - | - | 0.62 |
| BC006867 | unknown (protein for mgc:11792) | - | - | - | 0.62 |
| ENSMUSG00000041764 | ENSMUST00000047107 sodium/potassium/calcium exchanger 3 precursor na + /k + /ca 2+ exchange protein [source:ensembl\_protein\_families;acc:ensmusf00000005674]; sodium/potassium/calcium exchanger 3 precursor na + /k + /ca 2+ exchange protein | - | - | - | 0.62 |
| NM\_025705 | riken cdna 4631413k11; 4631413k11rik | - | - | - | 0.62 |
| AK019297 | cdna clone homolog to hypothetical protein kiaa0056 (fragment) | - | - | - | 0.62 |
| NM\_023117 | cell division cycle 25 homolog b (s. cerevisiae); cdc25b | - | - | - | 0.62 |
| AK016839 | wd domain; g-beta repeat containing protein data source:pfam; source key:pf00400; evidence:iss putative | - | - | - | 0.62 |
| BC011077 | similar to riken cdna 1200003m09 gene | - | - | - | 0.62 |
| NM\_019805 | anaphase-promoting complex subunit 7; apc7-pending | - | - | - | 0.62 |
| ENSMUSG00000039394 | ENSMUST00000048730 ambiguous [source:ensembl\_protein\_families;acc:ensmusf00000003173]; ambiguous | - | - | - | 0.62 |
| NM\_012058 | signal recognition particle 9 kda; srp9 | - | - | - | 0.62 |
| AF016099 | glycine receptor beta-subunit | - | - | - | 0.61 |
| ENSMUSG00000029424 | ENSMUST00000031374 nucleoporin [source:ensembl\_protein\_families;acc:ensmusf00000004182]; nucleoporin | - | - | - | 0.61 |
| ENSMUSG00000039141 | ENSMUST00000035977 unknown | - | - | - | 0.61 |
| ENSMUSG00000040199 | ENSMUST00000035972 ambiguous [source:ensembl\_protein\_families;acc:ensmusf00000007448]; ambiguous | - | - | - | 0.61 |
| NM\_009013 | rad51 associated protein 1; rad51ap1 | - | - | - | 0.61 |
| NM\_018776 | cytokine receptor-like factor 3; crlf3 | - | - | - | 0.61 |
| NM\_053089 | riken cdna 5730450d16; narg1-pending | - | - | - | 0.61 |
| AF044672 | alpha-synuclein | - | - | - | 0.61 |
| AK012959 | cdna clone zinc finger; c2h2 type containing protein | - | - | - | 0.61 |
| BC006788 | unknown (protein for mgc:8151) | - | - | - | 0.61 |
| BC004622 | similar to growth arrest specific 5 | 0.61 | - | - | - |
| AK003131 | data source:sptr; source key:q9ui09; evidence:iss homolog to nadh:ubiquinone oxidoreductase 17.2-kda subunit putative | - | - | - | 0.61 |
| BC026842 | similar to polymerase (rna) ii (dna directed) polypeptide e (25kd) | - | - | - | 0.61 |
| BC027172 | unknown (protein for image:3711174) | - | - | - | 0.61 |
| NM\_019428 | ribonuclease p2; rnasep2-pending | - | - | - | 0.61 |
| BC027326 | polymyositis/scleroderma autoantigen 2 | - | - | - | 0.61 |
| AF002823 | mitotic checkpoint protein kinase; bub1 | - | - | - | 0.61 |
| AK012744 | cdna clone budding uninhibited by benzimidazoles 3 homolog (s. cerevisiae) | - | - | - | 0.61 |
| NM\_010247 | thyroid autoantigen 70 kda; g22p1 | - | - | - | 0.61 |
| X57012 | transcription factor; ap-2 | - | - | - | 0.61 |
| AF027505 | putative membrane-associated guanylate kinase 1; magi-1 | - | - | - | 0.61 |
| BC022918 | similar to phosphatidylglycerophosphate synthase | - | - | - | 0.61 |
| NM\_025317 | mitochondrial ribosomal protein l54; mrpl54 | - | - | - | 0.61 |
| ENSMUSG00000042148 | ENSMUST00000049091 protoheme ix farnesyltransferase; mitochondrial precursor ec 2.5.1.- heme o [source:ensembl\_protein\_families;acc:ensmusf00000009273]; protoheme ix farnesyltransferase; mitochondrial precursor ec 2.5.1.- heme o | - | - | - | 0.61 |
| NM\_133756 | riken cdna 2410004j02; 2410004j02rik | - | - | - | 0.61 |
| AK016622 | similar to cellular retinaldehyde-binding protein (cralbp) | - | - | - | 0.61 |
| BC010583 | similar to mannosyl (alpha-1;6-)-glycoprotein beta-1;2-n-acetylglucosaminyltransferase | - | - | - | 0.60 |
| AK006556 | hypothetical protein | - | - | - | 0.60 |
| NM\_138596 | expressed sequence c78613; d13wsu50e | - | - | - | 0.60 |
| AK005100 | related to cg3641 protein | - | - | - | 0.60 |
| NM\_007669 | cyclin-dependent kinase inhibitor 1a (p21); cdkn1a | - | - | - | 0.60 |
| AB059278 | transcription factor elys; elys | - | - | - | 0.60 |
| AK011907 | cdna clone myosin tail containing protein | - | - | - | 0.60 |
| AK017824 | mrpl15 | - | - | - | 0.60 |
| BC008132 | unknown (protein for mgc:6664) | - | - | - | 0.60 |
| ENSMUSG00000001569 | ENSMUST00000001611 unknown | - | - | - | 0.60 |
| ENSMUSG00000033970 | ENSMUST00000038131 unknown | - | - | - | 0.60 |
| NM\_025281 | ly1 antibody reactive clone; lyar | - | - | - | 0.60 |
| AK011738 | cenph | - | - | - | 0.60 |
| ENSMUSG00000026615 | ENSMUST00000046350 bifunctional aminoacyl trna synthetase [includes: glutamyl trna synthetase ec 6.1.1.17 glutamate trna ligase ; prolyl trna synthetase ec 6.1.1.- 15 proline trna ligase [source:ensembl\_protein\_families;acc:ensmusf00000006094]; bifunction | - | - | - | 0.60 |
| NM\_008567 | mini chromosome maintenance deficient 6 (s. cerevisiae); mcmd6 | - | - | - | 0.60 |
| NM\_009193 | stem-loop binding protein; slbp | - | - | - | 0.60 |
| NM\_023140 | thioredoxin-like 2; txnl2 | - | - | - | 0.60 |
| NM\_026042 | riken cdna 2810405o22; 2810405o22rik | - | - | - | 0.60 |
| NM\_133678 | riken cdna 2410004c24; 2410004c24rik | - | - | - | 0.60 |
| AK020067 | hypothetical protein | - | - | - | 0.60 |
| AY055832 | myotubularin-related protein 2; mtmr2 | - | - | - | 0.60 |
| BC026381 | similar to dead/h (asp-glu-ala-asp/his) box polypeptide 27 | - | - | - | 0.60 |
| ENSMUSG00000021282 | ENSMUST00000001308 eukaryotic translation initiation factor 5 eif [source:ensembl\_protein\_families;acc:ensmusf00000006423]; eukaryotic translation initiation factor 5 eif | - | - | - | 0.60 |
| NM\_025564 | riken cdna 2010012c16; 2010012c16rik | - | - | - | 0.60 |
| AK019135 | phd-finger containing protein | - | - | - | 0.60 |
| ENSMUSG00000024036 | ENSMUST00000047565 glycerol 3 phosphate transporter g 3 p transporter g 3 p [source:ensembl\_protein\_families;acc:ensmusf00000003749]; glycerol 3 phosphate transporter g 3 p transporter g 3 p | - | - | - | 0.60 |
| AK010018 | related to ribosome recycling factor | - | - | - | 0.60 |
| NM\_138589 | dna segment; chr 7; wayne state university 128; expressed; d7wsu128e | - | - | - | 0.60 |
| ENSMUSG00000034581 | ENSMUST00000038866 unknown | - | - | - | 0.60 |
| NM\_008564 | mini chromosome maintenance deficient 2 (s. cerevisiae); mcmd2 | - | - | - | 0.60 |
| NM\_011638 | transferrin receptor; trfr | - | - | - | 0.60 |
| NM\_025391 | riken cdna 1110017c15; 1110017c15rik | - | - | - | 0.60 |
| AF247181 | fanca | - | - | - | 0.59 |
| ENSMUSG00000032297 | ENSMUST00000034839 cug triplet repeat rna binding protein 1 cug bp1 rna binding protein brunol 2 deadenylation factor cug bp protein [source:ensembl\_protein\_families;acc:ensmusf00000000443]; cug triplet repeat rna binding protein 1 cug bp1 rna binding pro | - | - | - | 0.59 |
| ENSMUSG00000032662 | ENSMUST00000040216 g2 protein [source:ensembl\_protein\_families;acc:ensmusf00000006745]; g2 protein | - | - | - | 0.59 |
| BC013717 | eukaryotic translation termination factor 1 | - | - | - | 0.59 |
| ENSMUSG00000037531 | ENSMUST00000043966 mitochondrial 39s ribosomal protein l47 mrp [source:ensembl\_protein\_families;acc:ensmusf00000011060]; mitochondrial 39s ribosomal protein l47 mrp | - | - | - | 0.59 |
| ENSMUSG00000018548 | ENSMUST00000041282 ambiguous [source:ensembl\_protein\_families;acc:ensmusf00000008846]; ambiguous | - | - | - | 0.59 |
| ENSMUSG00000039108 | ENSMUST00000041419 ba11m20 3 novel protein similar to pleurodeles waltlii rap55 protein; isoform [source:ensembl\_protein\_families;acc:ensmusf00000006617]; ba11m20 3 novel protein similar to pleurodeles waltlii rap55 protein; isoform | - | - | - | 0.59 |
| X92590 | hira protein; hira | - | - | - | 0.59 |
| ENSMUSG00000020185 | ENSMUST00000020402 similar to growth accentuating protein 43 [source:ensembl\_protein\_families;acc:ensmusf00000012495]; similar to growth accentuating protein 43 | - | - | - | 0.59 |
| ENSMUSG00000041611 | ENSMUST00000038123 meningioma expressed antigen 6/11 mea6 [source:ensembl\_protein\_families;acc:ensmusf00000001684]; meningioma expressed antigen 6/11 mea6 | - | - | - | 0.59 |
| NM\_009462 | ubiquintin c-terminal hydrolase related polypeptide; uchrp | - | - | - | 0.59 |
| NM\_013929 | cd27 binding protein (hindu god of destruction); siva-pending | - | - | - | 0.59 |
| NM\_013752 | nibrin; nbn | - | - | - | 0.59 |
| AK009245 | homolog to rna 3'-terminal phosphate cyclase (ec 6.5.1.4) (rna-3'-phosphate cyclase) (rna cyclase) | - | - | - | 0.59 |
| ENSMUSG00000028889 | ENSMUST00000030720 unknown | - | - | - | 0.59 |
| AK005212 | cdna clone homolog to hypothetical protein (fragment) | - | - | - | 0.59 |
| BC021747 | riken cdna 1200014i03 gene | - | - | - | 0.59 |
| NM\_080848 | bmp2-induced gene; big-pending | - | - | - | 0.59 |
| AF013117 | kinesin motor protein kifc4 | - | - | - | 0.59 |
| AK005498 | npm1 | - | - | - | 0.59 |
| BC005711 | similar to hypothetical protein mgc2744 | - | - | - | 0.59 |
| NM\_009982 | cathepsin c; ctsc | - | - | - | 0.59 |
| NM\_016900 | caveolin 2; cav2 | - | - | - | 0.59 |
| NM\_020619 | glucosidase 1; gcs1 | - | - | - | 0.59 |
| AK002517 | homolog to nadh-ubiquinone oxidoreductase pdsw subunit (ec 1.6.5.3) (ec 1.6.99.3) (complex i-pdsw) (ci-pdsw) | - | - | - | 0.59 |
| BC003294 | unknown (protein for mgc:7934) | - | - | - | 0.59 |
| ENSMUSG00000008754 | ENSMUST00000008898 eukaryotic translation initiation factor eif 1a eif [source:ensembl\_protein\_families;acc:ensmusf00000001982]; eukaryotic translation initiation factor eif 1a eif | - | - | - | 0.59 |
| ENSMUSG00000037621 | ENSMUST00000042646 hypothetical wd repeat protein cgi [source:ensembl\_protein\_families;acc:ensmusf00000011428]; hypothetical wd repeat protein cgi | - | - | - | 0.59 |
| NM\_019482 | pannexin 1; panx1 | - | - | - | 0.59 |
| AK011456 | prim1 | - | - | - | 0.59 |
| BC021784 | unknown (protein for mgc:28469) | - | - | - | 0.59 |
| NM\_026633 | riken cdna 9530058b02; 9530058b02rik | - | - | - | 0.59 |
| Y07685 | nuclear factor i; nfib | - | - | - | 0.59 |
| AF204156 | snrnp core protein smx5c; smx5c | - | - | - | 0.58 |
| NM\_011701 | vimentin; vim | - | - | - | 0.58 |
| AK019878 | hypothetical protein | - | - | - | 0.58 |
| ENSMUSG00000022538 | ENSMUST00000023190 unknown | - | - | - | 0.58 |
| NM\_009320 | taurine/beta-alanine transporter; slc6a6 | - | - | - | 0.58 |
| AK018316\_1-252 | unclassifiable transcript | - | - | - | 0.58 |
| NM\_008798 | programmed cell death 1; pdcd1 | - | - | - | 0.58 |
| AF123263 | phenylalanyl trna synthetase beta subunit; frsb | - | - | - | 0.58 |
| BC027191 | similar to riken cdna 1810020m02 gene | - | - | - | 0.58 |
| BC018399 | hypothetical protein similar to beta-transducin family | - | - | - | 0.58 |
| BC020103 | riken cdna 5830446m03 gene | - | - | - | 0.58 |
| ENSMUSG00000028683 | ENSMUST00000030445 translation initiation factor eif 2b gamma subunit eif 2b gdp gtp exchange [source:ensembl\_protein\_families;acc:ensmusf00000007122]; translation initiation factor eif 2b gamma subunit eif 2b gdp gtp exchange | - | - | - | 0.58 |
| NM\_026119 | riken cdna 2410046h15; 2410046h15rik | - | - | - | 0.58 |
| NM\_023663 | riken cdna 2310069j12; ankrd3 | - | - | - | 0.58 |
| NM\_010178 | neural-salient serine/arginine-rich; nssr | - | - | - | 0.58 |
| BC003950 | heat shock protein; 110 kda | - | - | - | 0.58 |
| NM\_020567 | geminin; geminin-pending | - | - | - | 0.58 |
| NM\_019517 | beta-site app-cleaving enzyme 2; bace2 | - | - | - | 0.58 |
| BC026953 | similar to protein phosphatase 2c | - | - | - | 0.58 |
| ENSMUSG00000035569 | ENSMUST00000039345 unknown | - | - | - | 0.58 |
| L38821 | mxi-wr protein | - | - | - | 0.58 |
| M33421 | protein-tyrosine kinase (ec 2.7.1.112) | - | - | - | 0.58 |
| NM\_026352 | peptidylprolyl isomerase d (cyclophilin d); ppid | - | - | - | 0.58 |
| ENSMUSG00000026798 | ENSMUST00000028137 ambiguous [source:ensembl\_protein\_families;acc:ensmusf00000008493]; ambiguous | - | - | - | 0.58 |
| AJ223293 | kinesin-related mitotic motor protein; eg5 | - | - | - | 0.58 |
| AK010489 | gnb1l | - | - | - | 0.58 |
| AK012890 | wd domain; g-beta repeat containing protein (4 wd40 repeats) | - | - | - | 0.58 |
| NM\_009466 | udp-glucose dehydrogenase; ugdh | - | - | - | 0.58 |
| NM\_011234 | rad51 homolog (s. cerevisiae); rad51 | - | - | - | 0.58 |
| NM\_026631 | riken cdna 2410130m07; 2410130m07rik | - | - | - | 0.58 |
| AK004600 | cdna clone homolog to rhogef (hypothetical 59.8 kda protein) | - | - | - | 0.58 |
| AY073520 | olfactory receptor mor185-7 | - | - | - | 0.58 |
| ENSMUSG00000033914 | ENSMUST00000035460 splicing [source:ensembl\_protein\_families;acc:ensmusf00000007023]; splicing | - | - | - | 0.58 |
| AK017747\_149-1558 | cdna clone homolog to chromosome-associated protein-e | - | - | - | 0.58 |
| NM\_009862 | cell division cycle 45 homolog (s. cerevisiae)-like; cdc45l | - | - | - | 0.58 |
| BC006019 | similar to sequestosome 1 | - | - | - | 0.58 |
| NM\_009760 | bcl2/adenovirus e1b 19 kda-interacting protein 1; nip3; bnip3 | - | - | - | 0.58 |
| BC018327 | unknown (protein for mgc:18664) | - | - | - | 0.57 |
| ENSMUSG00000040354 | ENSMUST00000037304 methionyl trna synthetase ec 6.1.1.10 methionine trna ligase [source:ensembl\_protein\_families;acc:ensmusf00000003974]; methionyl trna synthetase ec 6.1.1.10 methionine trna ligase | - | - | - | 0.57 |
| ENSMUSG00000041819 | ENSMUST00000049187 10 kda heat shock protein; mitochondrial hsp10.10 kda chaperonin [source:ensembl\_protein\_families;acc:ensmusf00000001452]; 10 kda heat shock protein; mitochondrial hsp10.10 kda chaperonin | - | - | - | 0.57 |
| NM\_025297 | nuclear receptor binding factor 1; nrbf1 | - | - | - | 0.57 |
| AK007708 | homolog to gtp-binding protein rheb | - | - | - | 0.57 |
| ENSMUSG00000035310 | ENSMUST00000046154 tesmin metallothionein like 5; testis specific testis specific metallothionein like [source:ensembl\_protein\_families;acc:ensmusf00000007366]; tesmin metallothionein like 5; testis specific testis specific metallothionein like | - | - | - | 0.57 |
| NM\_015787 | h1 histone family; member 4; h1f4 | - | - | - | 0.57 |
| AK003996 | homolog to kallikrein 5 precursor (ec 3.4.21.-) (stratum corneum tryptic enzyme) (kallikrein-like protein 2) (klk-l2) | - | - | - | 0.57 |
| NM\_007634 | cyclin f; ccnf | - | - | - | 0.57 |
| NM\_009004 | rab6; kinesin-like; rab6kifl | - | - | - | 0.57 |
| AY073035 | olfactory receptor mor30-3 | - | - | - | 0.57 |
| NM\_017373 | nuclear factor; interleukin 3; regulated; nfil3 | - | - | - | 0.57 |
| NM\_008765 | origin recognition complex; subunit 2 homolog (s. cerevisiae); orc2 | - | - | - | 0.57 |
| AF100956\_13 | ke4 | - | - | - | 0.57 |
| AK011491 | ncl | - | - | - | 0.57 |
| AY073479 | olfactory receptor mor220-3 | - | - | - | 0.57 |
| NM\_028770 | riken cdna 1200016g03; 1200016g03rik | - | - | - | 0.57 |
| X06453 | mouse cellular thyroid hormone binding protein p55; | - | - | - | 0.57 |
| ENSMUSG00000030430 | ENSMUST00000005090 troponin t1; skeletal; slow; skeletal muscle slow-twitch tnt. [source:refseq;acc:nm\_011618]; troponin t; muscle | - | - | - | 0.57 |
| ENSMUSG00000038744 | ENSMUST00000043620 ambiguous [source:ensembl\_protein\_families;acc:ensmusf00000008309]; ambiguous | - | - | - | 0.57 |
| NM\_008834 | per-hexamer repeat gene 3; phxr3 | - | - | - | 0.57 |
| NM\_011972 | polymerase (dna directed) iota; poli | - | - | - | 0.57 |
| AK011387 | cdna clone homolog to 60s acidic ribosomal protein po | - | - | - | 0.57 |
| AK003872 | data source:sptr; source key:q9h0x3; evidence:iss homolog to hypothetical 126.3 kda protein putative | - | - | - | 0.57 |
| NM\_016710 | nucleosome binding protein 1; nsbp1 | - | - | - | 0.57 |
| NM\_019836 | hypothetical brain protein similar to x96994 br-1 protein (helix pomatia); 2610024g14rik | - | - | - | 0.57 |
| AK013184 | data source:sptr; source key:q9y448; evidence:iss homolog to traf4 associated factor 1 (fragment) putative | - | - | - | 0.57 |
| ENSMUSG00000039655 | ENSMUST00000045750 similar to chromosome condensation protein g [source:ensembl\_protein\_families;acc:ensmusf00000002484]; similar to chromosome condensation protein g | - | - | - | 0.57 |
| NM\_025581 | riken cdna 2810433k01; 2810433k01rik | - | - | - | 0.57 |
| ENSMUSG00000038249 | ENSMUST00000040114 centrosomal nek2 associated protein [source:ensembl\_protein\_families;acc:ensmusf00000003999]; centrosomal nek2 associated protein | - | - | - | 0.57 |
| BC025144 | similar to gtp binding protein 5 (putative) | - | - | - | 0.57 |
| AK014996 | data source:sptr; source key:q9vyt5; evidence:iss putative related to cg10347 protein | - | - | - | 0.57 |
| AY073184 | olfactory receptor mor141-1 | - | - | - | 0.57 |
| AY073785 | olfactory receptor mor142-1 | - | - | - | 0.57 |
| ENSMUSG00000026844 | ENSMUST00000028193 argininosuccinate synthase ec 6.3.4.5 citrulline aspartate [source:ensembl\_protein\_families;acc:ensmusf00000002075]; argininosuccinate synthase ec 6.3.4.5 citrulline aspartate | - | - | - | 0.57 |
| ENSMUSG00000039267 | ENSMUST00000046514 bifunctional aminoacyl trna synthetase [includes: glutamyl trna synthetase ec 6.1.1.17 glutamate trna ligase ; prolyl trna synthetase ec 6.1.1.- 15 proline trna ligase [source:ensembl\_protein\_families;acc:ensmusf00000006094]; bifunction | - | - | - | 0.57 |
| NM\_016748 | ctp synthase; ctps | - | - | - | 0.57 |
| NM\_021512 | cdna sequence af104415; gtl1-13 | - | - | - | 0.57 |
| NM\_025437 | riken cdna 1500010b24; 1500010b24rik | - | - | - | 0.57 |
| NM\_019553 | dead/h (asp-glu-ala-asp/his) box polypeptide 21 (rna helicase ii/gu); ddx21 | - | - | - | 0.57 |
| ENSMUSG00000020093 | ENSMUST00000020290 60s ribosomal protein [source:ensembl\_protein\_families;acc:ensmusf00000001102]; 60s ribosomal protein | - | - | - | 0.57 |
| ENSMUSG00000029415 | ENSMUST00000031364 unknown | - | - | - | 0.57 |
| NM\_025415 | riken cdna 1110038l14; 1110038l14rik | - | - | - | 0.57 |
| BC013625 | unknown (protein for mgc:18985) | - | - | - | 0.56 |
| ENSMUSG00000029414 | ENSMUST00000031366 unknown | - | - | - | 0.56 |
| BC024478 | unknown (protein for mgc:37386) | - | - | - | 0.56 |
| NM\_008322 | isocitrate dehydrogenase 2 (nadp+); mitochondrial; idh2 | - | - | - | 0.56 |
| NM\_024434 | leucine aminopeptidase; lapep-pending | - | - | - | 0.56 |
| NM\_025904 | riken cdna 1600012f09; 1600012f09rik | - | - | - | 0.56 |
| M10811 | dihydrofolate reductase | - | - | - | 0.56 |
| AF303744 | oxidized ldl receptor; lox-1 | - | - | - | 0.56 |
| BC005796 | dihydrofolate reductase | - | - | - | 0.56 |
| ENSMUSG00000035612 | ENSMUST00000036140 glyceraldehyde 3-phosphate dehydrogenase (ec 1.2.1.12) (gapdh). [source:swissprot;acc:p16858]; glyceraldehyde 3 phosphate ec 1.2.1.12 | - | - | - | 0.56 |
| NM\_016750 | h2a histone family; member z; h2afz | - | - | - | 0.56 |
| NM\_009361 | transcription factor dp 1; tfdp1 | - | - | - | 0.56 |
| U01919 | dna topoisomerase iialpha/novel coding sequence fusion | - | - | - | 0.56 |
| X99638 | myosin-i beta | - | - | - | 0.56 |
| BC026468 | unknown (protein for image:4486265) | - | - | - | 0.56 |
| AF080252 | serine/threonine protein kinase 51pk(s) | - | - | - | 0.56 |
| BC023081 | similar to general transcription factor iif; polypeptide 1 (74kd subunit) | - | - | - | 0.56 |
| ENSMUSG00000034085 | ENSMUST00000044016 excision repair protein ercc 6 cockayne syndrome protein [source:ensembl\_protein\_families;acc:ensmusf00000001180]; excision repair protein ercc 6 cockayne syndrome protein | - | - | - | 0.56 |
| NM\_026190 | riken cdna 2310047g20; 2310047g20rik | - | - | - | 0.56 |
| AJ251594 | transmembrane glycoprotein; cd44 | - | - | - | 0.56 |
| BC003426 | similar to eukaryotic translation initiation factor 2b; subunit 1 (alpha; 26kd) | - | - | - | 0.56 |
| BC026464 | unknown (protein for image:4483715) | - | - | - | 0.56 |
| ENSMUSG00000033102 | ENSMUST00000039275 ambiguous [source:ensembl\_protein\_families;acc:ensmusf00000002427]; ambiguous | - | - | - | 0.56 |
| ENSMUSG00000039522 | ENSMUST00000041128 chromosome protein [source:ensembl\_protein\_families;acc:ensmusf00000002739]; chromosome protein | - | - | - | 0.56 |
| AK017472 | cdna clone homolog to hypothetical protein kiaa0136 (fragment) | - | - | - | 0.56 |
| NM\_013699 | upstream binding protein 1; ubp1 | - | - | - | 0.56 |
| BC006055 | similar to eukaryotic translation initiation factor 3; subunit 1 (alpha; 35kd) | - | - | - | 0.56 |
| BC019480 | riken cdna 1110032n12 gene | - | - | - | 0.56 |
| AF284755 | vps10 domain receptor sorcs1c splice variant | - | - | - | 0.56 |
| AF070066 | citron-k kinase | - | - | - | 0.56 |
| NM\_033574 | protocadherin gamma subfamily b; 1; pcdhgb1 | - | - | - | 0.56 |
| AK011544 | homolog to putative dimethyladenosine transferase | - | - | - | 0.55 |
| BC022136 | unknown (protein for image:5099723) | - | - | - | 0.55 |
| ENSMUSG00000040675 | ENSMUST00000043677 c 1 tetrahydrofolate synthase; cytoplasmic c1 thf synthase [includes: methylenetetrahydrofolate dehydrogenase ec 1.5.1.5 ; methenyltetrahydrofolate cyclohydrolase ec 3.5.4.- 9 ; formyltetrahydrofolate synthetase ec 6.3.-.- 4 3 [source:e | - | - | - | 0.55 |
| NM\_133227 | nucleoporin 155; nup155 | - | - | - | 0.55 |
| AF377871 | transcriptional repressor par-4-like protein pawr; pawr | - | - | - | 0.55 |
| NM\_013847 | glycine c-acetyltransferase (2-amino-3-ketobutyrate-coenzyme a ligase); gcat | - | - | - | 0.55 |
| ENSMUSG00000042481 | ENSMUST00000046491 glyceraldehyde 3 phosphate ec 1.2.1.12 [source:ensembl\_protein\_families;acc:ensmusf00000000166]; glyceraldehyde 3 phosphate ec 1.2.1.12 | - | - | - | 0.55 |
| U62923 | histone h1d | - | - | - | 0.55 |
| ENSMUSG00000041494 | ENSMUST00000044645 olfactory receptor [source:ensembl\_protein\_families;acc:ensmusf00000000083]; olfactory receptor | - | - | - | 0.55 |
| NM\_008722 | nucleophosmin 1; npm1 | - | - | - | 0.55 |
| NM\_027205 | riken cdna 1600015i10; 1600015i10rik | - | - | - | 0.55 |
| NM\_007991 | fibrillarin; fbl | - | - | - | 0.55 |
| NM\_009770 | b-cell translocation gene 3; btg3 | - | - | - | 0.55 |
| NM\_011676 | unc-119 homolog (c. elegans); unc119h | - | - | - | 0.55 |
| AK006283 | hypothetical protein | - | - | - | 0.55 |
| NM\_019638 | cold shock domain protein a; csda | - | - | - | 0.55 |
| NM\_009308 | synaptotagmin 4; syt4 | - | 0.55 | - | - |
| NM\_007532 | branched chain aminotransferase 1; cytosolic; bcat1 | - | - | - | 0.55 |
| AF194970 | lek1 | - | - | - | 0.55 |
| NM\_023058 | membrane-associated tyrosine-and threonine-specific cdc2-inhibitory kinase; pkmyt1-pending | - | - | - | 0.55 |
| NM\_026444 | citrate synthase; cs | - | - | - | 0.55 |
| AK017783 | cdna clone homolog to to kiaa1074 protein | - | - | - | 0.55 |
| NM\_007908 | eukaryotic elongation factor-2 kinase; eef2k | - | - | - | 0.55 |
| NM\_011640 | transformation related protein 53; trp53 | - | - | - | 0.55 |
| NM\_026024 | riken cdna 2700084l22; 2700084l22rik | - | - | - | 0.55 |
| AK006827 | cdna clone serine/threonine kinase 18 | - | - | - | 0.55 |
| AK020437 | data source:sptr; source key:q9qyu2; evidence:iss homolog to elongation factor putative | - | - | - | 0.55 |
| BC003427 | similar to kinesin-like 4 | - | - | - | 0.55 |
| ENSMUSG00000037940 | ENSMUST00000042529 inositol polyphosphate 4 phosphatase type i [source:ensembl\_protein\_families;acc:ensmusf00000002278]; inositol polyphosphate 4 phosphatase type i | - | - | - | 0.55 |
| NM\_013531 | guanine nucleotide binding protein; beta 4; gnb4 | - | - | - | 0.55 |
| NM\_019814 | hypoxia induced gene 1; hig1-pending | - | - | - | 0.55 |
| BC004644 | unknown (protein for mgc:7115) | - | - | - | 0.55 |
| NM\_134189 | udp-n-acetyl-alpha-d-galactosamine:polypeptide n-acetylgalactosaminyltransferase 9; galnt9 | - | - | - | 0.55 |
| NM\_009275 | signal recognition particle receptor; b subunit; srprb | - | - | - | 0.55 |
| NM\_133662 | immediate early response 3; ier3 | - | - | - | 0.55 |
| ENSMUSG00000030469 | ENSMUST00000013508 mszf6 (fragment). [source:sptrembl;acc:o88217]; zinc finger protein | - | - | - | 0.54 |
| AK005906 | data source:sptr; source key:q9ugy2; evidence:iss homolog to dj37e16.5 (novel protein similar to nitrophenylphosphatases from various organisms) (hypothetical 31.7 kda protein) putative | - | - | - | 0.54 |
| ENSMUSG00000018800 | ENSMUST00000043971 atp binding cassette [source:ensembl\_protein\_families;acc:ensmusf00000019390]; atp binding cassette | - | - | - | 0.54 |
| NM\_010175 | fas (tnfrsf6)-associated via death domain; fadd | - | - | - | 0.54 |
| NM\_009396 | tumor necrosis factor induced protein 2; tnfaip2 | - | - | - | 0.54 |
| AF246224 | rna binding protein msy4 | - | - | - | 0.54 |
| ENSMUSG00000031918 | ENSMUST00000034396 myotubularin related protein [source:ensembl\_protein\_families;acc:ensmusf00000000553]; myotubularin related protein | - | - | - | 0.54 |
| NM\_021511 | regulator for ribosome resistance homolog (s. cerevisiae); rrr-pending | - | - | - | 0.54 |
| NM\_024223 | riken cdna 0610010i23; 0610010i23rik | - | - | - | 0.54 |
| ENSMUSG00000041694 | ENSMUST00000039087 unknown | - | - | - | 0.54 |
| AK010365 | data source:sptr; source key:q9uja3; evidence:iss homolog to dj967n21.5 (novel mcm2/3/5 family member) putative | - | - | - | 0.54 |
| ENSMUSG00000026135 | ENSMUST00000027315 zinc finger 142. [source:sptrembl;acc:q925j5]; zinc finger protein | - | - | - | 0.54 |
| NM\_007684 | centrin 3; cetn3 | - | - | - | 0.54 |
| X62154 | p1.m protein | - | - | - | 0.54 |
| BC003232 | unknown (protein for image:3483627) | - | - | - | 0.54 |
| ENSMUSG00000037538 | ENSMUST00000045026 unknown | - | - | - | 0.54 |
| BC024790 | unknown (protein for image:5357662) | - | - | - | 0.54 |
| NM\_007917 | eukaryotic translation initiation factor 4e; eif4e | - | - | - | 0.54 |
| NM\_009765 | breast cancer 2; brca2 | - | - | - | 0.54 |
| ENSMUSG00000041064 | ENSMUST00000046821 dna helicase homolog [source:ensembl\_protein\_families;acc:ensmusf00000010887]; dna helicase homolog | - | - | - | 0.54 |
| NM\_021885 | tubby protein; tub | - | - | - | 0.54 |
| AK004768 | homolog to oxysterol binding protein-related protein 3 | - | - | - | 0.54 |
| AF411517 | mitotic phosphoprotein 44 | - | - | - | 0.54 |
| ENSMUSG00000034465 | ENSMUST00000043446 protein disulfide isomerase a5 precursor ec 5.3.4.1 protein disulfide isomerase related [source:ensembl\_protein\_families;acc:ensmusf00000006300]; protein disulfide isomerase a5 precursor ec 5.3.4.1 protein disulfide isomerase related | - | - | - | 0.54 |
| ENSMUSG00000006715 | ENSMUST00000006898 geminin. [source:refseq;acc:nm\_020567]; geminin | - | - | - | 0.54 |
| AK007641 | homolog to signal peptidase 21 kda subunit | - | - | - | 0.54 |
| BC002287 | similar to cell division cycle 25b | - | - | - | 0.54 |
| AK012074 | cdna clone 5-aminoimidazole-4-carboxamide ribonucleotide formyltransferase/imp cyclohydrolase | - | - | - | 0.54 |
| NM\_009226 | small nuclear ribonucleoprotein d1; snrpd1 | - | - | - | 0.54 |
| AF248546 | y-box protein 3 long isoform | - | - | - | 0.54 |
| AK018594 | cdna clone homolog to etaa16 protein | - | - | - | 0.54 |
| AF269250\_2 | pseudouridine synthase 1; pus1 | - | - | - | 0.54 |
| ENSMUSG00000034309 | ENSMUST00000044099 unknown | - | - | - | 0.54 |
| NM\_019716 | origin recognition complex; subunit 6-like (s. cerevisiae); orc6l | - | - | - | 0.54 |
| AF160990 | signaling lymphocytic activation molecule; slam | - | - | - | 0.53 |
| NM\_026030 | eukaryotic translation initiation factor 2; subunit 2 (beta; 38kda); eif2s2 | - | - | - | 0.53 |
| AF199010 | pals2-beta splice variant; pals2 | - | - | - | 0.53 |
| NM\_080639 | tissue inhibitor of metalloproteinase 4; timp4 | - | - | - | 0.53 |
| BC005734 | riken cdna 2610012o22 gene | - | - | - | 0.53 |
| NM\_013842 | x-box binding protein 1; xbp1 | - | - | - | 0.53 |
| NM\_023587 | protein tyrosine phosphatase-like protein ptplb; 6330408j20rik | - | - | - | 0.53 |
| NM\_053226 | vomeronasal 1 receptor; b3; v1rb3 | - | - | - | 0.53 |
| ENSMUSG00000042606 | ENSMUST00000037248 unknown | - | - | - | 0.53 |
| NM\_019499 | mad2 (mitotic arrest deficient; homolog)-like 1 (yeast); mad2l1 | - | - | - | 0.53 |
| AY073841 | olfactory receptor mor32-8 | - | - | - | 0.53 |
| NM\_016866 | serine/threonine kinase 39; ste20/sps1 homolog (yeast); stk39 | - | - | - | 0.53 |
| ENSMUSG00000041360 | ENSMUST00000047506 unknown | - | - | - | 0.53 |
| NM\_022724 | suppressor of variegation 3-9 homologue 2 (drosophila); suv39h2 | - | - | - | 0.53 |
| AF261919 | spindle assembly checkpoint protein; mad2a | - | - | - | 0.53 |
| ENSMUSG00000021532 | ENSMUST00000022005 unknown | - | - | - | 0.53 |
| ENSMUSG00000020014 | ENSMUST00000020200 ambiguous [source:ensembl\_protein\_families;acc:ensmusf00000008872]; ambiguous | - | - | - | 0.53 |
| AK010455 | similar to gats protein | - | - | - | 0.53 |
| BC026592 | similar to solute carrier family 4 (anion exchanger); member 4 | - | - | - | 0.53 |
| ENSMUSG00000020307 | ENSMUST00000020550 ubiquitin conjugating enzyme e2.32 kda complementing ec 6.3.2.19 ubiquitin protein ligase ubiquitin carrier protein e2 [source:ensembl\_protein\_families;acc:ensmusf00000004632]; ubiquitin conjugating enzyme e2.32 kda complementing ec 6.3 | - | - | - | 0.53 |
| NM\_013562 | interferon-related developmental regulator 1; ifrd1 | - | - | - | 0.53 |
| BC004733 | unknown (protein for mgc:7905) | - | - | - | 0.53 |
| NM\_008303 | heat shock 10 kda protein 1 (chaperonin 10); hspe1 | - | - | - | 0.53 |
| BC014703 | similar to u3 snornp-associated 55-kda protein | - | - | - | 0.53 |
| NM\_031201 | tissue specific transplantation antigen p35b; tstap35b | - | - | - | 0.53 |
| ENSMUSG00000030062 | ENSMUST00000032143 similar to ribophorin i. [source:sptrembl;acc:q91yq5]; dolichyl diphosphooligosaccharide protein glycosyltransferase 67 kda subunit precursor ec 2.4.1.119 ribophorin i rpn | - | - | - | 0.53 |
| BC013720 | unknown (protein for mgc:12121) | - | - | - | 0.53 |
| NM\_013882 | g two s phase expressed protein 1; gtse1 | - | - | - | 0.53 |
| AK014046 | related to hypothetical 15.0 kda protein | - | - | - | 0.52 |
| ENSMUSG00000001091 | ENSMUST00000001118 unknown | - | - | - | 0.52 |
| X64550 | ha receptor for hyaluronic acid; rhamm | - | - | - | 0.52 |
| X55499 | immunoglobulin enhancer binding protein; ig/ebp-1 | - | - | - | 0.52 |
| AK019247 | cdna clone homolog to ny-ren-41 antigen (fragment) | - | - | - | 0.52 |
| AK016522 | homolog to cdna: flj22612 fis; clone hsi04965 | - | - | - | 0.52 |
| ENSMUSG00000033747 | ENSMUST00000036681 putative transmembrane receptor [source:ensembl\_protein\_families;acc:ensmusf00000013620]; putative transmembrane receptor | - | - | - | 0.52 |
| M29395 | orotidine-5'-monophosphate decarboxylase | - | - | - | 0.52 |
| AF424698 | uridindiphosphoglucosepyrophosphorylase 2; ugp2 | - | - | - | 0.52 |
| NM\_008457 | kallikrein 8; klk8 | - | - | - | 0.52 |
| NM\_025310 | ectoplacental cone; invasive trophoblast giant cells; extraembryonic ectoderm and chorion sequence 3; epcs3-pending | - | - | - | 0.52 |
| NM\_007481 | adp-ribosylation factor 6; arf6 | - | - | - | 0.52 |
| AF115517 | survivin121 | - | - | - | 0.52 |
| ENSMUSG00000025899 | ENSMUST00000027030 unknown | - | - | - | 0.52 |
| NM\_010792 | methyltransferase-like 1 (s. cerevisiae); mettl1 | - | - | - | 0.52 |
| AK002554 | mrps18b | - | - | - | 0.52 |
| AF080580 | clk-1 | - | - | - | 0.52 |
| AF349432\_2 | testis-specific histone binding protein nasp; nasp | - | - | - | 0.52 |
| AK014640 | homolog to high-mobility group protein 2-like 1 (hmgbcg protein) | - | - | - | 0.52 |
| NM\_017393 | caseinolytic protease; atp-dependent; proteolytic subunit homolog (e. coli); clpp | - | - | - | 0.52 |
| AK010848 | cdna clone homolog to chromosome-associated polypeptide-c | - | - | - | 0.52 |
| NM\_011892 | sarcoglycan; gamma (35kd dystrophin-associated glycoprotein); sgcg | - | - | - | 0.52 |
| ENSMUSG00000033991 | ENSMUST00000046701 unknown | - | - | - | 0.52 |
| AK002894 | data source:sptr; source key:p52209; evidence:iss homolog to 6-phosphogluconate dehydrogenase; decarboxylating (ec 1.1.1.44) putative | - | - | - | 0.52 |
| U35641 | brca1 | - | - | - | 0.52 |
| ENSMUSG00000025049 | ENSMUST00000026027 wd repeat protein [source:ensembl\_protein\_families;acc:ensmusf00000000480]; wd repeat protein | - | - | - | 0.52 |
| BC011321 | similar to hypothetical protein flj20596 | - | - | - | 0.52 |
| BC003335 | similar to replication factor c (activator 1) 4 (37kd) | - | - | - | 0.52 |
| M68513 | receptor tyrosine kinase; mek4 | - | - | - | 0.52 |
| NM\_011499 | serine/threonine kinase receptor associated protein; strap | - | - | - | 0.52 |
| NM\_011799 | cell division cycle 6 homolog (s. cerevisiae); cdc6 | - | - | - | 0.52 |
| AK016771 | hypothetical protein | - | - | - | 0.51 |
| NM\_009391 | ran; member ras oncogene family; ran | - | - | - | 0.51 |
| NM\_011623 | topoisomerase (dna) ii alpha; top2a | - | - | - | 0.51 |
| BC004043 | unknown (protein for mgc:7672) | - | - | - | 0.51 |
| ENSMUSG00000015880 | ENSMUST00000016024 hypothetical 32.6 kda protein (fragment). [source:sptrembl;acc:q9jk28]; similar to chromosome condensation protein g | - | - | - | 0.51 |
| ENSMUSG00000026381 | ENSMUST00000027630 40s ribosomal protein s3a v fos transformation effector [source:ensembl\_protein\_families;acc:ensmusf00000000977]; 40s ribosomal protein s3a v fos transformation effector | - | - | - | 0.51 |
| AK016791 | similar to dna binding protein rfx1 | - | - | - | 0.51 |
| BC021623 | similar to histidine rich calcium binding protein | - | - | - | 0.51 |
| BC024730 | similar to dead/h (asp-glu-ala-asp/his) box polypeptide 27 | - | - | - | 0.51 |
| NM\_008869 | phospholipase a2; group iva (cytosolic; calcium-dependent); pla2g4a | - | - | - | 0.51 |
| NM\_011304 | ruvb-like protein 2; ruvbl2 | - | - | - | 0.51 |
| NM\_134073 | expressed sequence aa675328; aa675328 | - | - | - | 0.51 |
| J02935 | mouse camp-dependent protein kinase type ii regulatory subunit 3' end; ec 2.7.1.37 | - | - | - | 0.51 |
| NM\_016905 | galactokinase; glk | - | - | - | 0.51 |
| ENSMUSG00000027509 | ENSMUST00000029013 mrna associated protein mrnp 41 rae1 protein [source:ensembl\_protein\_families;acc:ensmusf00000006039]; mrna associated protein mrnp 41 rae1 protein | - | - | - | 0.51 |
| NM\_053082 | transmembrane 4 superfamily member 7; tm4sf7 | - | - | - | 0.51 |
| NM\_008799 | programmed cell death 2; pdcd2 | - | - | - | 0.51 |
| ENSMUSG00000033482 | ENSMUST00000046882 ambiguous [source:ensembl\_protein\_families;acc:ensmusf00000009502]; ambiguous | - | - | - | 0.51 |
| AK012148 | data source:sptr; source key:q9y448; evidence:iss homolog to traf4 associated factor 1 (fragment) putative | - | - | - | 0.51 |
| NM\_019736 | acyl-coenzyme a thioesterase 2; mitochondrial; acate2-pending | - | - | - | 0.51 |
| NM\_133933 | expressed sequence au018702; au018702 | - | - | - | 0.51 |
| NM\_025380 | eukaryotic translation elongation factor 1 epsilon 1; eef1e1 | - | - | - | 0.51 |
| AF249893 | phosphofructokinase-1 c isozyme; pfkc | - | - | - | 0.51 |
| AK017931 | homolog to hspc219 | - | - | - | 0.51 |
| NM\_133192 | g protein-coupled receptor 74; gpr74 | - | - | - | 0.51 |
| AK019451 | eif1a | - | - | - | 0.51 |
| ENSMUSG00000030059 | ENSMUST00000032140 tata element modulatory factor [source:ensembl\_protein\_families;acc:ensmusf00000008297]; tata element modulatory factor | - | - | - | 0.51 |
| AF477990 | cdt1 protein | - | - | - | 0.51 |
| AK017880 | homolog to chinese hamster ht protein | - | - | - | 0.51 |
| NM\_026438 | riken cdna 2010317e03; 2010317e03rik | - | - | - | 0.51 |
| NM\_009045 | avian reticuloendotheliosis viral (v-rel) oncogene homolog a; rela | - | - | - | 0.50 |
| BC002191 | similar to i-beta-1;3-n-acetylglucosaminyltransferase | - | - | - | 0.50 |
| BC025182 | unknown (protein for image:5035239) | - | - | - | 0.50 |
| NM\_010849 | myelocytomatosis oncogene; myc | - | - | - | 0.50 |
| ENSMUSG00000027200 | ENSMUST00000028625 semaphorin precursor semaphorin sema [source:ensembl\_protein\_families;acc:ensmusf00000001013]; semaphorin precursor semaphorin sema | - | - | - | 0.50 |
| NM\_133244 | membrane bound factor; mbf-pending | - | - | - | 0.50 |
| AK019977 | cdna clone homolog to chromosome-associated protein-e | - | - | - | 0.50 |
| NM\_008929 | dnaj (hsp40) homolog; subfamily c; member 3; dnajc3 | - | - | - | 0.50 |
| AF364579 | c1-tetrahydrofolate synthase; dcs | - | - | - | 0.50 |
| ENSMUSG00000041057 | ENSMUST00000046635 protein [source:ensembl\_protein\_families;acc:ensmusf00000010241]; protein | - | - | - | 0.50 |
| NM\_010715 | ligase i; dna; atp-dependent; lig1 | - | - | - | 0.50 |
| X03039\_3 | put. altern. eif-4a (aa 1-370) | - | - | - | 0.50 |
| ENSMUSG00000036777 | ENSMUST00000040912 unknown | - | - | - | 0.50 |
| NM\_019390 | lamin a; lmna | - | - | - | 0.50 |
| NM\_028232 | riken cdna 3300001m08; 3300001m08rik | - | - | - | 0.50 |
| ENSMUSG00000032493 | ENSMUST00000011392 serine protease-like 1. [source:sptrembl;acc:q924u6]; precursor | - | - | - | 0.50 |
| NM\_008198 | histocompatibility 2; complement component factor b; h2-bf | - | - | - | 0.50 |
| BC024921 | similar to hypothetical protein flj10858 | - | - | - | 0.50 |
| NM\_009505 | vascular endothelial growth factor a; vegfa | - | - | - | 0.50 |
| NM\_013646 | rar-related orphan receptor alpha; rora | - | - | - | 0.50 |
| ENSMUSG00000034349 | ENSMUST00000042901 chromosome assembly protein xcap [source:ensembl\_protein\_families;acc:ensmusf00000002747]; chromosome assembly protein xcap | - | - | - | 0.50 |
| AK015929 | unclassifiable | - | - | - | 0.50 |
| NM\_016661 | s-adenosylhomocysteine hydrolase; ahcy | - | - | - | 0.50 |
| BC011108 | unknown (protein for mgc:18986) | - | - | - | 0.50 |
| NM\_010444 | nuclear receptor subfamily 4; group a; member 1; nr4a1 | - | - | - | 0.49 |
| NM\_008298 | dnaj (hsp40) homolog; subfamily a; member 1; dnaja1 | - | - | - | 0.49 |
| NM\_011514 | suppressor of variegation 3-9 homolog 1 (drosophila); suv39h1 | - | - | - | 0.49 |
| NM\_013538 | gene rich cluster; c8 gene; grcc8 | - | - | - | 0.49 |
| NM\_019698 | pyrroline-5-carboxylate synthetase (glutamate gamma-semialdehyde synthetase); pycs | - | - | - | 0.49 |
| AK003179 | homolog to u-snrnp-associated cyclophilin (ec 5.2.1.8) | - | - | - | 0.49 |
| ENSMUSG00000039876 | ENSMUST00000036212 metabotropic glutamate receptor 8 precursor. [source:swissprot;acc:p47743]; metabotropic glutamate receptor precursor | - | - | - | 0.49 |
| ENSMUSG00000038106 | ENSMUST00000042824 ambiguous [source:ensembl\_protein\_families;acc:ensmusf00000005716]; ambiguous | - | - | - | 0.49 |
| AK010292 | homolog to ribonuclease hi large subunit (ec 3.1.26.-) (rnase hi large subunit) (rnase h(35)) (ribonuclease h2) (rnase h2) | - | - | - | 0.49 |
| ENSMUSG00000042708 | ENSMUST00000042373 unknown | - | - | - | 0.49 |
| BC026785 | unknown (protein for mgc:30237) | - | - | - | 0.49 |
| BC021337 | similar to hypothetical protein flj10407 | - | - | - | 0.49 |
| NM\_008565 | mini chromosome maintenance deficient 4 homolog (s. cerevisiae); mcmd4 | - | - | - | 0.49 |
| BC008614 | similar to riken cdna 4930527d15 gene | - | - | - | 0.49 |
| NM\_011660 | thioredoxin; txn1 | - | - | - | 0.49 |
| NM\_007573 | complement component 1; q subcomponent binding protein; c1qbp | - | - | - | 0.49 |
| ENSMUSG00000029246 | ENSMUST00000031159 amidophosphoribosyltransferase precursor ec 2.4.2.14 glutamine phosphoribosylpyrophosphate amidotransferase atase [source:ensembl\_protein\_families;acc:ensmusf00000004193]; amidophosphoribosyltransferase precursor ec 2.4.2.14 glutamine p | - | - | - | 0.49 |
| AJ237585 | hypothetical protein | - | - | - | 0.49 |
| AK006487 | hypothetical protein | - | - | - | 0.49 |
| NM\_133807 | expressed sequence aa959742; aa959742 | - | - | - | 0.49 |
| ENSMUSG00000025037 | ENSMUST00000041071 amine oxidase [flavin containing] ec 1.4.3.4 monoamine oxidase mao [source:ensembl\_protein\_families;acc:ensmusf00000001781]; amine oxidase [flavin containing] ec 1.4.3.4 monoamine oxidase mao | - | - | - | 0.49 |
| NM\_011467 | sepiapterin reductase; spr | - | - | - | 0.49 |
| ENSMUSG00000038412 | ENSMUST00000043270 hypoxia induced gene 1. [source:refseq;acc:nm\_019814]; ambiguous | - | - | - | 0.49 |
| AK014396 | homolog to copine vii | - | - | - | 0.48 |
| NM\_020616 | predicted gene icrfp703b1614q5.6; icrfp703b1614q5.6 | - | - | - | 0.48 |
| NM\_011631 | tumor rejection antigen gp96; tra1 | - | - | - | 0.48 |
| NM\_133905 | expressed sequence ai649009; ai649009 | - | - | - | 0.48 |
| AK005541 | homolog to probable trna (5-methylaminomethyl-2-thiouridylate)-methyltransferase (ec 2.1.1.61) | - | - | - | 0.48 |
| NM\_019468 | glucose-6-phosphate dehydrogenase 2; g6pd2 | - | - | - | 0.48 |
| NM\_007659 | cell division cycle 2 homolog a (s. pombe); cdc2a | - | - | - | 0.48 |
| AY073700 | olfactory receptor mor245-10 | - | - | - | 0.48 |
| AK020928 | homolog to melanoma antigen recognized by t-cells 1 (mart-1) (melan-a protein) (antigen sk29-aa) (antigen lb39-aa) | - | - | - | 0.48 |
| BC022907 | unknown (protein for mgc:25558) | - | - | - | 0.48 |
| ENSMUSG00000039619 | ENSMUST00000049440 unknown | - | - | - | 0.48 |
| NM\_020558 | nuclear dna-binding protein; c1d-pending | - | - | - | 0.48 |
| NM\_007547 | protein tyrosine phosphatase; non-receptor type substrate; ptpns1 | - | - | - | 0.48 |
| U89795 | protein kinase | - | - | - | 0.48 |
| AK003863 | homolog to kiaa-iso protein | - | - | - | 0.48 |
| NM\_008087 | growth arrest specific 2; gas2 | - | - | - | 0.48 |
| ENSMUSG00000035807 | ENSMUST00000037866 cg1/xap80 protein. [source:sptrembl;acc:q9qy60]; cg1 protein | - | - | - | 0.48 |
| ENSMUSG00000008110 | ENSMUST00000008254 glyceraldehyde 3 phosphate ec 1.2.1.12 [source:ensembl\_protein\_families;acc:ensmusf00000000166]; glyceraldehyde 3 phosphate ec 1.2.1.12 | - | - | - | 0.48 |
| NM\_031999 | transmembrane 7 superfamily member 1; tm7sf1 | - | - | - | 0.48 |
| NM\_026411 | riken cdna 1700021f05; 1700021f05rik | - | - | - | 0.48 |
| AK002601 | vezf1 | - | - | - | 0.48 |
| U68526 | branched chain aminotransferase; bcatm | - | - | - | 0.48 |
| NM\_007793 | cystatin b; cstb | - | - | - | 0.48 |
| S37052 | vascular endothelial growth factor-3; vascular endothelial growth factor-3; vegf-3 | - | - | - | 0.48 |
| NM\_007918 | eukaryotic translation initiation factor 4e binding protein 1; eif4ebp1 | - | - | - | 0.48 |
| AF095722 | somatic histone binding protein nasp | - | - | - | 0.48 |
| NM\_007691 | checkpoint kinase 1 homolog (s. pombe); chek1 | - | - | - | 0.48 |
| AK010832 | data source:sptr; source key:q9h370; evidence:iss homolog to pro1512 putative | - | - | - | 0.48 |
| L13791 | c/atf | - | - | - | 0.48 |
| NM\_025928 | riken cdna 2600009m07; 2600009m07rik | - | - | - | 0.48 |
| NM\_007637 | chaperonin subunit 5 (epsilon); cct5 | - | - | - | 0.48 |
| NM\_019700 | pseudouridine synthase 1; pus1 | - | - | - | 0.48 |
| AK010734 | cdna clone homolog to bifunctional aminoacyl-trna synthetase [includes: glutamyl-trna synthetase (ec 6.1.1.17) (glutamate--trna ligase); prolyl-trna synthetase (ec 6.1.1.15) (proline--trna ligase)] | - | - | - | 0.48 |
| NM\_026418 | regulator of g-protein signalling 10; rgs10 | - | - | - | 0.47 |
| ENSMUSG00000042320 | ENSMUST00000042567 homeobox prospero like protein prox1 prox [source:ensembl\_protein\_families;acc:ensmusf00000002527]; homeobox prospero like protein prox1 prox | - | - | - | 0.47 |
| AK010658 | data source:sptr; source key:q9y3b9; evidence:iss homolog to human cgi-115 protein putative | - | - | - | 0.47 |
| ENSMUSG00000039055 | ENSMUST00000039949 ambiguous [source:ensembl\_protein\_families;acc:ensmusf00000007869]; ambiguous | - | - | - | 0.47 |
| NM\_009716 | activating transcription factor 4; atf4 | - | - | - | 0.47 |
| NM\_030887 | jun dimerization protein 2; jundp2-pending | - | - | - | 0.47 |
| AK017911 | homolog to bcm-like membrane protein precursor | - | - | - | 0.47 |
| ENSMUSG00000026662 | ENSMUST00000027973 selenide;water dikinase ec 2.7.9.3 selenophosphate synthetase selenium donor protein [source:ensembl\_protein\_families;acc:ensmusf00000003174]; selenide;water dikinase ec 2.7.9.3 selenophosphate synthetase selenium donor protein | - | - | - | 0.47 |
| NM\_026405 | riken cdna 2810011a17; 2810011a17rik | - | - | - | 0.47 |
| NM\_008885 | peripheral myelin protein; 22 kda; pmp22 | - | - | - | 0.47 |
| NM\_011803 | core promoter element binding protein; copeb | - | 0.47 | - | - |
| ENSMUSG00000039867 | ENSMUST00000036053 metabotropic glutamate receptor precursor [source:ensembl\_protein\_families;acc:ensmusf00000000347]; metabotropic glutamate receptor precursor | - | - | - | 0.47 |
| ENSMUSG00000041771 | ENSMUST00000047197 sodium/potassium/calcium exchanger 3 precursor na + /k + /ca 2+ exchange protein [source:ensembl\_protein\_families;acc:ensmusf00000005674]; sodium/potassium/calcium exchanger 3 precursor na + /k + /ca 2+ exchange protein | - | - | - | 0.47 |
| NM\_007406 | adenylate cyclase 7; adcy7 | - | - | - | 0.47 |
| BC005617 | similar to riken cdna 2610020g18 gene | - | - | - | 0.47 |
| AK016972 | hypothetical protein | - | - | - | 0.47 |
| NM\_008186 | general transcription factor ii h; polypeptide 1 (62kd subunit); gtf2h1 | - | - | - | 0.47 |
| ENSMUSG00000032849 | ENSMUST00000036554 multidrug resistance associated protein [source:ensembl\_protein\_families;acc:ensmusf00000000159]; multidrug resistance associated protein | - | - | - | 0.47 |
| NM\_130889 | proliferation related acidic leucine rich protein pal31; pal31 | - | - | - | 0.47 |
| NM\_008705 | expressed in non-metastatic cells 2; protein (nm23b); nme2 | - | - | - | 0.47 |
| NM\_033268 | actinin alpha 2; actn2 | - | - | - | 0.47 |
| L21027 | a10 | - | - | - | 0.47 |
| NM\_010924 | nicotinamide n-methyltransferase; nnmt | - | - | - | 0.47 |
| ENSMUSG00000031530 | ENSMUST00000033930 dual specificity protein phosphatase ec 3.1.3.48 ec 3.1.3.- 16 map kinase phosphatase mkp [source:ensembl\_protein\_families;acc:ensmusf00000000315]; dual specificity protein phosphatase ec 3.1.3.48 ec 3.1.3.- 16 map kinase phosphatase mk | - | - | - | 0.47 |
| NM\_011284 | replication protein a2; rpa2 | - | - | - | 0.47 |
| ENSMUSG00000036281 | ENSMUST00000035427 cdna fis; clone ; weakly similar to homo sapiens snrna activating protein complex 190kd subunit snap190 [source:ensembl\_protein\_families;acc:ensmusf00000010431]; cdna fis; clone ; weakly similar to homo sapiens snrna activating protei | - | - | - | 0.47 |
| BC005799 | similar to hypothetical protein flj20354 | - | - | - | 0.47 |
| AK021194 | cdna clone homolog to art-4 protein | - | - | - | 0.46 |
| NM\_007900 | ect2 oncogene; ect2 | - | - | - | 0.46 |
| NM\_010783 | myod family inhibitor; mdfi | - | - | - | 0.46 |
| AK007900 | unclassifiable | - | - | - | 0.46 |
| NM\_013602 | metallothionein 1; mt1 | - | - | - | 0.46 |
| AK011289 | homolog to hypothetical 23.4 kda protein (fragment) | - | - | - | 0.46 |
| NM\_009791 | calmodulin binding protein 1; calmbp1 | - | - | - | 0.46 |
| NM\_011231 | rab geranylgeranyl transferase; b subunit; rabggtb | - | - | - | 0.46 |
| ENSMUSG00000040603 | ENSMUST00000042531 unknown | - | - | - | 0.46 |
| BC013673 | similar to tumor-associated antigen 1 | - | - | - | 0.46 |
| ENSMUSG00000031432 | ENSMUST00000033809 ribose phosphate pyrophosphokinase ec 2.7.6.1 phosphoribosyl pyrophosphate synthetase [source:ensembl\_protein\_families;acc:ensmusf00000001695]; ribose phosphate pyrophosphokinase ec 2.7.6.1 phosphoribosyl pyrophosphate synthetase | - | - | - | 0.46 |
| AK002567 | mt2 | - | - | - | 0.46 |
| AK011820 | f-box only protein 31 | - | - | - | 0.46 |
| M23384 | glucose transporter 1 | - | - | - | 0.46 |
| ENSMUSG00000032187 | ENSMUST00000035953 brg1 protein (fragment). [source:sptrembl;acc:o35845]; excision repair protein ercc 6 cockayne syndrome protein | - | - | - | 0.46 |
| NM\_013899 | translocase of inner mitochondrial membrane 13 homolog a (yeast); timm13a | - | - | - | 0.46 |
| BC013079 | similar to riken cdna 2600016b03 gene | - | - | - | 0.46 |
| AK016991 | wd domain; g-beta repeat containing protein | - | - | - | 0.46 |
| BC004835 | similar to hypothetical protein flj12806 | - | - | - | 0.46 |
| AK004619 | lmna | - | - | - | 0.46 |
| NM\_025923 | riken cdna 2010322c19; 2010322c19rik | - | - | - | 0.46 |
| NM\_023209 | t-cell-originated protein kinase; 2810434b10rik | - | - | - | 0.46 |
| AK012002\_76-1356 | similar to nuclear rna helicase bat1 | - | - | - | 0.46 |
| U86105 | nicotinamide n-methyltransferase; nnmt | - | - | - | 0.46 |
| NM\_026170 | riken cdna 1200007d18; 1200007d18rik | - | - | - | 0.46 |
| AK019636 | cdna clone hypothetical protein | - | - | - | 0.45 |
| BC027003 | similar to heterogeneous nuclear ribonucleoprotein h1 | - | - | - | 0.45 |
| ENSMUSG00000037736 | ENSMUST00000038188 ambiguous [source:ensembl\_protein\_families;acc:ensmusf00000009529]; ambiguous | - | - | - | 0.45 |
| NM\_011369 | shc sh2-domain binding protein 1; shcbp1 | - | - | - | 0.45 |
| AK021408 | hypothetical protein | - | - | - | 0.45 |
| ENSMUSG00000032560 | ENSMUST00000035170 unknown | - | - | - | 0.45 |
| BC003255 | similar to map-kinase activating death domain | - | - | - | 0.45 |
| NM\_015744 | phosphodiesterase i/nucleotide pyrophosphatase 2; enpp2 | - | - | - | 0.45 |
| NM\_018757 | expressed in non-metastatic cells 6; protein; nme6 | - | - | - | 0.45 |
| NM\_031202 | tyrosinase-related protein 1; tyrp1 | - | - | - | 0.45 |
| NM\_016662 | max dimerization protein 3; mad3 | - | - | - | 0.45 |
| S38083 | vascular endothelial growth factor-1; vascular endothelial growth factor-1; vegf-1 | - | - | - | 0.45 |
| AK014330 | data source:sptr; source key:o14929; evidence:iss homolog to histone acetyltransferase type b catalytic subunit (ec 2.3.1.48) putative | - | - | - | 0.45 |
| D17583 | pc6b | - | - | - | 0.45 |
| NM\_011132 | dna polymerase epsilon; pole | - | - | - | 0.45 |
| AK010905 | cdna clone homolog to hypothetical 31.3 kda protein | - | - | - | 0.45 |
| NM\_053180 | cdk-related protein kinase pnqlare; pnqlare | - | - | - | 0.45 |
| AK016062 | homolog to antisense rna overlapping mch protein | - | - | - | 0.45 |
| AF399831 | tim3 | - | - | - | 0.45 |
| AK004258 | similar to ultra-high sulphur keratin | - | - | - | 0.44 |
| NM\_025568 | riken cdna 2610002k22; 2610002k22rik | - | - | - | 0.44 |
| NM\_008892 | dna polymerase alpha 1; 180 kda; pola1 | - | - | - | 0.44 |
| NM\_010516 | cysteine rich protein 61; cyr61 | - | - | - | 0.44 |
| NM\_011166 | prolactin-like protein b; prlpb | - | - | - | 0.44 |
| ENSMUSG00000038335 | ENSMUST00000045807 ambiguous [source:ensembl\_protein\_families;acc:ensmusf00000007868]; ambiguous | - | - | - | 0.44 |
| AB011255 | bh-pcdh-c | - | - | - | 0.44 |
| BC021530 | unknown (protein for mgc:38539) | - | - | - | 0.44 |
| ENSMUSG00000026091 | ENSMUST00000027260 10 kda heat shock protein; mitochondrial hsp10.10 kda chaperonin [source:ensembl\_protein\_families;acc:ensmusf00000001452]; 10 kda heat shock protein; mitochondrial hsp10.10 kda chaperonin | - | - | - | 0.44 |
| NM\_133900 | expressed sequence ai480570; ai480570 | - | - | - | 0.44 |
| BC016211 | riken cdna 1110018p21 gene | - | - | - | 0.44 |
| AK011136 | homolog to nucleolar rna-helicase (hypothetical 61.6 kda protein) (dead-box rna helicase) (putative nucleolar rna helicase) | - | - | - | 0.44 |
| NM\_018861 | neutral amino acid transporter; slc1a4 | - | - | - | 0.44 |
| ENSMUSG00000040357 | ENSMUST00000043361 unknown | - | - | - | 0.44 |
| AK017458 | cub domain containing protein | - | - | - | 0.44 |
| AF115517\_2 | survivin40 | - | - | - | 0.44 |
| AK011279 | homolog to cdna flj14264 fis; clone place1002004 (unknown) (protein for mgc:2603) | - | - | - | 0.44 |
| NM\_027719 | riken cdna 4933436e20; 4933436e20rik | - | - | - | 0.44 |
| ENSMUSG00000037139 | ENSMUST00000035636 myomesin kda titin associated protein kda connectin associated [source:ensembl\_protein\_families;acc:ensmusf00000001533]; myomesin kda titin associated protein kda connectin associated | - | - | - | 0.44 |
| NM\_007642 | cd28 antigen; cd28 | - | - | - | 0.44 |
| NM\_009584 | zuotin related factor 2; zrf2 | - | - | - | 0.44 |
| NM\_025979 | riken cdna 2700091h24; 2700091h24rik | - | - | - | 0.44 |
| BC010318 | similar to phosphoenolpyruvate carboxykinase 2 (mitochondrial) | - | - | - | 0.44 |
| ENSMUSG00000019949 | ENSMUST00000020108 unknown | - | - | - | 0.44 |
| AY065521 | vomeronasal receptor v1rg6; v1rg6 | - | - | - | 0.43 |
| M68515 | mek4 secreted | - | - | - | 0.43 |
| NM\_007517 | ancient ubiquitous protein; aup1 | - | - | - | 0.43 |
| ENSMUSG00000033031 | ENSMUST00000048374 unknown | - | - | - | 0.43 |
| NM\_007629 | cyclin b1; related sequence 1; ccnb1-rs1 | - | - | - | 0.43 |
| ENSMUSG00000025542 | ENSMUST00000026622 mitochondrial import inner membrane translocase subunit tim8 a deafness dystonia protein 1 [source:ensembl\_protein\_families;acc:ensmusf00000005203]; mitochondrial import inner membrane translocase subunit tim8 a deafness dystonia protei | - | - | - | 0.43 |
| BC024653 | unknown (protein for mgc:30588) | - | - | - | 0.43 |
| ENSMUSG00000020520 | ENSMUST00000037122 similar to putative udp-galnac:polypeptide n-acetylgalactosaminyltransferase t9. [source:sptrembl;acc:q91yj6]; polypeptide n acetylgalactosaminyltransferase ec 2.4.1.41 protein udp acetylgalactosaminyltransferase udp galnac:polypeptide; | - | - | - | 0.43 |
| ENSMUSG00000024298 | ENSMUST00000025153 zinc finger protein 101. [source:refseq;acc:nm\_009542]; zinc finger protein | - | - | - | 0.43 |
| AK018094 | unclassifiable | - | - | - | 0.42 |
| AB049954 | mitochondrial ribosomal protein s18b; mrps18b | - | - | - | 0.42 |
| ENSMUSG00000021418 | ENSMUST00000021855 ribonuclease p protein subunit p40 ec 3.1.26.5 rnasep protein p40 rnase p subunit [source:ensembl\_protein\_families;acc:ensmusf00000009978]; ribonuclease p protein subunit p40 ec 3.1.26.5 rnasep protein p40 rnase p subunit | - | - | - | 0.42 |
| NM\_008343 | insulin-like growth factor binding protein 3; igfbp3 | - | - | - | 0.42 |
| ENSMUSG00000026195 | ENSMUST00000027392 atp binding cassette; sub family a; member atp binding cassette transporter atp binding cassette [source:ensembl\_protein\_families;acc:ensmusf00000000263]; atp binding cassette; sub family a; member atp binding cassette transporter atp b | - | - | - | 0.42 |
| NM\_016776 | myb binding protein (p160) 1a; mybbp1a | - | - | - | 0.42 |
| ENSMUSG00000036824 | ENSMUST00000042670 unknown | - | - | - | 0.42 |
| AK017628 | homolog to cdna flj10618 fis; clone nt2rp2005465; weakly similar to mitochondrial carrier protein rim2 | - | - | - | 0.42 |
| ENSMUSG00000033912 | ENSMUST00000046291 nucleolin protein [source:ensembl\_protein\_families;acc:ensmusf00000001428]; nucleolin protein | - | - | - | 0.42 |
| NM\_009689 | baculoviral iap repeat-containing 5; birc5 | - | - | - | 0.42 |
| BC006740 | similar to leucine zipper; putative tumor suppressor 1 | - | - | - | 0.42 |
| AK008785 | data source:sptr; source key:o00566; evidence:iss homolog to u3 small nucleolar ribonucleoprotein protein mpp10 (m phase phosphoprotein 10) putative | - | - | - | 0.42 |
| BC005509 | similar to lactate dehydrogenase a | - | - | - | 0.42 |
| AK011417 | prostate cancer overexpressed gene 1 | - | - | - | 0.41 |
| U85414 | gamma-glutamylcysteine synthetase | - | - | - | 0.41 |
| AK004820 | homolog to lon protease homolog; mitochondrial precursor (ec 3.4.21.-) (lon protease-like protein) (lonp) (lonhs) | - | - | - | 0.41 |
| ENSMUSG00000030851 | ENSMUST00000014540 l-lactate dehydrogenase c chain (ec 1.1.1.27) (ldh-c) (ldh testis subunit) (ldh-x). [source:swissprot;acc:p00342]; l lactate dehydrogenase chain ec 1.1.1.27 ldh | - | - | - | 0.41 |
| AK017804 | mrpl15 | - | - | - | 0.41 |
| U52461 | bg | - | - | - | 0.41 |
| BC025863 | riken cdna 1200007o21 gene | - | - | - | 0.41 |
| BC010793 | riken cdna 5031409g22 gene | - | - | - | 0.41 |
| NM\_019585 | espin; espn | - | - | - | 0.41 |
| BC022135 | unknown (protein for mgc:37844) | - | - | - | 0.41 |
| ENSMUSG00000025403 | ENSMUST00000026470 serine hydroxymethyltransferase (ec 2.1.2.1) (serine methylase) (glycine hydroxymethyltransferase) (shmt). [source:sptrembl;acc:q9czn7]; serine hydroxymethyltransferase; ec 2.1.2.1 serine methylase glycine hydroxymethyltransferase | - | - | - | 0.41 |
| NM\_015774 | ero1-like (s. cerevisiae); ero1l | - | - | - | 0.41 |
| BC016095 | similar to hypothetical protein dkfzp434g2226 | - | - | - | 0.41 |
| NM\_011547 | transcription factor ap-2; alpha; tcfap2a | - | - | 0.41 | 0.60 |
| L00039 | c-myc | - | - | - | 0.41 |
| NM\_009112 | s100 calcium binding protein a10 (calpactin); s100a10 | - | - | - | 0.41 |
| X81633 | s17 | - | - | - | 0.41 |
| NM\_016904 | cdc28 protein kinase 1; cks1 | - | - | - | 0.41 |
| NM\_023323 | riken cdna 2810470k21; 2810470k21rik | - | - | - | 0.41 |
| AL078630\_8 | 573k1.5 (novel protein similar to worm; yeast and plant proteins); 573k1.5 | - | - | - | 0.40 |
| AK011792 | nfyb | - | - | - | 0.40 |
| AB054027 | kinesin superfamily protein 23; kif23 | - | - | - | 0.40 |
| BC019962 | riken cdna 5430431l06 gene | - | - | - | 0.40 |
| NM\_010442 | heme oxygenase (decycling) 1; hmox1 | - | - | - | 0.40 |
| NM\_026630 | riken cdna 2410116g06; 2410116g06rik | - | - | - | 0.40 |
| NM\_026041 | riken cdna 2810430m08; 2810430m08rik | - | - | - | 0.40 |
| ENSMUSG00000033901 | ENSMUST00000036026 ambiguous [source:ensembl\_protein\_families;acc:ensmusf00000003624]; ambiguous | - | - | - | 0.40 |
| NM\_028228 | pin2/trf1-interacting protein; pinx1-pending | - | - | - | 0.40 |
| ENSMUSG00000034538 | ENSMUST00000035778 zinc finger protein [source:ensembl\_protein\_families;acc:ensmusf00000000001]; zinc finger protein | - | - | - | 0.40 |
| AK011664 | related to hypothetical 24.2 kda protein c13a11.03 in chromosome i | - | - | - | 0.40 |
| AK016487 | homolog to cdna: flj23554 fis; clone lng09359 | - | - | - | 0.39 |
| AK014651 | prdx4 | - | - | - | 0.39 |
| BC019785 | similar to calcium binding protein; 140 kda | - | - | - | 0.39 |
| ENSMUSG00000037137 | ENSMUST00000040288 glyceraldehyde 3 phosphate ec 1.2.1.12 [source:ensembl\_protein\_families;acc:ensmusf00000000166]; glyceraldehyde 3 phosphate ec 1.2.1.12 | - | - | - | 0.39 |
| NM\_011498 | basic helix-loop-helix domain containing; class b2; bhlhb2 | - | - | - | 0.39 |
| ENSMUSG00000031262 | ENSMUST00000033615 leucine rich primary response protein 1 follicle stimulating hormone primary response [source:ensembl\_protein\_families;acc:ensmusf00000005807]; leucine rich primary response protein 1 follicle stimulating hormone primary response | - | - | - | 0.39 |
| NM\_010090 | dual specificity phosphatase 2; dusp2 | - | - | - | 0.39 |
| AF259674 | phosphoserine aminotransferase | - | - | - | 0.39 |
| AF032875 | protein kinase; chk1 | - | - | - | 0.39 |
| NM\_007945 | epidermal growth factor receptor pathway substrate 8; eps8 | - | - | - | 0.39 |
| M69293 | id2 protein; id-2 | - | 0.45 | - | 0.39 |
| NM\_010699 | lactate dehydrogenase 1; a chain; ldh1 | - | - | - | 0.38 |
| BC022900 | similar to riken cdna 1110002o23 gene | - | - | - | 0.38 |
| AK011481 | cdna clone homolog to dj686c3.2 (nucleolar protein nop56) | - | - | - | 0.38 |
| ENSMUSG00000034773 | ENSMUST00000036548 unknown | - | - | - | 0.38 |
| NM\_024169 | riken cdna 1110002o23; 1110002o23rik | - | - | - | 0.37 |
| NM\_009375 | thyroglobulin; tgn | - | - | - | 0.37 |
| NM\_028712 | rap2b; member of ras oncogene family; rap2b | - | - | - | 0.37 |
| BC006605 | unknown (protein for mgc:7083) | - | - | - | 0.36 |
| NM\_008576 | atp-binding cassette; sub-family c; member 1a; abcc1a | - | - | - | 0.36 |
| AK009946 | homolog to rat growth and transformation-dependent (fragment) | - | - | - | 0.36 |
| NM\_030696 | monocarboxylate transporter 4; mct4 | - | - | - | 0.36 |
| ENSMUSG00000020228 | ENSMUST00000020449 dna helicase b. [source:sptrembl;acc:q9eqt8]; ambiguous | - | - | - | 0.36 |
| BC005657 | unknown (protein for mgc:12123) | - | - | - | 0.36 |
| AK002702 | cdna clone interferon-related developmental regulator 2 | - | - | - | 0.36 |
| AK012825 | evidence:nas putative unclassifiable transcript | - | - | - | 0.36 |
| AF153440 | nma | - | - | - | 0.36 |
| AB047007 | mmblr | - | - | - | 0.36 |
| NM\_008577 | solute carrier family 3 (activators of dibasic and neutral amino acid transport); member 2; slc3a2 | - | - | - | 0.35 |
| NM\_011812 | fibulin 5; fbln5 | - | - | - | 0.35 |
| NM\_007585 | calpactin i heavy chain; anxa2 | - | - | - | 0.35 |
| AK019097 | homolog to cdna flj13490 fis; clone place1004118 | - | - | - | 0.35 |
| AK012563 | anxa2 | - | - | - | 0.35 |
| AK013116 | homolog to hypothetical protein kiaa0186 | - | - | - | 0.35 |
| BC025169 | riken cdna 1810008k03 gene | - | - | - | 0.35 |
| BC004825 | riken cdna 2700043d08 gene | - | - | - | 0.35 |
| NM\_018826 | iroquois related homeobox 5 (drosophila); irx5 | - | - | - | 0.35 |
| NM\_022331 | homocysteine-inducible; endoplasmic reticulum stress-inducible; ubiquitin-like domain member 1; herpud1 | - | - | - | 0.35 |
| ENSMUSG00000031700 | ENSMUST00000034136 alanine aminotransferase ec 2.6.1.2 glutamic pyruvic transaminase gpt glutamic alanine [source:ensembl\_protein\_families;acc:ensmusf00000005326]; alanine aminotransferase ec 2.6.1.2 glutamic pyruvic transaminase gpt glutamic alanine | - | - | - | 0.35 |
| NM\_019703 | phosphofructokinase; platelet; pfkp | - | - | - | 0.34 |
| NM\_010481 | heat shock protein; 74 kda; a; hspa9a | - | - | - | 0.34 |
| AK019132 | data source:sptr; source key:q9nyl4; evidence:iss homolog to fk506 binding protein precursor putative | - | - | - | 0.34 |
| ENSMUSG00000035273 | ENSMUST00000045629 heparanase like protein [source:ensembl\_protein\_families;acc:ensmusf00000006546]; heparanase like protein | - | - | - | 0.34 |
| NM\_009272 | spermidine synthase; srm | - | - | - | 0.34 |
| ENSMUSG00000028330 | ENSMUST00000030014 80 kda nuclear cap binding protein ncbp 80 kda subunit [source:ensembl\_protein\_families;acc:ensmusf00000009719]; 80 kda nuclear cap binding protein ncbp 80 kda subunit | - | - | - | 0.34 |
| AK014990 | data source:sptr; source key:o95803; evidence:iss homolog to heparan sulfate n-deacetylase/n-sulfotransferase 3 putative | - | - | - | 0.34 |
| NM\_007699 | cholinergic receptor; muscarinic 4; chrm4 | - | - | - | 0.34 |
| NM\_134092 | expressed sequence ai429604; ai429604 | - | - | - | 0.34 |
| BC027063 | unknown (protein for image:5051325) | - | - | - | 0.34 |
| NM\_008939 | protease; serine; 12 neurotrypsin; (motopsin); prss12 | - | - | - | 0.34 |
| NM\_019939 | membrane protein; palmitoylated 3 (maguk p55 subfamily member 6); mpp6 | - | - | - | 0.34 |
| AK015007 | homolog to ny-ren-58 antigen | - | - | - | 0.33 |
| NM\_057173 | lim only 1; lmo1 | - | - | - | 0.33 |
| NM\_009472 | unc-5 homolog (c. elegans) 3; unc5h3 | - | - | - | 0.32 |
| AK002480\_18-1208 | homolog to cystathionine gamma-lyase (ec 4.4.1.1) (gamma-cystathionase) | - | - | - | 0.32 |
| AK021193 | cdna clone related to msf1 protein | - | - | - | 0.32 |
| AY073456 | olfactory receptor mor174-11 | - | - | - | 0.32 |
| AK007675 | evidence:nas hypothetical protein putative | - | - | - | 0.32 |
| NM\_010634 | keratinocyte lipid binding protein; fabp5 | - | - | - | 0.31 |
| L11330 | protein tyrosine phosphatase | - | - | - | 0.30 |
| NM\_013898 | translocase of inner mitochondrial membrane 8 homolog a (yeast); timm8a | - | - | - | 0.29 |
| ENSMUSG00000042354 | ENSMUST00000037739 cdna fis; clone ; weakly similar to autoantigen ngp [source:ensembl\_protein\_families;acc:ensmusf00000007348]; cdna fis; clone ; weakly similar to autoantigen ngp | - | - | - | 0.29 |
| AK006468 | unclassifiable | - | - | - | 0.29 |
| BC012955 | unknown (protein for mgc:18731) | - | - | - | 0.28 |
| NM\_010496 | inhibitor of dna binding 2; idb2 | - | - | 0.28 | 0.39 |
| AF321826 | beta-1;3-galactosyltransferase-related protein | - | - | - | 0.27 |
| ENSMUSG00000033397 | ENSMUST00000038084 zinc finger protein [source:ensembl\_protein\_families;acc:ensmusf00000019372]; zinc finger protein | - | - | - | 0.27 |
| ENSMUSG00000040391 | ENSMUST00000043581 l lactate dehydrogenase chain ec 1.1.1.27 ldh [source:ensembl\_protein\_families;acc:ensmusf00000000202]; l lactate dehydrogenase chain ec 1.1.1.27 ldh | - | - | - | 0.27 |
| AK014608 | homolog to cdna flj13936 fis; clone y79aa1000802 (fragment) | - | - | - | 0.26 |
| NM\_009951 | insulin-like growth factor 2; binding protein 1; igf2bp1 | - | - | - | 0.26 |
| ENSMUSG00000023468 | ENSMUST00000024234 binding protein [source:ensembl\_protein\_families;acc:ensmusf00000000155]; binding protein | - | - | - | 0.25 |
| AK011942 | cdna clone homolog to dj383j4.3 (a putative novel protein) (fragment) | - | - | - | 0.25 |
| AK011883 | cdna clone cyclin e2 | - | - | - | 0.24 |
| ENSMUSG00000035183 | ENSMUST00000044136 sodium/potassium/calcium exchanger 1 precursor na + /k + /ca 2+ exchange protein 1 retinal rod na ca+k [source:ensembl\_protein\_families;acc:ensmusf00000001598]; sodium/potassium/calcium exchanger 1 precursor na + /k + /ca 2+ exchange pr | - | - | - | 0.23 |
| U32446 | breast/ovarian cancer susceptibility protein homolog; brca1 | - | - | - | 0.20 |
| AJ314858\_2 | prolyl 4-hydroxylase alpha iia subunit; p4ha2 | - | - | - | 0.18 |
| NM\_026656 | riken cdna 3300002c04; 3300002c04rik | - | - | - | 0.18 |
| NM\_031378 | melanoma-derived leucine zipper; extra-nuclear factor; mlze | - | - | 0.29 | 0.15 |
| ENSMUSG00000030717 | ENSMUST00000032961 p8 protein; nuclear proten 1. [source:refseq;acc:nm\_019738]; unknown | - | - | - | 0.15 |
| NM\_011844 | monoglyceride lipase; mgll | - | - | - | 0.14 |
