# Supplementary material for: Global analysis of gene expression changes during retinoic acid-induced growth arrest and differentiation of melanoma: comparison to differentially expressed genes in melanocytes vs melanoma
Source: BMC Genomics. 2008 Oct 11;9:478. doi: 10.1186/1471-2164-9-478 (PMC2572629; doi:10.1186/1471-2164-9-478)
Supplement: Additional file 2 — List of genes whose expression is significantly different between the melan-a and B16 cell lines. Statistical significance is determined by SAM with a false discovery rate of 10% and a minimum fold change of 1.5. Average fold changes across six replicates are reported. Gene IDs (first column) are linked to search pages at the National Center for Biotechnology Information. [file 1471-2164-9-478-S2.htm]

Supplemental Table 2.

Additional file 2. List of genes whose expression is
significantly different between the melan-a and B16 cell lines. Statistical
significance was determined by SAM with a false discovery rate of 10% and a
minimum fold change of 1.5. Average fold changes across six replicates are
reported. Gene IDs (first column) are linked to search pages at the National Center for Biotechnology
Information.

| Name | Description | melan-a expression relative to B16 |
| --- | --- | --- |
| NM\_172884 | Mus musculus RIKEN cDNA 2900026A02 gene (2900026A02Rik), mRNA | 15.929 |
| NM\_013492 | Mus musculus clusterin (Clu), mRNA | 15.769 |
| BC039931 | Mus musculus, Similar to AHNAK nucleoprotein (desmoyokin), clone IMAGE:3599271, mRNA | 14.746 |
| NM\_177790 | Mus musculus hypothetical protein A930006D11 (A930006D11), mRNA | 12.360 |
| NM\_030704 | Mus musculus heat shock 27kDa protein 8 (Hspb8), mRNA | 11.421 |
| X65157 | M.musculus mRNA for desmoyokin, partial. | 11.338 |
| BC021494 | Mus musculus RIKEN cDNA 1110004P15 gene, mRNA (cDNA clone IMAGE:5345735), partial cds. | 11.154 |
| NM\_133891 | Mus musculus expressed sequence AW547365 (AW547365), mRNA | 10.757 |
| NM\_009465 | Mus musculus AXL receptor tyrosine kinase (Axl), mRNA | 10.379 |
| NM\_026336 | Mus musculus RIKEN cDNA 2310057J18 gene (2310057J18Rik), mRNA | 10.172 |
| X59289 | Mouse Xist (X inactive specific transcript) mRNA for open reading frame. | 10.150 |
| NM\_181728 | Mus musculus ADP-ribosyltransferase 3 (Art3), mRNA | 9.665 |
| NM\_009263 | Mus musculus secreted phosphoprotein 1 (Spp1), mRNA | 9.436 |
| NM\_029614 | Mus musculus RIKEN cDNA 2310046G15 gene (2310046G15Rik), mRNA | 9.241 |
| NM\_021278 | Mus musculus thymosin, beta 4, X chromosome (Tmsb4x), mRNA | 9.166 |
| NM\_010741 | Mus musculus lymphocyte antigen 6 complex, locus C (Ly6c), mRNA | 8.683 |
| NM\_010795 | Mus musculus mannoside acetylglucosaminyltransferase 3 (Mgat3), mRNA | 8.144 |
| NM\_012043 | Mus musculus immunoglobulin superfamily containing leucine-rich repeat (Islr), mRNA | 7.873 |
| NM\_010233 | Mus musculus fibronectin 1 (Fn1), mRNA | 7.265 |
| NM\_007742 | Mus musculus procollagen, type I, alpha 1 (Col1a1), mRNA | 7.185 |
| NM\_011313 | Mus musculus S100 calcium binding protein A6 (calcyclin) (S100a6), mRNA | 7.143 |
| NM\_013813 | Mus musculus erythrocyte protein band 4.1-like 3 (Epb4.1l3), mRNA | 7.128 |
| NM\_007929 | Mus musculus epithelial membrane protein 2 (Emp2), mRNA | 6.918 |
| AA733629 | AA733629 vu74b08.r1 Stratagene mouse skin (#937313) Mus musculus cDNA clone IMAGE:1197111 5' similar to gb:K02782 Mouse complement component C3 mRNA, alpha and beta subunits, (MOUSE);, mRNA sequence | 6.794 |
| AK004653 | Mus musculus adult male lung cDNA, RIKEN full-length enriched library, clone:1200008J08 product:thioredoxin interacting protein, full insert sequence. | 6.739 |
| NM\_011780 | Mus musculus a disintegrin and metalloprotease domain 23 (Adam23), mRNA | 6.662 |
| NM\_011526 | Mus musculus transgelin (Tagln), mRNA | 6.576 |
| TC1026197 | Unknown | 6.386 |
| NM\_019564 | Mus musculus protease, serine, 11 (Igf binding) (Prss11), mRNA | 6.344 |
| NM\_177836 | Mus musculus expressed sequence AW046396 (AW046396), mRNA | 6.333 |
| BC055908 | Mus musculus cDNA clone IMAGE:3602963, partial cds. | 6.296 |
| NM\_008530 | Mus musculus lymphocyte antigen 6 complex, locus F (Ly6f), mRNA | 6.276 |
| NM\_177037 | Mus musculus RIKEN cDNA A230059G12 gene (A230059G12Rik), mRNA | 6.132 |
| NM\_010884 | Mus musculus N-myc downstream regulated gene 1 (Ndrg1), mRNA | 6.123 |
| NM\_008610 | Mus musculus matrix metalloproteinase 2 (Mmp2), mRNA | 6.115 |
| AK014216 | Mus musculus 13 days embryo head cDNA, RIKEN full-length enriched library, clone:3110054C06 product:unknown EST, full insert sequence. | 6.111 |
| NM\_026125 | Mus musculus RIKEN cDNA 1110035L05 gene (1110035L05Rik), mRNA | 6.094 |
| NM\_011435 | Mus musculus superoxide dismutase 3, extracellular (Sod3), mRNA | 6.058 |
| NM\_011170 | Mus musculus prion protein (Prnp), mRNA | 6.038 |
| NM\_009373 | Mus musculus transglutaminase 2, C polypeptide (Tgm2), mRNA | 6.030 |
| NM\_023049 | Mus musculus ankyrin repeat and SOCS box-containing protein 2 (Asb2), mRNA | 5.924 |
| NM\_197986 | Mus musculus RIKEN cDNA 1110007F12 gene (1110007F12Rik), mRNA | 5.864 |
| AK007241 | Mus musculus adult male testis cDNA, RIKEN full-length enriched library, clone:1700122O11 product:hypothetical protein, full insert sequence. | 5.863 |
| NM\_021879 | Mus musculus pink-eyed dilution (p), mRNA | 5.858 |
| NM\_017372 | Mus musculus lysozyme (Lyzs), mRNA | 5.609 |
| NM\_054077 | Mus musculus proline arginine-rich end leucine-rich repeat (Prelp), mRNA | 5.592 |
| NM\_010701 | Mus musculus leukocyte cell derived chemotaxin 1 (Lect1), mRNA | 5.565 |
| NM\_026880 | Mus musculus PTEN induced putative kinase 1 (Pink1), mRNA | 5.470 |
| NM\_008242 | Mus musculus forkhead box D1 (Foxd1), mRNA | 5.463 |
| AK015518 | Mus musculus adult male testis cDNA, RIKEN full-length enriched library, clone:4930467D21 product:unknown EST, full insert sequence | 5.382 |
| NM\_007657 | Mus musculus CD9 antigen (Cd9), mRNA | 5.297 |
| NM\_007695 | Mus musculus chitinase 3-like 1 (Chi3l1), mRNA | 5.233 |
| C77713 | C77713 C77713 Mouse 3.5-dpc blastocyst cDNA Mus musculus cDNA clone J0036E01 3', mRNA sequence | 5.140 |
| NM\_029770 | Mus musculus unc-5 homolog B (C. elegans) (Unc5b), mRNA | 5.085 |
| NM\_016762 | Mus musculus matrilin 2 (Matn2), mRNA | 5.053 |
| AK020483 | Mus musculus 12 days embryo embryonic body between diaphragm region and neck cDNA, RIKEN full-length enriched library, clone:9430072K23 product:unknown EST, full insert sequence. | 5.050 |
| NM\_011340 | Mus musculus serine (or cysteine) proteinase inhibitor, clade F, member 1 (Serpinf1), mRNA | 5.042 |
| AK077477 | Mus musculus 8 days embryo whole body cDNA, RIKEN full-length enriched library, clone:5730419J04 product:insulin-like growth factor binding protein 3, full insert sequence. | 5.032 |
| NM\_053096 | Mus musculus camello-like 2 (Cml2), mRNA | 5.030 |
| AK034066 | Mus musculus adult male diencephalon cDNA, RIKEN full-length enriched library, clone:9330153E02 product:hypothetical BTB | 4.988 |
| NM\_024263 | Mus musculus RIKEN cDNA 1200013A08 gene (1200013A08Rik), mRNA | 4.922 |
| AK077243 | Mus musculus 11 days pregnant adult female ovary and uterus cDNA, RIKEN full-length enriched library, clone:5031412D17 product:interferon-induced protein with tetratricopeptide repeats 3, full insert sequence. | 4.906 |
| AK090111 | Mus musculus bladder RCB-0544 MBT-2 cDNA, RIKEN full-length enriched library, clone:G430136C21 product:unknown EST, full insert sequence. | 4.890 |
| NM\_013590 | Mus musculus P lysozyme structural (Lzp-s), mRNA | 4.850 |
| NM\_177280 | Mus musculus RIKEN cDNA B230206H07 gene (B230206H07Rik), mRNA | 4.794 |
| BC058124 | Mus musculus cDNA clone IMAGE:6815025, partial cds. | 4.794 |
| NM\_010580 | Mus musculus integrin beta 5 (Itgb5), mRNA | 4.754 |
| NM\_008086 | Mus musculus growth arrest specific 1 (Gas1), mRNA | 4.733 |
| AK010014 | Mus musculus adult male tongue cDNA, RIKEN full-length enriched library, clone:2310061N23 product:similar to ALPHA-INTERFERON INDUCIBLE PROTEIN (FRAGMENT) [Mesocricetus auratus], full insert sequence. | 4.726 |
| NM\_144938 | Mus musculus complement component 1, s subcomponent (C1s), mRNA | 4.717 |
| TC986224 | AP005082 MSHA biogenesis protein MshG {Vibrio parahaemolyticus}, partial (5%) | 4.643 |
| BC013561 | Mus musculus cDNA clone IMAGE:3492058, partial cds. | 4.604 |
| NM\_019946 | Mus musculus microsomal glutathione S-transferase 1 (Mgst1), mRNA | 4.554 |
| NM\_019503 | Mus musculus FXYD domain-containing ion transport regulator 1 (Fxyd1), transcript variant 1, mRNA | 4.547 |
| BC027183 | Mus musculus mRNA similar to complement component 1, s subcomponent (cDNA clone MGC:28492 IMAGE:4166254), complete cds | 4.514 |
| NM\_029537 | Mus musculus RIKEN cDNA 6530411B15 gene (6530411B15Rik), mRNA | 4.512 |
| TC1002499 | BC024495 secreted frizzled-related sequence protein 1 {Mus musculus}, complete | 4.451 |
| NM\_028266 | Mus musculus procollagen, type XVI, alpha 1 (Col16a1), mRNA | 4.381 |
| NM\_008655 | Mus musculus growth arrest and DNA-damage-inducible 45 beta (Gadd45b), mRNA | 4.376 |
| NM\_033590 | Mus musculus protocadherin gamma subfamily A, 7 (Pcdhga7), mRNA | 4.374 |
| NM\_008858 | Mus musculus protein kinase C, mu (Prkcm), mRNA | 4.324 |
| NM\_008548 | Mus musculus mannosidase 1, alpha (Man1a), mRNA | 4.242 |
| NM\_009260 | Mus musculus spectrin beta 2 (Spnb2), transcript variant 2, mRNA | 4.234 |
| NM\_009155 | Mus musculus selenoprotein P, plasma, 1 (Sepp1), mRNA | 4.233 |
| NM\_008695 | Mus musculus nidogen 2 (Nid2), mRNA | 4.220 |
| NM\_009931 | Mus musculus procollagen, type IV, alpha 1 (Col4a1), mRNA | 4.167 |
| NM\_009153 | Mus musculus sema domain, immunoglobulin domain (Ig), short basic domain, secreted, (semaphorin) 3B (Sema3b), mRNA | 4.164 |
| NM\_010501 | Mus musculus interferon-induced protein with tetratricopeptide repeats 3 (Ifit3), mRNA | 4.114 |
| NM\_023844 | Mus musculus junction adhesion molecule 2 (Jam2), mRNA | 4.103 |
| NM\_008223 | Mus musculus serine (or cysteine) proteinase inhibitor, clade D, member 1 (Serpind1), mRNA | 4.081 |
| NM\_030566 | Mus musculus rabaptin, RAB GTPase binding effector protein 2 (Rabep2), mRNA | 4.047 |
| AK122369 | Mus musculus mRNA for mKIAA0777 protein. | 4.045 |
| NM\_027533 | Mus musculus RIKEN cDNA 6330415F13 gene (6330415F13Rik), mRNA | 4.042 |
| NM\_023476 | Mus musculus lipocalin 7 (Lcn7), mRNA | 3.989 |
| AK129139 | Mus musculus mRNA for mKIAA0429 protein. | 3.975 |
| NM\_009735 | Mus musculus beta-2 microglobulin (B2m), mRNA | 3.971 |
| AY100452 | Mus musculus lmb145-cap1-243 protein precursor, mRNA, partial cds. | 3.964 |
| AK129230 | Mus musculus mRNA for mKIAA0857 protein. | 3.954 |
| AK090296 | Mus musculus 21 days neonate cerebellum cDNA, RIKEN full-length enriched library, clone:G630039L19 product:cytochrome P450, 2d22, full insert sequence | 3.954 |
| NM\_023118 | Mus musculus disabled homolog 2 (Drosophila) (Dab2), mRNA | 3.875 |
| NM\_026066 | Mus musculus chemokine-like factor super family 5 (Cklfsf5), mRNA | 3.863 |
| NM\_153155 | Mus musculus C1q-like (C1ql), mRNA | 3.830 |
| NM\_011921 | Mus musculus aldehyde dehydrogenase family 1, subfamily A7 (Aldh1a7), mRNA | 3.798 |
| NM\_007471 | Mus musculus amyloid beta (A4) precursor protein (App), mRNA | 3.786 |
| NM\_134006 | Mus musculus retinol dehydrogenase 5 (Rdh5), mRNA | 3.770 |
| AK007352 | Mus musculus 10 day old male pancreas cDNA, RIKEN full-length enriched library, clone:1810006K23 product:hypothetical protein, full insert sequence. | 3.767 |
| NM\_015734 | Mus musculus procollagen, type V, alpha 1 (Col5a1), mRNA | 3.765 |
| NM\_009079 | Mus musculus ribosomal protein L22 (Rpl22), mRNA | 3.741 |
| NAP069078-1 | Unknown | 3.738 |
| NM\_011057 | Mus musculus platelet derived growth factor, B polypeptide (Pdgfb), mRNA | 3.724 |
| AK040622 | Mus musculus 0 day neonate thymus cDNA, RIKEN full-length enriched library, clone:A430110F10 product:BCL-2 MODIFYING FACTOR homolog [Mus musculus], full insert sequence. | 3.710 |
| NM\_020258 | Mus musculus solute carrier family 37 (glycerol-3-phosphate transporter), member 2 (Slc37a2), mRNA | 3.696 |
| NM\_028535 | Mus musculus RIKEN cDNA 1700049E17 gene (1700049E17Rik), mRNA | 3.694 |
| NM\_011844 | Mus musculus monoglyceride lipase (Mgll), mRNA | 3.692 |
| NM\_015814 | Mus musculus dickkopf homolog 3 (Xenopus laevis) (Dkk3), mRNA | 3.689 |
| NM\_172872 | Mus musculus cDNA BC060737 (BC060737), mRNA | 3.675 |
| XM\_147957 | Mus musculus hypothetical protein D930017K21 (D930017K21), mRNA | 3.674 |
| AK122250 | Mus musculus mRNA for mKIAA0337 protein. | 3.672 |
| NM\_013506 | Mus musculus eukaryotic translation initiation factor 4A2 (Eif4a2), mRNA | 3.631 |
| NM\_008871 | Mus musculus serine (or cysteine) proteinase inhibitor, clade E, member 1 (Serpine1), mRNA | 3.630 |
| NR\_001463 | Mus musculus inactive X specific transcripts (Xist) on chromosome X, transcript variant 1 | 3.629 |
| AK020640 | Mus musculus adult male urinary bladder cDNA, RIKEN full-length enriched library, clone:9530073D23 product:unknown EST, full insert sequence. | 3.619 |
| NM\_015786 | Mus musculus histone 1, H1c (Hist1h1c), mRNA | 3.600 |
| NM\_013758 | Mus musculus adducin 3 (gamma) (Add3), mRNA | 3.599 |
| NM\_023249 | Mus musculus yippee-like 1 (Drosophila) (Ypel1), mRNA | 3.574 |
| NM\_194054 | Mus musculus reticulon 4 (Rtn4), transcript variant 1, mRNA | 3.567 |
| NM\_144556 | Mus musculus leucine-rich repeat LGI family, member 4 (Lgi4), mRNA | 3.554 |
| U37501 | Mus musculus laminin alpha 5 chain (Lama5) mRNA, partial cds. | 3.533 |
| NM\_008377 | Mus musculus leucine-rich repeats and immunoglobulin-like domains 1 (Lrig1), mRNA | 3.528 |
| NM\_145502 | Mus musculus similar to Caenorhabditis elegans protein C42C1.9 (Keo4), mRNA | 3.523 |
| AK008728 | Mus musculus adult male stomach cDNA, RIKEN full-length enriched library, clone:2210015B19 product:ankyrin repeat domain-containing SOCS box protein 13, full insert sequence. | 3.523 |
| NM\_009606 | Mus musculus actin, alpha 1, skeletal muscle (Acta1), mRNA | 3.508 |
| NAP007796-001 | Unknown | 3.501 |
| NM\_022420 | Mus musculus G protein-coupled receptor, family C, group 5, member B (Gprc5b), mRNA | 3.485 |
| NM\_172463 | Mus musculus secreted protein SST3 (SST3), mRNA | 3.485 |
| NM\_007472 | Mus musculus aquaporin 1 (Aqp1), mRNA | 3.475 |
| NM\_009685 | Mus musculus amyloid beta (A4) precursor protein-binding, family B, member 1 (Apbb1), mRNA | 3.466 |
| NM\_007731 | Mus musculus procollagen, type XIII, alpha 1 (Col13a1), mRNA | 3.462 |
| NM\_178726 | Mus musculus protein phosphatase 1 (formerly 2C)-like (Ppm1l), mRNA | 3.462 |
| NM\_008520 | Mus musculus latent transforming growth factor beta binding protein 3 (Ltbp3), mRNA | 3.459 |
| AK090301 | Mus musculus 10 days pregnant adult female ovary and uterus cDNA, RIKEN full-length enriched library, clone:G630042G04 product:hypothetical protein, full insert sequence. | 3.451 |
| NM\_199146 | Mus musculus tripartite motif protein 30-like (LOC209387), mRNA | 3.436 |
| BC034735 | Mus musculus RIKEN cDNA 2310047A01 gene, mRNA (cDNA clone IMAGE:4951483), partial cds | 3.426 |
| NM\_144811 | Mus musculus chromobox homolog 7 (Cbx7), mRNA | 3.426 |
| AF202039 | Mus musculus homeobox transcription factor (Nkx2-4) mRNA, partial cds. | 3.418 |
| NM\_008515 | Mus musculus leucine rich repeat (in FLII) interacting protein 1 (Lrrfip1), mRNA | 3.363 |
| NM\_011625 | Mus musculus protein phosphatase 1, regulatory (inhibitor) subunit 13B (Ppp1r13b), mRNA | 3.350 |
| NM\_025626 | Mus musculus RIKEN cDNA 3110001A13 gene (3110001A13Rik), mRNA | 3.322 |
| NM\_009099 | Mus musculus tripartite motif protein 30 (Trim30), mRNA | 3.322 |
| NM\_183274 | Mus musculus RIKEN cDNA 0610041G09 gene (0610041G09Rik), mRNA | 3.322 |
| NM\_011311 | Mus musculus S100 calcium binding protein A4 (S100a4), mRNA | 3.299 |
| NM\_010837 | Mus musculus microtubule-associated protein 6 (Mtap6), mRNA | 3.296 |
| NM\_153119 | Mus musculus expressed sequence AI840980 (AI840980), mRNA | 3.272 |
| NM\_146136 | Mus musculus solute carrier family 16 (monocarboxylic acid transporters), member 4 (Slc16a4), mRNA | 3.271 |
| AK049696 | Mus musculus 12 days embryo spinal cord cDNA, RIKEN full-length enriched library, clone:C530041K19 product:hypothetical protein, full insert sequence. | 3.268 |
| NM\_008744 | Mus musculus netrin 1 (Ntn1), mRNA | 3.248 |
| NM\_009504 | Mus musculus vitamin D receptor (Vdr), mRNA | 3.246 |
| NM\_010261 | Mus musculus Rab acceptor 1 (prenylated) (Rabac1), mRNA | 3.233 |
| ENSMUST00000067583 | Unknown | 3.229 |
| AK019477 | Mus musculus 0 day neonate skin cDNA, RIKEN full-length enriched library, clone:4631433M03 product:dystrobrevin alpha, full insert sequence. | 3.228 |
| NM\_008872 | Mus musculus plasminogen activator, tissue (Plat), mRNA | 3.215 |
| NAP026388-1 | Unknown | 3.209 |
| AK049901 | Mus musculus adult male hippocampus cDNA, RIKEN full-length enriched library, clone:C630010D07 product:unknown EST, full insert sequence. | 3.195 |
| NM\_198250 | Mus musculus leucine rich repeat containing 4B (Lrrc4b), mRNA | 3.184 |
| NM\_026993 | Mus musculus dimethylarginine dimethylaminohydrolase 1 (Ddah1), mRNA | 3.172 |
| AK173172 | Mus musculus 0 day neonate head cDNA, RIKEN full-length enriched library, clone:4833445A08 product:unknown EST, full insert sequence. [AK029482] | 3.162 |
| NM\_177834 | Mus musculus RIKEN cDNA 9030616D13 gene (9030616D13Rik), mRNA | 3.152 |
| NM\_008150 | Mus musculus glypican 4 (Gpc4), mRNA | 3.128 |
| NM\_213614 | Mus musculus septin 5 (Sept5), mRNA | 3.114 |
| AK009836 | Mus musculus adult male tongue cDNA, RIKEN full-length enriched library, clone:2310046A06 product:hypothetical protein, full insert sequence. | 3.089 |
| NM\_172597 | Mus musculus RIKEN cDNA 5730420B22 gene (5730420B22Rik), mRNA | 3.081 |
| NM\_008721 | Mus musculus neural proliferation, differentiation and control gene 1 (Npdc1), mRNA | 3.081 |
| NM\_013886 | Mus musculus hepatoma-derived growth factor, related protein 3 (Hdgfrp3), mRNA | 3.080 |
| AK005223 | Mus musculus adult male cerebellum cDNA, RIKEN full-length enriched library, clone:1500012A13 product:CLATHRIN COAT ASSEMBLY PROTEIN AP19 (CLATHRIN COAT ASSOCIATED PROTEIN AP19) (GOLGI ADAPTOR AP-1 19 KDA ADAPTIN) (HA1 19 KDA SUBUNIT) (CLATHRIN ASSEMBLY | 3.077 |
| NM\_007874 | Mus musculus deleted in polyposis 1 (Dp1), mRNA | 3.076 |
| NM\_007470 | Mus musculus apolipoprotein D (Apod), mRNA | 3.071 |
| NM\_181821 | Mus musculus host cell factor C1 regulator 1 (XPO1-dependent) (Hcfc1r1), mRNA | 3.068 |
| NM\_016696 | Mus musculus glypican 1 (Gpc1), mRNA | 3.064 |
| AK081810 | Mus musculus 16 days embryo head cDNA, RIKEN full-length enriched library, clone:C130078M18 product:fibroblast growth factor receptor 2, full insert sequence. | 3.064 |
| NM\_013494 | Mus musculus carboxypeptidase E (Cpe), mRNA | 3.062 |
| NM\_178644 | Mus musculus RIKEN cDNA D130038B21 gene (D130038B21Rik), mRNA | 3.058 |
| NM\_009273 | Mus musculus signal recognition particle 14 (Srp14), mRNA | 3.057 |
| NM\_013923 | Mus musculus ring finger protein (C3HC4 type) 19 (Rnf19), mRNA | 3.056 |
| AK030757 | Mus musculus 8 days embryo whole body cDNA, RIKEN full-length enriched library, clone:5730547A16 product:erythrocyte protein band 4.1-like 2, full insert sequence. | 3.046 |
| NM\_007778 | Mus musculus colony stimulating factor 1 (macrophage) (Csf1), mRNA | 3.046 |
| AK122378 | Mus musculus mRNA for mKIAA0818 protein. | 3.046 |
| AK013636 | Mus musculus adult male hippocampus cDNA, RIKEN full-length enriched library, clone:2900042B11 product:similar to HSPCO34 PROTEIN [Homo sapiens], full insert sequence. | 3.044 |
| NM\_023143 | Mus musculus complement component 1, r subcomponent (C1r), mRNA | 3.034 |
| AK014566 | Mus musculus 0 day neonate skin cDNA, RIKEN full-length enriched library, clone:4632411I06 product:TAP binding protein, full insert sequence. | 3.026 |
| AK122196 | Mus musculus mRNA for mKIAA0059 protein. | 3.025 |
| NM\_009368 | Mus musculus transforming growth factor, beta 3 (Tgfb3), mRNA | 3.024 |
| NM\_194347 | Mus musculus HN1-like (LOC278676), mRNA | 3.024 |
| NM\_013834 | Mus musculus secreted frizzled-related sequence protein 1 (Sfrp1), mRNA | 3.012 |
| NM\_011035 | Mus musculus p21 (CDKN1A)-activated kinase 1 (Pak1), mRNA | 3.011 |
| NM\_010101 | Mus musculus endothelial differentiation, sphingolipid G-protein-coupled receptor, 3 (Edg3), mRNA | 3.006 |
| NM\_009932 | Mus musculus procollagen, type IV, alpha 2 (Col4a2), mRNA | 3.004 |
| NM\_009136 | Mus musculus scrapie responsive gene 1 (Scrg1), mRNA | 3.004 |
| NM\_025367 | Mus musculus sphingosine kinase 1 (Sphk1), mRNA | 3.003 |
| NM\_145509 | Mus musculus RIKEN cDNA 5430435G22 gene (5430435G22Rik), mRNA | 2.996 |
| NM\_172694 | Mus musculus EGF-like-domain, multiple 5 (Egfl5), mRNA | 2.994 |
| AK087668 | Mus musculus 2 days pregnant adult female ovary cDNA, RIKEN full-length enriched library, clone:E330001K01 product:unclassifiable, full insert sequence. | 2.993 |
| NM\_011521 | Mus musculus syndecan 4 (Sdc4), mRNA | 2.987 |
| AK003880 | Mus musculus 18-day embryo whole body cDNA, RIKEN full-length enriched library, clone:1110020P09 product:hypothetical Histidine-rich region containing protein, full insert sequence. | 2.986 |
| NM\_013454 | Mus musculus ATP-binding cassette, sub-family A (ABC1), member 1 (Abca1), mRNA | 2.978 |
| NM\_010496 | Mus musculus inhibitor of DNA binding 2 (Idb2), mRNA | 2.969 |
| NM\_011892 | Mus musculus sarcoglycan, gamma (dystrophin-associated glycoprotein) (Sgcg), mRNA | 2.962 |
| XM\_354621 | Mus musculus similar to XIAP associated factor-1 (LOC380706), mRNA | 2.955 |
| NM\_008410 | Mus musculus integral membrane protein 2B (Itm2b), mRNA | 2.954 |
| NM\_013746 | Mus musculus pleckstrin homology domain containing, family B (evectins) member 1 (Plekhb1), mRNA | 2.942 |
| NM\_201410 | Mus musculus UDP glycosyltransferase 1 family polypeptide A9 (Ugt1a9), mRNA | 2.930 |
| NM\_013511 | Mus musculus erythrocyte protein band 4.1-like 2 (Epb4.1l2), mRNA | 2.926 |
| NM\_153484 | Mus musculus thyrotroph embryonic factor (Tef), transcript variant 2, mRNA | 2.922 |
| NM\_010509 | Mus musculus interferon (alpha and beta) receptor 2 (Ifnar2), mRNA | 2.922 |
| NM\_008862 | Mus musculus protein kinase inhibitor, alpha (Pkia), mRNA | 2.913 |
| NM\_018865 | Mus musculus WNT1 inducible signaling pathway protein 1 (Wisp1), mRNA | 2.912 |
| NM\_009807 | Mus musculus caspase 1 (Casp1), mRNA | 2.907 |
| AK008077 | Mus musculus adult male small intestine cDNA, RIKEN full-length enriched library, clone:2010003O02 product:unknown EST, full insert sequence. | 2.906 |
| NM\_133897 | Mus musculus RIKEN cDNA E430036I04 gene (E430036I04Rik), mRNA | 2.899 |
| NM\_011204 | Mus musculus protein tyrosine phosphatase, non-receptor type 13 (Ptpn13), mRNA | 2.890 |
| AK009517 | Mus musculus adult male tongue cDNA, RIKEN full-length enriched library, clone:2310026P19 product:hypothetical BTB/POZ domain containing protein, full insert sequence. | 2.886 |
| NM\_013469 | Mus musculus annexin A11 (Anxa11), mRNA | 2.883 |
| NM\_001001979 | Mus musculus RIKEN cDNA 3000002B06 gene (3000002B06Rik), mRNA | 2.881 |
| NM\_198113 | Mus musculus slingshot homolog 3 (Drosophila) (Ssh3), mRNA | 2.879 |
| BC004722 | Mus musculus RIKEN cDNA 2210401K01 gene, mRNA (cDNA clone IMAGE:3582796), partial cds | 2.868 |
| XM\_127032 | Mus musculus RIKEN cDNA 5730501N20 gene (5730501N20Rik), mRNA | 2.868 |
| NM\_025569 | Mus musculus microsomal glutathione S-transferase 3 (Mgst3), mRNA | 2.862 |
| NM\_019707 | Mus musculus cadherin 13 (Cdh13), mRNA | 2.861 |
| NM\_007621 | Mus musculus carbonyl reductase 2 (Cbr2), mRNA | 2.860 |
| AK008724 | Mus musculus adult male stomach cDNA, RIKEN full-length enriched library, clone:2210013O21 product:hypothetical protein, full insert sequence. | 2.859 |
| NM\_020590 | Mus musculus gamma-aminobutyric acid (GABA(A)) receptor-associated protein-like 1 (Gabarapl1), mRNA | 2.858 |
| NM\_198249 | Mus musculus RIKEN cDNA E130112L23 gene (E130112L23Rik), mRNA | 2.858 |
| NM\_026754 | Mus musculus RIKEN cDNA 1110017I16 gene (1110017I16Rik), mRNA | 2.857 |
| NM\_010156 | Mus musculus EST AA175286 (AA175286), mRNA | 2.838 |
| NM\_012021 | Mus musculus peroxiredoxin 5 (Prdx5), mRNA | 2.836 |
| AB041546 | Mus musculus brain cDNA, clone MNCb-3527, similar to AF220152 TACC2 (Homo sapiens). | 2.831 |
| NM\_213729 | Mus musculus expressed sequence AI842396 (AI842396), mRNA | 2.825 |
| NM\_153068 | Mus musculus EH-domain containing 2 (Ehd2), mRNA | 2.825 |
| AK029728 | Mus musculus adult male testis cDNA, RIKEN full-length enriched library, clone:4930513D10 product:hypothetical Tyrosine protein kinase/Serine/Threonine protein kinase/Eukaryotic protein kinase containing protein, full insert sequence. | 2.823 |
| NM\_009784 | Mus musculus calcium channel, voltage-dependent, alpha2/delta subunit 1 (Cacna2d1), mRNA | 2.819 |
| NM\_013478 | Mus musculus alpha-2-glycoprotein 1, zinc (Azgp1), mRNA | 2.810 |
| AK016466 | Mus musculus adult male testis cDNA, RIKEN full-length enriched library, clone:4931420C21 product:hypothetical SEC7-like domain/SEC7 domain profile containing protein, full insert sequence. | 2.806 |
| NM\_007569 | Mus musculus B-cell translocation gene 1, anti-proliferative (Btg1), mRNA | 2.803 |
| NM\_021439 | Mus musculus carbohydrate sulfotransferase 11 (Chst11), mRNA | 2.803 |
| NM\_178901 | Mus musculus expressed sequence AI467606 (AI467606), mRNA | 2.785 |
| NM\_173181 | Mus musculus RIKEN cDNA 3110050N22 gene (3110050N22Rik), mRNA | 2.777 |
| AI851065 | AI851065 UI-M-BH0-ajv-h-04-0-UI.s1 NIH\_BMAP\_M\_S1 Mus musculus cDNA clone UI-M-BH0-ajv-h-04-0-UI 3', mRNA sequence | 2.772 |
| NM\_028013 | Mus musculus RIKEN cDNA 2310067E08 gene (2310067E08Rik), mRNA | 2.772 |
| BC040364 | Mus musculus dystrobrevin alpha, transcript variant 1, mRNA (cDNA clone MGC:25316 IMAGE:4505583), complete cds. | 2.771 |
| NM\_178936 | Mus musculus RIKEN cDNA C730036B01 gene (C730036B01Rik), mRNA | 2.762 |
| NM\_007963 | Mus musculus ecotropic viral integration site 1 (Evi1), mRNA | 2.758 |
| BC055373 | Mus musculus cDNA clone MGC:60763 IMAGE:30058959, complete cds. | 2.754 |
| XM\_129028 | Mus musculus hypothetical protein G630054C21 (G630054C21), mRNA | 2.744 |
| NM\_011785 | Mus musculus thymoma viral proto-oncogene 3 (Akt3), mRNA | 2.743 |
| NM\_011339 | Mus musculus chemokine (C-X-C motif) ligand 15 (Cxcl15), mRNA | 2.736 |
| NM\_026436 | Mus musculus RIKEN cDNA 1810054O13 gene (1810054O13Rik), mRNA | 2.729 |
| NM\_133943 | Mus musculus hydroxy-delta-5-steroid dehydrogenase, 3 beta- and steroid delta-isomerase 7 (Hsd3b7), mRNA | 2.727 |
| BC029863 | Mus musculus cDNA clone MGC:36861 IMAGE:4460168, complete cds. | 2.724 |
| NAP029508-1 | Unknown | 2.721 |
| NM\_178688 | Mus musculus actin-binding LIM protein 1 (Ablim1), mRNA | 2.721 |
| NM\_011268 | Mus musculus regulator of G-protein signaling 9 (Rgs9), mRNA | 2.718 |
| AK019835 | Mus musculus adult male testis cDNA, RIKEN full-length enriched library, clone:4930590G08 product:par-6 partitioning defective 6 homolog gamma (C. elegans), full insert sequence. | 2.712 |
| NM\_023270 | Mus musculus ring finger protein 128 (Rnf128), mRNA | 2.701 |
| NM\_023053 | Mus musculus twisted gastrulation homolog 1 (Drosophila) (Twsg1), mRNA | 2.698 |
| NM\_181397 | Mus musculus RIKEN cDNA 2310015N21 gene (2310015N21Rik), mRNA | 2.695 |
| NM\_011303 | Mus musculus dehydrogenase/reductase (SDR family) member 3 (Dhrs3), mRNA | 2.693 |
| NM\_008048 | Mus musculus insulin-like growth factor binding protein 7 (Igfbp7), mRNA | 2.692 |
| BC055897 | Mus musculus natriuretic peptide receptor 3, mRNA (cDNA clone MGC:68237 IMAGE:4019152), complete cds. | 2.691 |
| NM\_178149 | Mus musculus RIKEN cDNA 1500004A08 gene (1500004A08Rik), mRNA | 2.683 |
| NM\_138661 | Mus musculus protocadherin alpha 9 (Pcdha9), mRNA | 2.672 |
| NM\_008397 | Mus musculus integrin alpha 6 (Itga6), mRNA | 2.666 |
| NM\_010260 | Mus musculus guanylate nucleotide binding protein 2 (Gbp2), mRNA | 2.662 |
| NM\_010128 | Mus musculus epithelial membrane protein 1 (Emp1), mRNA | 2.661 |
| AK045650 | Mus musculus adult male corpora quadrigemina cDNA, RIKEN full-length enriched library, clone:B230218J24 product:hypothetical protein, full insert sequence. | 2.661 |
| NAP048198-1 | Unknown | 2.659 |
| NM\_008760 | Mus musculus osteoglycin (Ogn), mRNA | 2.658 |
| AK050560 | Mus musculus adult pancreas islet cells cDNA, RIKEN full-length enriched library, clone:C820018D16 product:Nedd4 WW binding protein 5, full insert sequence. | 2.657 |
| NM\_026062 | Mus musculus RIKEN cDNA 2900024C23 gene (2900024C23Rik), mRNA | 2.655 |
| NM\_019498 | Mus musculus olfactomedin 1 (Olfm1), mRNA | 2.651 |
| NM\_016678 | Mus musculus reversion-inducing-cysteine-rich protein with kazal motifs (Reck), mRNA | 2.638 |
| NM\_177689 | Mus musculus hypothetical protein B930018B01 (B930018B01), mRNA | 2.637 |
| NM\_175383 | Mus musculus RIKEN cDNA 1500032M01 gene (1500032M01Rik), mRNA | 2.634 |
| NM\_152808 | Mus musculus RIKEN cDNA 1110028E10 gene (1110028E10Rik), mRNA | 2.634 |
| AK122282 | Mus musculus mRNA for mKIAA0438 protein. | 2.619 |
| AK038662 | Mus musculus adult male hypothalamus cDNA, RIKEN full-length enriched library, clone:A230053F13 product:unknown EST, full insert sequence | 2.618 |
| NM\_138313 | Mus musculus Bcl2 modifying factor (Bmf), mRNA | 2.615 |
| NM\_178218 | Mus musculus histone 3, H2a (Hist3h2a), mRNA | 2.612 |
| BC059025 | Mus musculus RIKEN cDNA 2810011L19 gene, mRNA (cDNA clone IMAGE:6810678), partial cds | 2.611 |
| NM\_173788 | Mus musculus natriuretic peptide receptor 2 (Npr2), mRNA | 2.609 |
| NM\_029565 | Mus musculus open reading frame 18 (ORF18), mRNA | 2.609 |
| XM\_146397 | Mus musculus similar to hypothetical protein (LOC211241), mRNA | 2.602 |
| NM\_015740 | Mus musculus biogenesis of lysosome-related organelles complex-1, subunit 1 (Bloc1s1), mRNA | 2.601 |
| AK033517 | Mus musculus adult male colon cDNA, RIKEN full-length enriched library, clone:9030417P20 product:ecotropic viral integration site 1, full insert sequence. | 2.595 |
| AK045686 | Mus musculus adult male corpora quadrigemina cDNA, RIKEN full-length enriched library, clone:B230304B05 product:interleukin 1 receptor accessory protein, full insert sequence. | 2.593 |
| NM\_010197 | Mus musculus fibroblast growth factor 1 (Fgf1), mRNA | 2.592 |
| NM\_178660 | Mus musculus RIKEN cDNA 6720477E09 gene (6720477E09Rik), mRNA | 2.589 |
| AK014035 | Mus musculus 13 days embryo head cDNA, RIKEN full-length enriched library, clone:3110009M16 product:hypothetical protein, full insert sequence. | 2.586 |
| BI152692 | 602918394F1 NCI\_CGAP\_Lu29 Mus musculus cDNA clone IMAGE:5068942 5', mRNA sequence | 2.585 |
| NM\_011716 | Mus musculus Wolfram syndrome 1 homolog (human) (Wfs1), mRNA | 2.584 |
| NM\_008415 | Mus musculus jerky (Jrk), mRNA | 2.583 |
| NM\_018827 | Mus musculus cytokine receptor-like factor 1 (Crlf1), mRNA | 2.583 |
| NM\_009721 | Mus musculus ATPase, Na+/K+ transporting, beta 1 polypeptide (Atp1b1), mRNA | 2.582 |
| NM\_053147 | Mus musculus protocadherin beta 22 (Pcdhb22), mRNA | 2.579 |
| NM\_009755 | Mus musculus bone morphogenetic protein 1 (Bmp1), mRNA | 2.578 |
| NM\_010395 | Mus musculus histocompatibility 2, T region locus 10 (H2-T10), mRNA | 2.576 |
| NM\_080555 | Mus musculus phosphatidic acid phosphatase type 2B (Ppap2b), mRNA | 2.574 |
| NM\_010859 | Mus musculus myosin, light polypeptide 3 (Myl3), mRNA | 2.573 |
| NM\_026514 | Mus musculus CDC42 effector protein (Rho GTPase binding) 3 (Cdc42ep3), mRNA | 2.569 |
| NM\_054041 | Mus musculus anthrax toxin receptor 1 (Antxr1), mRNA | 2.560 |
| NM\_007740 | Mus musculus procollagen, type IX, alpha 1 (Col9a1), mRNA | 2.560 |
| BC060120 | Mus musculus cDNA clone IMAGE:6412831, partial cds | 2.559 |
| NM\_007853 | Mus musculus degenerative spermatocyte homolog (Drosophila) (Degs), mRNA | 2.559 |
| NM\_008669 | Mus musculus N-acetyl galactosaminidase, alpha (Naga), mRNA | 2.558 |
| NM\_019634 | Mus musculus transmembrane 4 superfamily member 2 (Tm4sf2), mRNA | 2.557 |
| NM\_027870 | Mus musculus armadillo repeat containing, X-linked 3 (Armcx3), mRNA | 2.553 |
| AK052981 | Mus musculus 15 days embryo head cDNA, RIKEN full-length enriched library, clone:D930007L06 product:weakly similar to TUMOR SUPPRESSOR PHYDE [Rattus norvegicus], full insert sequence. | 2.550 |
| NM\_030263 | Mus musculus cDNA sequence BC00398 (BC003498), mRNA | 2.544 |
| NM\_177390 | Mus musculus RIKEN cDNA 9930104H07 gene (9930104H07Rik), mRNA | 2.543 |
| AK015966 | Mus musculus adult male testis cDNA, RIKEN full-length enriched library, clone:4930534K13 product:hypothetical protein, full insert sequence. | 2.543 |
| NM\_025378 | Mus musculus interferon induced transmembrane protein 3 (Ifitm3), mRNA | 2.538 |
| AK010169 | Mus musculus adult male tongue cDNA, RIKEN full-length enriched library, clone:2310075E07 product:unknown EST, full insert sequence. | 2.533 |
| NM\_025987 | Mus musculus NADH dehydrogenase (ubiquinone) 1 alpha subcomplex, 6 (B14) (Ndufa6), mRNA | 2.532 |
| A\_51\_P297993 | Unknown | 2.531 |
| NM\_007755 | Mus musculus cytoplasmic polyadenylation element binding protein 1 (Cpeb1), mRNA | 2.527 |
| NM\_027430 | Mus musculus RIKEN cDNA 2610205H19 gene (2610205H19Rik), mRNA | 2.525 |
| M89800 | Mouse Wnt-6 mRNA, complete cds. | 2.523 |
| NM\_144808 | Mus musculus solute carrier family 39 (zinc transporter), member 14 (Slc39a14), mRNA | 2.523 |
| NM\_133721 | Mus musculus integrin alpha 9 (Itga9), mRNA | 2.520 |
| NM\_008986 | Mus musculus polymerase I and transcript release factor (Ptrf), mRNA | 2.519 |
| AK089717 | Mus musculus activated spleen cDNA, RIKEN full-length enriched library, clone:F830010I22 product:weakly similar to CDNA FLJ10923 FIS, CLONE OVARC1000437, MODERATELY SIMILAR TO TENSIN [Homo sapiens], full insert sequence. | 2.517 |
| NM\_009371 | Mus musculus transforming growth factor, beta receptor II (Tgfbr2), transcript variant 1, mRNA | 2.517 |
| AY035899 | Mus musculus nephronectin long isoform (Neph1) mRNA, complete cds, alternatively spliced. | 2.517 |
| NM\_011125 | Mus musculus phospholipid transfer protein (Pltp), mRNA | 2.510 |
| NM\_019771 | Mus musculus destrin (Dstn), mRNA | 2.501 |
| NM\_011792 | Mus musculus beta-site APP cleaving enzyme 1 (Bace1), mRNA | 2.486 |
| NM\_026629 | Mus musculus RIKEN cDNA 2410066E13 gene (2410066E13Rik), mRNA | 2.486 |
| NM\_018738 | Mus musculus interferon gamma induced GTPase (Igtp), mRNA | 2.486 |
| AK080940 | Mus musculus 4 days neonate male adipose cDNA, RIKEN full-length enriched library, clone:B430214H24 product:nuclear factor I | 2.484 |
| NM\_145418 | Mus musculus cDNA sequence BC013529 (BC013529), mRNA | 2.483 |
| NM\_010163 | Mus musculus exostoses (multiple) 2 (Ext2), mRNA | 2.482 |
| NM\_007533 | Mus musculus branched chain ketoacid dehydrogenase E1, alpha polypeptide (Bckdha), mRNA | 2.482 |
| NM\_054071 | Mus musculus fibroblast growth factor receptor-like 1 (Fgfrl1), mRNA | 2.476 |
| NM\_008304 | Mus musculus syndecan 2 (Sdc2), mRNA | 2.476 |
| X69620 | M.musculus mRNA for inhibin beta-B subunit. | 2.475 |
| AK009829 | Mus musculus adult male tongue cDNA, RIKEN full-length enriched library, clone:2310045N01 product:hypothetical protein, full insert sequence. | 2.474 |
| NM\_011815 | Mus musculus FYN binding protein (Fyb), mRNA | 2.464 |
| BC060266 | Mus musculus dimethylarginine dimethylaminohydrolase 1, mRNA (cDNA clone IMAGE:5698949), complete cds. | 2.463 |
| M63554 | Mus musculus inhibitor protein of cAMP-dependent protein kinase, complete cds. | 2.452 |
| BC016223 | Mus musculus molybdenum cofactor sulfurase, mRNA (cDNA clone IMAGE:4486687), containing frame-shift errors | 2.450 |
| NM\_012032 | Mus musculus tumor differentially expressed 1 (Tde1), mRNA | 2.449 |
| AK088619 | Mus musculus 2 days neonate thymus thymic cells cDNA, RIKEN full-length enriched library, clone:E430021K16 product:hypothetical protein, full insert sequence | 2.448 |
| BC025091 | Mus musculus sterol O-acyltransferase 1, mRNA (cDNA clone IMAGE:4007277), partial cds. | 2.444 |
| NM\_008556 | Mus musculus phosphoprotein enriched in astrocytes 15 (Pea15), mRNA | 2.441 |
| NM\_010189 | Mus musculus Fc receptor, IgG, alpha chain transporter (Fcgrt), mRNA | 2.440 |
| NM\_008624 | Mus musculus muscle and microspikes RAS (Mras), mRNA | 2.439 |
| AK080672 | Mus musculus 10 days neonate cortex cDNA, RIKEN full-length enriched library, clone:A830062A20 product:gamma-aminobutyric acid (GABA-A) receptor, subunit beta 3, full insert sequence. | 2.437 |
| NM\_020573 | Mus musculus oxysterol binding protein-like 1A (Osbpl1a), mRNA | 2.437 |
| NM\_016919 | Mus musculus procollagen, type V, alpha 3 (Col5a3), mRNA | 2.437 |
| NM\_180962 | Mus musculus cysteine and histidine rich 1 (Cyhr1), mRNA | 2.437 |
| NM\_019691 | Mus musculus glutamate receptor, ionotropic, AMPA4 (alpha 4) (Gria4), mRNA | 2.435 |
| NM\_144859 | Mus musculus praja 2, RING-H2 motif containing (Pja2), mRNA | 2.431 |
| NM\_026388 | Mus musculus RIKEN cDNA 1200015A19 gene (1200015A19Rik), mRNA | 2.429 |
| NM\_153560 | Mus musculus RIKEN cDNA C230093N12 gene (C230093N12Rik), mRNA | 2.427 |
| NM\_008492 | Mus musculus lactate dehydrogenase 2, B chain (Ldh2), mRNA | 2.425 |
| NM\_017464 | Mus musculus neural precursor cell expressed, developmentally down-regulated gene 9 (Nedd9), mRNA | 2.422 |
| NM\_008609 | Mus musculus matrix metalloproteinase 15 (Mmp15), mRNA | 2.421 |
| NM\_027166 | Mus musculus yippee-like 5 (Drosophila) (Ypel5), mRNA | 2.419 |
| NM\_007598 | Mus musculus CAP, adenylate cyclase-associated protein 1 (yeast) (Cap1), mRNA | 2.418 |
| NM\_025735 | Mus musculus microtubule-associated protein 1 light chain 3 alpha (Map1lc3a), mRNA | 2.412 |
| NM\_182784 | Mus musculus DNA segment, Chr 6, ERATO Doi 349, expressed (D6Ertd349e), mRNA | 2.406 |
| NM\_020031 | Mus musculus prefoldin 5 (Pfdn5), mRNA | 2.403 |
| NM\_008615 | Mus musculus malic enzyme, supernatant (Mod1), mRNA | 2.399 |
| NM\_013931 | Mus musculus mitogen-activated protein kinase 8 interacting protein 3 (Mapk8ip3), mRNA | 2.396 |
| NM\_007804 | Mus musculus cut-like 2 (Drosophila) (Cutl2), mRNA | 2.396 |
| AK129301 | Mus musculus mRNA for mKIAA1162 protein. | 2.394 |
| AK076432 | Mus musculus 0 day neonate head cDNA, RIKEN full-length enriched library, clone:4832415J17 product:hypothetical Protein kinase-like (PK-like) structure containing protein, full insert sequence. | 2.392 |
| NM\_010516 | Mus musculus cysteine rich protein 61 (Cyr61), mRNA | 2.392 |
| ENSMUST00000049432 | I68673 gene X123 protein - human (fragment), partial (67%) [TC1042646] | 2.391 |
| NM\_133687 | Mus musculus CXXC finger 5 (Cxxc5), mRNA | 2.390 |
| BC055006 | Mus musculus cDNA clone MGC:57099 IMAGE:6491507, complete cds. | 2.388 |
| TC959587 | Unknown | 2.388 |
| NM\_029035 | Mus musculus RIKEN cDNA 4930422J18 gene (4930422J18Rik), mRNA | 2.386 |
| NM\_029322 | Mus musculus RIKEN cDNA 1700024B05 gene (1700024B05Rik), mRNA | 2.383 |
| NM\_175356 | Mus musculus phosphatidylinositol 4-kinase, catalytic, beta polypeptide (Pik4cb), mRNA | 2.383 |
| AF116911 | Mus musculus thymic dendritic cell-derived factor 1 mRNA, complete cds. | 2.383 |
| BC053842 | Mus musculus growth factor receptor bound protein 10, mRNA (cDNA clone IMAGE:6311825), partial cds. | 2.382 |
| NM\_134042 | Mus musculus aldehyde dehydrogenase family 6, subfamily A1 (Aldh6a1), mRNA | 2.379 |
| BC020023 | Mus musculus glutaminyl-peptide cyclotransferase (glutaminyl cyclase), mRNA (cDNA clone MGC:27858 IMAGE:3491756), complete cds. | 2.379 |
| NM\_025932 | Mus musculus synapse associated protein 1 (Syap1), mRNA | 2.370 |
| AK129382 | Mus musculus mRNA for mKIAA1522 protein. | 2.370 |
| NM\_021881 | Mus musculus quaking (Qk), mRNA | 2.365 |
| NM\_025926 | Mus musculus DnaJ (Hsp40) homolog, subfamily B, member 4 (Dnajb4), mRNA | 2.364 |
| BC048004 | Mus musculus formin-like 2, mRNA (cDNA clone IMAGE:5033815), partial cds. | 2.363 |
| NM\_009242 | Mus musculus secreted acidic cysteine rich glycoprotein (Sparc), mRNA | 2.362 |
| NM\_024207 | Mus musculus RIKEN cDNA 1110021N07 gene (1110021N07Rik), mRNA | 2.361 |
| NM\_013565 | Mus musculus integrin alpha 3 (Itga3), mRNA | 2.360 |
| XM\_127961 | Mus musculus RIKEN cDNA 9930116P15 gene (9930116P15Rik), mRNA | 2.358 |
| NM\_026790 | Mus musculus DNA segment, Chr 12, ERATO Doi 647, expressed (D12Ertd647e), transcript variant 1, mRNA | 2.351 |
| NM\_172961 | Mus musculus 4-aminobutyrate aminotransferase (Abat), mRNA | 2.350 |
| NM\_053146 | Mus musculus protocadherin beta 21 (Pcdhb21), mRNA | 2.350 |
| NM\_172498 | Mus musculus PTK2 protein tyrosine kinase 2 beta (Ptk2b), mRNA | 2.349 |
| NM\_018798 | Mus musculus ubiquilin 2 (Ubqln2), mRNA | 2.349 |
| NM\_026556 | Mus musculus RIKEN cDNA 6720463E02 gene (6720463E02Rik), mRNA | 2.348 |
| NM\_009976 | Mus musculus cystatin C (Cst3), mRNA | 2.345 |
| NM\_025440 | Mus musculus mitochondrial ribosomal protein S16 (Mrps16), mRNA | 2.344 |
| NM\_145925 | Mus musculus pituitary tumor-transforming 1 interacting protein (Pttg1ip), mRNA | 2.343 |
| NM\_008788 | Mus musculus procollagen C-proteinase enhancer protein (Pcolce), mRNA | 2.343 |
| NM\_175108 | Mus musculus RIKEN cDNA 2310047C17 gene (2310047C17Rik), mRNA | 2.336 |
| NM\_011218 | Mus musculus protein tyrosine phosphatase, receptor type, S (Ptprs), mRNA | 2.334 |
| AY033771 | Mus musculus type II cAMP-dependent protein kinase anchoring protein Ht31 mRNA, partial cds. | 2.333 |
| NM\_029669 | Mus musculus RIKEN cDNA 2700075B01 gene (2700075B01Rik), mRNA | 2.333 |
| NM\_177583 | Mus musculus hypothetical protein 4632417K02 (4632417K02), mRNA | 2.332 |
| NM\_007705 | Mus musculus cold inducible RNA binding protein (Cirbp), mRNA | 2.327 |
| AK020134 | Mus musculus 12 days embryo male wolffian duct includes surrounding region cDNA, RIKEN full-length enriched library, clone:6720458D04 product:receptor (calcitonin) activity modifying protein 2, full insert sequence. | 2.323 |
| BC051045 | Mus musculus cDNA clone MGC:59118 IMAGE:4505411, complete cds. | 2.323 |
| AK030696 | Mus musculus 10 days neonate head cDNA, RIKEN full-length enriched library, clone:5530400J02 product:similar to DJ971N18.2.1 (NOVEL PROTEIN (ISOFORM 1)) [Homo sapiens], full insert sequence. | 2.321 |
| NM\_026418 | Mus musculus regulator of G-protein signalling 10 (Rgs10), mRNA | 2.316 |
| AB011255 | Mus musculus mRNA for BH-Pcdh-c, partial cds. | 2.315 |
| NM\_134189 | Mus musculus UDP-N-acetyl-alpha-D-galactosamine:polypeptide N-acetylgalactosaminyltransferase 10 (Galnt10), mRNA | 2.314 |
| NM\_009655 | Mus musculus activated leukocyte cell adhesion molecule (Alcam), mRNA | 2.313 |
| NM\_026908 | Mus musculus RIKEN cDNA 1500031K13 gene (1500031K13Rik), mRNA | 2.311 |
| NM\_177776 | Mus musculus RIKEN cDNA D130058I21 gene (D130058I21Rik), mRNA | 2.310 |
| NM\_009701 | Mus musculus aquaporin 5 (Aqp5), mRNA | 2.310 |
| BC019134 | Mus musculus, clone IMAGE:5037334, mRNA, partial cds. | 2.309 |
| NAP014312-001 | Unknown | 2.308 |
| NM\_145835 | Mus musculus lactase-like (Lctl), mRNA | 2.307 |
| NM\_019410 | Mus musculus profilin 2 (Pfn2), mRNA | 2.306 |
| NM\_013750 | Mus musculus pleckstrin homology-like domain, family A, member 3 (Phlda3), mRNA | 2.304 |
| NM\_009334 | Mus musculus transcription factor AP-2 beta (Tcfap2b), mRNA | 2.304 |
| AK017654 | Mus musculus 8 days embryo whole body cDNA, RIKEN full-length enriched library, clone:5730450N06 product:unknown EST, full insert sequence. | 2.302 |
| NM\_053145 | Mus musculus protocadherin beta 20 (Pcdhb20), mRNA | 2.300 |
| NM\_021474 | Mus musculus epidermal growth factor-containing fibulin-like extracellular matrix protein 2 (Efemp2), mRNA | 2.300 |
| NM\_026316 | Mus musculus aldehyde dehydrogenase 3 family, member B1 (Aldh3b1), mRNA | 2.295 |
| BC020182 | Mus musculus RIKEN cDNA 2700082O15 gene, mRNA (cDNA clone IMAGE:4501462), containing frame-shift errors. | 2.290 |
| AK031010 | Mus musculus adult male thymus cDNA, RIKEN full-length enriched library, clone:5830488H16 product:unknown EST, full insert sequence. | 2.289 |
| NM\_009187 | Mus musculus cytochrome c oxidase subunit VIIa polypeptide 2-like (Cox7a2l), mRNA | 2.287 |
| BC059832 | Mus musculus activin A receptor, type 1B, mRNA (cDNA clone IMAGE:6844539), partial cds | 2.285 |
| BC029027 | Mus musculus calsyntenin 1, mRNA (cDNA clone IMAGE:5358946), partial cds. | 2.285 |
| NM\_010415 | Mus musculus diphtheria toxin receptor (Dtr), mRNA | 2.284 |
| NM\_007584 | Mus musculus discoidin domain receptor family, member 1 (Ddr1), mRNA | 2.284 |
| BI646741 | BI646741 603279769F1 NCI\_CGAP\_Mam3 Mus musculus cDNA clone IMAGE:5320025 5', mRNA sequence | 2.282 |
| TC1059245 | Unknown | 2.279 |
| XM\_129785 | Mus musculus hypothetical protein C230017K02 (C230017K02), mRNA | 2.279 |
| XM\_111887 | Mus musculus similar to B-cell translocation gene 1; B-cell translocation gene 1, anti-proliferative (LOC194735), mRNA | 2.277 |
| NM\_020557 | Mus musculus thymidylate kinase family LPS-inducible member (Tyki), mRNA | 2.277 |
| BC049662 | Mus musculus membrane protein, palmitoylated 7 (MAGUK p55 subfamily member 7), mRNA (cDNA clone IMAGE:6532780), partial cds. | 2.277 |
| NM\_133922 | Mus musculus RIKEN cDNA A930040G15 gene (A930040G15Rik), mRNA | 2.275 |
| NM\_010952 | Mus musculus ornithine decarboxylase antizyme 2 (Oaz2), mRNA | 2.274 |
| AF220209 | Mus musculus Nedd4 WW domain-binding protein 5 mRNA, partial cds. | 2.274 |
| BC023928 | Mus musculus cDNA sequence BC023928, mRNA (cDNA clone IMAGE:5324278), partial cds. | 2.271 |
| NM\_011349 | Mus musculus sema domain, immunoglobulin domain (Ig), short basic domain, secreted, (semaphorin) 3 F (Sema3f), mRNA | 2.271 |
| NM\_145434 | Mus musculus nuclear receptor subfamily 1, group D, member 1 (Nr1d1), mRNA | 2.270 |
| NM\_173777 | Mus musculus RIKEN cDNA A030009A06 gene (A030009A06Rik), mRNA | 2.270 |
| NM\_007725 | Mus musculus calponin 2 (Cnn2), mRNA | 2.268 |
| NM\_010500 | Mus musculus immediate early response 5 (Ier5), mRNA | 2.268 |
| NM\_001001309 | Mus musculus integrin alpha 8 (Itga8), mRNA | 2.268 |
| NM\_146102 | Mus musculus expressed sequence AU041783 (AU041783), mRNA | 2.267 |
| NM\_080638 | Mus musculus major vault protein (Mvp), mRNA | 2.267 |
| NM\_146008 | Mus musculus RIKEN cDNA E430026E19 gene (E430026E19Rik), mRNA | 2.265 |
| AK007199 | Mus musculus adult male testis cDNA, RIKEN full-length enriched library, clone:1700113K09 product:unclassifiable, full insert sequence. | 2.265 |
| NM\_008813 | Mus musculus ectonucleotide pyrophosphatase/phosphodiesterase 1 (Enpp1), mRNA | 2.262 |
| NM\_023638 | Mus musculus porcupine homolog (Drosophila) (Porcn), transcript variant Mporc-d, mRNA | 2.257 |
| ENSMUST00000037357 | Unknown | 2.256 |
| BC059256 | Mus musculus cDNA clone MGC:67592 IMAGE:6406444, complete cds. | 2.255 |
| NM\_153573 | Mus musculus cDNA sequence BC029109 (BC029109), mRNA | 2.254 |
| NM\_172282 | Mus musculus RIKEN cDNA B230339H12 gene (B230339H12Rik), mRNA | 2.252 |
| NM\_030689 | Mus musculus neuronal pentraxin receptor (Nptxr), mRNA | 2.252 |
| AF061283 | Mus musculus neuronal protein 4.1 mRNA, complete cds. | 2.251 |
| NM\_176848 | Mus musculus F-box only protein 2 (Fbxo2), mRNA | 2.250 |
| D14636 | Mouse mRNA for PEBP2a1 protein, complete cds. | 2.248 |
| AK076221 | Mus musculus 15 days embryo head cDNA, RIKEN full-length enriched library, clone:4022442N15 product:similar to KIAA0940 PROTEIN [Homo sapiens], full insert sequence | 2.248 |
| NM\_007603 | Mus musculus calpain 6 (Capn6), mRNA | 2.248 |
| NM\_134076 | Mus musculus abhydrolase domain containing 4 (Abhd4), mRNA | 2.247 |
| XM\_357279 | Mus musculus similar to myelin P2 protein - mouse (LOC383825), mRNA | 2.245 |
| NM\_019517 | Mus musculus beta-site APP-cleaving enzyme 2 (Bace2), mRNA | 2.245 |
| BE447944 | ut39e06.y1 Soares mouse 3NbMS Mus musculus cDNA clone IMAGE:3330274 5'. | 2.241 |
| NM\_198429 | Mus musculus nuclear factor of activated T-cells, cytoplasmic, calcineurin-dependent 1 (Nfatc1), mRNA | 2.240 |
| NM\_019765 | Mus musculus restin (Reed-Steinberg cell-expressed intermediate filament-associated protein) (Rsn), mRNA | 2.240 |
| NM\_028763 | Mus musculus neuronal pentraxin receptor (Nptxr), mRNA | 2.239 |
| NM\_198293 | Mus musculus RIKEN cDNA 4921518A06 gene (4921518A06Rik), mRNA | 2.239 |
| NM\_021424 | Mus musculus poliovirus receptor-related 1 (Pvrl1), mRNA | 2.238 |
| NM\_027044 | Mus musculus RIKEN cDNA 1700010A06 gene (1700010A06Rik), mRNA | 2.234 |
| AK053910 | Mus musculus 0 day neonate eyeball cDNA, RIKEN full-length enriched library, clone:E130320J01 product:runt related transcription factor 3, full insert sequence. | 2.233 |
| XM\_148062 | Mus musculus RIKEN cDNA 2310015A05 gene (2310015A05Rik), mRNA | 2.230 |
| NM\_031199 | Mus musculus transforming growth factor alpha (Tgfa), mRNA | 2.230 |
| NM\_145512 | Mus musculus RIKEN cDNA 2010005O13 gene (2010005O13Rik), mRNA | 2.229 |
| NM\_023608 | Mus musculus glycerophosphodiester phosphodiesterase domain containing 2 (Gdpd2), mRNA | 2.226 |
| AK030884 | Mus musculus adult male thymus cDNA, RIKEN full-length enriched library, clone:5830443C17 product:unknown EST, full insert sequence. | 2.226 |
| NM\_198607 | Mus musculus RIKEN cDNA 4930572J05 gene (4930572J05Rik), mRNA | 2.224 |
| NM\_018884 | Mus musculus PDZ domain containing RING finger 3 (Pdzrn3), mRNA | 2.222 |
| AK052241 | Mus musculus 13 days embryo heart cDNA, RIKEN full-length enriched library, clone:D330012G24 product:insulin receptor substrate 1, full insert sequence. | 2.221 |
| BC048835 | Mus musculus filamin, beta, mRNA (cDNA clone IMAGE:4913636), partial cds. | 2.220 |
| AK050387 | Mus musculus adult male liver tumor cDNA, RIKEN full-length enriched library, clone:C730042J18 product:hypothetical protein, full insert sequence. | 2.219 |
| AK002271 | Mus musculus adult male kidney cDNA, RIKEN full-length enriched library, clone:0610007B22 product:tropomyosin 2, beta, full insert sequence. | 2.218 |
| NM\_177411 | Mus musculus RAB5B, member RAS oncogene family (Rab5b), mRNA | 2.215 |
| NM\_013626 | Mus musculus peptidylglycine alpha-amidating monooxygenase (Pam), mRNA | 2.210 |
| NM\_019793 | Mus musculus transmembrane 4 superfamily member 8 (Tm4sf8), mRNA | 2.210 |
| NM\_020498 | Mus musculus lymphocyte antigen 6 complex, locus I (Ly6i), mRNA | 2.210 |
| AK082964 | Mus musculus 12 days embryo spinal cord cDNA, RIKEN full-length enriched library, clone:C530025K05 product:unknown EST, full insert sequence | 2.209 |
| NM\_133987 | Mus musculus solute carrier family 6 (neurotransmitter transporter, creatine), member 8 (Slc6a8), mRNA | 2.208 |
| XM\_354846 | Mus musculus similar to RIKEN cDNA 4930503E14 (LOC380938), mRNA | 2.207 |
| NAP104671-1 | Unknown | 2.207 |
| NM\_009842 | Mus musculus CD151 antigen (Cd151), mRNA | 2.207 |
| NM\_025326 | Mus musculus RIKEN cDNA 0610011I04 gene (0610011I04Rik), mRNA | 2.205 |
| NM\_144899 | Mus musculus thrombospondin repeat containing 1 (Tsrc1), mRNA | 2.203 |
| NM\_008027 | Mus musculus flotillin 1 (Flot1), mRNA | 2.197 |
| AK048603 | Mus musculus 16 days embryo head cDNA, RIKEN full-length enriched library, clone:C130085G02 product:hypothetical Serine-rich region/Tyrosine specific protein phosphatase and dual specificity protein phosphatase family/Dual specificity protein phosphatas | 2.197 |
| NM\_009636 | Mus musculus AE binding protein 1 (Aebp1), mRNA | 2.196 |
| AK129463 | Mus musculus mRNA for mKIAA1888 protein. | 2.196 |
| NM\_145980 | Mus musculus RIKEN cDNA 8430408G22 gene (8430408G22Rik), mRNA | 2.196 |
| NM\_010687 | Mus musculus like-glycosyltransferase (Large), mRNA | 2.195 |
| XM\_125904 | Mus musculus SLIT-ROBO Rho GTPase activating protein 1 (Srgap1), mRNA | 2.195 |
| AK081887 | Mus musculus 16 days embryo head cDNA, RIKEN full-length enriched library, clone:C130084O20 product:selected mouse cDNA on the X, full insert sequence. | 2.195 |
| AK122490 | Mus musculus mRNA for mKIAA1302 protein. | 2.194 |
| NM\_133350 | Mus musculus microtubule-associated protein, RP/EB family, member 3 (Mapre3), mRNA | 2.194 |
| NM\_028127 | Mus musculus RIKEN cDNA 4930488L10 gene (4930488L10Rik), mRNA | 2.193 |
| AK013788 | Mus musculus adult male hippocampus cDNA, RIKEN full-length enriched library, clone:2900075A18 product:CAM-KII INHIBITORY PROTEIN homolog [Homo sapiens], full insert sequence. | 2.193 |
| NM\_030261 | Mus musculus sestrin 3 (Sesn3), mRNA | 2.191 |
| NM\_007899 | Mus musculus extracellular matrix protein 1 (Ecm1), mRNA | 2.190 |
| NM\_011046 | Mus musculus furin (paired basic amino acid cleaving enzyme) (Furin), mRNA | 2.189 |
| AK003667 | Mus musculus 18-day embryo whole body cDNA, RIKEN full-length enriched library, clone:1110013L07 product:inferred: COTE1 PROTEIN. [Human] {Homo sapiens}, full insert sequence. | 2.188 |
| AK021106 | Mus musculus adult male corpus striatum cDNA, RIKEN full-length enriched library, clone:C030027H14 product:unknown EST, full insert sequence. | 2.188 |
| NM\_212446 | Mus musculus proteasome (prosome, macropain) inhibitor subunit 1 (Psmf1), mRNA | 2.185 |
| AK129084 | Mus musculus mRNA for mKIAA0193 protein. | 2.185 |
| NM\_025473 | Mus musculus RIKEN cDNA 1810037C20 gene (1810037C20Rik), mRNA | 2.185 |
| AK090034 | Mus musculus female sarcoma RCB-0464 Meth-A cDNA, RIKEN full-length enriched library, clone:G430067O17 product:hypothetical protein, full insert sequence. | 2.185 |
| AK030860 | Mus musculus adult male thymus cDNA, RIKEN full-length enriched library, clone:5830432P16 product:unknown EST, full insert sequence | 2.184 |
| NM\_029519 | Mus musculus RIKEN cDNA 5830461H18 gene (5830461H18Rik), mRNA | 2.184 |
| NM\_025432 | Mus musculus trafficking protein particle complex 2 (Trappc2), mRNA | 2.182 |
| TC1069351 | U43585 protein kinase related to Raf protein kinases; Method: conceptual translation supplied by author {Mus musculus}, complete | 2.180 |
| NM\_008010 | Mus musculus fibroblast growth factor receptor 3 (Fgfr3), mRNA | 2.178 |
| NM\_028643 | Mus musculus RIKEN cDNA 1110008L20 gene (1110008L20Rik), mRNA | 2.177 |
| NM\_009982 | Mus musculus cathepsin C (Ctsc), mRNA | 2.175 |
| BC028320 | Mus musculus RIKEN cDNA 4732418C07 gene, mRNA (cDNA clone IMAGE:4037144), partial cds. | 2.174 |
| NM\_021389 | Mus musculus SH3-domain kinase binding protein 1 (Sh3kbp1), mRNA | 2.172 |
| NAP042901-1 | Unknown | 2.172 |
| NM\_133189 | Mus musculus calcium channel, voltage-dependent, gamma subunit 7 (Cacng7), mRNA | 2.171 |
| NM\_008985 | Mus musculus protein tyrosine phosphatase, receptor type, N (Ptprn), mRNA | 2.163 |
| AK122468 | Mus musculus mRNA for mKIAA1209 protein. | 2.162 |
| NM\_008640 | Mus musculus lysosomal-associated protein transmembrane 4A (Laptm4a), mRNA | 2.161 |
| AK009939 | Mus musculus adult male tongue cDNA, RIKEN full-length enriched library, clone:2310056B04 product:unknown EST, full insert sequence. | 2.160 |
| NM\_021412 | Mus musculus matrix metalloproteinase 19 (Mmp19), mRNA | 2.158 |
| NM\_007680 | Mus musculus Eph receptor B6 (Ephb6), mRNA | 2.158 |
| NM\_024406 | Mus musculus fatty acid binding protein 4, adipocyte (Fabp4), mRNA | 2.158 |
| NM\_198860 | Mus musculus expressed sequence AI646023 (AI646023), mRNA | 2.157 |
| TC1050071 | Unknown | 2.156 |
| NM\_025549 | Mus musculus arrestin domain containing 4 (Arrdc4), mRNA | 2.156 |
| NM\_053117 | Mus musculus par-6 partitioning defective 6 homolog gamma (C. elegans) (Pard6g), mRNA | 2.156 |
| NM\_133950 | Mus musculus KDEL (Lys-Asp-Glu-Leu) endoplasmic reticulum protein retention receptor 1 (Kdelr1), mRNA | 2.156 |
| BC058110 | Mus musculus RIKEN cDNA A730011F23 gene, mRNA (cDNA clone IMAGE:6835298), partial cds. | 2.153 |
| NM\_172698 | Mus musculus RIKEN cDNA 4732418C07 gene (4732418C07Rik), mRNA | 2.152 |
| NM\_146120 | Mus musculus gelsolin (Gsn), mRNA | 2.149 |
| NM\_010656 | Mus musculus sarcospan (Sspn), mRNA | 2.148 |
| NM\_153078 | Mus musculus DNA sequence AF424697 (AF424697), mRNA | 2.148 |
| BC059938 | Mus musculus cDNA clone MGC:65558 IMAGE:6485174, complete cds. | 2.148 |
| NM\_008235 | Mus musculus hairy and enhancer of split 1 (Drosophila) (Hes1), mRNA | 2.147 |
| XM\_147036 | Mus musculus RIKEN cDNA 1190002N15 gene (1190002N15Rik), mRNA | 2.147 |
| NM\_021889 | Mus musculus synaptotagmin 9 (Syt9), mRNA | 2.146 |
| NM\_024219 | Mus musculus heat shock factor binding protein 1 (Hsbp1), mRNA | 2.146 |
| NM\_009343 | Mus musculus PHD finger protein 1 (Phf1), mRNA | 2.145 |
| NM\_173426 | Mus musculus RIKEN cDNA 1700012H17 gene (1700012H17Rik), mRNA | 2.143 |
| NM\_009674 | Mus musculus annexin A7 (Anxa7), mRNA | 2.143 |
| NM\_145429 | Mus musculus arrestin, beta 2 (Arrb2), mRNA | 2.142 |
| BC055753 | Mus musculus Sestrin 1, mRNA (cDNA clone MGC:67135 IMAGE:6414521), complete cds. | 2.141 |
| NM\_001001980 | Mus musculus RIKEN cDNA 3732412D22 gene (3732412D22Rik), mRNA | 2.136 |
| M29546 | Mouse MOD-1 null malic enzyme mRNA, partial cds. | 2.133 |
| AK035049 | Mus musculus 12 days embryo embryonic body between diaphragm region and neck cDNA, RIKEN full-length enriched library, clone:9430079B08 product:unknown EST, full insert sequence | 2.132 |
| NM\_144919 | Mus musculus histone deacetylase 11 (Hdac11), mRNA | 2.130 |
| NM\_008543 | Mus musculus MAD homolog 7 (Drosophila) (Smad7), mRNA | 2.129 |
| NM\_010284 | Mus musculus growth hormone receptor (Ghr), mRNA | 2.125 |
| NM\_198095 | Mus musculus RIKEN cDNA 2310015I10 gene (2310015I10Rik), mRNA | 2.121 |
| NM\_175145 | Mus musculus RIKEN cDNA 2310003P10 gene (2310003P10Rik), mRNA | 2.121 |
| NM\_018804 | Mus musculus synaptotagmin 11 (Syt11), mRNA | 2.119 |
| NM\_026821 | Mus musculus DNA segment, Chr 4, Brigham & Women's Genetics 0951 expressed (D4Bwg0951e), mRNA | 2.118 |
| NM\_175937 | Mus musculus cytoplasmic polyadenylation element binding protein 2 (Cpeb2), mRNA | 2.117 |
| AK016572 | Mus musculus adult male testis cDNA, RIKEN full-length enriched library, clone:4932443D16 product:hypothetical PA-phosphatase related phosphoesterase containing protein, full insert sequence. | 2.117 |
| BC053741 | Mus musculus SH3-domain binding protein 5 (BTK-associated), mRNA (cDNA clone MGC:59324 IMAGE:6336511), complete cds. | 2.114 |
| NM\_194342 | Mus musculus expressed sequence AI551766 (AI551766), mRNA | 2.112 |
| NM\_013587 | Mus musculus low density lipoprotein receptor-related protein associated protein 1 (Lrpap1), mRNA | 2.108 |
| NM\_007399 | Mus musculus a disintegrin and metalloprotease domain 10 (Adam10), mRNA | 2.105 |
| NM\_029870 | Mus musculus RIKEN cDNA A930001N09 gene (A930001N09Rik), mRNA | 2.105 |
| AA204140 | AA204140 mu57h01.r1 Soares mouse lymph node NbMLN Mus musculus cDNA clone IMAGE:643537 5' similar to gb:Z25524 M.musculus integrin associated protein mRNA, complete (MOUSE);, mRNA sequence | 2.105 |
| NM\_198602 | Mus musculus cut-like 1 (Drosophila) (Cutl1), transcript variant 2, mRNA | 2.103 |
| NM\_175266 | Mus musculus EPM2A (laforin) interacting protein 1 (Epm2aip1), mRNA | 2.103 |
| NM\_146145 | Mus musculus Janus kinase 1 (Jak1), mRNA | 2.103 |
| NM\_028375 | Mus musculus RIKEN cDNA 2900027G03 gene (2900027G03Rik), mRNA | 2.102 |
| NM\_178926 | Mus musculus expressed sequence AI662250 (AI662250), mRNA | 2.102 |
| NM\_172875 | Mus musculus RIKEN cDNA 4933429I20 gene (4933429I20Rik), mRNA | 2.101 |
| AK083715 | Mus musculus 9 days embryo whole body cDNA, RIKEN full-length enriched library, clone:D030070D03 product:unknown EST, full insert sequence. | 2.101 |
| NM\_007670 | Mus musculus cyclin-dependent kinase inhibitor 2B (p15, inhibits CDK4) (Cdkn2b), mRNA | 2.100 |
| NM\_177298 | Mus musculus RIKEN cDNA 9030221M09 gene (9030221M09Rik), mRNA | 2.099 |
| NM\_053078 | Mus musculus DNA segment, human D4S114 (D0H4S114), mRNA | 2.096 |
| AK088710 | Mus musculus 2 days neonate thymus thymic cells cDNA, RIKEN full-length enriched library, clone:E430024D02 product:LAR-INTERACTING PROTEIN 1B homolog [Homo sapiens], full insert sequence. | 2.095 |
| AK029831 | Mus musculus adult male testis cDNA, RIKEN full-length enriched library, clone:4931406B08 product:unknown EST, full insert sequence | 2.095 |
| NM\_013472 | Mus musculus annexin A6 (Anxa6), mRNA | 2.095 |
| NM\_010320 | Mus musculus guanine nucleotide binding protein (G protein), gamma 8 subunit (Gng8), mRNA | 2.095 |
| NM\_011341 | Mus musculus stromal cell derived factor 4 (Sdf4), mRNA | 2.094 |
| AK049924 | Mus musculus adult male hippocampus cDNA, RIKEN full-length enriched library, clone:C630011I23 product:hypothetical protein, full insert sequence. | 2.092 |
| NM\_133969 | Mus musculus cytochrome P450, family 4, subfamily v, polypeptide 3 (Cyp4v3), mRNA | 2.090 |
| AK079560 | Mus musculus adult male hypothalamus cDNA, RIKEN full-length enriched library, clone:A230074B03 product:TSPAN-2 PROTEIN homolog [Rattus norvegicus], full insert sequence | 2.089 |
| AK122438 | Mus musculus mRNA for mKIAA1086 protein | 2.088 |
| NM\_144549 | Mus musculus tribbles homolog 1 (Drosophila) (Trib1), mRNA | 2.087 |
| NM\_013912 | Mus musculus apelin (Apln), mRNA | 2.081 |
| NM\_010087 | Mus musculus dystrobrevin alpha (Dtna), transcript variant 2, mRNA | 2.080 |
| NM\_025912 | Mus musculus RIKEN cDNA 2010011I20 gene (2010011I20Rik), mRNA | 2.079 |
| NM\_019769 | Mus musculus RIKEN cDNA 1500003O03 gene (1500003O03Rik), mRNA | 2.079 |
| NM\_172767 | Mus musculus loss of heterozygosity, 11, chromosomal region 2, gene A homolog (human) (Loh11cr2a), mRNA | 2.079 |
| NM\_026420 | Mus musculus polyadenylate-binding protein-interacting protein 2 (Paip2), mRNA | 2.079 |
| NM\_172442 | Mus musculus deltex 4 homolog (Drosophila) (Dtx4), mRNA | 2.079 |
| NM\_177595 | Mus musculus RIKEN cDNA 9430023B20 gene (9430023B20Rik), mRNA | 2.078 |
| BC003348 | Mus musculus cDNA clone IMAGE:3481673, partial cds. | 2.077 |
| NM\_030693 | Mus musculus activating transcription factor 5 (Atf5), mRNA | 2.076 |
| NM\_028802 | Mus musculus RIKEN cDNA 2310032D16 gene (2310032D16Rik), mRNA | 2.075 |
| NM\_145550 | Mus musculus RIKEN cDNA C030002N13 gene (C030002N13Rik), mRNA | 2.075 |
| NM\_011863 | Mus musculus 3'-phosphoadenosine 5'-phosphosulfate synthase 1 (Papss1), mRNA | 2.075 |
| NM\_008808 | Mus musculus platelet derived growth factor, alpha (Pdgfa), mRNA | 2.071 |
| NM\_008160 | Mus musculus glutathione peroxidase 1 (Gpx1), mRNA | 2.068 |
| NM\_026192 | Mus musculus RIKEN cDNA 1810009B06 gene (1810009B06Rik), mRNA | 2.068 |
| NM\_153198 | Mus musculus high mobility group box transcription factor 1 (Hbp1), mRNA | 2.067 |
| NM\_178060 | Mus musculus thyroid hormone receptor alpha (Thra), mRNA | 2.067 |
| NM\_013867 | Mus musculus breast cancer anti-estrogen resistance 3 (Bcar3), mRNA | 2.066 |
| NM\_139295 | Mus musculus multiple coagulation factor deficiency 2 (Mcfd2), mRNA | 2.066 |
| BC003951 | Mus musculus insulin-like growth factor binding protein 5, mRNA (cDNA clone IMAGE:3487482), partial cds. | 2.066 |
| AK018172 | Mus musculus adult male medulla oblongata cDNA, RIKEN full-length enriched library, clone:6330414G02 product:unknown EST, full insert sequence | 2.065 |
| NM\_023738 | Mus musculus ubiquitin-activating enzyme E1-like (Ube1l), mRNA | 2.064 |
| NM\_177725 | Mus musculus leucine-rich repeat-containing 8 (Lrrc8), mRNA | 2.063 |
| NM\_031184 | Mus musculus GLIS family zinc finger 2 (Glis2), mRNA | 2.062 |
| NM\_019764 | Mus musculus angiomotin like 2 (Amotl2), mRNA | 2.061 |
| NM\_027154 | Mus musculus RIKEN cDNA 2310061B02 gene (2310061B02Rik), mRNA | 2.061 |
| NM\_019979 | Mus musculus selenoprotein K (Selk), mRNA | 2.060 |
| NM\_133655 | Mus musculus CD 81 antigen (Cd81), mRNA | 2.060 |
| TC982356 | Unknown | 2.059 |
| NM\_011175 | Mus musculus legumain (Lgmn), mRNA | 2.059 |
| NM\_008727 | Mus musculus natriuretic peptide receptor 1 (Npr1), mRNA | 2.058 |
| NM\_026321 | Mus musculus RIKEN cDNA 2310044D20 gene (2310044D20Rik), mRNA | 2.056 |
| NM\_030694 | Mus musculus interferon induced transmembrane protein 2 (Ifitm2), mRNA | 2.055 |
| NM\_007467 | Mus musculus amyloid beta (A4) precursor-like protein 1 (Aplp1), mRNA | 2.054 |
| NM\_177344 | Mus musculus RIKEN cDNA C730025P13 gene (C730025P13Rik), mRNA | 2.053 |
| NM\_133937 | Mus musculus RIKEN cDNA 6720456B07 gene (6720456B07Rik), mRNA | 2.052 |
| NM\_177662 | Mus musculus cathepsin O (Ctso), mRNA | 2.050 |
| AK078889 | Mus musculus adult male colon cDNA, RIKEN full-length enriched library, clone:9030621G03 product:unknown EST, full insert sequence | 2.049 |
| NM\_182997 | Mus musculus protein kinase, AMP-activated, beta 2 non-catalytic subunit (Prkab2), mRNA | 2.049 |
| NM\_183285 | Mus musculus potassium channel tetramerisation domain containing 2 (Kctd2), mRNA | 2.047 |
| NM\_145533 | Mus musculus spermine oxidase (Smox), mRNA | 2.047 |
| XM\_138240 | Mus musculus similar to hypothetical protein A030003A19 (LOC238395), mRNA | 2.044 |
| NM\_008963 | Mus musculus prostaglandin D2 synthase (brain) (Ptgds), mRNA | 2.042 |
| AK041412 | Mus musculus 3 days neonate thymus cDNA, RIKEN full-length enriched library, clone:A630007N06 product:hypothetical RING finger, Protease associated (PA) domain containing protein, full insert sequence. | 2.042 |
| NM\_021299 | Mus musculus adenylate kinase 3 alpha-like (Ak3l), mRNA | 2.041 |
| NM\_026142 | Mus musculus RIKEN cDNA 3632451O06 gene (3632451O06Rik), mRNA | 2.039 |
| AK019067 | Mus musculus adult male stomach cDNA, RIKEN full-length enriched library, clone:2210415I11 product:unclassifiable, full insert sequence. | 2.037 |
| NM\_019677 | Mus musculus phospholipase C, beta 1 (Plcb1), mRNA | 2.032 |
| NM\_016791 | Mus musculus nuclear factor of activated T-cells, cytoplasmic, calcineurin-dependent 1 (Nfatc1), mRNA | 2.032 |
| NM\_008452 | Mus musculus Kruppel-like factor 2 (lung) (Klf2), mRNA | 2.031 |
| AK033397 | Mus musculus 16 days embryo lung cDNA, RIKEN full-length enriched library, clone:8430429I17 product:unclassifiable, full insert sequence | 2.029 |
| NM\_175260 | Mus musculus myosin heavy chain 10, non-muscle (Myh10), mRNA | 2.028 |
| AK009137 | Mus musculus adult male tongue cDNA, RIKEN full-length enriched library, clone:2310004G06 product:hypothetical Glycerophosphoryl diester phosphodiesterase/Glycosyl hydrolase, starch-binding domain containing protein, full insert sequence. | 2.027 |
| AK035814 | Mus musculus 16 days neonate cerebellum cDNA, RIKEN full-length enriched library, clone:9630007E23 product:unclassifiable, full insert sequence. | 2.027 |
| NM\_133697 | Mus musculus RIKEN cDNA 1110003E01 gene (1110003E01Rik), mRNA | 2.026 |
| NM\_177708 | Mus musculus reticulon 4 receptor-like 1 (Rtn4rl1), mRNA | 2.024 |
| NM\_026693 | Mus musculus GABA(A) receptor-associated protein like 2 (Gabarapl2), mRNA | 2.023 |
| NM\_020026 | Mus musculus UDP-Gal:betaGlcNAc beta 1,3-galactosyltransferase, polypeptide 3 (B3galt3), mRNA | 2.021 |
| AK086795 | Mus musculus 0 day neonate lung cDNA, RIKEN full-length enriched library, clone:E030001K11 product:hypothetical Leucine-rich repeat/Ankyrin-repeat/ATP/GTP-binding site motif A (P-loop)/Leucine-rich repeat, typical subtype containing protein, full insert | 2.021 |
| NM\_024243 | Mus musculus fucosidase, alpha-L- 1, tissue (Fuca), mRNA | 2.020 |
| NM\_172506 | Mus musculus biregional cell adhesion molecule-related/down-regulated by oncogenes (Cdon) binding protein (Boc), mRNA | 2.016 |
| NM\_007644 | Mus musculus scavenger receptor class B, member 2 (Scarb2), mRNA | 2.016 |
| NM\_019722 | Mus musculus ADP-ribosylation factor-like 2 (Arl2), mRNA | 2.016 |
| TC975512 | AF406992 A-kinase anchoring protein {Homo sapiens}, partial (22%) | 2.013 |
| BC049774 | Mus musculus frizzled homolog 2 (Drosophila), mRNA (cDNA clone MGC:59284 IMAGE:6517721), complete cds. | 2.012 |
| A\_51\_P499979 | Unknown | 2.009 |
| NM\_008483 | Mus musculus laminin, beta 2 (Lamb2), mRNA | 2.009 |
| NM\_008364 | Mus musculus interleukin 1 receptor accessory protein (Il1rap), mRNA | 2.009 |
| BC011344 | Mus musculus cDNA clone MGC:5739 IMAGE:3481870, complete cds. | 2.009 |
| BC023820 | Mus musculus follicular lymphoma variant translocation 1, mRNA (cDNA clone MGC:36450 IMAGE:5354804), complete cds. | 2.008 |
| NM\_013876 | Mus musculus ring finger protein 11 (Rnf11), mRNA | 2.008 |
| NM\_053201 | Mus musculus melanoma antigen, family E, 1 (Magee1), mRNA | 2.008 |
| NM\_145070 | Mus musculus huntingtin interacting protein 1 related (Hip1r), mRNA | 2.008 |
| NM\_009001 | Mus musculus RAB3A, member RAS oncogene family (Rab3a), mRNA | 2.007 |
| BC024571 | Mus musculus cDNA clone IMAGE:5036677, partial cds. | 2.005 |
| BQ958019 | AGENCOURT\_8857443 NCI\_CGAP\_Mam2 Mus musculus cDNA clone IMAGE:6441431 5', mRNA sequence | 2.004 |
| NM\_172630 | Mus musculus metallophosphoesterase 1 (Mppe1), mRNA | 2.003 |
| NM\_023051 | Mus musculus calsyntenin 1 (Clstn1), mRNA | 2.003 |
| NM\_008071 | Mus musculus gamma-aminobutyric acid (GABA-A) receptor, subunit beta 3 (Gabrb3), mRNA | 2.002 |
| NM\_172579 | Mus musculus signal-induced proliferation-associated 1 like 1 (Sipa1l1), mRNA | 2.000 |
| BC049983 | Mus musculus gene model 944, (NCBI), mRNA (cDNA clone IMAGE:5150298), partial cds. | 2.000 |
| NM\_028181 | Mus musculus cell cycle progression 1 (Ccpg1), mRNA | 1.999 |
| NM\_019572 | Mus musculus histone deacetylase 7A (Hdac7a), mRNA | 1.998 |
| NM\_031163 | Mus musculus procollagen, type II, alpha 1 (Col2a1), mRNA | 1.998 |
| NM\_173401 | Mus musculus : F-box protein 44 (Fbxo44), mRNA | 1.996 |
| NM\_010360 | Mus musculus glutathione S-transferase, mu 5 (Gstm5), mRNA | 1.996 |
| BC029810 | Mus musculus phosphodiesterase 2A, cGMP-stimulated, mRNA (cDNA clone IMAGE:4949471), partial cds. | 1.994 |
| NM\_016707 | Mus musculus B-cell CLL/lymphoma 11A (zinc finger protein) (Bcl11a), mRNA | 1.994 |
| NM\_025424 | Mus musculus RIKEN cDNA 1110060M21 gene (1110060M21Rik), mRNA | 1.993 |
| NM\_025622 | Mus musculus lectin, galactose-binding, soluble 2 (Lgals2), mRNA | 1.991 |
| NM\_145586 | Mus musculus RIKEN cDNA 8430420C20 gene (8430420C20Rik), mRNA | 1.990 |
| AK018640 | Mus musculus adult male cecum cDNA, RIKEN full-length enriched library, clone:9130020G22 product:hypothetical protein, full insert sequence. | 1.989 |
| NM\_024190 | Mus musculus RIKEN cDNA 2810405I11 gene (2810405I11Rik), mRNA | 1.989 |
| NM\_176996 | Mus musculus smoothened homolog (Drosophila) (Smo), mRNA | 1.988 |
| NM\_175327 | Mus musculus RIKEN cDNA B630019K06 gene (B630019K06Rik), mRNA | 1.986 |
| NM\_023465 | Mus musculus catenin beta interacting protein 1 (Catnbip1), mRNA | 1.985 |
| XM\_142239 | Mus musculus similar to GTP-binding protein ragB (LOC245670), mRNA | 1.985 |
| BC048838 | Mus musculus RIKEN cDNA 5730417B17 gene, mRNA (cDNA clone MGC:54904 IMAGE:4948470), complete cds. | 1.984 |
| NM\_177879 | Mus musculus sidekick homolog 1 (chicken) (Sdk1), mRNA | 1.983 |
| AF171081 | Mus musculus histone macroH2A1.1 variant mRNA, partial cds. | 1.982 |
| NM\_027859 | Mus musculus RIKEN cDNA 0610009J22 gene (0610009J22Rik), mRNA | 1.982 |
| NM\_016858 | Mus musculus RAB33B, member of RAS oncogene family (Rab33b), mRNA | 1.982 |
| NM\_022417 | Mus musculus integral membrane protein 2C (Itm2c), mRNA | 1.981 |
| NM\_027789 | Mus musculus RIKEN cDNA 5430416O09 gene (5430416O09Rik), mRNA | 1.981 |
| NM\_010495 | Mus musculus inhibitor of DNA binding 1 (Idb1), mRNA | 1.979 |
| NM\_019760 | Mus musculus tumor differentially expressed 2 (Tde2), mRNA | 1.979 |
| NM\_172553 | Mus musculus cartilage homeo protein 1 (Cart1), mRNA | 1.978 |
| NM\_181391 | Mus musculus RIKEN cDNA 1810049H20 gene (1810049H20Rik), mRNA | 1.977 |
| NM\_008536 | Mus musculus transmembrane 4 superfamily member 1 (Tm4sf1), mRNA | 1.976 |
| NM\_007514 | Mus musculus solute carrier family 7 (cationic amino acid transporter, y+ system), member 2 (Slc7a2), mRNA | 1.975 |
| AK031598 | Mus musculus 13 days embryo male testis cDNA, RIKEN full-length enriched library, clone:6030458D19 product:mannan-binding lectin serine protease 1, full insert sequence. | 1.974 |
| AK004534 | Mus musculus 18-day embryo whole body cDNA, RIKEN full-length enriched library, clone:1190017B18 product:hypothetical P-loop containing nucleotide triphosphate hydrolases structure containing protein, full insert sequence. | 1.974 |
| NM\_009808 | Mus musculus caspase 12 (Casp12), mRNA | 1.973 |
| AK004186 | Mus musculus 18-day embryo whole body cDNA, RIKEN full-length enriched library, clone:1110046J04 product:unknown EST, full insert sequence. | 1.973 |
| NM\_175836 | Mus musculus spectrin beta 2 (Spnb2), transcript variant 1, mRNA | 1.973 |
| NM\_173781 | Mus musculus DNA segment, Chr 9, Brigham & Women's Genetics 0185 expressed (D9Bwg0185e), mRNA | 1.972 |
| NM\_021515 | Mus musculus adenylate kinase 1 (Ak1), mRNA | 1.972 |
| NM\_022982 | Mus musculus reticulon 4 receptor (Rtn4r), mRNA | 1.971 |
| NM\_023047 | Mus musculus dihydropyrimidinase-like 5 (Dpysl5), mRNA | 1.970 |
| AK012979 | Mus musculus 10, 11 days embryo whole body cDNA, RIKEN full-length enriched library, clone:2810404D04 product:unknown EST, full insert sequence. | 1.969 |
| AK013056 | Mus musculus 10, 11 days embryo whole body cDNA, RIKEN full-length enriched library, clone:2810409K11 product:hypothetical KRAB box containing protein, full insert sequence. | 1.962 |
| AF089734 | Mus musculus phospholemman precursor, mRNA, complete cds. | 1.962 |
| XM\_283603 | Mus musculus lymphoid nuclear protein related to AF4 (Laf4), mRNA | 1.962 |
| NM\_020606 | Mus musculus parvin, alpha (Parva), mRNA | 1.962 |
| AK049094 | Mus musculus 0 day neonate cerebellum cDNA, RIKEN full-length enriched library, clone:C230098G14 product:unclassifiable, full insert sequence. | 1.962 |
| AK005601 | Mus musculus adult male testis cDNA, RIKEN full-length enriched library, clone:1700001J11 product:similar to RING FINGER PROTEIN 19 (XY BODY PROTEIN) (XYBP) (GAMETOGENESIS EXPRESSED PROTEIN GEG-154) (UBCM4-INTERACTING PROTEIN 117) (UIP117) [Mus musculus | 1.961 |
| NM\_207233 | Mus musculus cDNA sequence BC040774 (BC040774), mRNA | 1.960 |
| AK173013 | Unknown | 1.958 |
| NM\_177614 | Mus musculus RIKEN cDNA 4632413K17 gene (4632413K17Rik), mRNA | 1.958 |
| AK042138 | Mus musculus 3 days neonate thymus cDNA, RIKEN full-length enriched library, clone:A630062P04 product:unknown EST, full insert sequence. | 1.957 |
| NM\_011890 | Mus musculus sarcoglycan, beta (dystrophin-associated glycoprotein) (Sgcb), mRNA | 1.957 |
| AK034889 | Mus musculus 12 days embryo embryonic body between diaphragm region and neck cDNA, RIKEN full-length enriched library, clone:9430057E15 product:cysteine-rich motor neuron 1, full insert sequence. | 1.953 |
| NM\_016783 | Mus musculus progesterone receptor membrane component 1 (Pgrmc1), mRNA | 1.952 |
| NM\_010723 | Mus musculus LIM domain only 4 (Lmo4), mRNA | 1.951 |
| NM\_023168 | Mus musculus glutamate receptor, ionotropic, N-methyl D-asparate-associated protein 1 (glutamate binding) (Grina), mRNA | 1.951 |
| NM\_009906 | Mus musculus ceroid-lipofuscinosis, neuronal 2 (Cln2), mRNA | 1.951 |
| NM\_025341 | Mus musculus abhydrolase domain containing 6 (Abhd6), mRNA | 1.949 |
| NM\_172601 | Mus musculus RIKEN cDNA 1500012D09 gene (1500012D09Rik), mRNA | 1.947 |
| NM\_010513 | Mus musculus insulin-like growth factor I receptor (Igf1r), mRNA | 1.944 |
| NM\_026887 | Mus musculus adaptor-related protein complex 1, sigma 2 subunit (Ap1s2), mRNA | 1.942 |
| NM\_024462 | Mus musculus RIKEN cDNA 2410005K17 gene (2410005K17Rik), mRNA | 1.941 |
| TC948448 | D49835 DNA-binding protein {Homo sapiens}, partial (32%) | 1.940 |
| NM\_016765 | Mus musculus dimethylarginine dimethylaminohydrolase 2 (Ddah2), mRNA | 1.940 |
| NM\_178764 | Mus musculus RIKEN cDNA B930006L02 gene (B930006L02Rik), mRNA | 1.938 |
| NM\_010764 | Mus musculus mannosidase 2, alpha B1 (Man2b1), mRNA | 1.938 |
| AK077250 | Mus musculus 11 days pregnant adult female ovary and uterus cDNA, RIKEN full-length enriched library, clone:5031422I09 product:NEURAL PLAKOPHILIN-RELATED ARM REPEAT PROTEIN (FRAGMENT) homolog [Salmo trutta], full insert sequence. | 1.938 |
| NM\_011577 | Mus musculus transforming growth factor, beta 1 (Tgfb1), mRNA | 1.938 |
| NM\_033268 | Mus musculus actinin alpha 2 (Actn2), mRNA | 1.938 |
| NM\_008905 | Mus musculus protein tyrosine phosphatase, receptor-type, F interacting protein, binding protein 2 (Ppfibp2), mRNA | 1.937 |
| NM\_177068 | Mus musculus RIKEN cDNA 4832415H08 gene (4832415H08Rik), mRNA | 1.937 |
| NM\_011594 | Mus musculus tissue inhibitor of metalloproteinase 2 (Timp2), mRNA | 1.936 |
| AK011935 | Mus musculus 10 days embryo whole body cDNA, RIKEN full-length enriched library, clone:2610209L14 product:SNF2alpha protein homolog [Homo sapiens], full insert sequence. | 1.935 |
| NM\_010438 | Mus musculus hexokinase 1 (Hk1), mRNA | 1.934 |
| AK033699 | Mus musculus adult male cecum cDNA, RIKEN full-length enriched library, clone:9130225G07 product:G PROTEIN-COUPLED RECEPTOR LGR4 homolog [Rattus norvegicus], full insert sequence. | 1.934 |
| AK122487 | Mus musculus mRNA for mKIAA1296 protein. | 1.933 |
| AK037008 | Mus musculus adult female vagina cDNA, RIKEN full-length enriched library, clone:9930036K04 product:unknown EST, full insert sequence | 1.932 |
| NM\_172529 | Mus musculus expressed sequence AU067744 (AU067744), mRNA | 1.932 |
| NM\_010286 | Mus musculus delta sleep inducing peptide, immunoreactor (Dsip1), mRNA | 1.931 |
| AK077382 | Mus musculus 6 days neonate head cDNA, RIKEN full-length enriched library, clone:5430409C21 product:unknown EST, full insert sequence | 1.930 |
| AK044329 | Mus musculus adult retina cDNA, RIKEN full-length enriched library, clone:A930007L12 product:unknown EST, full insert sequence. | 1.928 |
| NM\_011123 | Mus musculus proteolipid protein (myelin) (Plp), mRNA | 1.928 |
| AK028779 | Mus musculus 10 days neonate skin cDNA, RIKEN full-length enriched library, clone:4732455O04 product:inferred: POTENTIAL PHOSPHOLIPID-TRANSPORTING ATPASE IH (EC 3.6.3.13). [Mouse] {Mus musculus}, full insert sequence. | 1.927 |
| AY578330 | Mus musculus calpain 11 (Capn11) mRNA, complete cds. | 1.925 |
| NM\_009372 | Mus musculus TG interacting factor (Tgif), mRNA | 1.924 |
| NM\_053177 | Mus musculus mucolipin 1 (Mcoln1), mRNA | 1.924 |
| NM\_008906 | Mus musculus protective protein for beta-galactosidase (Ppgb), mRNA | 1.923 |
| AK122431 | Mus musculus mRNA for mKIAA1058 protein. | 1.923 |
| NM\_009145 | Mus musculus stromal cell derived factor receptor 1 (Sdfr1), mRNA | 1.921 |
| AK013404 | Mus musculus 10, 11 days embryo whole body cDNA, RIKEN full-length enriched library, clone:2810473M14 product:unknown EST, full insert sequence. | 1.920 |
| XM\_129479 | Mus musculus similar to Lactase-phlorizin hydrolase precursor (Lactase-glycosylceramidase) (LOC226413), mRNA | 1.920 |
| NM\_031394 | Mus musculus synaptotagmin-like 2 (Sytl2), mRNA | 1.919 |
| NM\_130885 | Mus musculus oxidation resistance 1 (Oxr1), mRNA | 1.919 |
| NM\_023670 | Mus musculus insulin-like growth factor 2, binding protein 3 (Igf2bp3), mRNA | 1.919 |
| AK016675 | Mus musculus adult male testis cDNA, RIKEN full-length enriched library, clone:4933405M22 product:hypothetical protein, full insert sequence. | 1.918 |
| AK004276 | Mus musculus 18-day embryo whole body cDNA, RIKEN full-length enriched library, clone:1110056P05 product:unclassifiable, full insert sequence. | 1.917 |
| NM\_029814 | Mus musculus RIKEN cDNA 2210412K09 gene (2210412K09Rik), mRNA | 1.917 |
| NM\_011901 | Mus musculus TAF7 RNA polymerase II, TATA box binding protein (TBP)-associated factor (Taf7), mRNA | 1.916 |
| AK075797 | Mus musculus 10 day old male pancreas cDNA, RIKEN full-length enriched library, clone:1810049E24 product:DNA segment, Chr 12, ERATO Doi 647, expressed, full insert sequence. | 1.915 |
| NM\_023422 | Mus musculus histone 1, H2bc (Hist1h2bc), mRNA | 1.915 |
| BC027278 | Mus musculus mRNA similar to heat shock protein, 30 kDa (cDNA clone IMAGE:3483818) | 1.915 |
| NM\_016892 | Mus musculus copper chaperone for superoxide dismutase (Ccs), mRNA | 1.915 |
| NM\_009895 | Mus musculus cytokine inducible SH2-containing protein (Cish), mRNA | 1.915 |
| NM\_146131 | Mus musculus pre-B-cell leukemia transcription factor interacting protein 1 (Pbxip1), mRNA | 1.914 |
| NM\_028846 | Mus musculus ubiquitin specific protease 20 (Usp20), mRNA | 1.913 |
| NM\_029131 | Mus musculus RIKEN cDNA 4930503E14 gene (4930503E14Rik), mRNA | 1.913 |
| NM\_008197 | Mus musculus H1 histone family, member 0 (H1f0), mRNA | 1.913 |
| NM\_146917 | Mus musculus olfactory receptor 1179 (Olfr1179), mRNA | 1.912 |
| NM\_172574 | Mus musculus RIKEN cDNA E030024M05 gene (E030024M05Rik), mRNA | 1.912 |
| J00623 | MUSRGEB3 Mus musculus 18S ribosomal RNA gene, partial sequence; internal transcribed spacer 1, 5.8S ribosomal RNA gene and internal transcribed spacer 1, complete sequence; and 28S ribosomal RNA gene, partial sequence | 1.911 |
| NM\_010349 | Mus musculus glutamate receptor, ionotropic, kainate 2 (beta 2) (Grik2), mRNA | 1.909 |
| NM\_172856 | Mus musculus longevity assurance homolog 6 (S. cerevisiae) (Lass6), mRNA | 1.905 |
| NM\_178890 | Mus musculus expressed sequence AW539457 (AW539457), mRNA | 1.905 |
| NM\_023835 | Mus musculus tripartite motif protein 12 (Trim12), mRNA | 1.903 |
| AK018420 | Mus musculus 16 days embryo lung cDNA, RIKEN full-length enriched library, clone:8430416G17 product:hypothetical Cysteine-rich region containing protein, full insert sequence. | 1.902 |
| AK013457 | Mus musculus adult male hippocampus cDNA, RIKEN full-length enriched library, clone:2900001A12 product:hypothetical protein, full insert sequence. | 1.901 |
| NM\_175149 | Mus musculus RIKEN cDNA 2310022B05 gene (2310022B05Rik), mRNA | 1.900 |
| NM\_177687 | Mus musculus hypothetical protein B230205M03 (B230205M03), mRNA | 1.899 |
| NM\_030706 | Mus musculus tripartite motif protein 2 (Trim2), mRNA | 1.899 |
| NM\_010006 | Mus musculus cytochrome P450, family 2, subfamily d, polypeptide 9 (Cyp2d9), mRNA | 1.898 |
| NM\_025445 | Mus musculus ADP-ribosylation factor GTPase activating protein 3 (Arfgap3), mRNA | 1.898 |
| BC013803 | Mus musculus mannosidase, beta A, lysosomal-like, mRNA (cDNA clone MGC:19248 IMAGE:3674568), complete cds. | 1.894 |
| NM\_008859 | Mus musculus protein kinase C, theta (Prkcq), mRNA | 1.894 |
| NM\_053131 | Mus musculus protocadherin beta 6 (Pcdhb6), mRNA | 1.893 |
| NM\_009318 | Mus musculus TAP binding protein (Tapbp), mRNA | 1.893 |
| S63763 | me/Hcph (mev)=viable motheaten/hematopoietic cell protein-tyrosine phosphatase {insertion} [mice, mRNA Partial Mutant, 120 nt]. | 1.892 |
| NM\_016975 | Mus musculus gap junction membrane channel protein alpha 3 (Gja3), mRNA | 1.892 |
| BC062811 | Mus musculus cDNA clone MGC:73649 IMAGE:1264501, complete cds. | 1.892 |
| NM\_009502 | Mus musculus vinculin (Vcl), mRNA | 1.892 |
| NM\_026200 | Mus musculus potassium channel, subfamily V, member 1 (Kcnv1), mRNA | 1.892 |
| NM\_172256 | Mus musculus RIKEN cDNA 4933404O11 gene (4933404O11Rik), mRNA | 1.889 |
| NM\_011413 | Mus musculus sex-limited protein (Slp), mRNA | 1.889 |
| BC052489 | Mus musculus RIKEN cDNA 3110001I20 gene, mRNA (cDNA clone IMAGE:6439419), partial cds | 1.889 |
| NM\_007679 | Mus musculus CCAAT/enhancer binding protein (C/EBP), delta (Cebpd), mRNA | 1.886 |
| NM\_026432 | Mus musculus RIKEN cDNA 1810045K07 gene (1810045K07Rik), mRNA | 1.886 |
| NM\_173733 | Mus musculus sulfite oxidase (Suox), mRNA | 1.886 |
| AK029143 | Mus musculus 10 days neonate skin cDNA, RIKEN full-length enriched library, clone:4732496O19 product:MEMBRANE-ASSOCIATED GUANYLATE KINASE-RELATED MAGI-3 homolog [Mus musculus], full insert sequence. | 1.884 |
| NM\_146126 | Mus musculus sorbitol dehydrogenase 1 (Sdh1), mRNA | 1.883 |
| NM\_009255 | Mus musculus serine (or cysteine) proteinase inhibitor, clade E, member 2 (Serpine2), mRNA | 1.882 |
| NM\_022432 | Mus musculus sirtuin 2 (silent mating type information regulation 2, homolog) 2 (S. cerevisiae) (Sirt2), mRNA | 1.882 |
| NM\_001001602 | Mus musculus disabled homolog 2 (Drosophila) interacting protein (Dab2ip), mRNA | 1.881 |
| AK122235 | Mus musculus mRNA for mKIAA0280 protein. | 1.880 |
| BC034073 | Mus musculus avian musculoaponeurotic fibrosarcoma (v-maf) AS42 oncogene homolog, mRNA (cDNA clone IMAGE:4221113), partial cds. | 1.878 |
| NM\_175123 | Mus musculus RIKEN cDNA 1110051M20 gene (1110051M20Rik), transcript variant 2, mRNA | 1.878 |
| NM\_009775 | Mus musculus benzodiazepine receptor, peripheral (Bzrp), mRNA | 1.877 |
| NM\_153521 | Mus musculus RIKEN cDNA D630045E04 gene (D630045E04Rik), mRNA | 1.876 |
| NM\_153557 | Mus musculus cDNA sequence BC029214 (BC029214), mRNA | 1.875 |
| NM\_146258 | Mus musculus serologically defined colon cancer antigen 13 (Stard13), mRNA | 1.874 |
| NM\_026137 | Mus musculus WD repeat domain 13 (Wdr13), mRNA | 1.873 |
| BC042711 | Mus musculus RIKEN cDNA 3100002L24 gene, mRNA (cDNA clone MGC:51354 IMAGE:4503689), complete cds. | 1.873 |
| BC037033 | Mus musculus solute carrier family 41, member 1, mRNA (cDNA clone IMAGE:4504894), partial cds | 1.872 |
| NM\_146251 | Mus musculus cDNA sequence BC027342 (BC027342), mRNA | 1.869 |
| AK013800 | Mus musculus adult male hippocampus cDNA, RIKEN full-length enriched library, clone:2900078C09 product:hypothetical protein, full insert sequence. | 1.869 |
| AK004226 | Mus musculus 18-day embryo whole body cDNA, RIKEN full-length enriched library, clone:1110051A18 product:hypothetical protein, full insert sequence. | 1.867 |
| BC050903 | Mus musculus mRNA similar to double cortin and calcium/calmodulin-dependent protein kinase-like 1 (cDNA clone MGC:63284 IMAGE:6416546), complete cds. | 1.867 |
| XM\_130277 | Mus musculus plakophilin 4 (Pkp4), mRNA | 1.866 |
| J05277 | Mouse hexokinase mRNA, complete cds. | 1.866 |
| NM\_020578 | Mus musculus EH-domain containing 3 (Ehd3), mRNA | 1.866 |
| NM\_010387 | Mus musculus histocompatibility 2, class II, locus Mb1 (H2-DMb1), mRNA | 1.864 |
| NM\_053267 | Mus musculus selenoprotein M (Sepm), mRNA | 1.864 |
| NM\_027251 | Mus musculus RIKEN cDNA 2010107G23 gene (2010107G23Rik), mRNA | 1.863 |
| NM\_172599 | Mus musculus DNA segment, Chr 14, ERATO Doi 436, expressed (D14Ertd436e), mRNA | 1.863 |
| NM\_027857 | Mus musculus RIKEN cDNA 0610006H10 gene (0610006H10Rik), mRNA | 1.863 |
| NAP093165-001 | Unknown | 1.863 |
| AK005381 | Mus musculus adult male cerebellum cDNA, RIKEN full-length enriched library, clone:1500041B16 product:hypothetical protein, full insert sequence. | 1.862 |
| NM\_026883 | Mus musculus RIKEN cDNA 1500011H22 gene (1500011H22Rik), mRNA | 1.861 |
| NM\_009115 | Mus musculus S100 protein, beta polypeptide, neural (S100b), mRNA | 1.860 |
| AK052456 | Mus musculus 13 days embryo lung cDNA, RIKEN full-length enriched library, clone:D430026P16 product:weakly similar to TRANSCRIPTIONAL REGULATOR [Methanothermobacter thermautotrophicus], full insert sequence | 1.860 |
| NM\_019654 | Mus musculus suppressor of cytokine signaling 5 (Socs5), mRNA | 1.860 |
| NM\_010684 | Mus musculus lysosomal membrane glycoprotein 1 (Lamp1), mRNA | 1.860 |
| NM\_201389 | Mus musculus plectin 1 (Plec1), transcript variant 6, mRNA | 1.859 |
| NM\_007945 | Mus musculus epidermal growth factor receptor pathway substrate 8 (Eps8), mRNA | 1.859 |
| NM\_027287 | Mus musculus DnaJ (Hsp40) homolog, subfamily B, member 4 (Dnajb4), mRNA | 1.858 |
| NM\_027935 | Mus musculus RIKEN cDNA 3200001F09 gene (3200001F09Rik), mRNA | 1.858 |
| NM\_153195 | Mus musculus F-box only protein 7 (Fbxo7), mRNA | 1.857 |
| NM\_133677 | Mus musculus RIKEN cDNA 2310061J03 gene (2310061J03Rik), mRNA | 1.857 |
| ENSMUST00000023249 | Unknown | 1.857 |
| NM\_175134 | Mus musculus RIKEN cDNA 1110054N06 gene (1110054N06Rik), mRNA | 1.856 |
| NM\_170599 | Mus musculus immunoglobulin superfamily, member 11 (Igsf11), mRNA | 1.854 |
| NM\_009794 | Mus musculus calpain 2 (Capn2), mRNA | 1.854 |
| NM\_021099 | Mus musculus kit oncogene (Kit), mRNA | 1.852 |
| XM\_133979 | Mus musculus RIKEN cDNA C030014K08 gene (C030014K08Rik), mRNA | 1.851 |
| NM\_026897 | Mus musculus hydroxyacylglutathione hydrolase-like (Haghl), mRNA | 1.850 |
| NM\_172868 | Mus musculus paralemmin 2 (Palm2), mRNA | 1.850 |
| AK047461 | Mus musculus 10 days neonate cerebellum cDNA, RIKEN full-length enriched library, clone:B930067C23 product:unknown EST, full insert sequence. | 1.849 |
| NM\_007379 | Mus musculus ATP-binding cassette, sub-family A (ABC1), member 2 (Abca2), mRNA | 1.848 |
| NM\_181070 | Mus musculus RAB18, member RAS oncogene family (Rab18), mRNA | 1.847 |
| NM\_025917 | Mus musculus RIKEN cDNA 2010315L10 gene (2010315L10Rik), mRNA | 1.847 |
| BC002262 | Mus musculus cDNA clone MGC:7623 IMAGE:3495045, complete cds. | 1.847 |
| NM\_011992 | Mus musculus reticulocalbin 2 (Rcn2), mRNA | 1.847 |
| AK122403 | Mus musculus mRNA for mKIAA0933 protein. | 1.846 |
| NM\_153534 | Mus musculus adenylate cyclase 2 (Adcy2), mRNA | 1.845 |
| NM\_172689 | Mus musculus DEAD (Asp-Glu-Ala-Asp) box polypeptide 58 (Ddx58), mRNA | 1.843 |
| BC024833 | Mus musculus, clone IMAGE:5361724, mRNA, partial cds. | 1.843 |
| NM\_021494 | Mus musculus Rab6 interacting protein 1 (Rab6ip1), mRNA | 1.842 |
| NM\_023277 | Mus musculus junction adhesion molecule 3 (Jam3), mRNA | 1.842 |
| BC052504 | Mus musculus cDNA clone IMAGE:1244570, partial cds | 1.842 |
| NM\_010281 | Mus musculus gamma-glutamyl hydrolase (Ggh), mRNA | 1.837 |
| NM\_024465 | Mus musculus RIKEN cDNA 6330583M11 gene (6330583M11Rik), mRNA | 1.836 |
| AK031204 | Mus musculus 13 days embryo forelimb cDNA, RIKEN full-length enriched library, clone:5930429B03 product:unknown EST, full insert sequence | 1.834 |
| NM\_175344 | Mus musculus transmembrane protein 16F (Tmem16f), mRNA | 1.834 |
| TC1075190 | JAK1\_MOUSE Tyrosine-protein kinase JAK1(Janus kinase 1) (JAK-1). [Mouse] {Mus musculus}, complete | 1.833 |
| NM\_212470 | Mus musculus RIKEN cDNA 0610007C21 gene (0610007C21Rik), transcript variant 2, mRNA | 1.831 |
| NM\_033134 | Mus musculus inositol polyphosphate-5-phosphatase E (Inpp5e), mRNA | 1.830 |
| AK129160 | Mus musculus mRNA for mKIAA0543 protein | 1.829 |
| NM\_010011 | Mus musculus cytochrome P450, family 4, subfamily a, polypeptide 10 (Cyp4a10), mRNA | 1.827 |
| NM\_026170 | Mus musculus RIKEN cDNA 1200007D18 gene (1200007D18Rik), mRNA | 1.827 |
| NM\_009923 | Mus musculus cyclic nucleotide phosphodiesterase 1 (Cnp1), mRNA | 1.826 |
| NM\_008301 | Mus musculus heat shock protein 2 (Hspa2), transcript variant 1, mRNA | 1.826 |
| NM\_021430 | Mus musculus RIKEN cDNA 2900002H16 gene (2900002H16Rik), mRNA | 1.826 |
| NM\_021568 | Mus musculus poly(rC) binding protein 3 (Pcbp3), mRNA | 1.825 |
| AK047312 | Mus musculus 10 days neonate cerebellum cDNA, RIKEN full-length enriched library, clone:B930048G21 product:hypothetical Rhodanese/cdc25 fold/Rhodanese signatures containing protein, full insert sequence. | 1.825 |
| NM\_008512 | Mus musculus low density lipoprotein receptor-related protein 1 (Lrp1), mRNA | 1.824 |
| AK053139 | Mus musculus 0 day neonate lung cDNA, RIKEN full-length enriched library, clone:E030012J21 product:tripartite motif protein 16, full insert sequence. | 1.824 |
| NM\_030127 | Mus musculus RIKEN cDNA 9530081K03 gene (9530081K03Rik), mRNA | 1.824 |
| NM\_019990 | Mus musculus START domain containing 10 (Stard10), mRNA | 1.823 |
| BC025841 | Mus musculus cDNA clone IMAGE:5149318, partial cds. | 1.823 |
| TC956884 | AF251290 glutamic acid-rich protein {Plasmodium falciparum}, partial (5%) | 1.821 |
| NM\_013862 | Mus musculus RIKEN cDNA 9630005B12 gene (9630005B12Rik), mRNA | 1.820 |
| AK004618 | Mus musculus adult male lung cDNA, RIKEN full-length enriched library, clone:1200006M05 product:similar to PC326 PROTEIN [Homo sapiens], full insert sequence. | 1.820 |
| NM\_144935 | Mus musculus cDNA sequence BC018242 (BC018242), mRNA | 1.820 |
| NAP028770-1 | Unknown | 1.820 |
| BC003494 | Mus musculus protein O-fucosyltransferase 2, mRNA (cDNA clone IMAGE:2812143), complete cds. | 1.820 |
| NM\_011728 | Mus musculus xeroderma pigmentosum, complementation group A (Xpa), mRNA | 1.819 |
| NM\_007709 | Mus musculus Cbp/p300-interacting transactivator with Glu/Asp-rich carboxy-terminal domain 1 (Cited1), mRNA | 1.818 |
| AK012837 | Mus musculus 10, 11 days embryo whole body cDNA, RIKEN full-length enriched library, clone:2810028G24 product:utrophin, full insert sequence. | 1.818 |
| NM\_033521 | Mus musculus lysosomal-associated protein transmembrane 4B (Laptm4b), mRNA | 1.817 |
| BC046641 | Mus musculus low density lipoprotein receptor-related protein associated protein 1, mRNA (cDNA clone IMAGE:5135375), partial cds. | 1.817 |
| NM\_028021 | Mus musculus myosin, heavy polypeptide 14 (Myh14), mRNA | 1.815 |
| AK012553 | Mus musculus 11 days embryo whole body cDNA, RIKEN full-length enriched library, clone:2700082O15 product:FETAL BRAIN PROTEIN 239 (239FB) homolog [Homo sapiens], full insert sequence. | 1.814 |
| NM\_013495 | Mus musculus carnitine palmitoyltransferase 1a, liver (Cpt1a), mRNA | 1.814 |
| NM\_008782 | Mus musculus paired box gene 5 (Pax5), mRNA | 1.814 |
| NM\_013646 | Mus musculus RAR-related orphan receptor alpha (Rora), mRNA | 1.814 |
| NM\_026416 | Mus musculus S100 calcium binding protein A16 (S100a16), mRNA | 1.813 |
| BC038619 | Mus musculus cDNA clone IMAGE:4219727, partial cds | 1.812 |
| NM\_198410 | Mus musculus RIKEN cDNA 1500001B10 gene (1500001B10Rik), mRNA | 1.811 |
| NM\_207217 | Mus musculus expressed sequence AI429612 (AI429612), mRNA | 1.811 |
| NM\_009433 | Mus musculus testis-specific protein, Y-encoded-like (Tspyl), mRNA | 1.810 |
| NM\_010425 | Mus musculus forkhead box D3 (Foxd3), mRNA | 1.809 |
| BC006692 | Mus musculus cDNA clone MGC:7898 IMAGE:3582717, complete cds. | 1.809 |
| NM\_146260 | Mus musculus transmembrane inner ear (Tmie), mRNA | 1.808 |
| NM\_029657 | Mus musculus mahogunin, ring finger 1 (Mgrn1), mRNA | 1.807 |
| AK021409 | Mus musculus 0 day neonate eyeball cDNA, RIKEN full-length enriched library, clone:E130302J09 product:hypothetical protein, full insert sequence. | 1.807 |
| NM\_145930 | Mus musculus expressed sequence AW549877 (AW549877), mRNA | 1.806 |
| NM\_134096 | Mus musculus expressed sequence AW049604 (AW049604), mRNA | 1.806 |
| NM\_010308 | Mus musculus guanine nucleotide binding protein, alpha o (Gnao), mRNA | 1.805 |
| NM\_022999 | Mus musculus proline-rich Gla (G-carboxyglutamic acid) polypeptide 2 (Prrg2), mRNA | 1.804 |
| NM\_009469 | Mus musculus Unc-51 like kinase 1 (C. elegans) (Ulk1), mRNA | 1.804 |
| NM\_024451 | Mus musculus unc-84 homolog A (C. elegans) (Unc84a), mRNA | 1.804 |
| BC023406 | Mus musculus mRNA similar to hypothetical protein FLJ21936 (cDNA clone IMAGE:5031343). | 1.803 |
| NM\_029100 | Mus musculus selenoprotein N, 1 (Sepn1), mRNA | 1.802 |
| NM\_199310 | Mus musculus gene model 631, (NCBI) (Gm631), mRNA | 1.801 |
| NM\_144819 | Mus musculus DNA segment, Chr 5, Brigham & Women's Genetics 0834 expressed (D5Bwg0834e), mRNA | 1.801 |
| NM\_010388 | Mus musculus histocompatibility 2, class II, locus Mb2 (H2-DMb2), mRNA | 1.801 |
| AK129274 | Mus musculus mRNA for mKIAA1062 protein. | 1.799 |
| NM\_011682 | Mus musculus utrophin (Utrn), mRNA | 1.798 |
| NM\_130861 | Mus musculus solute carrier organic anion transporter family, member 1a5 (Slco1a5), mRNA | 1.797 |
| NM\_181401 | Mus musculus RIKEN cDNA 9630015D15 gene (9630015D15Rik), mRNA | 1.796 |
| AK050117 | Mus musculus adult male liver tumor cDNA, RIKEN full-length enriched library, clone:C730016N02 product:unknown EST, full insert sequence. | 1.796 |
| AK028246 | Mus musculus 12 days embryo head cDNA, RIKEN full-length enriched library, clone:3000002B05 product:lymphoid enhancer binding factor 1, full insert sequence. | 1.796 |
| NM\_009614 | Mus musculus a disintegrin and metalloproteinase domain 15 (metargidin) (Adam15), mRNA | 1.796 |
| NM\_010753 | Mus musculus Max dimerization protein 4 (Mxd4), mRNA | 1.795 |
| NM\_020564 | Mus musculus sulfotransferase family 5A, member 1 (Sult5a1), mRNA | 1.795 |
| NM\_145978 | Mus musculus PDZ and LIM domain 2 (Pdlim2), mRNA | 1.795 |
| NM\_172606 | Mus musculus RIKEN cDNA F830029L24 gene (F830029L24Rik), mRNA | 1.795 |
| NM\_013865 | Mus musculus N-myc downstream regulated gene 3 (Ndrg3), mRNA | 1.794 |
| BC061132 | Mus musculus cDNA clone MGC:74262 IMAGE:30295979, complete cds. | 1.794 |
| XM\_148244 | Mus musculus golgi autoantigen, golgin subfamily b, macrogolgin 1 (Golgb1), mRNA | 1.794 |
| AB024689 | Mus musculus gene, exon 3, partial sequence | 1.794 |
| NM\_025457 | Mus musculus RIKEN cDNA 1810008A14 gene (1810008A14Rik), mRNA | 1.793 |
| NM\_145402 | Mus musculus cDNA sequence BC003277 (BC003277), mRNA | 1.792 |
| AK045648 | Mus musculus adult male corpora quadrigemina cDNA, RIKEN full-length enriched library, clone:B230218H05 product:weakly similar to MSZF87 (FRAGMENT) [Mus musculus], full insert sequence | 1.791 |
| NM\_013736 | Mus musculus transcription elongation factor B (SIII), polypeptide 3 (Tceb3), mRNA | 1.790 |
| U34361 | Mus musculus lymphoid nuclear protein (LAF-4) mRNA, partial cds. | 1.789 |
| NM\_033146 | Mus musculus RIKEN cDNA 1500005A01 gene (1500005A01Rik), mRNA | 1.789 |
| NM\_009332 | Mus musculus transcription factor 3 (Tcf3), mRNA | 1.789 |
| NM\_026373 | Mus musculus DNA segment, Chr 19, ERATO Doi 144, expressed (D19Ertd144e), mRNA | 1.788 |
| AK079249 | Mus musculus adult male urinary bladder cDNA, RIKEN full-length enriched library, clone:9530052P13 product:GERMINAL CENTER KINASE RELATED PROTEIN KINASE homolog [Homo sapiens], full insert sequence. | 1.787 |
| NM\_031874 | Mus musculus RAB3D, member RAS oncogene family (Rab3d), mRNA | 1.787 |
| AK035894 | Mus musculus 16 days neonate cerebellum cDNA, RIKEN full-length enriched library, clone:9630015D15 product:hypothetical protein, full insert sequence. | 1.786 |
| NM\_021458 | Mus musculus frizzled homolog 3 (Drosophila) (Fzd3), mRNA | 1.786 |
| NM\_009131 | Mus musculus stem cell growth factor (Scgf), mRNA | 1.786 |
| NM\_177001 | Mus musculus RIKEN cDNA 9130023H24 gene (9130023H24Rik), mRNA | 1.785 |
| NM\_025889 | Mus musculus RIKEN cDNA 2410001H17 gene (2410001H17Rik), mRNA | 1.784 |
| NM\_028747 | Mus musculus RIKEN cDNA 0610012H03 gene (0610012H03Rik), mRNA | 1.784 |
| NM\_011050 | Mus musculus programmed cell death 4 (Pdcd4), mRNA | 1.783 |
| NM\_023483 | Mus musculus RIKEN cDNA 1110032A03 gene (1110032A03Rik), mRNA | 1.783 |
| NM\_011723 | Mus musculus xanthine dehydrogenase (Xdh), mRNA | 1.781 |
| AK031222 | Mus musculus 13 days embryo forelimb cDNA, RIKEN full-length enriched library, clone:5930431H10 product:hypothetical Signal peptidase containing protein, full insert sequence. | 1.778 |
| NM\_011415 | Mus musculus snail homolog 2 (Drosophila) (Snai2), mRNA | 1.778 |
| NM\_013642 | Mus musculus dual specificity phosphatase 1 (Dusp1), mRNA | 1.778 |
| NM\_011693 | Mus musculus vascular cell adhesion molecule 1 (Vcam1), mRNA | 1.777 |
| BC048857 | Mus musculus integrin alpha V, mRNA (cDNA clone IMAGE:5362788), partial cds. | 1.774 |
| AK017138 | Mus musculus adult male testis cDNA, RIKEN full-length enriched library, clone:4933440N22 product:hypothetical protein, full insert sequence. | 1.772 |
| NM\_177698 | Mus musculus hypothetical protein D430018P08 (D430018P08), mRNA | 1.771 |
| NM\_011179 | Mus musculus prosaposin (Psap), mRNA | 1.771 |
| NM\_177740 | Mus musculus RGM domain family, member A (Rgma), mRNA | 1.771 |
| NM\_010846 | Mus musculus myxovirus (influenza virus) resistance 1 (Mx1), mRNA | 1.770 |
| AK021199 | Mus musculus ES cells cDNA, RIKEN full-length enriched library, clone:C330013J21 product:similar to KRUPPEL-RELATED ZINC FINGER PROTEIN F80-L [Mus musculus], full insert sequence. | 1.770 |
| NM\_022656 | Mus musculus nischarin (Nisch), mRNA | 1.769 |
| NM\_021528 | Mus musculus carbohydrate sulfotransferase 12 (Chst12), mRNA | 1.769 |
| NM\_007437 | Mus musculus aldehyde dehydrogenase family 3, subfamily A2 (Aldh3a2), mRNA | 1.769 |
| NM\_007478 | Mus musculus ADP-ribosylation factor 3 (Arf3), mRNA | 1.768 |
| NM\_033525 | Mus musculus nephronectin (Npnt), mRNA | 1.766 |
| NM\_025950 | Mus musculus cell division cycle 37 homolog (S. cerevisiae)-like (Cdc37l), mRNA | 1.766 |
| NM\_133716 | Mus musculus RIKEN cDNA 1810031K02 gene (1810031K02Rik), mRNA | 1.765 |
| NM\_025334 | Mus musculus RIKEN cDNA 0610040B21 gene (0610040B21Rik), mRNA | 1.764 |
| NM\_172372 | Mus musculus WD repeat domain, X-linked 1 (Wdrx1), mRNA | 1.763 |
| AK008803 | Mus musculus adult male stomach cDNA, RIKEN full-length enriched library, clone:2210402M20 product:hypothetical Src homology 2 (SH2) domain containing protein, full insert sequence. | 1.763 |
| NM\_011854 | Mus musculus 2'-5' oligoadenylate synthetase-like 2 (Oasl2), mRNA | 1.763 |
| NM\_133838 | Mus musculus EH-domain containing 4 (Ehd4), mRNA | 1.762 |
| AK079669 | Mus musculus 0 day neonate thymus cDNA, RIKEN full-length enriched library, clone:A430002D02 product:unclassifiable, full insert sequence | 1.761 |
| NM\_019924 | Mus musculus ribosomal protein S6 kinase, polypeptide 4 (Rps6ka4), mRNA | 1.761 |
| NM\_009691 | Mus musculus amyloid beta (A4) precursor-like protein 2 (Aplp2), mRNA | 1.760 |
| S60315 | DMR-B15=myotonic dystrophy kinase {3' region, alternatively spliced, clone delta V} [mice, brain, mRNA Partial, 2474 nt]. | 1.757 |
| NM\_013872 | Mus musculus phosphomannomutase 1 (Pmm1), mRNA | 1.756 |
| AF464177 | Mus musculus protocadherin mRNA, partial cds; alternatively spliced. | 1.756 |
| NM\_030262 | Mus musculus protein O-fucosyltransferase 2 (Pofut2), mRNA | 1.755 |
| AK089462 | Mus musculus B6-derived CD11 +ve dendritic cells cDNA, RIKEN full-length enriched library, clone:F730035E03 product:DJ687F11.2 (NOVEL PROTEIN (CONTAINS PART OF TRANSLATION OF CDNA DKFZP434N061, EM:AL110249)) (FRAGMENT) homolog [Homo sapiens], full inser | 1.755 |
| NM\_080563 | Mus musculus ring finger protein 144 (Rnf144), mRNA | 1.755 |
| BC021944 | Mus musculus RIKEN cDNA 2310040A07 gene, mRNA (cDNA clone MGC:37775 IMAGE:5097248), complete cds. | 1.755 |
| NM\_029934 | Mus musculus RIKEN cDNA 5730589L02 gene (5730589L02Rik), mRNA | 1.754 |
| NM\_008786 | Mus musculus protein-L-isoaspartate (D-aspartate) O-methyltransferase 1 (Pcmt1), mRNA | 1.754 |
| NM\_021457 | Mus musculus frizzled homolog 1 (Drosophila) (Fzd1), mRNA | 1.752 |
| NM\_201359 | Mus musculus DNA segment, Chr 15, ERATO Doi 405, expressed (D15Ertd405e), mRNA | 1.752 |
| AK016443 | Mus musculus adult male testis cDNA, RIKEN full-length enriched library, clone:4931408A02 product:hypothetical D-galactoside/L-rhamnose binding SUEL lectin domain containing protein, full insert sequence. | 1.751 |
| NM\_025769 | Mus musculus RIKEN cDNA 5430404L10 gene (5430404L10Rik), mRNA | 1.751 |
| NM\_007981 | Mus musculus acyl-CoA synthetase long-chain family member 1 (Acsl1), mRNA | 1.750 |
| AK080748 | Mus musculus adult retina cDNA, RIKEN full-length enriched library, clone:A930037H23 product:hypothetical protein, full insert sequence. | 1.748 |
| NM\_134083 | Mus musculus chromosome condensation 1-like (Chc1l), mRNA | 1.748 |
| BC066088 | Mus musculus cDNA clone IMAGE:6837954, containing frame-shift errors | 1.748 |
| NM\_148937 | Mus musculus phospholipase C, delta 4 (Plcd4), mRNA | 1.748 |
| NM\_010145 | Mus musculus epoxide hydrolase 1, microsomal (Ephx1), mRNA | 1.747 |
| AK013062 | Mus musculus 10, 11 days embryo whole body cDNA, RIKEN full-length enriched library, clone:2810410A03 product:unknown EST, full insert sequence. | 1.747 |
| NM\_009984 | Mus musculus cathepsin L (Ctsl), mRNA | 1.747 |
| NM\_023794 | Mus musculus ets variant gene 5 (Etv5), mRNA | 1.747 |
| NM\_026875 | Mus musculus RIKEN cDNA 0610043B10 gene (0610043B10Rik), mRNA | 1.747 |
| TC1050509 | IDS\_MOUSE Iduronate 2-sulfatase precursor. [Mouse] {Mus musculus}, partial (95%) | 1.746 |
| NM\_026037 | Mus musculus O-acyltransferase (membrane bound) domain containing 2 (Oact2), mRNA | 1.746 |
| X15052 | Mouse mRNA for 3'-end of NCAM-140 and NCAM-180 isoforms. | 1.744 |
| NM\_134030 | Mus musculus RIKEN cDNA 4632423N09 gene (4632423N09Rik), mRNA | 1.744 |
| NM\_019963 | Mus musculus signal transducer and activator of transcription 2 (Stat2), mRNA | 1.743 |
| AK081137 | Mus musculus 10 days neonate cerebellum cDNA, RIKEN full-length enriched library, clone:B930091O06 product:CORTACTIN-BINDING PROTEIN 2 homolog [Homo sapiens], full insert sequence. | 1.743 |
| AV075202 | AV075202 AV075202 Mus musculus stomach C57BL/6J adult Mus musculus cDNA clone 2210012H21, mRNA sequence | 1.742 |
| NM\_011933 | Mus musculus 2-4-dienoyl-Coenzyme A reductase 2, peroxisomal (Decr2), mRNA | 1.741 |
| NM\_130864 | Mus musculus acetyl-Coenzyme A acyltransferase 1 (Acaa1), mRNA | 1.741 |
| AK078797 | Mus musculus 15 days embryo embryonic body below diaphragm cDNA, RIKEN full-length enriched library, clone:8230402D24 product:unknown EST, full insert sequence. | 1.741 |
| NM\_053141 | Mus musculus protocadherin beta 16 (Pcdhb16), mRNA | 1.740 |
| NM\_008212 | Mus musculus L-3-hydroxyacyl-Coenzyme A dehydrogenase, short chain (Hadhsc), mRNA | 1.740 |
| BC025649 | Mus musculus fer-1-like 3, myoferlin (C. elegans), mRNA (cDNA clone IMAGE:5324940), partial cds. | 1.740 |
| AK043994 | Mus musculus 10 days neonate cortex cDNA, RIKEN full-length enriched library, clone:A830076K11 product:unknown EST, full insert sequence. | 1.740 |
| NM\_023268 | Mus musculus quiescin Q6 (Qscn6), mRNA | 1.739 |
| BC016111 | Mus musculus growth factor receptor bound protein 10, mRNA (cDNA clone MGC:28740 IMAGE:4481345), complete cds. | 1.738 |
| AK018460 | Mus musculus 16 days embryo lung cDNA, RIKEN full-length enriched library, clone:8430436F23 product:unknown EST, full insert sequence | 1.737 |
| NM\_027741 | Mus musculus Maestro (Mro), mRNA | 1.736 |
| NM\_019573 | Mus musculus WW domain-containing oxidoreductase (Wwox), mRNA | 1.735 |
| AK045923 | Mus musculus adult male corpora quadrigemina cDNA, RIKEN full-length enriched library, clone:B230323K17 product:WDC146 homolog [Homo sapiens], full insert sequence. | 1.734 |
| AK038070 | Mus musculus 16 days neonate thymus cDNA, RIKEN full-length enriched library, clone:A130075N07 product:unknown EST, full insert sequence. | 1.734 |
| NM\_013703 | Mus musculus very low density lipoprotein receptor (Vldlr), mRNA | 1.733 |
| AK009012 | Mus musculus adult male tongue cDNA, RIKEN full-length enriched library, clone:2300002L19 product:similar to CHITOTRIOSIDASE PRECURSOR [Homo sapiens], full insert sequence. | 1.731 |
| NM\_145076 | Mus musculus tripartite motif protein 24 (Trim24), mRNA | 1.730 |
| BC024804 | Mus musculus RIKEN cDNA 2210403N09 gene, mRNA (cDNA clone IMAGE:5361390), partial cds. | 1.730 |
| AK038312 | Mus musculus 16 days neonate thymus cDNA, RIKEN full-length enriched library, clone:A130095C03 product:weakly similar to 2900053G10RIK PROTEIN [Mus musculus], full insert sequence. | 1.729 |
| AK017897 | Mus musculus adult male thymus cDNA, RIKEN full-length enriched library, clone:5830405C08 product:SYNTAXIN 11 homolog [Homo sapiens], full insert sequence. | 1.729 |
| BC058962 | Mus musculus neuronal pentraxin receptor, mRNA (cDNA clone IMAGE:6826075), partial cds. | 1.729 |
| AK034147 | Mus musculus adult male diencephalon cDNA, RIKEN full-length enriched library, clone:9330159L23 product:glutamate receptor, ionotropic, kainate 2 (beta 2), full insert sequence. | 1.729 |
| AK012385 | Mus musculus 11 days embryo whole body cDNA, RIKEN full-length enriched library, clone:2700046G09 product:hypothetical protein, full insert sequence. | 1.728 |
| AK122232 | Mus musculus mRNA for mKIAA0267 protein. | 1.728 |
| NM\_025661 | Mus musculus ORM1-like 3 (S. cerevisiae) (Ormdl3), mRNA | 1.728 |
| NM\_007428 | Mus musculus angiotensinogen (Agt), mRNA | 1.727 |
| NM\_027196 | Mus musculus polymerase (DNA-directed), delta 4 (Pold4), mRNA | 1.726 |
| BY707078 | BY707078 RIKEN full-length enriched, adult male testis Mus musculus cDNA clone 1700086P04 5'. | 1.723 |
| NM\_172615 | Mus musculus RIKEN cDNA 1700021K19 gene (1700021K19Rik), mRNA | 1.722 |
| NM\_019949 | Mus musculus ubiquitin-conjugating enzyme E2L 6 (Ube2l6), mRNA | 1.722 |
| NM\_029509 | Mus musculus RIKEN cDNA 5830443L24 gene (5830443L24Rik), mRNA | 1.720 |
| NM\_025421 | Mus musculus acylphosphatase 1, erythrocyte (common) type (Acyp1), mRNA | 1.719 |
| XM\_358573 | Mus musculus LOC381422 (LOC381422), mRNA | 1.719 |
| AK079126 | Mus musculus 12 days embryo embryonic body between diaphragm region and neck cDNA, RIKEN full-length enriched library, clone:9430028P18 product:hypothetical Arginine-rich region containing protein, full insert sequence. | 1.719 |
| AK005412 | Mus musculus adult female placenta cDNA, RIKEN full-length enriched library, clone:1600010D10 product:unknown EST, full insert sequence. | 1.718 |
| NM\_022329 | Mus musculus interferon alpha responsive gene (Ifrg15), mRNA | 1.718 |
| NM\_007570 | Mus musculus B-cell translocation gene 2, anti-proliferative (Btg2), mRNA | 1.718 |
| NM\_018734 | Mus musculus guanylate nucleotide binding protein 3 (Gbp3), mRNA | 1.718 |
| NM\_013471 | Mus musculus annexin A4 (Anxa4), mRNA | 1.718 |
| AK030674 | Mus musculus 6 days neonate head cDNA, RIKEN full-length enriched library, clone:5430419M07 product:unknown EST, full insert sequence. | 1.717 |
| NM\_145519 | Mus musculus FERM, RhoGEF and pleckstrin domain protein 2 (Farp2), mRNA | 1.715 |
| 5730553K21 | 5'-AMP-ACTIVATED PROTEIN KINASE, BETA-2 SUBUNIT (AMPK BETA-2 CHAIN) homolog [Rattus norvegicus] | 1.715 |
| NM\_016898 | Mus musculus CD164 antigen (Cd164), mRNA | 1.715 |
| NM\_026145 | Mus musculus potassium channel tetramerisation domain containing 10 (Kctd10), mRNA | 1.715 |
| AK031324 | Mus musculus 13 days embryo male testis cDNA, RIKEN full-length enriched library, clone:6030407H20 product:hypothetical Yeast DNA-binding domain containing protein, full insert sequence | 1.714 |
| AK089177 | Mus musculus NOD-derived CD11c +ve dendritic cells cDNA, RIKEN full-length enriched library, clone:F630005K14 product:hypothetical protein, full insert sequence | 1.714 |
| NM\_176968 | Mus musculus RIKEN cDNA 6030401B09 gene (6030401B09Rik), mRNA | 1.707 |
| NM\_053197 | Mus musculus sideroflexin 3 (Sfxn3), mRNA | 1.707 |
| NM\_007706 | Mus musculus suppressor of cytokine signaling 2 (Socs2), mRNA | 1.706 |
| NM\_133983 | Mus musculus RIKEN cDNA 6030411F23 gene (6030411F23Rik), mRNA | 1.706 |
| ENSMUST00000050440 | Unknown | 1.706 |
| NM\_008292 | Mus musculus hydroxysteroid (17-beta) dehydrogenase 4 (Hsd17b4), mRNA | 1.706 |
| AK031967 | Mus musculus adult male medulla oblongata cDNA, RIKEN full-length enriched library, clone:6330513N08 product:hypothetical protein, full insert sequence. | 1.706 |
| NM\_153136 | Mus musculus cDNA sequence BC036718 (BC036718), mRNA | 1.705 |
| NM\_172755 | Mus musculus splicing factor, arginine/serine-rich 14 (Sfrs14), mRNA | 1.705 |
| NM\_029432 | Mus musculus RIKEN cDNA 4930402H24 gene (4930402H24Rik), mRNA | 1.702 |
| NM\_009366 | Mus musculus transforming growth factor beta 1 induced transcript 4 (Tgfb1i4), transcript variant 2, mRNA | 1.700 |
| NM\_022305 | Mus musculus UDP-Gal:betaGlcNAc beta 1,4- galactosyltransferase, polypeptide 1 (B4galt1), mRNA | 1.699 |
| AK030386 | Mus musculus adult male pituitary gland cDNA, RIKEN full-length enriched library, clone:5330405N19 product:hypothetical protein, full insert sequence. | 1.698 |
| NM\_198862 | Mus musculus neuroligin 2 (Nlgn2), mRNA | 1.698 |
| NM\_030259 | Mus musculus cDNA sequence BC003324 (BC003324), mRNA | 1.697 |
| NM\_031249 | Mus musculus cleavage stimulation factor, 3' pre-RNA subunit 2, tau (Cstf2t), mRNA | 1.697 |
| NM\_023485 | Mus musculus syncoilin (Sync), mRNA | 1.697 |
| NM\_017367 | Mus musculus cyclin I (Ccni), mRNA | 1.697 |
| NAP014889-001 | Unknown | 1.697 |
| NM\_026160 | Mus musculus microtubule-associated protein 1 light chain 3 beta (Map1lc3b), mRNA | 1.696 |
| AK028914 | Mus musculus 10 days neonate skin cDNA, RIKEN full-length enriched library, clone:4732469M24 product:amylo-1,6-glucosidase, 4-alpha-glucanotransferase, full insert sequence. | 1.696 |
| NM\_018820 | Mus musculus SERTA domain containing 1 (Sertad1), mRNA | 1.695 |
| NM\_008995 | Mus musculus peroxisome biogenesis factor 5 (Pex5), mRNA | 1.695 |
| NM\_016769 | Mus musculus MAD homolog 3 (Drosophila) (Smad3), mRNA | 1.694 |
| AK006956 | Mus musculus adult male testis cDNA, RIKEN full-length enriched library, clone:1700080G18 product:hypothetical protein, full insert sequence. | 1.694 |
| NM\_025459 | Mus musculus RIKEN cDNA 1810015C04 gene (1810015C04Rik), mRNA | 1.693 |
| NM\_144801 | Mus musculus RIKEN cDNA 2310076O21 gene (2310076O21Rik), mRNA | 1.693 |
| NM\_010142 | Mus musculus Eph receptor B2 (Ephb2), mRNA | 1.693 |
| NM\_053142 | Mus musculus protocadherin beta 17 (Pcdhb17), mRNA | 1.693 |
| AK039198 | Mus musculus adult male hypothalamus cDNA, RIKEN full-length enriched library, clone:A230106O05 product:ubiquitin-conjugating enzyme E2H, full insert sequence | 1.693 |
| BC019714 | Mus musculus RIKEN cDNA 2010209O12 gene, mRNA (cDNA clone IMAGE:3987018), partial cds. | 1.692 |
| AK020591 | Mus musculus adult male urinary bladder cDNA, RIKEN full-length enriched library, clone:9530039J15 product:unknown EST, full insert sequence. | 1.691 |
| NM\_028197 | Mus musculus RIKEN cDNA 2510003E04 gene (2510003E04Rik), mRNA | 1.690 |
| NM\_009466 | Mus musculus UDP-glucose dehydrogenase (Ugdh), mRNA | 1.690 |
| NM\_010875 | Mus musculus neural cell adhesion molecule 1 (Ncam1), mRNA | 1.690 |
| AI467211 | vd74h04.x1 Beddington mouse embryonic region Mus musculus cDNA clone IMAGE:806359 3'. | 1.689 |
| NM\_013888 | Mus musculus DnaJ (Hsp40) homolog, subfamily C, member 12 (Dnajc12), mRNA | 1.689 |
| NM\_172777 | Mus musculus hypothetical protein E430029F06 (E430029F06), mRNA | 1.688 |
| NM\_178787 | Mus musculus RIKEN cDNA A930037G23 gene (A930037G23Rik), mRNA | 1.688 |
| AK004434 | Mus musculus 18-day embryo whole body cDNA, RIKEN full-length enriched library, clone:1190001L17 product:unknown EST, full insert sequence. | 1.688 |
| BC037677 | Mus musculus RIKEN cDNA 1110038G02 gene, mRNA (cDNA clone MGC:46974 IMAGE:3491666), complete cds. | 1.688 |
| NM\_172525 | Mus musculus RIKEN cDNA B130017I01 gene (B130017I01Rik), mRNA | 1.687 |
| NM\_198864 | Mus musculus SLIT and NTRK-like family, member 3 (Slitrk3), mRNA | 1.687 |
| BC034071 | Mus musculus, clone IMAGE:4218313, mRNA, partial cds. | 1.686 |
| NM\_198411 | Mus musculus RIKEN cDNA 2610204M08 gene (2610204M08Rik), mRNA | 1.686 |
| NM\_009149 | Mus musculus golgi apparatus protein 1 (Glg1), mRNA | 1.685 |
| NM\_025944 | Mus musculus RIKEN cDNA 2810432L12 gene (2810432L12Rik), mRNA | 1.685 |
| NM\_007913 | Mus musculus early growth response 1 (Egr1), mRNA | 1.685 |
| NM\_016759 | Mus musculus Rap2 interacting protein (Rap2ip), mRNA | 1.685 |
| NAP122799-1 | Unknown | 1.684 |
| BC055394 | Mus musculus RIKEN cDNA 1810064L21 gene, mRNA (cDNA clone MGC:67516 IMAGE:5709667), complete cds. | 1.683 |
| BC046229 | Mus musculus RIKEN cDNA 2010305K11 gene, mRNA (cDNA clone IMAGE:5721302), partial cds. | 1.683 |
| NM\_018761 | Mus musculus catenin alpha-like 1 (Catnal1), mRNA | 1.682 |
| NM\_009510 | Mus musculus villin 2 (Vil2), mRNA | 1.682 |
| AK076529 | Mus musculus 0 day neonate head cDNA, RIKEN full-length enriched library, clone:4833442H12 product:hypothetical Leucine-rich repeat containing protein, full insert sequence. | 1.681 |
| AK020698 | Mus musculus 6 days neonate skin cDNA, RIKEN full-length enriched library, clone:A030006P16 product:similar to KERATIN ASSOCIATED PROTEIN 16.1 [Homo sapiens], full insert sequence. | 1.681 |
| NM\_007862 | Mus musculus discs, large homolog 1 (Drosophila) (Dlgh1), mRNA | 1.681 |
| NM\_009790 | Mus musculus calmodulin 1 (Calm1), mRNA | 1.680 |
| NM\_145837 | Mus musculus interleukin 17D (Il17d), mRNA | 1.680 |
| AK081382 | Mus musculus 16 days embryo head cDNA, RIKEN full-length enriched library, clone:C130012K10 product:hypothetical Serine-rich region containing protein, full insert sequence. | 1.679 |
| NM\_010570 | Mus musculus insulin receptor substrate 1 (Irs1), mRNA | 1.678 |
| NM\_138309 | Mus musculus MIC2 (monoclonal Imperial Cancer Research Fund 2)-like 1 (Mic2l1), mRNA | 1.678 |
| NM\_144500 | Mus musculus oxysterol binding protein-like 2 (Osbpl2), mRNA | 1.678 |
| NM\_019709 | Mus musculus membrane-bound transcription factor protease, site 1 (Mbtps1), mRNA | 1.678 |
| NM\_010612 | Mus musculus kinase insert domain protein receptor (Kdr), mRNA | 1.678 |
| NM\_175387 | Mus musculus RNA binding motif protein 9 (Rbm9), mRNA | 1.676 |
| BC029161 | Mus musculus cDNA clone IMAGE:4952261, partial cds. | 1.676 |
| AK008529 | Mus musculus adult male small intestine cDNA, RIKEN full-length enriched library, clone:2010305K11 product:hypothetical protein, full insert sequence. | 1.675 |
| AK080318 | Mus musculus 3 days neonate thymus cDNA, RIKEN full-length enriched library, clone:A630052N20 product:weakly similar to SIMILAR TO FORMIN-LIKE (FRAGMENT) [Homo sapiens], full insert sequence. | 1.675 |
| NM\_025988 | Mus musculus acyl-Coenzyme A binding domain containing 4 (Acbd4), mRNA | 1.675 |
| NM\_011857 | Mus musculus odd Oz/ten-m homolog 3 (Drosophila) (Odz3), mRNA | 1.674 |
| BU841889 | AGENCOURT\_10119157 NIH\_MGC\_144 Mus musculus cDNA clone IMAGE:6535561 5', mRNA sequence | 1.673 |
| NM\_009073 | Mus musculus rod outer segment membrane protein 1 (Rom1), mRNA | 1.673 |
| AK122419 | Mus musculus mRNA for mKIAA1025 protein. | 1.672 |
| AF148511 | Mus musculus hermes mRNA, complete cds. | 1.671 |
| NM\_026787 | Mus musculus RIKEN cDNA 1110012L19 gene (1110012L19Rik), mRNA | 1.671 |
| BC068142 | Mus musculus RIKEN cDNA 8430403M15 gene, mRNA (cDNA clone IMAGE:30536534), partial cds | 1.671 |
| NM\_173442 | Mus musculus glucosaminyl (N-acetyl) transferase 1, core 2 (Gcnt1), mRNA | 1.670 |
| AK034202 | Mus musculus adult male diencephalon cDNA, RIKEN full-length enriched library, clone:9330162P19 product:unknown EST, full insert sequence | 1.669 |
| NAP002638-001 | Unknown | 1.669 |
| NM\_134099 | Mus musculus F-box only protein 4 (Fbxo4), mRNA | 1.669 |
| NM\_026967 | Mus musculus Ras homolog enriched in brain like 1 (Rhebl1), mRNA | 1.669 |
| NM\_009675 | Mus musculus amine oxidase, copper containing 3 (Aoc3), mRNA | 1.669 |
| NM\_183251 | Mus musculus RIKEN cDNA 1810020D17 gene (1810020D17Rik), mRNA | 1.668 |
| NM\_027334 | Mus musculus RIKEN cDNA 3300001H21 gene (3300001H21Rik), mRNA | 1.667 |
| NM\_026797 | Mus musculus DNA segment, Chr 2, Brigham & Women's Genetics 0891 expressed (D2Bwg0891e), mRNA | 1.666 |
| NM\_028883 | Mus musculus RIKEN cDNA 4632415K11 gene (4632415K11Rik), mRNA | 1.666 |
| NM\_009686 | Mus musculus amyloid beta (A4) precursor protein-binding, family B, member 2 (Apbb2), mRNA | 1.665 |
| NM\_010162 | Mus musculus exostoses (multiple) 1 (Ext1), mRNA | 1.664 |
| NM\_013863 | Mus musculus Bcl2-associated athanogene 3 (Bag3), mRNA | 1.664 |
| NM\_019739 | Mus musculus forkhead box O1 (Foxo1), mRNA | 1.664 |
| NM\_173350 | Mus musculus oxysterol binding protein-like 9 (Osbpl9), transcript variant 2, mRNA | 1.663 |
| NM\_010498 | Mus musculus iduronate 2-sulfatase (Ids), mRNA | 1.662 |
| NM\_010200 | Mus musculus fibroblast growth factor 13 (Fgf13), mRNA | 1.661 |
| BC006717 | Mus musculus DNA segment, Chr 9, ERATO Doi 392, expressed, mRNA (cDNA clone MGC:12151 IMAGE:3711012), complete cds. | 1.661 |
| NM\_008394 | Mus musculus interferon dependent positive acting transcription factor 3 gamma (Isgf3g), mRNA | 1.661 |
| AK032356 | Mus musculus adult male olfactory brain cDNA, RIKEN full-length enriched library, clone:6430526E05 product:CORTACTIN-BINDING PROTEIN 2 homolog [Homo sapiens], full insert sequence. | 1.660 |
| NM\_026058 | Mus musculus longevity assurance homolog 4 (S. cerevisiae) (Lass4), mRNA | 1.660 |
| NM\_011182 | Mus musculus pleckstrin homology, Sec7 and coiled-coil domains 3 (Pscd3), mRNA | 1.660 |
| Y18276 | Mus musculus mRNA for neurobeachin. | 1.660 |
| NM\_053256 | Mus musculus tuberoinfundibular peptide of 39 residues (TIP39) preprohormone (Tifp39), mRNA | 1.659 |
| AK047055 | Mus musculus 10 days neonate cerebellum cDNA, RIKEN full-length enriched library, clone:B930014F08 product:unclassifiable, full insert sequence | 1.658 |
| NM\_017366 | Mus musculus acyl-Coenzyme A dehydrogenase, very long chain (Acadvl), mRNA | 1.657 |
| BC024687 | Mus musculus vang, van gogh-like 1 (Drosophila), mRNA (cDNA clone IMAGE:3669388), partial cds. | 1.656 |
| NM\_133218 | Mus musculus glucocorticoid induced gene 1 (Gig1), mRNA | 1.656 |
| 5730414I01 | unknown EST | 1.655 |
| NM\_144534 | Mus musculus RIKEN cDNA 1110001E17 gene (1110001E17Rik), mRNA | 1.655 |
| NM\_011082 | Mus musculus polymeric immunoglobulin receptor (Pigr), mRNA | 1.654 |
| NM\_080556 | Mus musculus transmembrane 9 superfamily member 2 (Tm9sf2), mRNA | 1.654 |
| NM\_175930 | Mus musculus Rap guanine nucleotide exchange factor (GEF) 5 (Rapgef5), mRNA | 1.653 |
| NM\_015797 | Mus musculus F-box only protein 6b (Fbxo6b), mRNA | 1.652 |
| NM\_010581 | Mus musculus CD47 antigen (Rh-related antigen, integrin-associated signal transducer) (Cd47), mRNA | 1.652 |
| NM\_016763 | Mus musculus hydroxyacyl-Coenzyme A dehydrogenase type II (Hadh2), mRNA | 1.651 |
| NM\_021292 | Mus musculus Ellis van Creveld gene homolog (human) (Evc), mRNA | 1.650 |
| NM\_026441 | Mus musculus RIKEN cDNA 2600002E23 gene (2600002E23Rik), mRNA | 1.650 |
| AK046628 | Mus musculus 4 days neonate male adipose cDNA, RIKEN full-length enriched library, clone:B430212B02 product:METASTASIS SUPPRESSOR PROTEIN homolog [Homo sapiens], full insert sequence | 1.650 |
| NM\_010397 | Mus musculus histocompatibility 2, T region locus 22 (H2-T22), mRNA | 1.650 |
| AK129178 | Mus musculus mRNA for mKIAA0629 protein. | 1.649 |
| XM\_145511 | Mus musculus similar to heat shock 20-kDa protein (LOC243912), mRNA | 1.649 |
| AK044446 | Mus musculus adult retina cDNA, RIKEN full-length enriched library, clone:A930013L13 product:HUNTINGTIN INTERACTING PROTEIN HYPE (FRAGMENT) homolog [Homo sapiens], full insert sequence. | 1.648 |
| AK052077 | Mus musculus 12 days embryo eyeball cDNA, RIKEN full-length enriched library, clone:D230043L20 product:similar to NT2RP4001730 PROTEIN (FRAGMENT) [Homo sapiens], full insert sequence. | 1.648 |
| BC017623 | Mus musculus expressed sequence AI481716, mRNA (cDNA clone IMAGE:4487867), partial cds. | 1.648 |
| AK122295 | Mus musculus mRNA for mKIAA0483 protein. | 1.647 |
| NM\_144551 | Mus musculus tribbles homolog 2 (Drosophila) (Trib2), mRNA | 1.647 |
| NM\_019833 | Mus musculus RIKEN cDNA B230317C12 gene (B230317C12Rik), mRNA | 1.647 |
| BC042507 | Mus musculus RIKEN cDNA 2410066E13 gene, mRNA (cDNA clone MGC:30499 IMAGE:4235651), complete cds. | 1.646 |
| AK078252 | Mus musculus adult male olfactory brain cDNA, RIKEN full-length enriched library, clone:6430540O04 product:unclassifiable, full insert sequence. | 1.645 |
| NM\_008633 | Mus musculus microtubule-associated protein 4 (Mtap4), mRNA | 1.645 |
| NM\_008580 | Mus musculus mitogen activated protein kinase kinase kinase 5 (Map3k5), mRNA | 1.643 |
| NM\_145220 | Mus musculus Dip3 beta (Dip3b), mRNA | 1.643 |
| BC027382 | Mus musculus cDNA sequence BC027382, mRNA (cDNA clone IMAGE:4948091), partial cds. | 1.643 |
| NM\_007760 | Mus musculus carnitine acetyltransferase (Crat), mRNA | 1.639 |
| AK036277 | Mus musculus 16 days neonate cerebellum cDNA, RIKEN full-length enriched library, clone:9630053D03 product:N-myc downstream regulated 3, full insert sequence. | 1.639 |
| NM\_144529 | Mus musculus Rho GTPase activating protein 17 (Arhgap17), mRNA | 1.638 |
| NM\_025858 | Mus musculus scotin gene (Scotin), mRNA | 1.637 |
| AK122299 | Mus musculus mRNA for mKIAA0513 protein. | 1.637 |
| NM\_177545 | Mus musculus vang, van gogh-like 1 (Drosophila) (Vangl1), mRNA | 1.637 |
| NM\_007537 | Mus musculus Bcl2-like 2 (Bcl2l2), mRNA | 1.636 |
| TC1029420 | ATXX\_MOUSE Ataxin-10 (Spinocerebellar ataxia type 10 protein homolog) (Brain protein E46). [Mouse] {Mus musculus}, partial (27%) | 1.636 |
| NM\_024477 | Mus musculus expressed sequence AI428795 (AI428795), mRNA | 1.636 |
| NM\_133745 | Mus musculus DNA segment, Chr 10, Brigham & Women's Genetics 0791 expressed (D10Bwg0791e), mRNA | 1.635 |
| NM\_011191 | Mus musculus protease (prosome, macropain) 28 subunit, beta, b (Psme2b), mRNA | 1.635 |
| NM\_008165 | Mus musculus glutamate receptor, ionotropic, AMPA1 (alpha 1) (Gria1), mRNA | 1.634 |
| NM\_013674 | Mus musculus interferon regulatory factor 4 (Irf4), mRNA | 1.634 |
| NM\_145135 | Mus musculus ribonuclease/angiogenin inhibitor 1 (Rnh1), mRNA | 1.633 |
| AK015348 | Mus musculus adult male testis cDNA, RIKEN full-length enriched library, clone:4930440H19 product:hypothetical Ubiquitin-conjugating enzymes containing protein, full insert sequence. | 1.633 |
| NM\_015772 | Mus musculus sal-like 2 (Drosophila) (Sall2), mRNA | 1.632 |
| AK048044 | Mus musculus 16 days embryo head cDNA, RIKEN full-length enriched library, clone:C130031H07 product:cell division cycle 37 homolog (S. cerevisiae)-like, full insert sequence. | 1.632 |
| NM\_172443 | Mus musculus cDNA sequence BC026530 (BC026530), mRNA | 1.632 |
| NM\_016794 | Mus musculus vesicle-associated membrane protein 8 (Vamp8), mRNA | 1.631 |
| NM\_177152 | Mus musculus RIKEN cDNA 9430095K15 gene (9430095K15Rik), mRNA | 1.631 |
| NM\_026942 | Mus musculus stomatin-like 1 (Stoml1), mRNA | 1.629 |
| A\_51\_P301713 | Unknown | 1.629 |
| NM\_010470 | Mus musculus heterochromatin protein 1, binding protein 3 (Hp1bp3), mRNA | 1.628 |
| NM\_133962 | Mus musculus rho/rac guanine nucleotide exchange factor (GEF) 18 (Arhgef18), mRNA | 1.626 |
| NM\_016969 | Mus musculus myeloid-associated differentiation marker (Myadm), mRNA | 1.625 |
| NM\_007870 | Mus musculus deoxyribonuclease 1-like 3 (Dnase1l3), mRNA | 1.625 |
| NM\_008846 | Mus musculus phosphatidylinositol-4-phosphate 5-kinase, type 1 alpha (Pip5k1a), mRNA | 1.625 |
| NM\_008983 | Mus musculus protein tyrosine phosphatase, receptor type, K (Ptprk), mRNA | 1.625 |
| BC049279 | Mus musculus v-erb-b2 erythroblastic leukemia viral oncogene homolog 3 (avian), mRNA (cDNA clone IMAGE:4460595), partial cds. | 1.624 |
| AK007954 | Mus musculus 10 day old male pancreas cDNA, RIKEN full-length enriched library, clone:1810064L21 product:UDP-GLUCOSE:GLYCOPROTEIN GLUCOSYLTRANSFERASE 2 PRECURSOR homolog [Homo sapiens], full insert sequence. | 1.623 |
| AK049856 | Mus musculus adult male hippocampus cDNA, RIKEN full-length enriched library, clone:C630004B07 product:solute carrier family 11 (proton-coupled divalent metal ion transporters), member 2, full insert sequence. | 1.623 |
| AK129247 | Mus musculus mRNA for mKIAA0953 protein. | 1.622 |
| NM\_028053 | Mus musculus DNA segment, Chr 4, ERATO Doi 89, expressed (D4Ertd89e), mRNA | 1.622 |
| NM\_053194 | Mus musculus expressed sequence AI114950 (AI114950), mRNA | 1.622 |
| NM\_025823 | Mus musculus prenylcysteine oxidase 1 (Pcyox1), mRNA | 1.621 |
| NM\_181072 | Mus musculus myosin IE (Myo1e), mRNA | 1.621 |
| AK129273 | Mus musculus mRNA for mKIAA1055 protein. | 1.620 |
| NM\_144797 | Mus musculus cDNA sequence BC019776 (BC019776), mRNA | 1.620 |
| BC058264 | Mus musculus serologically defined colon cancer antigen 33, mRNA (cDNA clone MGC:65389 IMAGE:6488400), complete cds. | 1.618 |
| BC023202 | Mus musculus cDNA clone IMAGE:3986405 | 1.618 |
| BC006733 | Mus musculus cDNA sequence BC037006, mRNA (cDNA clone IMAGE:3963643), partial cds. | 1.618 |
| NM\_008917 | Mus musculus palmitoyl-protein thioesterase 1 (Ppt1), mRNA | 1.617 |
| NM\_177359 | Mus musculus RIKEN cDNA 6030490I01 gene (6030490I01Rik), mRNA | 1.617 |
| NM\_030714 | Mus musculus deltex 3 homolog (Drosophila) (Dtx3), mRNA | 1.616 |
| NM\_016677 | Mus musculus hippocalcin-like 1 (Hpcal1), mRNA | 1.616 |
| AK028992 | Mus musculus 10 days neonate skin cDNA, RIKEN full-length enriched library, clone:4732479G04 product:unknown EST, full insert sequence | 1.616 |
| NM\_008055 | Mus musculus frizzled homolog 4 (Drosophila) (Fzd4), mRNA | 1.615 |
| BC002083 | Mus musculus FBJ osteosarcoma oncogene B, mRNA (cDNA clone IMAGE:3484944), partial cds. | 1.614 |
| AK002861 | Mus musculus adult male kidney cDNA, RIKEN full-length enriched library, clone:0610040B10 product:unknown EST, full insert sequence. | 1.614 |
| NM\_033476 | Mus musculus transcription factor CP2 (Tcfcp2), mRNA | 1.614 |
| NM\_133766 | Mus musculus RIKEN cDNA C920006C10 gene (C920006C10Rik), mRNA | 1.613 |
| BC030872 | Mus musculus attractin like protein, mRNA (cDNA clone IMAGE:4221668), partial cds. | 1.613 |
| NM\_009789 | Mus musculus calbindin 3, (vitamin D-dependent calcium binding protein) (Calb3), mRNA | 1.612 |
| TC973638 | BC018534 protective protein for beta-galactosidase {Mus musculus}, partial (31%) | 1.611 |
| AK017516 | Mus musculus 8 days embryo whole body cDNA, RIKEN full-length enriched library, clone:5730407K14 product:hypothetical protein, full insert sequence. | 1.611 |
| NM\_133201 | Mus musculus mitofusin 2 (Mfn2), mRNA | 1.611 |
| NM\_008413 | Mus musculus Janus kinase 2 (Jak2), mRNA | 1.610 |
| NM\_009963 | Mus musculus cryptochrome 2 (photolyase-like) (Cry2), mRNA | 1.610 |
| NM\_009730 | Mus musculus attractin (Atrn), mRNA | 1.609 |
| NM\_134257 | Mus musculus regulator of G-protein signaling 3 (Rgs3), mRNA | 1.608 |
| NM\_023063 | Mus musculus DNA segment, Chr 15, ERATO Doi 366, expressed (D15Ertd366e), mRNA | 1.608 |
| AK009454 | Mus musculus adult male tongue cDNA, RIKEN full-length enriched library, clone:2310021P13 product:hypothetical protein, full insert sequence. | 1.608 |
| NM\_207237 | Mus musculus mannosidase, alpha, class 1C, member 1 (Man1c1), mRNA | 1.608 |
| AK019082 | Mus musculus adult male tongue cDNA, RIKEN full-length enriched library, clone:2310010J17 product:unknown EST, full insert sequence. | 1.607 |
| NM\_144794 | Mus musculus cDNA sequence BC014795 (BC014795), mRNA | 1.605 |
| NM\_146230 | Mus musculus 3-ketoacyl-CoA thiolase B (MGC29978), mRNA | 1.605 |
| NM\_148925 | Mus musculus FYVE and coiled-coil domain containing 1 (Fyco1), mRNA | 1.605 |
| NM\_024452 | Mus musculus leucine zipper protein 1 (Luzp1), mRNA | 1.605 |
| NM\_172588 | Mus musculus RIKEN cDNA A130038L21 gene (A130038L21Rik), mRNA | 1.604 |
| BC021818 | Mus musculus helicase with zinc finger domain, mRNA (cDNA clone IMAGE:4007187), partial cds. | 1.604 |
| NM\_019831 | Mus musculus zinc finger protein 261 (Zfp261), mRNA | 1.603 |
| AK122509 | Mus musculus mRNA for mKIAA1429 protein. | 1.602 |
| NM\_024289 | Mus musculus oxysterol binding protein-like 5 (Osbpl5), mRNA | 1.602 |
| NM\_172546 | Mus musculus membrane associated guanylate kinase interacting protein-like 1 (Magi1), mRNA | 1.601 |
| BC025071 | Mus musculus DNA segment, Chr 8, ERATO Doi 325, expressed, mRNA (cDNA clone MGC:36794 IMAGE:3498003), complete cds. | 1.600 |
| NM\_176845 | Mus musculus RIKEN cDNA 4921528E07 gene (4921528E07Rik), mRNA | 1.600 |
| NM\_133349 | Mus musculus expressed sequence AA407930 (AA407930), mRNA | 1.599 |
| AK129255 | Mus musculus mRNA for mKIAA0990 protein. | 1.598 |
| NM\_025482 | Mus musculus tumor protein D52-like 2 (Tpd52l2), mRNA | 1.598 |
| X86010 | M.musculus mRNA for rev-erbA alpha, orphan nuclear receptor. | 1.598 |
| AK085821 | Mus musculus 16 days neonate heart cDNA, RIKEN full-length enriched library, clone:D830014B20 product:hypothetical protein, full insert sequence. | 1.598 |
| NM\_028310 | Mus musculus RIKEN cDNA 2810006K23 gene (2810006K23Rik), mRNA | 1.597 |
| AK122501 | Mus musculus mRNA for mKIAA1389 protein. | 1.597 |
| NM\_011030 | Mus musculus procollagen-proline, 2-oxoglutarate 4-dioxygenase (proline 4-hydroxylase), alpha 1 polypeptide (P4ha1), mRNA | 1.596 |
| NM\_199011 | Mus musculus diacylglycerol kinase, theta (Dgkq), mRNA | 1.596 |
| BC051391 | Mus musculus myosin IE, mRNA (cDNA clone MGC:58876 IMAGE:5053090), complete cds. | 1.595 |
| NM\_178066 | Mus musculus RIKEN cDNA 1110012D08 gene (1110012D08Rik), mRNA | 1.595 |
| AK041011 | Mus musculus adult male aorta and vein cDNA, RIKEN full-length enriched library, clone:A530060J16 product:hypothetical protein, full insert sequence. | 1.595 |
| NM\_008090 | Mus musculus GATA binding protein 2 (Gata2), mRNA | 1.595 |
| NM\_013457 | Mus musculus adducin 1 (alpha) (Add1), mRNA | 1.595 |
| NM\_145934 | Mus musculus expressed sequence AW049765 (AW049765), mRNA | 1.594 |
| NM\_009900 | Mus musculus chloride channel 2 (Clcn2), mRNA | 1.594 |
| NM\_144880 | Mus musculus protein phosphatase 2, regulatory subunit B (B56), alpha isoform (Ppp2r5a), mRNA | 1.594 |
| NM\_025590 | Mus musculus thioesterase, adipose associated (Thea), mRNA | 1.593 |
| NM\_023824 | Mus musculus RIKEN cDNA 1500004C10 gene (1500004C10Rik), mRNA | 1.593 |
| NM\_029094 | Mus musculus phosphatidylinositol 3-kinase, catalytic, beta polypeptide (Pik3cb), mRNA | 1.592 |
| NM\_009933 | Mus musculus procollagen, type VI, alpha 1 (Col6a1), mRNA | 1.592 |
| AK090104 | Mus musculus 1.5 years female mammary gland CRL-2116 JC cDNA, RIKEN full-length enriched library, clone:G430127E12 product:weakly similar to RSEC15 [Rattus norvegicus], full insert sequence. | 1.592 |
| NM\_009176 | Mus musculus sialyltransferase 6 (N-acetyllacosaminide alpha 2,3-sialyltransferase) (Siat6), mRNA | 1.591 |
| TC984338 | BC002094 Creb3 protein {Mus musculus}, complete | 1.590 |
| AB093227 | Mus musculus mRNA for mKIAA0312 protein. | 1.590 |
| NM\_023160 | Mus musculus camello-like 1 (Cml1), mRNA | 1.589 |
| NM\_009277 | Mus musculus tripartite motif protein 21 (Trim21), mRNA | 1.589 |
| AK005758 | Mus musculus adult male testis cDNA, RIKEN full-length enriched library, clone:1700008D07 product:hypothetical Leucine-rich repeat containing protein, full insert sequence. | 1.589 |
| AK007164 | Mus musculus adult male testis cDNA, RIKEN full-length enriched library, clone:1700110M21 product:hypothetical protein, full insert sequence. | 1.588 |
| NM\_028007 | Mus musculus DNA segment, Chr 8, Wayne State University 49, expressed (D8Wsu49e), mRNA | 1.588 |
| NM\_010860 | Mus musculus myosin, light polypeptide 6, alkali, smooth muscle and non-muscle (Myl6), mRNA | 1.588 |
| AK075794 | Mus musculus 10 day old male pancreas cDNA, RIKEN full-length enriched library, clone:1810043G02 product:transient receptor protein 7, full insert sequence. | 1.588 |
| NM\_053015 | Mus musculus melanophilin (Mlph), mRNA | 1.587 |
| AK086993 | Mus musculus 0 day neonate lung cDNA, RIKEN full-length enriched library, clone:E030018N11 product:hypothetical protein, full insert sequence | 1.587 |
| AK129163 | Mus musculus mRNA for mKIAA0562 protein. | 1.587 |
| NM\_177672 | Mus musculus expressed sequence AV028368 (AV028368), mRNA | 1.586 |
| BC025076 | Mus musculus cDNA sequence BC025076, mRNA (cDNA clone MGC:36859 IMAGE:4459181), complete cds. | 1.586 |
| NM\_025703 | Mus musculus RIKEN cDNA 3930402F23 gene (3930402F23Rik), mRNA | 1.584 |
| AK014716 | Mus musculus 0 day neonate head cDNA, RIKEN full-length enriched library, clone:4833417J20 product:unknown EST, full insert sequence | 1.583 |
| D10920 | Mus musculus NEDD-10 mRNA, partial sequence. | 1.583 |
| AK029925 | Mus musculus adult male testis cDNA, RIKEN full-length enriched library, clone:4932405E23 product:hypothetical Glycoside hydrolase family 35 containing protein, full insert sequence. | 1.582 |
| AK017277 | Mus musculus 6 days neonate head cDNA, RIKEN full-length enriched library, clone:5430405N12 product:unclassifiable, full insert sequence. | 1.582 |
| AK081290 | Mus musculus adult male corpus striatum cDNA, RIKEN full-length enriched library, clone:C030047F10 product:unknown EST, full insert sequence | 1.582 |
| NM\_026582 | Mus musculus RIKEN cDNA 5031439A09 gene (5031439A09Rik), mRNA | 1.582 |
| NM\_011952 | Mus musculus mitogen activated protein kinase 3 (Mapk3), mRNA | 1.581 |
| NM\_020266 | Mus musculus DnaJ (Hsp40) homolog, subfamily B, member 10 (Dnajb10), mRNA | 1.581 |
| BC058972 | Mus musculus RIKEN cDNA 5330439J01 gene, mRNA (cDNA clone MGC:67221 IMAGE:5698469), complete cds. | 1.581 |
| NM\_010273 | Mus musculus guanosine diphosphate (GDP) dissociation inhibitor 1 (Gdi1), mRNA | 1.579 |
| NM\_023224 | Mus musculus Casitas B-lineage lymphoma c (Cblc), mRNA | 1.579 |
| NM\_023781 | Mus musculus RIKEN cDNA 1700020D05 gene (1700020D05Rik), mRNA | 1.578 |
| BC039571 | Mus musculus RIKEN cDNA 3300001A09 gene, mRNA (cDNA clone MGC:48200 IMAGE:1515262), complete cds. | 1.578 |
| NM\_010747 | Mus musculus Yamaguchi sarcoma viral (v-yes-1) oncogene homolog (Lyn), mRNA | 1.578 |
| NM\_019972 | Mus musculus sortilin 1 (Sort1), mRNA | 1.578 |
| AK122302 | Mus musculus mRNA for mKIAA0532 protein. | 1.578 |
| AK014507 | Mus musculus 0 day neonate skin cDNA, RIKEN full-length enriched library, clone:4631402G10 product:CYCLIN-BOX CARRYING PROTEIN homolog [Homo sapiens], full insert sequence. | 1.578 |
| NM\_028166 | Mus musculus RIKEN cDNA 1600014C10 gene (1600014C10Rik), mRNA | 1.577 |
| NM\_025516 | Mus musculus serologically defined breast cancer antigen 84 (Sdbcag84), mRNA | 1.575 |
| NM\_173751 | Mus musculus ilvB (bacterial acetolactate synthase)-like (Ilvbl), mRNA | 1.575 |
| BM932442 | UI-M-BH2.1-apb-a-07-0-UI.r1 NIH\_BMAP\_M\_S3.1 Mus musculus cDNA clone UI-M-BH2.1-apb-a-07-0-UI 5', mRNA sequence | 1.574 |
| NM\_025311 | Mus musculus DNA segment, Chr 14, ERATO Doi 449, expressed (D14Ertd449e), mRNA | 1.573 |
| NM\_145476 | Mus musculus DNA segment, Chr 15, ERATO Doi 781, expressed (D15Ertd781e), mRNA | 1.573 |
| TC1080318 | Unknown | 1.573 |
| AK011539 | Mus musculus 10 days embryo whole body cDNA, RIKEN full-length enriched library, clone:2610024M03 product:unknown EST, full insert sequence. | 1.573 |
| BC049680 | Mus musculus RIKEN cDNA 1110001A23 gene, mRNA (cDNA clone MGC:58544 IMAGE:6596401), complete cds. | 1.573 |
| NM\_172631 | Mus musculus DNA segment, Chr 18, ERATO Doi 653, expressed (D18Ertd653e), mRNA | 1.573 |
| NM\_011530 | Mus musculus transporter 2, ATP-binding cassette, sub-family B (MDR/TAP) (Tap2), mRNA | 1.572 |
| NM\_013885 | Mus musculus chloride intracellular channel 4 (mitochondrial) (Clic4), mRNA | 1.572 |
| NM\_009283 | Mus musculus signal transducer and activator of transcription 1 (Stat1), mRNA | 1.571 |
| NM\_026667 | Mus musculus RIKEN cDNA 9130005N14 gene (9130005N14Rik), mRNA | 1.571 |
| AK005166 | Mus musculus adult male cerebellum cDNA, RIKEN full-length enriched library, clone:1500005N04 product:PIUS homolog [Rattus norvegicus], full insert sequence. | 1.570 |
| NM\_178665 | Mus musculus LIM domain containing preferred translocation partner in lipoma (Lpp), mRNA | 1.570 |
| NM\_010518 | Mus musculus insulin-like growth factor binding protein 5 (Igfbp5), mRNA | 1.570 |
| AK009249 | Mus musculus adult male tongue cDNA, RIKEN full-length enriched library, clone:2310009E04 product:WEAKLY SIMILAR TO L-RIBULOKINASE homolog [Homo sapiens], full insert sequence. | 1.569 |
| NM\_198625 | Mus musculus cDNA sequence BC060632 (BC060632), mRNA | 1.569 |
| NM\_178598 | Mus musculus transgelin 2 (Tagln2), mRNA | 1.569 |
| NM\_147219 | Mus musculus ATP-binding cassette, sub-family A (ABC1), member 5 (Abca5), mRNA | 1.568 |
| NM\_008686 | Mus musculus nuclear factor, erythroid derived 2,-like 1 (Nfe2l1), mRNA | 1.567 |
| NM\_181348 | Mus musculus RIKEN cDNA A230083H22 gene (A230083H22Rik), mRNA | 1.566 |
| NM\_181516 | Mus musculus tafazzin (Taz), mRNA | 1.565 |
| NM\_017375 | Mus musculus osteoclast stimulating factor 1 (Ostf1), mRNA | 1.565 |
| AK048138 | Mus musculus 16 days embryo head cDNA, RIKEN full-length enriched library, clone:C130036O19 product:DJ5O6.2 (NOVEL PROTEIN SIMILAR TO C. ELEGANS F40E10.6 (ISOFORM 1)) (FRAGMENT) homolog [Homo sapiens], full insert sequence. | 1.565 |
| NM\_178653 | Mus musculus RIKEN cDNA C330023F11 gene (C330023F11Rik), mRNA | 1.565 |
| NM\_011962 | Mus musculus procollagen-lysine, 2-oxoglutarate 5-dioxygenase 3 (Plod3), mRNA | 1.565 |
| NM\_011327 | Mus musculus sterol carrier protein 2, liver (Scp2), mRNA | 1.564 |
| NM\_145486 | Mus musculus RIKEN cDNA 9530046H09 gene (9530046H09Rik), mRNA | 1.564 |
| NM\_026859 | Mus musculus MAF1 homolog (yeast) (Maf1), mRNA | 1.563 |
| NM\_027188 | Mus musculus SET and MYND domain containing 3 (Smyd3), mRNA | 1.563 |
| NM\_010271 | Mus musculus glycerol-3-phosphate dehydrogenase 1 (soluble) (Gpd1), mRNA | 1.562 |
| NM\_176948 | Mus musculus RIKEN cDNA 9330133O14 gene (9330133O14Rik), mRNA | 1.560 |
| NM\_009444 | Mus musculus trans-golgi network protein 2 (Tgoln2), mRNA | 1.559 |
| AK089654 | Mus musculus activated spleen cDNA, RIKEN full-length enriched library, clone:F830007D21 product:PROTEIN KINASE NYD-SP9 homolog [Homo sapiens], full insert sequence. | 1.559 |
| NM\_030750 | Mus musculus sphingosine-1-phosphate phosphatase 1 (Sgpp1), mRNA | 1.559 |
| NM\_053246 | Mus musculus docking protein 4 (Dok4), mRNA | 1.559 |
| NM\_026213 | Mus musculus RIKEN cDNA 2900001O04 gene (2900001O04Rik), mRNA | 1.557 |
| NM\_178098 | Mus musculus RIKEN cDNA 4930486L24 gene (4930486L24Rik), mRNA | 1.557 |
| NM\_009397 | Mus musculus tumor necrosis factor, alpha-induced protein 3 (Tnfaip3), mRNA | 1.557 |
| NM\_175529 | Mus musculus RIKEN cDNA F630035L11 gene (F630035L11Rik), mRNA | 1.557 |
| NM\_029868 | Mus musculus RIKEN cDNA 5330440M15 gene (5330440M15Rik), mRNA | 1.556 |
| NM\_021329 | Mus musculus RAN guanine nucleotide release factor (Rangnrf), mRNA | 1.556 |
| BC067008 | Mus musculus cDNA clone MGC:93029 IMAGE:6418163, complete cds. | 1.555 |
| AK013363 | Mus musculus 10, 11 days embryo whole body cDNA, RIKEN full-length enriched library, clone:2810457N15 product:unknown EST, full insert sequence. | 1.555 |
| NM\_173378 | Mus musculus transformation related protein 53 binding protein 2 (Trp53bp2), mRNA | 1.555 |
| NM\_009713 | Mus musculus arylsulfatase A (Arsa), mRNA | 1.553 |
| AK035296 | Mus musculus adult male urinary bladder cDNA, RIKEN full-length enriched library, clone:9530011J05 product:hypothetical ZIP Zinc transporter containing protein, full insert sequence. | 1.553 |
| NM\_026505 | Mus musculus BMP and activin membrane-bound inhibitor, homolog (Xenopus laevis) (Bambi), mRNA | 1.553 |
| NM\_028527 | Mus musculus RIKEN cDNA 1700047I17 gene (1700047I17Rik), mRNA | 1.552 |
| NM\_010241 | Mus musculus fused toes (Fts), mRNA | 1.552 |
| NM\_001001326 | Mus musculus suppression of tumorigenicity 5 (St5), mRNA | 1.551 |
| NM\_172540 | Mus musculus RIKEN cDNA 4732479N06 gene (4732479N06Rik), mRNA | 1.550 |
| AK044171 | Mus musculus 10 days neonate cortex cDNA, RIKEN full-length enriched library, clone:A830097C19 product:similar to IONOTROPIC GLUTAMATE RECEPTOR NMDA subunit NR3A[Rattus norvegicus], full insert sequence | 1.550 |
| NM\_023041 | Mus musculus peroxisome biogenesis factor 19 (Pex19), mRNA | 1.550 |
| NAP040989-1 | Unknown | 1.549 |
| NM\_019978 | Mus musculus double cortin and calcium/calmodulin-dependent protein kinase-like 1 (Dcamkl1), mRNA | 1.548 |
| NM\_177219 | Mus musculus RIKEN cDNA A730055L17 gene (A730055L17Rik), mRNA | 1.548 |
| NM\_008107 | Mus musculus growth differentiation factor 1 (Gdf1), mRNA | 1.548 |
| AK084676 | Mus musculus 13 days embryo heart cDNA, RIKEN full-length enriched library, clone:D330028N13 product:B-CELL CLL/LYMPHOMA 9 (FRAGMENT) homolog [Sus scrofa], full insert sequence. | 1.548 |
| NM\_027109 | Mus musculus RIKEN cDNA 2310005K03 gene (2310005K03Rik), mRNA | 1.547 |
| AK018785 | Mus musculus adult male cerebellum cDNA, RIKEN full-length enriched library, clone:1500036H15 product:RIT PROTEIN homolog [Homo sapiens], full insert sequence. | 1.547 |
| NM\_145478 | Mus musculus proviral integration site 3 (Pim3), mRNA | 1.547 |
| NM\_023505 | Mus musculus glutaredoxin 2 (thioltransferase) (Glrx2), mRNA | 1.546 |
| NM\_145984 | Mus musculus RIKEN cDNA D030028O16 gene (D030028O16Rik), mRNA | 1.546 |
| NM\_173038 | Mus musculus RIKEN cDNA E130107N23 gene (E130107N23Rik), mRNA | 1.546 |
| NM\_029985 | Mus musculus RIKEN cDNA A930011F22 gene (A930011F22Rik), mRNA | 1.545 |
| NM\_175184 | Mus musculus RIKEN cDNA 2610528K11 gene (2610528K11Rik), mRNA | 1.545 |
| NM\_023403 | Mus musculus mesoderm development candiate 2 (Mesdc2), mRNA | 1.544 |
| NM\_028186 | Mus musculus naked cuticle 2 homolog (Drosophila) (Nkd2), mRNA | 1.543 |
| AK079320 | Mus musculus 16 days neonate cerebellum cDNA, RIKEN full-length enriched library, clone:9630021C19 product:unknown EST, full insert sequence | 1.543 |
| NM\_133683 | Mus musculus transmembrane protein 19 (Tmem19), mRNA | 1.543 |
| AK007121 | Mus musculus adult male testis cDNA, RIKEN full-length enriched library, clone:1700104P03 product:hypothetical protein, full insert sequence. | 1.543 |
| NM\_007646 | Mus musculus CD38 antigen (Cd38), mRNA | 1.542 |
| NM\_182991 | Mus musculus RIKEN cDNA 5330410G16 gene (5330410G16Rik), mRNA | 1.542 |
| NM\_029759 | Mus musculus RIKEN cDNA 2410166I05 gene (2410166I05Rik), mRNA | 1.542 |
| NM\_177876 | Mus musculus cDNA sequence BC026744 (BC026744), mRNA | 1.541 |
| AK021021 | Mus musculus 4 days neonate male adipose cDNA, RIKEN full-length enriched library, clone:B430305P08 product:hypothetical protein, full insert sequence. | 1.540 |
| AK086536 | Mus musculus 15 days embryo head cDNA, RIKEN full-length enriched library, clone:D930035P19 product:hypothetical protein, full insert sequence. | 1.539 |
| NM\_133926 | Mus musculus calcium/calmodulin-dependent protein kinase I (Camk1), mRNA | 1.539 |
| NM\_019919 | Mus musculus latent transforming growth factor beta binding protein 1 (Ltbp1), transcript variant 1, mRNA | 1.538 |
| NM\_011627 | Mus musculus trophoblast glycoprotein (Tpbg), mRNA | 1.537 |
| NM\_053084 | Mus musculus tripartite motif protein 32 (Trim32), mRNA | 1.536 |
| NM\_177606 | Mus musculus expressed sequence AI256725 (AI256725), mRNA | 1.535 |
| BC002272 | Mus musculus Rab6 interacting protein 2, mRNA (cDNA clone IMAGE:3496274), complete cds. | 1.535 |
| NM\_010265 | Mus musculus glucosaminyl (N-acetyl) transferase 1, core 2 (Gcnt1), mRNA | 1.535 |
| BI080428 | 602876888F1 NCI\_CGAP\_Mam2 Mus musculus cDNA clone IMAGE:5008749 5', mRNA sequence | 1.534 |
| NM\_029631 | Mus musculus RIKEN cDNA 1810013B01 gene (1810013B01Rik), mRNA | 1.533 |
| AK078860 | Mus musculus adult male colon cDNA, RIKEN full-length enriched library, clone:9030225E15 product:homeo box B9, full insert sequence | 1.533 |
| NM\_026078 | Mus musculus phosphatidylinositol glycan, classC (Pigc), mRNA | 1.533 |
| NM\_025827 | Mus musculus RIKEN cDNA 1300002A08 gene (1300002A08Rik), mRNA | 1.530 |
| AJ250687 | Mus musculus partial mRNA for mg638 protein. | 1.529 |
| NM\_205536 | Mus musculus ELK3, member of ETS oncogene family (Elk3), transcript variant 2, mRNA | 1.529 |
| NM\_153679 | Mus musculus carnitine palmitoyltransferase 1c (Cpt1c), mRNA | 1.528 |
| AK017901 | Mus musculus adult male thymus cDNA, RIKEN full-length enriched library, clone:5830406C15 product:unknown EST, full insert sequence. | 1.528 |
| NM\_133734 | Mus musculus WD repeat domain 23 (Wdr23), mRNA | 1.528 |
| NM\_008800 | Mus musculus phosphodiesterase 1B, Ca2+-calmodulin dependent (Pde1b), mRNA | 1.528 |
| AK077391 | Mus musculus 6 days neonate head cDNA, RIKEN full-length enriched library, clone:5430417L22 product:unknown EST, full insert sequence | 1.527 |
| AK049219 | Mus musculus ES cells cDNA, RIKEN full-length enriched library, clone:C330013O11 product:KIAA1454-LIKE PROTEIN (FRAGMENT) homolog [Rattus norvegicus], full insert sequence. | 1.527 |
| NM\_145619 | Mus musculus ADP-ribosyltransferase (NAD+, poly (ADP-ribose polymerase)-like 3 (Adprtl3), mRNA | 1.526 |
| X71478 | M.musculus Cyp4a-10 mRNA. | 1.526 |
| AK122280 | Mus musculus mRNA for mKIAA0424 protein. | 1.526 |
| NM\_182927 | Mus musculus RIKEN cDNA D130060H24 gene (D130060H24Rik), mRNA | 1.526 |
| NM\_029590 | Mus musculus RIKEN cDNA 1700010D01 gene (1700010D01Rik), mRNA | 1.524 |
| NM\_198305 | Mus musculus cDNA sequence BC058738 (BC058738), mRNA | 1.524 |
| BC059257 | Mus musculus pam, highwire, rpm 1, mRNA (cDNA clone IMAGE:6406467), partial cds. | 1.523 |
| AK052380 | Mus musculus 13 days embryo heart cDNA, RIKEN full-length enriched library, clone:D330045P03 product:SERINE/THREONINE-PROTEIN KINASE 9 (EC 2.7.1.37) homolog [Homo sapiens], full insert sequence. | 1.521 |
| AK080874 | Mus musculus 4 days neonate male adipose cDNA, RIKEN full-length enriched library, clone:B430113B11 product:weakly similar to GC-RICH SEQUENCE DNA-BINDING FACTOR (GCF) (TRANSCRIPTION FACTOR 9) (TCF-9) [Homo sapiens], full insert sequence | 1.521 |
| NM\_183220 | Mus musculus RIKEN cDNA 2610203E10 gene (2610203E10Rik), mRNA | 1.521 |
| BC052851 | Mus musculus torsin family 3, member A, mRNA (cDNA clone IMAGE:30058244), with apparent retained intron | 1.520 |
| AK004787 | Mus musculus adult male lung cDNA, RIKEN full-length enriched library, clone:1200015G06 product:unknown EST, full insert sequence. | 1.520 |
| XM\_110852 | Mus musculus RIKEN cDNA 2810455B10 gene (2810455B10Rik), mRNA | 1.520 |
| NM\_178888 | Mus musculus GTPase activating RANGAP domain-like 3 (Garnl3), mRNA | 1.519 |
| AK018430 | Mus musculus 16 days embryo lung cDNA, RIKEN full-length enriched library, clone:8430421H08 product:similar to IDN4-GGTR7 PROTEIN [Homo sapiens], full insert sequence. | 1.518 |
| AK017227 | Mus musculus adult male xiphoid cartilage cDNA, RIKEN full-length enriched library, clone:5230400J22 product:carbonic anhydrase 3, full insert sequence. | 1.518 |
| NM\_145553 | Mus musculus cDNA sequence BC008163 (BC008163), mRNA | 1.517 |
| AK090119 | Mus musculus female sarcoma RCB-0464 Meth-A cDNA, RIKEN full-length enriched library, clone:G431001E03 product:similar to BA541N10.2 (NOVEL PROTEIN (ORTHOLOG OF MOUSE FISH PROTEIN)) (FRAGMENT) [Homo sapiens], full insert sequence | 1.517 |
| NM\_030749 | Mus musculus RIKEN cDNA 1810057E01 gene (1810057E01Rik), mRNA | 1.516 |
| NM\_011199 | Mus musculus parathyroid hormone receptor 1 (Pthr1), mRNA | 1.516 |
| AK038840 | Mus musculus adult male hypothalamus cDNA, RIKEN full-length enriched library, clone:A230067K14 product:COLLYBISTIN II homolog [Rattus norvegicus], full insert sequence. | 1.515 |
| NM\_145356 | Mus musculus RIKEN cDNA B230208J24 gene (B230208J24Rik), mRNA | 1.515 |
| BC064745 | Mus musculus cDNA clone MGC:76529 IMAGE:30093795, complete cds. | 1.514 |
| NM\_008450 | Mus musculus kinesin 2 (Kns2), mRNA | 1.514 |
| NM\_019877 | Mus musculus coatomer protein complex, subunit zeta 2 (Copz2), mRNA | 1.514 |
| NM\_019656 | Mus musculus transmembrane 4 superfamily member 6 (Tm4sf6), mRNA | 1.514 |
| NM\_019961 | Mus musculus peroxisomal biogenesis factor 3 (Pex3), mRNA | 1.513 |
| NM\_032418 | Mus musculus dystrophia myotonica kinase, B15 (Dm15), mRNA | 1.513 |
| NM\_025562 | Mus musculus tetratricopeptide repeat domain 11 (Ttc11), mRNA | 1.513 |
| NM\_016721 | Mus musculus IQ motif containing GTPase activating protein 1 (Iqgap1), mRNA | 1.513 |
| NM\_018769 | Mus musculus deafness, autosomal dominant 5 homolog (human) (Dfna5h), mRNA | 1.512 |
| NM\_025635 | Mus musculus DNA segment, Chr 10, ERATO Doi 749, expressed (D10Ertd749e), mRNA | 1.512 |
| AK006823 | Mus musculus adult male testis cDNA, RIKEN full-length enriched library, clone:1700057H01 product:unknown EST, full insert sequence. | 1.511 |
| NM\_177353 | Mus musculus RIKEN cDNA A530087D17 gene (A530087D17Rik), mRNA | 1.511 |
| AK082166 | Mus musculus 0 day neonate cerebellum cDNA, RIKEN full-length enriched library, clone:C230016J21 product:unknown EST, full insert sequence | 1.510 |
| NM\_028836 | Mus musculus chitobiase, di-N-acetyl- (Ctbs), mRNA | 1.510 |
| NM\_145602 | Mus musculus N-myc downstream regulated gene 4 (Ndrg4), mRNA | 1.510 |
| NM\_013468 | Mus musculus ankyrin repeat domain 1 (cardiac muscle) (Ankrd1), mRNA | 1.509 |
| NM\_020009 | Mus musculus FK506 binding protein 12-rapamycin associated protein 1 (Frap1), mRNA | 1.509 |
| NM\_026179 | Mus musculus abhydrolase domain containing 5 (Abhd5), mRNA | 1.509 |
| NM\_054093 | Mus musculus ubiquitin protein ligase E3B (Ube3b), mRNA | 1.509 |
| BC046969 | Mus musculus mRNA similar to hypothetical protein DKFZp564L2423 (cDNA clone MGC:54877 IMAGE:6313679), complete cds. | 1.508 |
| NM\_172661 | Mus musculus RIKEN cDNA 5830434P21 gene (5830434P21Rik), mRNA | 1.508 |
| AK034301 | Mus musculus adult male diencephalon cDNA, RIKEN full-length enriched library, clone:9330175B01 product:hypothetical protein, full insert sequence. | 1.508 |
| NM\_011101 | Mus musculus protein kinase C, alpha (Prkca), mRNA | 1.507 |
| NM\_027756 | Mus musculus RIKEN cDNA 4933428A15 gene (4933428A15Rik), mRNA | 1.507 |
| NM\_011879 | Mus musculus IK cytokine (Ik), mRNA | 1.506 |
| NM\_172784 | Mus musculus low density lipoprotein receptor-related protein 11 (Lrp11), mRNA | 1.506 |
| NM\_019922 | Mus musculus cartilage associated protein (Crtap), mRNA | 1.506 |
| NM\_175484 | Mus musculus RIKEN cDNA E130012P22 gene (E130012P22Rik), mRNA | 1.506 |
| NM\_020332 | Mus musculus progressive ankylosis (Ank), mRNA | 1.506 |
| NM\_009624 | Mus musculus adenylate cyclase 9 (Adcy9), mRNA | 1.506 |
| AK031405 | Mus musculus 13 days embryo male testis cDNA, RIKEN full-length enriched library, clone:6030424E15 product:hypothetical Major sperm protein (MSP) domain/Cellular retinaldehyde-binding protein (CRAL)/Triple function domain (TRIO) containing protein, full | 1.505 |
| NM\_018781 | Mus musculus early growth response 3 (Egr3), mRNA | 1.505 |
| NM\_013891 | Mus musculus SAM pointed domain containing ets transcription factor (Spdef), mRNA | 1.504 |
| AK046595 | Mus musculus 4 days neonate male adipose cDNA, RIKEN full-length enriched library, clone:B430201A12 product:weakly similar to RE17452P [Drosophila melanogaster], full insert sequence. | 1.503 |
| NM\_010277 | Mus musculus glial fibrillary acidic protein (Gfap), mRNA | 1.502 |
| NM\_019488 | Mus musculus solute carrier family 2, (facilitated glucose transporter), member 8 (Slc2a8), mRNA | 1.502 |
| NM\_172782 | Mus musculus RIKEN cDNA 6330587F24 gene (6330587F24Rik), mRNA | 1.501 |
| NAP061485-1 | Unknown | 1.501 |
| NM\_177305 | Mus musculus RIKEN cDNA A630084M22 gene (A630084M22Rik), mRNA | 1.501 |
| AK041989 | Mus musculus 3 days neonate thymus cDNA, RIKEN full-length enriched library, clone:A630051C02 product:unknown EST, full insert sequence. | 1.500 |
| NM\_053074 | Mus musculus nucleoporin 62 (Nup62), mRNA | 0.667 |
| NM\_008565 | Mus musculus minichromosome maintenance deficient 4 homolog (S. cerevisiae) (Mcm4), mRNA | 0.666 |
| NM\_133993 | Mus musculus RIKEN cDNA 2310058A11 gene (2310058A11Rik), mRNA | 0.666 |
| NM\_177564 | Mus musculus cDNA sequence BC022224 (BC022224), mRNA | 0.666 |
| AK029149 | Mus musculus 10 days neonate skin cDNA, RIKEN full-length enriched library, clone:4732497K21 product:weakly similar to RIBONUCLEASE 6 PRECURSOR [Homo sapiens], full insert sequence | 0.666 |
| AK078801 | Mus musculus 16 days embryo lung cDNA, RIKEN full-length enriched library, clone:8430401M20 product:apoptosis inhibitory protein 5, full insert sequence. | 0.666 |
| NM\_013924 | Mus musculus activator of basal transcription (Abt1), mRNA | 0.666 |
| NM\_029761 | Mus musculus docking protein 5 (Dok5), mRNA | 0.666 |
| AK017725 | Mus musculus 8 days embryo whole body cDNA, RIKEN full-length enriched library, clone:5730493J03 product:tropomodulin 3, full insert sequence. | 0.666 |
| NM\_173737 | Mus musculus RIKEN cDNA 8430410A17 gene (8430410A17Rik), mRNA | 0.665 |
| AK052419 | Mus musculus 13 days embryo lung cDNA, RIKEN full-length enriched library, clone:D430014C20 product:hypothetical alpha/beta-Hydrolases structure containing protein, full insert sequence. | 0.665 |
| AK035842 | Mus musculus 16 days neonate cerebellum cDNA, RIKEN full-length enriched library, clone:9630009N10 product:unc5 homolog (C. elegans) 3, full insert sequence. | 0.665 |
| NM\_173441 | Mus musculus RIKEN cDNA 1700069O15 gene (1700069O15Rik), mRNA | 0.665 |
| NM\_175195 | Mus musculus RIKEN cDNA 3110056O03 gene (3110056O03Rik), mRNA | 0.665 |
| NM\_008251 | Mus musculus high mobility group nucleosomal binding domain 1 (Hmgn1), mRNA | 0.665 |
| NAP061492-1 | Unknown | 0.665 |
| NM\_148941 | Mus musculus elongation of very long chain fatty acids (FEN1/Elo2, SUR4/Elo3, yeast)-like 4 (Elovl4), mRNA | 0.665 |
| NM\_146218 | Mus musculus cDNA sequence BC027246 (BC027246), mRNA | 0.665 |
| NM\_012053 | Mus musculus ribosomal protein L8 (Rpl8), mRNA | 0.665 |
| BC049262 | Mus musculus RIKEN cDNA 2810442I22 gene, mRNA (cDNA clone IMAGE:5365647), partial cds. | 0.664 |
| NM\_133692 | Mus musculus polymerase (DNA-directed), delta 3, accessory subunit (Pold3), mRNA | 0.664 |
| NM\_007809 | Mus musculus cytochrome P450, family 17, subfamily a, polypeptide 1 (Cyp17a1), mRNA | 0.664 |
| NM\_008908 | Mus musculus peptidylprolyl isomerase C (Ppic), mRNA | 0.664 |
| AK009365 | Mus musculus adult male tongue cDNA, RIKEN full-length enriched library, clone:2310015K15 product:hypothetical protein, full insert sequence. | 0.664 |
| AK010815 | Mus musculus ES cells cDNA, RIKEN full-length enriched library, clone:2410153K17 product:hypothetical Armadillo repeat/Armadillo/plakoglobin ARM repeat profile containing protein, full insert sequence. | 0.664 |
| AK122228 | Mus musculus mRNA for mKIAA0241 protein. | 0.663 |
| XM\_127312 | Mus musculus expressed sequence C78339 (C78339), mRNA | 0.663 |
| NM\_172117 | Mus musculus ectonucleoside triphosphate diphosphohydrolase 6 (Entpd6), mRNA | 0.663 |
| NM\_178643 | Mus musculus RIKEN cDNA C230052I12 gene (C230052I12Rik), mRNA | 0.662 |
| NM\_030064 | Mus musculus RIKEN cDNA 2410141M05 gene (2410141M05Rik), mRNA | 0.662 |
| NM\_009418 | Mus musculus tripeptidyl peptidase II (Tpp2), mRNA | 0.662 |
| NM\_007476 | Mus musculus ADP-ribosylation factor 1 (Arf1), mRNA | 0.662 |
| M17518 | Mouse lactate dehydrogenase A-4 pseudogene mRNA, 3' end. | 0.662 |
| AK122503 | Mus musculus mRNA for mKIAA1400 protein. | 0.662 |
| NM\_052993 | Mus musculus core 1 UDP-galactose:N-acetylgalactosamine-alpha-R beta 1,3-galactosyltransferase (C1galt1), mRNA | 0.662 |
| NM\_008231 | Mus musculus hepatoma-derived growth factor (Hdgf), mRNA | 0.662 |
| XM\_139515 | Mus musculus hypothetical protein 9030019H09 (9030019H09), mRNA | 0.662 |
| NM\_030209 | Mus musculus RIKEN cDNA 1810049K24 gene (1810049K24Rik), mRNA | 0.662 |
| NM\_023292 | Mus musculus RIKEN cDNA 2610020J05 gene (2610020J05Rik), mRNA | 0.662 |
| AK017206 | Mus musculus 11 days pregnant adult female ovary and uterus cDNA, RIKEN full-length enriched library, clone:5033430I15 product:hypothetical protein, full insert sequence. | 0.662 |
| AK015651 | Mus musculus adult male testis cDNA, RIKEN full-length enriched library, clone:4930488P18 product:hypothetical Lysine-rich region containing protein, full insert sequence. | 0.662 |
| AK041481 | Mus musculus 3 days neonate thymus cDNA, RIKEN full-length enriched library, clone:A630014C11 product:hypothetical 'Winged helix' DNA-binding domain structure containing protein, full insert sequence. | 0.661 |
| NM\_173453 | Mus musculus RIKEN cDNA 5730466P16 gene (5730466P16Rik), mRNA | 0.661 |
| NM\_153570 | Mus musculus expressed sequence AI326906 (AI326906), mRNA | 0.661 |
| TC1024770 | AF302077 neprilysin-like peptidase gamma {Mus musculus}, partial (5%) | 0.661 |
| NM\_133227 | Mus musculus nucleoporin 155 (Nup155), mRNA | 0.661 |
| AK032995 | Mus musculus 12 days embryo male wolffian duct includes surrounding region cDNA, RIKEN full-length enriched library, clone:6720487G11 product:unclassifiable, full insert sequence. | 0.661 |
| BY723611 | BY723611 RIKEN full-length enriched, 16 days neonate thymus Mus musculus cDNA clone A130085H05 5'. | 0.661 |
| NM\_019930 | Mus musculus RAN binding protein 9 (Ranbp9), mRNA | 0.661 |
| AK081480 | Mus musculus 16 days embryo head cDNA, RIKEN full-length enriched library, clone:C130020C13 product:unknown EST, full insert sequence. | 0.661 |
| BC068207 | Mus musculus RIKEN cDNA 5730445F03 gene, mRNA (cDNA clone IMAGE:5255534), partial cds. | 0.661 |
| NM\_138659 | Mus musculus pre-mRNA processing factor 8 (Prpf8), mRNA | 0.661 |
| NM\_019570 | Mus musculus REV1-like (S. cerevisiae) (Rev1l), mRNA | 0.661 |
| NM\_011665 | Mus musculus ubiquitin-conjugating enzyme E2I (Ube2i), mRNA | 0.661 |
| AY314008 | Mus musculus skin calmodulin-related protein 2 (Scarf2) mRNA, complete cds. | 0.660 |
| AK012351 | Mus musculus 11 days embryo whole body cDNA, RIKEN full-length enriched library, clone:2700038P16 product:hypothetical Amidase containing protein, full insert sequence. | 0.660 |
| NM\_054050 | Mus musculus Rap guanine nucleotide exchange factor (GEF) 1 (Rapgef1), mRNA | 0.660 |
| NM\_009648 | Mus musculus A kinase (PRKA) anchor protein 1 (Akap1), mRNA | 0.660 |
| AK079430 | Mus musculus adult female vagina cDNA, RIKEN full-length enriched library, clone:9930005C23 product:ATPase, aminophospholipid transporter (APLT), class I, type 8A, member 1, full insert sequence. | 0.660 |
| NM\_013854 | Mus musculus ATP-binding cassette, sub-family F (GCN20), member 1 (Abcf1), mRNA | 0.660 |
| BC058968 | Mus musculus methionine-tRNA synthetase, mRNA (cDNA clone IMAGE:6816085), partial cds | 0.660 |
| NM\_023628 | Mus musculus annexin A9 (Anxa9), mRNA | 0.660 |
| NM\_197981 | Mus musculus RIKEN cDNA 5930416I19 gene (5930416I19Rik), mRNA | 0.660 |
| AK083742 | Mus musculus 9 days embryo whole body cDNA, RIKEN full-length enriched library, clone:D030073O17 product:unknown EST, full insert sequence. | 0.660 |
| AY155196 | Mus musculus truncated groucho protein GRG1-S (Tle1) mRNA, complete cds; alternatively spliced. | 0.660 |
| NM\_011818 | Mus musculus germ cell-less homolog (Drosophila) (Gcl), mRNA | 0.660 |
| NM\_177577 | Mus musculus doublecortin domain containing 2 (Dcdc2), mRNA | 0.660 |
| TC1062282 | AF182814 methionine adenosyltransferase regulatory beta subunit {Homo sapiens}, partial (26%) | 0.659 |
| AK004148 | Mus musculus 18-day embryo whole body cDNA, RIKEN full-length enriched library, clone:1110037P11 product:weakly similar to MYOSIN-BINDING PROTEIN H (MYBP-H) (H-PROTEIN) [Mus musculus], full insert sequence. | 0.659 |
| NM\_028207 | Mus musculus dual specificity phosphatase 3 (vaccinia virus phosphatase VH1-related) (Dusp3), mRNA | 0.659 |
| NM\_023231 | Mus musculus stomatin (Epb7.2)-like 2 (Stoml2), mRNA | 0.659 |
| BC053752 | Mus musculus cDNA clone MGC:59570 IMAGE:6506619, complete cds. | 0.659 |
| BC051409 | Mus musculus RNA binding motif protein 15, mRNA (cDNA clone IMAGE:6490953), partial cds. | 0.659 |
| NM\_010469 | Mus musculus homeo box D4 (Hoxd4), mRNA | 0.658 |
| NM\_008189 | Mus musculus guanylate cyclase activator 1a (retina) (Guca1a), mRNA | 0.658 |
| NM\_021343 | Mus musculus spermatogenesis associated 5 (Spata5), mRNA | 0.658 |
| NM\_011564 | Mus musculus sex determining region of Chr Y (Sry), mRNA | 0.658 |
| NM\_011903 | Mus musculus tousled-like kinase 2 (Arabidopsis) (Tlk2), mRNA | 0.658 |
| NM\_134250 | Mus musculus hepatitis A virus cellular receptor 2 (Havcr2), mRNA | 0.658 |
| AY319515 | Mus musculus TARDBP S8 (TARDBP) mRNA, complete cds, alternative transcript. | 0.657 |
| NM\_134009 | Mus musculus RIKEN cDNA 3100002P13 gene (3100002P13Rik), mRNA | 0.657 |
| NM\_173392 | Mus musculus zinc finger, FYVE domain containing 16 (Zfyve16), mRNA | 0.657 |
| NM\_009269 | Mus musculus serine palmitoyltransferase, long chain base subunit 1 (Sptlc1), mRNA | 0.657 |
| AK080389 | Mus musculus 3 days neonate thymus cDNA, RIKEN full-length enriched library, clone:A630089O11 product:unclassifiable, full insert sequence. | 0.657 |
| NM\_025829 | Mus musculus RIKEN cDNA 1300018P11 gene (1300018P11Rik), mRNA | 0.657 |
| AK006541 | Mus musculus adult male testis cDNA, RIKEN full-length enriched library, clone:1700030F05 product:fatty acid Coenzyme A ligase, long chain 5, full insert sequence. | 0.657 |
| NM\_146034 | Mus musculus meningioma expressed antigen 6 (coiled-coil proline-rich) (Mgea6), mRNA | 0.657 |
| NM\_009574 | Mus musculus Zic family member 2 (odd-paired homolog, Drosophila) (Zic2), mRNA | 0.657 |
| AK037330 | Mus musculus 16 days neonate thymus cDNA, RIKEN full-length enriched library, clone:A130007E13 product:unclassifiable, full insert sequence | 0.657 |
| NM\_134115 | Mus musculus serine/threonine kinase 38 (Stk38), mRNA | 0.657 |
| NM\_153538 | Mus musculus zinc finger, CCHC domain containing 6 (Zcchc6), mRNA | 0.656 |
| NM\_009727 | Mus musculus ATPase, aminophospholipid transporter (APLT), class I, type 8A, member 1 (Atp8a1), mRNA | 0.656 |
| NM\_027351 | Mus musculus peptidylprolyl isomerase (cyclophilin)-like 3 (Ppil3), mRNA | 0.656 |
| NM\_146003 | Mus musculus SUMO/sentrin specific protease 6 (Senp6), mRNA | 0.656 |
| NM\_028083 | Mus musculus chromatin assembly factor 1, subunit B (p60) (Chaf1b), mRNA | 0.656 |
| NM\_198304 | Mus musculus nucleoporin 188 (Nup188), mRNA | 0.656 |
| NM\_177333 | Mus musculus SEC6-like 1 (S. cerevisiae) (Sec6l1), mRNA | 0.656 |
| ENSMUST00000039918 | Unknown | 0.656 |
| NM\_011317 | Mus musculus KH domain containing, RNA binding, signal transduction associated 1 (Khdrbs1), mRNA | 0.656 |
| NM\_173740 | Mus musculus monoamine oxidase A (Maoa), mRNA | 0.656 |
| NM\_009457 | Mus musculus ubiquitin-activating enzyme E1, Chr X (Ube1x), mRNA | 0.656 |
| NM\_011304 | Mus musculus RuvB-like protein 2 (Ruvbl2), mRNA | 0.655 |
| NM\_021468 | Mus musculus unc-13 homolog A (C. elegans) (Unc13a), mRNA | 0.655 |
| X69942 | M.musculus mRNA of enhancer-trap-locus 1. | 0.655 |
| BC059218 | Mus musculus expressed sequence AI256361, mRNA (cDNA clone IMAGE:6409892), complete cds. | 0.655 |
| NM\_146066 | Mus musculus G1 to phase transition 1 (Gspt1), mRNA | 0.655 |
| NM\_029091 | Mus musculus RIKEN cDNA 1200014P03 gene (1200014P03Rik), mRNA | 0.655 |
| NM\_023585 | Mus musculus ubiquitin-conjugating enzyme E2 variant 2 (Ube2v2), mRNA | 0.655 |
| NM\_011462 | Mus musculus spindlin (Spin), mRNA | 0.655 |
| BC024681 | Mus musculus hypothetical protein LOC230595, mRNA (cDNA clone IMAGE:3662238), with apparent retained intron | 0.655 |
| BB781167 | BB781167 RIKEN full-length enriched, RCB-0035 WEHI-3 cDNA Mus musculus cDNA clone G430071H02 3', mRNA sequence | 0.654 |
| NM\_008891 | Mus musculus pinin (Pnn), mRNA | 0.654 |
| Y17344 | Mus musculus mRNA for protein-tyrosine-phosphatase IF2. | 0.654 |
| AI428996 | AI428996 vo85e06.y1 Barstead mouse irradiated colon MPLRB7 Mus musculus cDNA clone IMAGE:1065922 5', mRNA sequence | 0.654 |
| AK002307 | Mus musculus adult male kidney cDNA, RIKEN full-length enriched library, clone:0610007P08 product:PUTATIVE REPAIR AND RECOMBINATION HELICASE RAD26L (FRAGMENT) homolog [Mus musculus], full insert sequence. | 0.654 |
| TC955750 | BC030378 solute carrier family 38, member 1 {Mus musculus}, complete | 0.654 |
| NM\_172714 | Mus musculus expressed sequence AI461788 (AI461788), mRNA | 0.653 |
| AK090026 | Mus musculus embryo RCB-0549 Cle-H3 cDNA, RIKEN full-length enriched library, clone:G430065H05 product:HYPOTHETICAL 75.2 KDA PROTEIN homolog [Homo sapiens], full insert sequence | 0.653 |
| NM\_198101 | Mus musculus Gem-interacting protein (Gmip), mRNA | 0.653 |
| NM\_011945 | Mus musculus mitogen activated protein kinase kinase kinase 1 (Map3k1), mRNA | 0.653 |
| NM\_007896 | Mus musculus microtubule-associated protein, RP/EB family, member 1 (Mapre1), mRNA | 0.653 |
| BC006897 | Mus musculus protein phosphatase 1, regulatory (inhibitor) subunit 15b, mRNA (cDNA clone IMAGE:3599662), partial cds. | 0.653 |
| AK083076 | Mus musculus 12 days embryo spinal cord cDNA, RIKEN full-length enriched library, clone:C530045D03 product:pyruvate kinase 3, full insert sequence. | 0.652 |
| NM\_011307 | Mus musculus retinoid X receptor interacting protein 110 (Rxrip110), mRNA | 0.652 |
| AI627124 | mu56a10.y1 Soares mouse lymph node NbMLN Mus musculus cDNA clone IMAGE:643386 5'. | 0.652 |
| NM\_025630 | Mus musculus RIKEN cDNA 2010009L17 gene (2010009L17Rik), mRNA | 0.652 |
| AK079933 | Mus musculus adult male aorta and vein cDNA, RIKEN full-length enriched library, clone:A530014K12 product:unknown EST, full insert sequence. | 0.652 |
| AK031338 | Mus musculus 13 days embryo male testis cDNA, RIKEN full-length enriched library, clone:6030408K16 product:antigen identified by monoclonal antibody 2A8, full insert sequence. | 0.652 |
| NAP058478-1 | Unknown | 0.652 |
| NM\_030724 | Mus musculus uridine monophosphate kinase (Umpk), mRNA | 0.652 |
| NM\_026002 | Mus musculus RIKEN cDNA 2610103J23 gene (2610103J23Rik), mRNA | 0.652 |
| AK049589 | Mus musculus 7 days embryo whole body cDNA, RIKEN full-length enriched library, clone:C430048E02 product:hypothetical protein, full insert sequence. | 0.652 |
| AK031051 | Mus musculus adult male thymus cDNA, RIKEN full-length enriched library, clone:5832443G15 product:unclassifiable, full insert sequence. | 0.652 |
| NM\_144543 | Mus musculus thymocyte protein thy28 (Thy28), mRNA | 0.651 |
| NM\_152234 | Mus musculus ubiquilin 1 (Ubqln1), transcript variant 2, mRNA | 0.651 |
| U34973 | Mus musculus protein tyrosine phosphatase-like mRNA, unspliced c-terminal product and spliced c-terminal end STYX, complete cds. | 0.651 |
| NM\_024250 | Mus musculus PHD finger protein 10 (Phf10), mRNA | 0.651 |
| XM\_358059 | Mus musculus similar to U2 small nuclear ribonucleoprotein B (LOC385110), mRNA | 0.651 |
| NM\_010401 | Mus musculus histidine ammonia lyase (Hal), mRNA | 0.651 |
| NM\_175205 | Mus musculus RIKEN cDNA 4632419I22 gene (4632419I22Rik), mRNA | 0.651 |
| NM\_018861 | Mus musculus solute carrier family 1 (glutamate/neutral amino acid transporter), member 4 (Slc1a4), mRNA | 0.651 |
| NM\_008781 | Mus musculus paired box gene 3 (Pax3), mRNA | 0.651 |
| NM\_019770 | Mus musculus RIKEN cDNA 1110032D12 gene (1110032D12Rik), mRNA | 0.650 |
| NM\_177252 | Mus musculus RIKEN cDNA 9630011N22 gene (9630011N22Rik), mRNA | 0.650 |
| NM\_025680 | Mus musculus catenin, beta like 1 (Ctnnbl1), mRNA | 0.650 |
| NM\_025825 | Mus musculus amyloid beta precursor protein (cytoplasmic tail) binding protein 2 (Appbp2), mRNA | 0.650 |
| AK087319 | Mus musculus 0 day neonate lung cDNA, RIKEN full-length enriched library, clone:E030044I16 product:hypothetical protein, full insert sequence. | 0.650 |
| NM\_018776 | Mus musculus cytokine receptor-like factor 3 (Crlf3), mRNA | 0.650 |
| NM\_029092 | Mus musculus RNA (guanine-9-) methyltransferase domain containing 1 (Rg9mtd1), mRNA | 0.650 |
| NM\_011483 | Mus musculus zinc and ring finger 4 (Znrf4), mRNA | 0.650 |
| NM\_027149 | Mus musculus RIKEN cDNA 2310040A13 gene (2310040A13Rik), mRNA | 0.650 |
| NM\_012019 | Mus musculus programmed cell death 8 (Pdcd8), mRNA | 0.649 |
| BC043060 | Mus musculus FUS interacting protein (serine-arginine rich) 1, mRNA (cDNA clone MGC:57963 IMAGE:6419883), complete cds. | 0.649 |
| NM\_020046 | Mus musculus dihydroorotate dehydrogenase (Dhodh), mRNA | 0.649 |
| NM\_201230 | Mus musculus Fgfr1 oncogene partner (Fgfr1op), mRNA | 0.649 |
| NM\_153196 | Mus musculus ribokinase (Rbks), mRNA | 0.649 |
| NM\_013699 | Mus musculus upstream binding protein 1 (Ubp1), mRNA | 0.649 |
| NM\_023799 | Mus musculus meningioma expressed antigen 5 (hyaluronidase) (Mgea5), mRNA | 0.649 |
| NM\_025407 | Mus musculus ubiquinol-cytochrome c reductase core protein 1 (Uqcrc1), mRNA | 0.649 |
| NM\_009068 | Mus musculus receptor (TNFRSF)-interacting serine-threonine kinase 1 (Ripk1), mRNA | 0.649 |
| BC020137 | Mus musculus RIKEN cDNA 1500032H18 gene, mRNA (cDNA clone MGC:28063 IMAGE:3709157), complete cds. | 0.649 |
| NM\_016737 | Mus musculus stress-induced phosphoprotein 1 (Stip1), mRNA | 0.648 |
| NM\_019794 | Mus musculus DnaJ (Hsp40) homolog, subfamily A, member 2 (Dnaja2), mRNA | 0.648 |
| NM\_010833 | Mus musculus moesin (Msn), mRNA | 0.648 |
| NM\_024274 | Mus musculus phenylalanine-tRNA synthetase 1 (mitochondrial) (Fars1), mRNA | 0.648 |
| NM\_175106 | Mus musculus RIKEN cDNA 2300008B03 gene (2300008B03Rik), mRNA | 0.648 |
| NM\_026603 | Mus musculus density-regulated protein (Denr), mRNA | 0.648 |
| NM\_009516 | Mus musculus wee 1 homolog (S. pombe) (Wee1), mRNA | 0.648 |
| NM\_025921 | Mus musculus RIKEN cDNA 2610002M06 gene (2610002M06Rik), mRNA | 0.648 |
| AK041753 | Mus musculus 3 days neonate thymus cDNA, RIKEN full-length enriched library, clone:A630035A22 product:unknown EST, full insert sequence | 0.647 |
| XM\_354572 | Mus musculus similar to lin-28 homolog; RNA-binding protein LIN-28; zinc finger, CCHC domain containing 1 (LOC380669), mRNA | 0.647 |
| NM\_020275 | Mus musculus tumor necrosis factor receptor superfamily, member 10b (Tnfrsf10b), mRNA | 0.647 |
| AK088974 | Mus musculus 2 days neonate thymus thymic cells cDNA, RIKEN full-length enriched library, clone:E430034D20 product:BIFUNCTIONAL AMINOACYL-TRNA SYNTHETASE [INCLUDES: GLUTAMYL-TRNA SYNTHETASE (EC 6.1.1.17) (GLUTAMATE--TRNA LIGASE); PROLYL-TRNA SYNTHETASE | 0.647 |
| AK003078 | Mus musculus adult male spleen cDNA, RIKEN full-length enriched library, clone:0910001J09 product:ubiquitin carrier protein E2 homolog [Homo sapiens], full insert sequence | 0.647 |
| NM\_013711 | Mus musculus thioredoxin reductase 2 (Txnrd2), mRNA | 0.647 |
| AK021408 | Mus musculus 0 day neonate eyeball cDNA, RIKEN full-length enriched library, clone:E130301L11 product:weakly similar to TROPHININ-ASSOCIATED PROTEIN (TASTIN) (TROPHININ-ASSISTING PROTEIN) [Homo sapiens], full insert sequence. | 0.647 |
| AK042102 | Mus musculus 3 days neonate thymus cDNA, RIKEN full-length enriched library, clone:A630058G12 product:hypothetical protein, full insert sequence. | 0.647 |
| NM\_026402 | Mus musculus APG3 autophagy 3-like (S. cerevisiae) (Apg3l), mRNA | 0.647 |
| NM\_026422 | Mus musculus mitochondrial ribosome recycling factor (Mrrf), mRNA | 0.647 |
| ENSMUST00000023146 | NUCLEOTIDE-BINDING PROTEIN 1 (NBP 1). [Source:SWISSPROT;Acc:Q9R060] | 0.647 |
| AK043288 | Mus musculus 7 days neonate cerebellum cDNA, RIKEN full-length enriched library, clone:A730081H22 product:similar to I3 BINDING PROTEIN [Homo sapiens], full insert sequence. | 0.647 |
| NM\_009138 | Mus musculus chemokine (C-C motif) ligand 25 (Ccl25), mRNA | 0.646 |
| NM\_030152 | Mus musculus nucleolar protein 3 (apoptosis repressor with CARD domain) (Nol3), mRNA | 0.646 |
| BC024640 | Mus musculus RIKEN cDNA 2010003J03 gene, mRNA (cDNA clone MGC:28430 IMAGE:4038046), complete cds. | 0.646 |
| NM\_007932 | Mus musculus endoglin (Eng), mRNA | 0.646 |
| AK004450 | Mus musculus 18-day embryo whole body cDNA, RIKEN full-length enriched library, clone:1190002H09 product:hypothetical protein, full insert sequence. | 0.646 |
| AK029866 | Mus musculus adult male testis cDNA, RIKEN full-length enriched library, clone:4931417G23 product:hypothetical ATP/GTP-binding site motif A (P-loop) containing protein, full insert sequence. | 0.646 |
| XM\_354943 | Mus musculus similar to hypothetical protein MGC24665 (LOC381034), mRNA | 0.646 |
| NM\_010268 | Mus musculus ganglioside-induced differentiation-associated-protein 10 (Gdap10), mRNA | 0.646 |
| AK122507 | Mus musculus mRNA for mKIAA1423 protein | 0.646 |
| XM\_205168 | Mus musculus similar to L-lactate dehydrogenase A chain (LDH-A) (LDH muscle subunit) (LDH-M) (LOC278062), mRNA | 0.646 |
| XM\_136222 | Mus musculus similar to hypothetical protein MGC10120 (LOC240750), mRNA | 0.646 |
| NAP033304-1 | Unknown | 0.645 |
| NM\_007552 | Mus musculus B lymphoma Mo-MLV insertion region 1 (Bmi1), mRNA | 0.645 |
| Y15798 | Mus musculus mRNA for G-protein coupled receptor kinase 6-B. | 0.645 |
| NM\_009323 | Mus musculus T-box 15 (Tbx15), mRNA | 0.645 |
| NM\_009451 | Mus musculus tubulin, beta 4 (Tubb4), mRNA | 0.645 |
| NM\_011130 | Mus musculus polymerase (DNA directed), beta (Polb), mRNA | 0.645 |
| BC028790 | Mus musculus BCL2/adenovirus E1B 19kDa-interacting protein 1, NIP1, mRNA (cDNA clone IMAGE:1264175), with apparent retained intron. | 0.645 |
| BC040802 | Mus musculus glutamyl-prolyl-tRNA synthetase, mRNA (cDNA clone IMAGE:3980665), partial cds. | 0.645 |
| NM\_021529 | Mus musculus RIKEN cDNA 4930511A21 gene (4930511A21Rik), mRNA | 0.644 |
| NM\_011791 | Mus musculus ash2 (absent, small, or homeotic)-like (Drosophila) (Ash2l), mRNA | 0.644 |
| NM\_145985 | Mus musculus archain 1 (Arcn1), mRNA | 0.644 |
| AV166465 | AV166465 AV166465 Mus musculus head C57BL/6J 13-day embryo Mus musculus cDNA clone 3110045H24, mRNA sequence | 0.644 |
| NM\_026713 | Mus musculus monoacylglycerol O-acyltransferase 1 (Mogat1), mRNA | 0.644 |
| NM\_027532 | Mus musculus RIKEN cDNA 3200002M19 gene (3200002M19Rik), mRNA | 0.644 |
| BC062923 | Mus musculus cDNA clone MGC:73431 IMAGE:6848546, complete cds. | 0.644 |
| NM\_212444 | Mus musculus glycerol kinase (Gyk), transcript variant 2, mRNA | 0.644 |
| AK037311 | Mus musculus 16 days neonate thymus cDNA, RIKEN full-length enriched library, clone:A130005I12 product:unknown EST, full insert sequence. | 0.644 |
| NM\_025460 | Mus musculus RIKEN cDNA 1810020E01 gene (1810020E01Rik), mRNA | 0.644 |
| NM\_016774 | Mus musculus ATP synthase, H+ transporting mitochondrial F1 complex, beta subunit (Atp5b), mRNA | 0.644 |
| AK049789 | Mus musculus 12 days embryo spinal cord cDNA, RIKEN full-length enriched library, clone:C530050A08 product:similar to RNA POLYMERASE III SUBUNIT [Homo sapiens], full insert sequence. | 0.644 |
| NM\_028117 | Mus musculus dermatan 4 sulfotransferase 1 (D4st1), mRNA | 0.643 |
| NAP096498-001 | Unknown | 0.643 |
| NM\_029522 | Mus musculus G-protein signalling modulator 2 (AGS3-like, C. elegans) (Gpsm2), mRNA | 0.643 |
| NM\_177090 | Mus musculus RIKEN cDNA D330022A01 gene (D330022A01Rik), mRNA | 0.643 |
| NM\_138669 | Mus musculus DEAD (Asp-Glu-Ala-Asp) box polypeptide 48 (Ddx48), mRNA | 0.643 |
| NM\_026964 | Mus musculus RIKEN cDNA 1810023B24 gene (1810023B24Rik), mRNA | 0.643 |
| NM\_133837 | Mus musculus RIKEN cDNA G431001I09 gene (G431001I09Rik), mRNA | 0.643 |
| NM\_010481 | Mus musculus heat shock protein, A (Hspa9a), mRNA | 0.643 |
| AK051789 | Mus musculus 12 days embryo spinal ganglion cDNA, RIKEN full-length enriched library, clone:D130081A22 product:unknown EST, full insert sequence. | 0.643 |
| NM\_007530 | Mus musculus B-cell receptor-associated protein 29 (Bcap29), mRNA | 0.643 |
| NM\_025844 | Mus musculus cysteine and histidine-rich domain (CHORD)-containing, zinc-binding protein 1 (Chordc1), mRNA | 0.642 |
| NM\_146468 | Mus musculus olfactory receptor 1391 (Olfr1391), mRNA | 0.642 |
| AJ296079 | Mus musculus mRNA for SCAD family protein. | 0.642 |
| AK040462 | Mus musculus 0 day neonate thymus cDNA, RIKEN full-length enriched library, clone:A430099H23 product:hypothetical Lectin domain of ricin B chain profile containing protein, full insert sequence. | 0.642 |
| NM\_025863 | Mus musculus RIKEN cDNA 2310035M22 gene (2310035M22Rik), mRNA | 0.642 |
| NM\_025788 | Mus musculus BTB (POZ) domain containing 14B (Btbd14b), mRNA | 0.642 |
| BC027371 | Mus musculus RIKEN cDNA 5830411E10 gene, mRNA (cDNA clone IMAGE:4954817), partial cds. | 0.642 |
| AK014534 | Mus musculus 0 day neonate skin cDNA, RIKEN full-length enriched library, clone:4631424J17 product:CDNA FLJ31359 FIS, CLONE MESAN2000501, WEAKLY SIMILAR TO HOMO SAPIENS DNA CYTOSINE METHYLTRANSFERASE 3 ALPHA (DNMT3A) MRNA homolog [Homo sapiens], full in | 0.641 |
| NM\_178118 | Mus musculus DIX domain containing 1 (Dixdc1), mRNA | 0.641 |
| NM\_007527 | Mus musculus Bcl2-associated X protein (Bax), mRNA | 0.641 |
| AK129204 | Mus musculus mRNA for mKIAA0742 protein. | 0.641 |
| AK081873 | Mus musculus 16 days embryo head cDNA, RIKEN full-length enriched library, clone:C130083M23 product:unclassifiable, full insert sequence. | 0.641 |
| NM\_175400 | Mus musculus selenophosphate synthetase 1 (Sephs1), mRNA | 0.641 |
| NM\_025832 | Mus musculus RIKEN cDNA 1300019C06 gene (1300019C06Rik), mRNA | 0.641 |
| NM\_146045 | Mus musculus xylosylprotein beta1,4-galactosyltransferase, polypeptide 7 (galactosyltransferase I) (B4galt7), mRNA | 0.641 |
| XM\_355768 | Mus musculus RIKEN cDNA 2510038N07 gene (2510038N07Rik), mRNA | 0.641 |
| NAP101526-1 | Unknown | 0.640 |
| AK076240 | Mus musculus 14 days embryo liver cDNA, RIKEN full-length enriched library, clone:4432412N19 product:unknown EST, full insert sequence. | 0.640 |
| NM\_211358 | Mus musculus RIKEN cDNA E430007K15 gene (E430007K15Rik), transcript variant 1, mRNA | 0.640 |
| NM\_153787 | Mus musculus BCL2-associated transcription factor 1 (Bclaf1), mRNA | 0.640 |
| XM\_139151 | Mus musculus similar to 40S ribosomal protein S2 (LOC219180), mRNA | 0.640 |
| BC032981 | Mus musculus expressed sequence AW538212, mRNA (cDNA clone MGC:41684 IMAGE:1346965), complete cds. | 0.640 |
| AK088623 | Mus musculus 2 days neonate thymus thymic cells cDNA, RIKEN full-length enriched library, clone:E430021L16 product:neuroblastoma ras oncogene, full insert sequence. | 0.639 |
| X92590 | M.musculus mRNA for HIRA protein. | 0.639 |
| NM\_053159 | Mus musculus mitochondrial ribosomal protein L3 (Mrpl3), mRNA | 0.639 |
| NM\_025888 | Mus musculus RIKEN cDNA 2410004N11 gene (2410004N11Rik), mRNA | 0.639 |
| NM\_178645 | Mus musculus bleomycin hydrolase (Blmh), mRNA | 0.639 |
| NAP058380-1 | Unknown | 0.639 |
| NM\_175152 | Mus musculus RIKEN cDNA 2010013E08 gene (2010013E08Rik), mRNA | 0.639 |
| NAP018615-001 | Unknown | 0.639 |
| NM\_016963 | Mus musculus tropomodulin 3 (Tmod3), mRNA | 0.639 |
| NM\_025479 | Mus musculus RIKEN cDNA 2810021B07 gene (2810021B07Rik), mRNA | 0.639 |
| AK045540 | Mus musculus adult male corpora quadrigemina cDNA, RIKEN full-length enriched library, clone:B230209H17 product:RAP GUANINE NUCLEOTIDE EXCHANGE FACTOR homolog [Homo sapiens], full insert sequence. | 0.639 |
| NAP027922-1 | Unknown | 0.638 |
| NM\_134151 | Mus musculus tyrosyl-tRNA synthetase (Yars), mRNA | 0.638 |
| BC046812 | Mus musculus cytotoxic granule-associated RNA binding protein 1, mRNA (cDNA clone MGC:61396 IMAGE:5702183), complete cds. | 0.638 |
| AK016321 | Mus musculus adult male testis cDNA, RIKEN full-length enriched library, clone:4930579G24 product:hypothetical protein, full insert sequence. | 0.638 |
| U05247 | Mus musculus c-Src kinase (Csk) mRNA, complete cds. | 0.638 |
| NM\_007499 | Mus musculus ataxia telangiectasia mutated homolog (human) (Atm), mRNA | 0.638 |
| NM\_152817 | Mus musculus RIKEN cDNA 2610511O17 gene (2610511O17Rik), mRNA | 0.638 |
| NM\_134041 | Mus musculus RIKEN cDNA 4930427A07 gene (4930427A07Rik), mRNA | 0.638 |
| NM\_178620 | Mus musculus RIKEN cDNA 2600014M03 gene (2600014M03Rik), mRNA | 0.637 |
| XM\_148986 | Mus musculus expressed sequence AI481316 (AI481316), mRNA | 0.637 |
| XM\_193663 | Mus musculus similar to 40S ribosomal protein S2 (LOC268539), mRNA | 0.637 |
| NM\_133768 | Mus musculus argininosuccinate lyase (Asl), mRNA | 0.637 |
| NM\_011519 | Mus musculus syndecan 1 (Sdc1), mRNA | 0.637 |
| AK084912 | Mus musculus 13 days embryo lung cDNA, RIKEN full-length enriched library, clone:D430010N08 product:hypothetical Combined RanBP1/WASP domain , ARM repeat, PHdomain-like containing protein, full insert sequence. | 0.637 |
| AK016065 | Mus musculus adult male testis cDNA, RIKEN full-length enriched library, clone:4930548D04 product:similar to M-PHASE PHOSPHOPROTEIN 9 (FRAGMENT) [Homo sapiens], full insert sequence. | 0.637 |
| BC022907 | Mus musculus amphoterin induced gene and ORF, mRNA (cDNA clone MGC:25558 IMAGE:3980456), complete cds. | 0.637 |
| NM\_013719 | Mus musculus eukaryotic translation initiation factor 2 alpha kinase 4 (Eif2ak4), mRNA | 0.636 |
| AK049228 | Mus musculus ES cells cDNA, RIKEN full-length enriched library, clone:C330014P03 product:RAP GUANINE NUCLEOTIDE EXCHANGE FACTOR homolog [Homo sapiens], full insert sequence. | 0.636 |
| NM\_009533 | Mus musculus X-ray repair complementing defective repair in Chinese hamster cells 5 (Xrcc5), mRNA | 0.635 |
| NM\_153552 | Mus musculus THO complex 1 (Thoc1), mRNA | 0.635 |
| NM\_175113 | Mus musculus RIKEN cDNA 3300001M20 gene (3300001M20Rik), mRNA | 0.635 |
| NM\_028956 | Mus musculus RIKEN cDNA 4933435A13 gene (4933435A13Rik), mRNA | 0.635 |
| NM\_026175 | Mus musculus splicing factor 3a, subunit 1 (Sf3a1), mRNA | 0.635 |
| AK017799 | Mus musculus 8 days embryo whole body cDNA, RIKEN full-length enriched library, clone:5730530J16 product:inferred: RIKEN cDNA 5730530J16 gene, full insert sequence | 0.635 |
| BC006060 | Mus musculus leucyl-tRNA synthetase, mRNA (cDNA clone MGC:8232 IMAGE:3591521), complete cds. | 0.635 |
| NM\_028876 | Mus musculus RIKEN cDNA 4432412D15 gene (4432412D15Rik), mRNA | 0.635 |
| NM\_024242 | Mus musculus RIO kinase 1 (yeast) (Riok1), mRNA | 0.635 |
| NM\_009472 | Mus musculus unc-5 homolog C (C. elegans) (Unc5c), mRNA | 0.635 |
| AK035125 | Mus musculus 12 days embryo embryonic body between diaphragm region and neck cDNA, RIKEN full-length enriched library, clone:9430091J20 product:WD repeat domain 4, full insert sequence | 0.635 |
| AK006099 | Mus musculus adult male testis cDNA, RIKEN full-length enriched library, clone:1700018P20 product:unclassifiable, full insert sequence. | 0.634 |
| NM\_007861 | Mus musculus dihydrolipoamide dehydrogenase (Dld), mRNA | 0.634 |
| NM\_011401 | Mus musculus solute carrier family 2 (facilitated glucose transporter), member 3 (Slc2a3), mRNA | 0.634 |
| AK042783 | Mus musculus 7 days neonate cerebellum cDNA, RIKEN full-length enriched library, clone:A730024C05 product:exportin 4, full insert sequence. | 0.634 |
| AK078769 | Mus musculus 15 days embryo male testis cDNA, RIKEN full-length enriched library, clone:8030467N07 product:DUDULIN 2 homolog [Mus musculus], full insert sequence. | 0.634 |
| NM\_172438 | Mus musculus Fms interacting protein (Fmip), mRNA | 0.633 |
| NM\_172592 | Mus musculus expressed sequence AI450757 (AI450757), mRNA | 0.633 |
| NM\_134255 | Mus musculus ELOVL family member 5, elongation of long chain fatty acids (yeast) (Elovl5), mRNA | 0.633 |
| AK083741 | Mus musculus 9 days embryo whole body cDNA, RIKEN full-length enriched library, clone:D030073N12 product:unknown EST, full insert sequence | 0.633 |
| NM\_010120 | Mus musculus eukaryotic translation initiation factor 1A (Eif1a), mRNA | 0.633 |
| NM\_023633 | Mus musculus RIKEN cDNA 2410016O06 gene (2410016O06Rik), mRNA | 0.632 |
| CA871398 | K0910A09-5N NIA Mouse Neural Stem Cell (Undifferentiated) cDNA Library (Long) Mus musculus cDNA clone NIA:K0910A09 IMAGE:30085160 5'. | 0.632 |
| NM\_025538 | Mus musculus RIKEN cDNA 2310045B01 gene (2310045B01Rik), mRNA | 0.632 |
| NM\_027427 | Mus musculus TAF15 RNA polymerase II, TATA box binding protein (TBP)-associated factor (Taf15), mRNA | 0.632 |
| NM\_016690 | Mus musculus heterogeneous nuclear ribonucleoprotein D-like (Hnrpdl), mRNA | 0.632 |
| AK076719 | Mus musculus adult male testis cDNA, RIKEN full-length enriched library, clone:4930420K17 product:unknown EST, full insert sequence. | 0.632 |
| AK047770 | Mus musculus adult male corpus striatum cDNA, RIKEN full-length enriched library, clone:C030032K12 product:unclassifiable, full insert sequence. | 0.632 |
| NM\_009830 | Mus musculus cyclin E2 (Ccne2), mRNA | 0.632 |
| NM\_008591 | Mus musculus met proto-oncogene (Met), mRNA | 0.631 |
| AK040605 | Mus musculus 0 day neonate thymus cDNA, RIKEN full-length enriched library, clone:A430108K10 product:hypothetical protein, full insert sequence. | 0.631 |
| BC023749 | Mus musculus, clone IMAGE:5354346, mRNA | 0.631 |
| L04649 | Mouse mRNA sequence. | 0.631 |
| XM\_134412 | Mus musculus expressed sequence AA589507 (AA589507), mRNA | 0.631 |
| NM\_012058 | Mus musculus signal recognition particle 9 (Srp9), mRNA | 0.631 |
| AK019507 | Mus musculus 0 day neonate skin cDNA, RIKEN full-length enriched library, clone:4632434B16 product:casein kinase II, alpha 2, polypeptide, full insert sequence. | 0.631 |
| NM\_010356 | Mus musculus glutathione S-transferase, alpha 3 (Gsta3), mRNA | 0.631 |
| U89435 | Mus musculus strain BALB/c unknown mRNA. | 0.630 |
| NM\_011694 | Mus musculus voltage-dependent anion channel 1 (Vdac1), mRNA | 0.630 |
| NM\_024210 | Mus musculus RIKEN cDNA 2310033P09 gene (2310033P09Rik), mRNA | 0.630 |
| NM\_026476 | Mus musculus RIKEN cDNA 2610101N10 gene (2610101N10Rik), mRNA | 0.630 |
| NM\_144802 | Mus musculus RIKEN cDNA 2810036L13 gene (2810036L13Rik), mRNA | 0.630 |
| BC009091 | Mus musculus zinc finger protein 162, mRNA (cDNA clone MGC:7095 IMAGE:3157495), complete cds. | 0.630 |
| NM\_130450 | Mus musculus ELOVL family member 6, elongation of long chain fatty acids (yeast) (Elovl6), mRNA | 0.630 |
| NM\_175351 | Mus musculus RIKEN cDNA C330008L01 gene (C330008L01Rik), mRNA | 0.630 |
| NM\_019512 | Mus musculus transcription elongation regulator 1 (CA150) (Tcerg1), mRNA | 0.630 |
| AK004463 | Mus musculus 18-day embryo whole body cDNA, RIKEN full-length enriched library, clone:1190003B21 product:PHENYLALANYL-TRNA SYNTHETASE ALPHA CHAIN (EC 6.1.1.20) (PHENYLALANINE- -TRNA LIGASE ALPHA CHAIN) (PHERS) (CML33) homolog [Homo sapiens], full insert | 0.630 |
| NM\_134028 | Mus musculus tubulin, gamma 2 (Tubg2), mRNA | 0.630 |
| U73445 | Mus musculus dihydrolipoamide dehydrogenase (Dld) mRNA, complete cds. | 0.630 |
| NM\_025855 | Mus musculus enoyl Coenzyme A hydratase domain containing 1 (Echdc1), mRNA | 0.629 |
| ENSMUST00000046370 | Unknown | 0.629 |
| NM\_030096 | Mus musculus DEAD (Asp-Glu-Ala-Asp) box polypeptide 52 (Ddx52), mRNA | 0.629 |
| AK046880 | Mus musculus 10 days neonate medulla oblongata cDNA, RIKEN full-length enriched library, clone:B830047G01 product:Erbb2 interacting protein, full insert sequence. | 0.629 |
| NM\_017380 | Mus musculus septin 9 (Sept9), mRNA | 0.629 |
| NM\_153556 | Mus musculus postmeiotic segregation increased 1 (S. cerevisiae) (Pms1), mRNA | 0.628 |
| AK122191 | Mus musculus mRNA for mKIAA0029 protein. | 0.628 |
| AK050039 | Mus musculus adult male liver tumor cDNA, RIKEN full-length enriched library, clone:C730006D09 product:hypothetical SNF2 related domain , Helicase c-terminal domain, DEAD/DEAH box helicase containing protein, full insert sequence. | 0.628 |
| XM\_139078 | Mus musculus similar to Nol5a protein (LOC219106), mRNA | 0.628 |
| NM\_025833 | Mus musculus RIKEN cDNA 1300006M19 gene (1300006M19Rik), mRNA | 0.628 |
| NM\_183034 | Mus musculus pleckstrin homology domain containing, family M (with RUN domain) member 1 (Plekhm1), mRNA | 0.627 |
| NM\_172595 | Mus musculus ADP-ribosylation factor related protein 2 (Arfrp2), mRNA | 0.627 |
| NM\_178610 | Mus musculus HIV-1 Rev binding protein 2 (Hrb2), mRNA | 0.627 |
| NM\_028259 | Mus musculus ribosomal protein S6 kinase, polypeptide 1 (Rps6kb1), mRNA | 0.627 |
| NM\_028298 | Mus musculus RIKEN cDNA 2700038I16 gene (2700038I16Rik), mRNA | 0.627 |
| NM\_010748 | Mus musculus lysosomal trafficking regulator (Lyst), mRNA | 0.627 |
| NM\_177586 | Mus musculus eukaryotic translation initiation factor 5A2 (Eif5a2), mRNA | 0.627 |
| NM\_026173 | Mus musculus RIKEN cDNA 1200014M14 gene (1200014M14Rik), mRNA | 0.627 |
| NM\_011391 | Mus musculus solute carrier family 16 (monocarboxylic acid transporters), member 7 (Slc16a7), mRNA | 0.627 |
| BC050855 | Mus musculus expressed sequence AI255170, mRNA (cDNA clone IMAGE:6333217), partial cds. | 0.627 |
| NM\_007916 | Mus musculus DEAD (Asp-Glu-Ala-Asp) box polypeptide 19 (Ddx19), mRNA | 0.627 |
| BC052676 | Mus musculus cDNA clone MGC:60766 IMAGE:30062085, complete cds. | 0.626 |
| NM\_010471 | Mus musculus hippocalcin (Hpca), mRNA | 0.626 |
| NM\_153525 | Mus musculus DNA segment, Chr 7, ERATO Doi 743, expressed (D7Ertd743e), mRNA | 0.626 |
| BC027163 | Mus musculus cDNA clone MGC:36902 IMAGE:4935235, complete cds. | 0.626 |
| NM\_026171 | Mus musculus nuclear VCP-like (Nvl), mRNA | 0.626 |
| AK028699 | Mus musculus 10 days neonate skin cDNA, RIKEN full-length enriched library, clone:4732438L20 product:similar to VERY LONG-CHAIN ACYL-COA SYNTHETASE HOMOLOG 1 [Homo sapiens], full insert sequence | 0.626 |
| BC025453 | Mus musculus cDNA clone IMAGE:5347145, partial cds. | 0.625 |
| NM\_175553 | Mus musculus RIKEN cDNA E030026I10 gene (E030026I10Rik), mRNA | 0.625 |
| AK088223 | Mus musculus 2 days neonate thymus thymic cells cDNA, RIKEN full-length enriched library, clone:E430007J19 product:sorting nexin 5, full insert sequence. | 0.625 |
| NM\_175472 | Mus musculus zinc finger, CCHC domain containing 11 (Zcchc11), mRNA | 0.625 |
| NM\_153065 | Mus musculus DEAD (Asp-Glu-Ala-Asp) box polypeptide 27 (Ddx27), mRNA | 0.625 |
| NM\_198033 | Mus musculus expressed sequence AW060766 (AW060766), mRNA | 0.625 |
| NM\_009319 | Mus musculus TAR (HIV) RNA binding protein 2 (Tarbp2), mRNA | 0.625 |
| NM\_139149 | Mus musculus fusion, derived from t(12;16) malignant liposarcoma (human) (Fus), mRNA | 0.625 |
| BC021408 | Mus musculus RIKEN cDNA 9030416H16 gene, mRNA (cDNA clone MGC:29439 IMAGE:3964501), complete cds. | 0.625 |
| NM\_007794 | Mus musculus CCCTC-binding factor (Ctcf), mRNA | 0.624 |
| NM\_019652 | Mus musculus arsA (bacterial) arsenite transporter, ATP-binding, homolog 1 (Asna1), mRNA | 0.624 |
| NM\_025390 | Mus musculus processing of precursor 4, ribonuclease P/MRP family, (S. cerevisiae) (Pop4), mRNA | 0.624 |
| NM\_181328 | Mus musculus solute carrier family 25 (mitochondrial carrier, palmitoylcarnitine transporter), member 29 (Slc25a29), mRNA | 0.624 |
| BC027640 | Mus musculus mRNA similar to methylcrotonoyl-Coenzyme A carboxylase 2 (beta) (cDNA clone MGC:41339 IMAGE:1244820), complete cds. | 0.624 |
| NM\_145605 | Mus musculus kelch domain containing 4 (Klhdc4), mRNA | 0.624 |
| NM\_207010 | Mus musculus MAM domain containing 1 (Mamdc1), mRNA | 0.624 |
| NM\_025478 | Mus musculus RIKEN cDNA 2610034N03 gene (2610034N03Rik), mRNA | 0.624 |
| NM\_133775 | Mus musculus RIKEN cDNA 9230117N10 gene (9230117N10Rik), mRNA | 0.624 |
| NM\_010774 | Mus musculus methyl-CpG binding domain protein 4 (Mbd4), mRNA | 0.624 |
| NM\_133966 | Mus musculus TAF5-like RNA polymerase II, p300/CBP-associated factor (PCAF)-associated factor (Taf5l), mRNA | 0.623 |
| NM\_026344 | Mus musculus diptheria toxin resistance protein required for diphthamide biosynthesis (Saccharomyces)-like 2 (Dph2l2), mRNA | 0.623 |
| BC052723 | Mus musculus cDNA clone IMAGE:6811344, with apparent retained intron | 0.623 |
| NM\_177474 | Mus musculus DNA segment, Chr 19, Brigham & Women's Genetics 1357 expressed (D19Bwg1357e), mRNA | 0.623 |
| NM\_198605 | Mus musculus RIKEN cDNA F630043A04 gene (F630043A04Rik), mRNA | 0.623 |
| NM\_172471 | Mus musculus inter-alpha (globulin) inhibitor H5 (Itih5), mRNA | 0.623 |
| NM\_212473 | Mus musculus RIKEN cDNA A930008G19 gene (A930008G19Rik), mRNA | 0.623 |
| AK084821 | Mus musculus 13 days embryo heart cDNA, RIKEN full-length enriched library, clone:D330048D07 product:cadherin 2, full insert sequence. | 0.622 |
| NM\_172413 | Mus musculus RAP2C, member of RAS oncogene family (Rap2c), mRNA | 0.622 |
| TC955797 | U39074 thymopoietin beta {Mus musculus}, complete | 0.622 |
| NM\_145585 | Mus musculus THUMP domain containing 1 (Thumpd1), mRNA | 0.622 |
| NM\_008316 | Mus musculus Hus1 homolog (S. pombe) (Hus1), mRNA | 0.622 |
| AK083711 | Mus musculus 9 days embryo whole body cDNA, RIKEN full-length enriched library, clone:D030069J18 product:unknown EST, full insert sequence. | 0.622 |
| NM\_133678 | Mus musculus RIKEN cDNA 2410004C24 gene (2410004C24Rik), mRNA | 0.622 |
| AK053232 | Mus musculus 0 day neonate lung cDNA, RIKEN full-length enriched library, clone:E030047A07 product:unknown EST, full insert sequence. | 0.621 |
| NM\_178413 | Mus musculus cDNA sequence BC051244 (BC051244), mRNA | 0.621 |
| NM\_027698 | Mus musculus RIKEN cDNA 4933424N09 gene (4933424N09Rik), mRNA | 0.621 |
| NM\_009059 | Mus musculus ral guanine nucleotide dissociation stimulator-like 2 (Rgl2), mRNA | 0.621 |
| NM\_007690 | Mus musculus chromodomain helicase DNA binding protein 1 (Chd1), mRNA | 0.621 |
| NAP057019-1 | Unknown | 0.621 |
| NM\_030257 | Mus musculus cDNA sequence BC003322 (BC003322), mRNA | 0.621 |
| NM\_001001986 | Mus musculus RIKEN cDNA 8430427H17 gene (8430427H17Rik), mRNA | 0.621 |
| AK028342 | Mus musculus 12 days embryo embryonic body below diaphragm region cDNA, RIKEN full-length enriched library, clone:3732415M03 product:hypothetical DEAD/DEAH box helicase containing protein, full insert sequence. | 0.621 |
| AK051765 | Mus musculus 12 days embryo spinal ganglion cDNA, RIKEN full-length enriched library, clone:D130074O18 product:hypothetical protein, full insert sequence. | 0.621 |
| NM\_007597 | Mus musculus calnexin (Canx), mRNA | 0.621 |
| NM\_027399 | Mus musculus six transmembrane epithelial antigen of the prostate (Steap), mRNA | 0.621 |
| NM\_011112 | Mus musculus poly (A) polymerase alpha (Papola), mRNA | 0.621 |
| NM\_011390 | Mus musculus solute carrier family 12, member 7 (Slc12a7), mRNA | 0.620 |
| NM\_198102 | Mus musculus RIKEN cDNA G430041M01 gene (G430041M01Rik), mRNA | 0.620 |
| NM\_011550 | Mus musculus transcription factor-like 4 (Tcfl4), mRNA | 0.620 |
| NM\_013634 | Mus musculus peroxisome proliferator activated receptor binding protein (Pparbp), mRNA | 0.620 |
| NM\_021512 | Mus musculus nucleoporin 160 (Nup160), mRNA | 0.620 |
| AK039142 | Mus musculus adult male hypothalamus cDNA, RIKEN full-length enriched library, clone:A230102B06 product:similar to PRESENILINS ASSOCIATED RHOMBOID-LIKE PROTEIN [Homo sapiens], full insert sequence | 0.620 |
| NM\_009627 | Mus musculus adrenomedullin (Adm), mRNA | 0.620 |
| NM\_029339 | Mus musculus RIKEN cDNA 1700023O11 gene (1700023O11Rik), mRNA | 0.620 |
| BC027063 | Mus musculus 3-hydroxybutyrate dehydrogenase (heart, mitochondrial), mRNA (cDNA clone IMAGE:5051325), partial cds. | 0.620 |
| NM\_144873 | Mus musculus ubiquitin-like, containing PHD and RING finger domains 2 (Uhrf2), mRNA | 0.620 |
| NM\_026277 | Mus musculus RIKEN cDNA 1700021I09 gene (1700021I09Rik), mRNA | 0.620 |
| AK034417 | Mus musculus adult male diencephalon cDNA, RIKEN full-length enriched library, clone:9330188P03 product:unknown EST, full insert sequence. | 0.620 |
| BC050863 | Mus musculus RIKEN cDNA 4930562C03 gene, mRNA (cDNA clone MGC:59488 IMAGE:6329366), complete cds. | 0.619 |
| NM\_010494 | Mus musculus intercellular adhesion molecule 2 (Icam2), mRNA | 0.619 |
| NAP028427-1 | Unknown | 0.619 |
| NM\_007879 | Mus musculus developmentally regulated GTP binding protein 1 (Drg1), mRNA | 0.619 |
| NM\_011866 | Mus musculus phosphodiesterase 10A (Pde10a), mRNA | 0.619 |
| AK016872 | Mus musculus adult male testis cDNA, RIKEN full-length enriched library, clone:4933422M21 product:hypothetical ADP-ribosylation factors family containing protein, full insert sequence. | 0.619 |
| XM\_141680 | Mus musculus similar to Testis derived transcript (LOC245376), mRNA | 0.619 |
| NM\_026168 | Mus musculus RIKEN cDNA 1200009B18 gene (1200009B18Rik), mRNA | 0.618 |
| AK014527 | Mus musculus 0 day neonate skin cDNA, RIKEN full-length enriched library, clone:4631422C13 product:weakly similar to KIAA1569 PROTEIN (FRAGMENT) [Homo sapiens], full insert sequence. | 0.618 |
| NM\_177216 | Mus musculus RIKEN cDNA D630003K02 gene (D630003K02Rik), mRNA | 0.618 |
| XM\_135632 | Mus musculus similar to Eukaryotic translation initiation factor 1A (eIF-1A) (eIF-4C) (LOC236359), mRNA | 0.618 |
| NM\_009812 | Mus musculus caspase 8 (Casp8), mRNA | 0.618 |
| AK011315 | Mus musculus 10 days embryo whole body cDNA, RIKEN full-length enriched library, clone:2610005D18 product:RIKEN cDNA 2610005D18 gene, full insert sequence. | 0.618 |
| NM\_026313 | Mus musculus RIKEN cDNA 3300001P08 gene (3300001P08Rik), mRNA | 0.618 |
| NM\_009536 | Mus musculus tyrosine 3-monooxygenase/tryptophan 5-monooxygenase activation protein, epsilon polypeptide (Ywhae), mRNA | 0.618 |
| BC023136 | Mus musculus sno, strawberry notch homolog 1 (Drosophila), mRNA (cDNA clone IMAGE:3671884), partial cds. | 0.618 |
| NM\_007531 | Mus musculus B-cell receptor-associated protein 37 (Bcap37), mRNA | 0.618 |
| NM\_178677 | Mus musculus SEC22 vesicle trafficking protein-like 3 (S. cerevisiae) (Sec22l3), mRNA | 0.618 |
| NM\_178693 | Mus musculus DNA segment, Chr 2, ERATO Doi 97, expressed (D2Ertd97e), mRNA | 0.618 |
| AK089997 | Mus musculus male submandibular gland CRL-1734 SCA-9 clone 15 cDNA, RIKEN full-length enriched library, clone:G430050D18 product:similar to MITOTIC PHOSPHOPROTEIN 44 [Xenopus laevis], full insert sequence. | 0.618 |
| TC1005843 | Unknown | 0.617 |
| NM\_194257 | Mus musculus RIKEN cDNA C030004A17 gene (C030004A17Rik), transcript variant 1, mRNA | 0.617 |
| BC055777 | Mus musculus nuclear cap binding protein subunit 1, 80kDa, mRNA (cDNA clone IMAGE:6402922). | 0.617 |
| AK048970 | Mus musculus 0 day neonate cerebellum cDNA, RIKEN full-length enriched library, clone:C230086M12 product:unknown EST, full insert sequence. | 0.617 |
| NM\_177873 | Mus musculus RIKEN cDNA A330009G12 gene (A330009G12Rik), mRNA | 0.617 |
| NM\_172262 | Mus musculus amine oxidase, flavin containing 1 (Aof1), mRNA | 0.617 |
| NM\_177884 | Mus musculus expressed sequence AW146020 (AW146020), mRNA | 0.617 |
| NM\_021888 | Mus musculus queuine tRNA-ribosyltransferase 1 (Qtrt1), mRNA | 0.617 |
| AK035203 | Mus musculus 12 days embryo embryonic body between diaphragm region and neck cDNA, RIKEN full-length enriched library, clone:9430099P11 product:unclassifiable, full insert sequence | 0.617 |
| AK043419 | Mus musculus 7 days neonate cerebellum cDNA, RIKEN full-length enriched library, clone:A730094G16 product:unspliced dna for: PROLIFERATION RELATED ACIDIC LEUCINE RICH PROTEIN PAL31 (SIMILAR TO ACIDIC PROTEIN RICH IN LEUCINES) homolog [Mus musculus], .. | 0.617 |
| NM\_134037 | Mus musculus ATP citrate lyase (Acly), mRNA | 0.617 |
| NM\_182990 | Mus musculus structure specific recognition protein 1 (Ssrp1), mRNA | 0.617 |
| AK037232 | Mus musculus 6 days neonate skin cDNA, RIKEN full-length enriched library, clone:A030012C10 product:unknown EST, full insert sequence. | 0.617 |
| NM\_011479 | Mus musculus serine palmitoyltransferase, long chain base subunit 2 (Sptlc2), mRNA | 0.617 |
| NM\_017475 | Mus musculus Ras-related GTP binding C (Rragc), mRNA | 0.617 |
| AK048462 | Mus musculus 16 days embryo head cDNA, RIKEN full-length enriched library, clone:C130062I06 product:similar to FIBRILLARIN (NUCLEOLAR PROTEIN 1) [Mus musculus], full insert sequence. | 0.617 |
| AK081982 | Mus musculus 16 days embryo head cDNA, RIKEN full-length enriched library, clone:C130091B22 product:EUCARYOTIC TRANSLATION INITIATION FACTOR 4G ISOFORM 2 (FRAGMENT) homolog [Homo sapiens], full insert sequence. | 0.616 |
| NM\_198023 | Mus musculus DNA segment, Chr 12, Wayne State University 95, expressed (D12Wsu95e), mRNA | 0.616 |
| AK011739 | Mus musculus 10 days embryo whole body cDNA, RIKEN full-length enriched library, clone:2610042E16 product:centromere autoantigen H, full insert sequence. | 0.616 |
| NM\_028959 | Mus musculus RIKEN cDNA 4933440J22 gene (4933440J22Rik), mRNA | 0.616 |
| NM\_199299 | Mus musculus PHD finger protein 15 (Phf15), mRNA | 0.616 |
| NM\_011408 | Mus musculus schlafen 2 (Slfn2), mRNA | 0.616 |
| NM\_054070 | Mus musculus AFG3(ATPase family gene 3)-like 1 (yeast) (Afg3l1), mRNA | 0.616 |
| NAP101659-1 | Unknown | 0.616 |
| NM\_177367 | Mus musculus gem (nuclear organelle) associated protein 4 (Gemin4), mRNA | 0.616 |
| TC1012118 | CUL3\_MOUSE Cullin homolog 3 (CUL-3). [Mouse] {Mus musculus}, complete | 0.616 |
| NM\_025588 | Mus musculus SEC5-like 1 (S. cerevisiae) (Sec5l1), mRNA | 0.616 |
| NM\_177691 | Mus musculus expressed sequence C79127 (C79127), mRNA | 0.616 |
| AK041455 | Mus musculus 3 days neonate thymus cDNA, RIKEN full-length enriched library, clone:A630011J05 product:unknown EST, full insert sequence. | 0.616 |
| TC994428 | X83413 U88 {Human herpesvirus 6}, partial (7%) | 0.615 |
| NM\_027371 | Mus musculus RIKEN cDNA 2310066N05 gene (2310066N05Rik), mRNA | 0.615 |
| BC002236 | Mus musculus cDNA sequence BC002236, mRNA (cDNA clone IMAGE:3491659), complete cds. | 0.615 |
| AK042548 | Mus musculus 7 days neonate cerebellum cDNA, RIKEN full-length enriched library, clone:A730004A21 product:Similar to nuclear matrix protein p84, full insert sequence. | 0.615 |
| NM\_153566 | Mus musculus cDNA sequence BC023823 (BC023823), mRNA | 0.615 |
| BC064713 | Mus musculus RIKEN cDNA 4922501C03 gene, mRNA (cDNA clone MGC:74349 IMAGE:30248844), complete cds. | 0.615 |
| NM\_013498 | Mus musculus cAMP responsive element modulator (Crem), mRNA | 0.615 |
| AK037109 | Mus musculus adult female vagina cDNA, RIKEN full-length enriched library, clone:9930116O05 product:hypothetical protein, full insert sequence. | 0.615 |
| AK086861 | Mus musculus 0 day neonate lung cDNA, RIKEN full-length enriched library, clone:E030006G18 product:unknown EST, full insert sequence. | 0.615 |
| AI553296 | vi29d03.y1 Barstead mouse irradiated colon MPLRB7 Mus musculus cDNA clone IMAGE:905189 5'. | 0.614 |
| NAP113002-1 | Unknown | 0.614 |
| NM\_011751 | Mus musculus zinc finger protein 207 (Zfp207), mRNA | 0.614 |
| NAP029477-1 | Unknown | 0.614 |
| NM\_027919 | Mus musculus RIKEN cDNA 1300017K07 gene (1300017K07Rik), mRNA | 0.614 |
| AK009476 | Mus musculus adult male tongue cDNA, RIKEN full-length enriched library, clone:2310022K01 product:hypothetical RNA-binding region RNP-1 (RNA recognition motif) containing protein, full insert sequence. | 0.614 |
| NM\_033074 | Mus musculus threonyl-tRNA synthetase (Tars), mRNA | 0.614 |
| BC027166 | Mus musculus eukaryotic translation initiation factor 4, gamma 1, mRNA (cDNA clone IMAGE:4950789), partial cds. | 0.613 |
| NM\_024260 | Mus musculus RIKEN cDNA 1700034M03 gene (1700034M03Rik), mRNA | 0.613 |
| NM\_008179 | Mus musculus G1 to phase transition 2 (Gspt2), mRNA | 0.613 |
| AK034793 | Mus musculus 12 days embryo embryonic body between diaphragm region and neck cDNA, RIKEN full-length enriched library, clone:9430039I15 product:nuclear factor I/B, full insert sequence. | 0.612 |
| NM\_023323 | Mus musculus brix domain containing 1 (Bxdc1), mRNA | 0.612 |
| NM\_133188 | Mus musculus DAZ associated protein 1 (Dazap1), mRNA | 0.612 |
| AK012045 | Mus musculus 10 days embryo whole body cDNA, RIKEN full-length enriched library, clone:2610318I15 product:unknown EST, full insert sequence. | 0.612 |
| NM\_207219 | Mus musculus expressed sequence AI314976 (AI314976), mRNA | 0.612 |
| AK012959 | Mus musculus 10, 11 days embryo whole body cDNA, RIKEN full-length enriched library, clone:2810401C22 product:hypothetical Cytochrome c family heme-binding site/Zinc finger, C2H2 type containing protein, full insert sequence. | 0.612 |
| AK042495 | Mus musculus 3 days neonate thymus cDNA, RIKEN full-length enriched library, clone:A630096I15 product:unclassifiable, full insert sequence. | 0.612 |
| NM\_018864 | Mus musculus inositol (myo)-1(or 4)-monophosphatase 1 (Impa1), mRNA | 0.612 |
| NM\_172757 | Mus musculus RIKEN cDNA C030036P15 gene (C030036P15Rik), mRNA | 0.611 |
| BC031756 | Mus musculus cDNA clone IMAGE:4222597, with apparent retained intron. | 0.611 |
| AK079112 | Mus musculus 12 days embryo embryonic body between diaphragm region and neck cDNA, RIKEN full-length enriched library, clone:9430010A17 product:IL-5 PROMOTER REII-REGION-BINDING PROTEIN homolog [Homo sapiens], full insert sequence. | 0.611 |
| NM\_029977 | Mus musculus polymerase (DNA directed), theta (Polq), mRNA | 0.611 |
| BC058590 | Mus musculus cDNA clone IMAGE:5361444, partial cds | 0.611 |
| BC046402 | Mus musculus, Similar to fringe connection, clone IMAGE:5355469, mRNA, partial cds. | 0.611 |
| NM\_175090 | Mus musculus solute carrier family 31, member 1 (Slc31a1), mRNA | 0.611 |
| NM\_153785 | Mus musculus cyclin-dependent kinase-like 3 (Cdkl3), mRNA | 0.611 |
| NM\_013770 | Mus musculus solute carrier family 25 (mitochondrial carrier, dicarboxylate transporter), member 10 (Slc25a10), mRNA | 0.611 |
| BC010727 | Mus musculus splicing factor 3a, subunit 1, mRNA (cDNA clone IMAGE:3707947), complete cds. | 0.611 |
| NM\_025922 | Mus musculus inosine triphosphatase (nucleoside triphosphate pyrophosphatase) (Itpa), mRNA | 0.611 |
| AK044132 | Mus musculus 10 days neonate cortex cDNA, RIKEN full-length enriched library, clone:A830093E13 product:hypothetical protein, full insert sequence. | 0.611 |
| NM\_211355 | Mus musculus RIKEN cDNA 1110034C04 gene (1110034C04Rik), mRNA | 0.610 |
| NM\_146222 | Mus musculus cDNA sequence BC024479 (BC024479), mRNA | 0.610 |
| NM\_019411 | Mus musculus protein phosphatase 2a, catalytic subunit, alpha isoform (Ppp2ca), mRNA | 0.610 |
| AK006314 | Mus musculus adult male testis cDNA, RIKEN full-length enriched library, clone:1700024P20 product:spermatid perinuclear RNA binding protein, full insert sequence. | 0.610 |
| NM\_010455 | Mus musculus homeo box A7 (Hoxa7), mRNA | 0.610 |
| NM\_009033 | Mus musculus RNA binding motif protein, X chromosome retrogene (Rbmxrt), mRNA | 0.610 |
| NM\_172726 | Mus musculus RIKEN cDNA E130309D02 gene (E130309D02Rik), mRNA | 0.610 |
| NM\_027494 | Mus musculus zinc finger, CCHC domain containing 8 (Zcchc8), mRNA | 0.610 |
| NM\_030210 | Mus musculus acetoacetyl-CoA synthetase (Aacs), mRNA | 0.610 |
| TC968012 | Unknown | 0.609 |
| NM\_008253 | Mus musculus high mobility group box 3 (Hmgb3), mRNA | 0.609 |
| NM\_029852 | Mus musculus RIKEN cDNA 4921537D05 gene (4921537D05Rik), mRNA | 0.609 |
| BC049261 | Mus musculus DEAD (Asp-Glu-Ala-Asp) box polypeptide 10, mRNA (cDNA clone IMAGE:5364672), partial cds. | 0.609 |
| AK006112 | Mus musculus adult male testis cDNA, RIKEN full-length enriched library, clone:1700019D06 product:STRETCH RESPONSIVE PROTEIN 278 (FRAGMENT) homolog [Mus musculus], full insert sequence. | 0.609 |
| AK036079 | Mus musculus 16 days neonate cerebellum cDNA, RIKEN full-length enriched library, clone:9630032O13 product:unknown EST, full insert sequence | 0.609 |
| AK089746 | Mus musculus activated spleen cDNA, RIKEN full-length enriched library, clone:F830014G06 product:Tumor necrosis factor-like weak inducer of apoptosis-Receptor, full insert sequence. | 0.609 |
| BC003206 | Mus musculus RIKEN cDNA 1300006C19 gene, mRNA (cDNA clone IMAGE:3587254), partial cds. | 0.609 |
| AK003687 | Mus musculus 18-day embryo whole body cDNA, RIKEN full-length enriched library, clone:1110014E10 product:unknown EST, full insert sequence. | 0.609 |
| NM\_197982 | Mus musculus DEAD (Asp-Glu-Ala-Asp) box polypeptide 39 (Ddx39), mRNA | 0.608 |
| NM\_013792 | Mus musculus alpha-N-acetylglucosaminidase (Sanfilippo disease IIIB) (Naglu), mRNA | 0.608 |
| NM\_145610 | Mus musculus peter pan homolog (Drosophila) (Ppan), mRNA | 0.608 |
| NM\_024212 | Mus musculus ribosomal protein L4 (Rpl4), mRNA | 0.608 |
| NM\_146151 | Mus musculus testis-specific kinase 2 (Tesk2), mRNA | 0.608 |
| AK032547 | Mus musculus adult male olfactory brain cDNA, RIKEN full-length enriched library, clone:6430590C21 product:PLATELET ACTIVATING FACTOR RECEPTOR, full insert sequence. | 0.607 |
| NM\_011365 | Mus musculus SH3 domain protein 1B (Sh3d1B), mRNA | 0.607 |
| NM\_145457 | Mus musculus polyadenylate binding protein-interacting protein 1 (Paip1), mRNA | 0.607 |
| NM\_009211 | Mus musculus SWI/SNF related, matrix associated, actin dependent regulator of chromatin, subfamily c, member 1 (Smarcc1), mRNA | 0.607 |
| AK013903 | Mus musculus 12 days embryo head cDNA, RIKEN full-length enriched library, clone:3010033P07 product:SIMILAR TO RIBOSOMAL PROTEIN S9 (UNKNOWN) (PROTEIN FOR MGC:14341) (PROTEIN FOR MGC:2458) (PROTEIN FOR MGC:4138) homolog [Homo sapiens], full insert seque | 0.607 |
| BC006867 | Mus musculus testis expressed gene 10, mRNA (cDNA clone MGC:11792 IMAGE:3595167), complete cds. | 0.607 |
| AK083899 | Mus musculus 12 days embryo spinal ganglion cDNA, RIKEN full-length enriched library, clone:D130055P07 product:RNA binding motif protein, X chromosome, full insert sequence | 0.607 |
| NM\_133916 | Mus musculus eukaryotic translation initiation factor 3, subunit 9 (eta) (Eif3s9), mRNA | 0.607 |
| NM\_182995 | Mus musculus RIKEN cDNA 6330503K22 gene (6330503K22Rik), mRNA | 0.607 |
| NM\_008575 | Mus musculus transformed mouse 3T3 cell double minute 4 (Mdm4), mRNA | 0.607 |
| NAP057035-1 | Unknown | 0.607 |
| NM\_011636 | Mus musculus phospholipid scramblase 1 (Plscr1), mRNA | 0.606 |
| NM\_019501 | Mus musculus trans-prenyltransferase (Tprt), mRNA | 0.606 |
| NM\_010561 | Mus musculus interleukin enhancer binding factor 3 (Ilf3), mRNA | 0.606 |
| NM\_023042 | Mus musculus RecQ protein-like (Recql), mRNA | 0.606 |
| AK036089 | Mus musculus 16 days neonate cerebellum cDNA, RIKEN full-length enriched library, clone:9630033P22 product:weakly similar to HYPOTHETICAL 33.8 KDA PROTEIN [Homo sapiens], full insert sequence. | 0.606 |
| NM\_175310 | Mus musculus androgen-induced proliferation inhibitor (Aprin), mRNA | 0.606 |
| NM\_010709 | Mus musculus ligatin (Lgtn), mRNA | 0.605 |
| NM\_026218 | Mus musculus FGFR1 oncogene partner 2 (Fgfr1op2), mRNA | 0.605 |
| NAP043218-1 | Unknown | 0.605 |
| NM\_010442 | Mus musculus heme oxygenase (decycling) 1 (Hmox1), mRNA | 0.605 |
| XM\_127336 | Mus musculus RIKEN cDNA 2810012K13 gene (2810012K13Rik), mRNA | 0.605 |
| BC033915 | Mus musculus cDNA sequence BC033915, mRNA (cDNA clone IMAGE:1264225), partial cds. | 0.605 |
| NM\_144786 | Mus musculus gamma-glutamyltransferase-like 3 (Ggtl3), mRNA | 0.605 |
| AK008884 | Mus musculus adult male stomach cDNA, RIKEN full-length enriched library, clone:2210410E06 product:hypothetical protein, full insert sequence. | 0.605 |
| NM\_133771 | Mus musculus RIKEN cDNA 0610016J10 gene (0610016J10Rik), mRNA | 0.604 |
| NM\_199467 | Mus musculus RIKEN cDNA F730047E07 gene (F730047E07Rik), mRNA | 0.604 |
| NM\_028233 | Mus musculus leucine-rich PPR-motif containing (Lrpprc), mRNA | 0.604 |
| NM\_010721 | Mus musculus lamin B1 (Lmnb1), mRNA | 0.604 |
| NM\_025652 | Mus musculus general transcription factor III A (Gtf3a), mRNA | 0.604 |
| NM\_144528 | Mus musculus ring finger protein 126 (Rnf126), mRNA | 0.604 |
| NM\_198899 | Mus musculus UDP-glucose ceramide glucosyltransferase-like 1 (Ugcgl1), mRNA | 0.604 |
| AF064782 | Mus musculus clone pEN87 unknown mRNA, partial cds. | 0.604 |
| NM\_172748 | Mus musculus cDNA sequence BC059812 (BC059812), mRNA | 0.603 |
| AK010665 | Mus musculus ES cells cDNA, RIKEN full-length enriched library, clone:2410044A07 product:hypothetical Cysteinyl-tRNA synthetase containing protein, full insert sequence. | 0.603 |
| NM\_175405 | Mus musculus RIKEN cDNA A930024E05 gene (A930024E05Rik), mRNA | 0.603 |
| AK129031 | Mus musculus mRNA for mKIAA0007 protein. | 0.603 |
| NM\_008186 | Mus musculus general transcription factor II H, polypeptide 1 (Gtf2h1), mRNA | 0.603 |
| NM\_016897 | Mus musculus translocase of inner mitochondrial membrane 23 homolog (yeast) (Timm23), mRNA | 0.603 |
| XM\_125510 | Mus musculus RIKEN cDNA 3110003A17 gene (3110003A17Rik), mRNA | 0.603 |
| NM\_009085 | Mus musculus RNA polymerase 1-1 (Rpo1-1), mRNA | 0.603 |
| U91923 | Mus musculus unknown protein mRNA, partial cds. | 0.603 |
| NM\_008263 | Mus musculus homeo box A10 (Hoxa10), mRNA | 0.603 |
| AK040611 | Mus musculus 0 day neonate thymus cDNA, RIKEN full-length enriched library, clone:A430109D20 product:myeloid ecotropic viral integration site-related gene 1, full insert sequence | 0.603 |
| NM\_144904 | Mus musculus ROD1 regulator of differentiation 1 (S. pombe) (Rod1), mRNA | 0.602 |
| AK015293 | Mus musculus adult male testis cDNA, RIKEN full-length enriched library, clone:4930432K21 product:hypothetical protein, full insert sequence. | 0.602 |
| XM\_130319 | Mus musculus RIKEN cDNA 2410066K11 gene (2410066K11Rik), mRNA | 0.602 |
| AK012919 | Mus musculus 10, 11 days embryo whole body cDNA, RIKEN full-length enriched library, clone:2810047L02 product:L2DTL PROTEIN (RA-REGULATED NUCLEAR MATRIX-ASSOCIATED PROTEIN) homolog [Homo sapiens], full insert sequence. | 0.602 |
| NM\_027911 | Mus musculus RIKEN cDNA 1300006N24 gene (1300006N24Rik), mRNA | 0.602 |
| NM\_028752 | Mus musculus RIKEN cDNA 0610039P13 gene (0610039P13Rik), mRNA | 0.602 |
| NM\_133888 | Mus musculus sphingomyelin phosphodiesterase, acid-like 3B (Smpdl3b), mRNA | 0.601 |
| BC068113 | Mus musculus testis expressed gene 292, mRNA (cDNA clone MGC:92952 IMAGE:5707865), complete cds. | 0.601 |
| NM\_175186 | Mus musculus RIKEN cDNA 2700022J23 gene (2700022J23Rik), mRNA | 0.601 |
| NM\_007727 | Mus musculus contactin 1 (Cntn1), mRNA | 0.601 |
| NM\_013509 | Mus musculus enolase 2, gamma neuronal (Eno2), mRNA | 0.601 |
| AK053606 | Mus musculus 0 day neonate eyeball cDNA, RIKEN full-length enriched library, clone:E130113K22 product:hypothetical FYVE/PHD zinc finger structure containing protein, full insert sequence. | 0.601 |
| NAP028877-1 | Unknown | 0.600 |
| AK079340 | Mus musculus 16 days neonate cerebellum cDNA, RIKEN full-length enriched library, clone:9630030I15 product:inferred: heat shock factor 2 {Mus musculus}, full insert sequence. | 0.600 |
| NM\_146215 | Mus musculus cDNA sequence BC025546 (BC025546), mRNA | 0.600 |
| NM\_177752 | Mus musculus hypothetical protein 6820428D13 (6820428D13), mRNA | 0.600 |
| NM\_010825 | Mus musculus myeloid ecotropic viral integration site-related gene 1 (Mrg1), mRNA | 0.600 |
| AK006434 | Mus musculus adult male testis cDNA, RIKEN full-length enriched library, clone:1700027L20 product:inferred: RIKEN cDNA 1700027L20 gene / putative [Mus musculus], full insert sequence. | 0.599 |
| NM\_133787 | Mus musculus expressed sequence C87860 (C87860), mRNA | 0.599 |
| NM\_009832 | Mus musculus cyclin K (Ccnk), mRNA | 0.599 |
| NM\_198127 | Mus musculus abl-interactor 2 (Abi2), mRNA | 0.599 |
| NM\_008787 | Mus musculus pericentrin 2 (Pcnt2), mRNA | 0.599 |
| NM\_183392 | Mus musculus RIKEN cDNA 3110079L04 gene (3110079L04Rik), mRNA | 0.598 |
| AK011415 | Mus musculus 10 days embryo whole body cDNA, RIKEN full-length enriched library, clone:2610016F04 product:unknown EST, full insert sequence. | 0.598 |
| BC030460 | Mus musculus cDNA clone MGC:40698 IMAGE:4946146, complete cds. | 0.598 |
| NAP115295-1 | Unknown | 0.598 |
| BC052463 | Mus musculus inositol polyphosphate multikinase, mRNA (cDNA clone MGC:63346 IMAGE:6834748), complete cds. | 0.598 |
| NM\_026531 | Mus musculus RIKEN cDNA 2700083B06 gene (2700083B06Rik), mRNA | 0.598 |
| AK077693 | Mus musculus 8 days embryo whole body cDNA, RIKEN full-length enriched library, clone:5730526A15 product:TRANSMEMBRANE PROTEIN H4 homolog [Homo sapiens], full insert sequence. | 0.598 |
| NM\_178599 | Mus musculus COMM domain containing 8 (Commd8), mRNA | 0.598 |
| AK050851 | Mus musculus 9 days embryo whole body cDNA, RIKEN full-length enriched library, clone:D030026B04 product:unknown EST, full insert sequence. | 0.598 |
| NM\_009829 | Mus musculus cyclin D2 (Ccnd2), mRNA | 0.598 |
| NM\_134034 | Mus musculus expressed sequence AW011752 (AW011752), mRNA | 0.597 |
| AK045715 | Mus musculus adult male corpora quadrigemina cDNA, RIKEN full-length enriched library, clone:B230307D21 product:unknown EST, full insert sequence. | 0.597 |
| NAP093999-001 | Unknown | 0.597 |
| BC045204 | Mus musculus RIKEN cDNA 1110054H05 gene, mRNA (cDNA clone IMAGE:5376424), partial cds. | 0.597 |
| NM\_010877 | Mus musculus neutrophil cytosolic factor 2 (Ncf2), mRNA | 0.597 |
| NM\_019766 | Mus musculus telomerase binding protein, p23 (Tebp), mRNA | 0.597 |
| NM\_010324 | Mus musculus glutamate oxaloacetate transaminase 1, soluble (Got1), mRNA | 0.597 |
| NM\_133796 | Mus musculus Rho GDP dissociation inhibitor (GDI) alpha (Arhgdia), mRNA | 0.596 |
| NM\_025893 | Mus musculus RIKEN cDNA 1500031H04 gene (1500031H04Rik), mRNA | 0.596 |
| NM\_023223 | Mus musculus cell division cycle 20 homolog (S. cerevisiae) (Cdc20), mRNA | 0.596 |
| NM\_025879 | Mus musculus RIKEN cDNA 2410002O22 gene (2410002O22Rik), mRNA | 0.596 |
| NM\_133777 | Mus musculus ubiquitin-conjugating enzyme E2S (Ube2s), mRNA | 0.595 |
| AK122296 | Mus musculus mRNA for mKIAA0488 protein. | 0.595 |
| NM\_177682 | Mus musculus expressed sequence AU022870 (AU022870), mRNA | 0.595 |
| AV241894 | AV241894 RIKEN full-length enriched, 0 day neonate head Mus musculus cDNA clone 4831401G08 3'. | 0.595 |
| NM\_194339 | Mus musculus BMS1-like, ribosome assembly protein (yeast) (Bms1l), mRNA | 0.595 |
| NM\_027355 | Mus musculus RIKEN cDNA 3110001H15 gene (3110001H15Rik), mRNA | 0.594 |
| X14206 | Mouse mRNA for poly (ADP-ribose) polymerase (EC 2.4.2.30). | 0.594 |
| NM\_019836 | Mus musculus RIKEN cDNA 2610024G14 gene (2610024G14Rik), mRNA | 0.594 |
| NM\_148932 | Mus musculus nuclear pore membrane protein 121 (Pom121), mRNA | 0.594 |
| NM\_025443 | Mus musculus RIKEN cDNA 1810003N24 gene (1810003N24Rik), mRNA | 0.594 |
| AK047448 | Mus musculus 10 days neonate cerebellum cDNA, RIKEN full-length enriched library, clone:B930063P07 product:unknown EST, full insert sequence. | 0.594 |
| NM\_133800 | Mus musculus expressed sequence C78541 (C78541), mRNA | 0.593 |
| NM\_025904 | Mus musculus RIKEN cDNA 1600012F09 gene (1600012F09Rik), mRNA | 0.593 |
| NM\_011906 | Mus musculus transmembrane domain protein regulated in adipocytes (Tpra40), mRNA | 0.593 |
| NM\_021465 | Mus musculus stromal antigen 2 (Stag2), mRNA | 0.593 |
| AK010292 | Mus musculus ES cells cDNA, RIKEN full-length enriched library, clone:2400006P09 product:RIBONUCLEASE HI LARGE SUBUNIT (EC 3.1.26.-) (RNASE HI LARGE SUBUNIT) (RNASE H(35)) (RIBONUCLEASE H2) (RNASE H2) homolog [Homo sapiens], full insert sequence. [AK010 | 0.593 |
| BE985144 | UI-M-CG0p-bdi-a-04-0-UI.s1 NIH\_BMAP\_Ret4\_S2 Mus musculus cDNA clone UI-M-CG0p-bdi-a-04-0-UI 3'. | 0.593 |
| XM\_146154 | Mus musculus similar to 40S ribosomal protein S2 (LOC244229), mRNA | 0.592 |
| NM\_144852 | Mus musculus solute carrier family 7 (cationic amino acid transporter, y+ system), member 4 (Slc7a4), mRNA | 0.592 |
| NM\_011657 | Mus musculus tubby-like protein 3 (Tulp3), mRNA | 0.592 |
| NM\_007636 | Mus musculus chaperonin subunit 2 (beta) (Cct2), mRNA | 0.592 |
| BC047067 | Mus musculus, Similar to RIKEN cDNA 5730521P14 gene, clone IMAGE:5036926, mRNA, partial cds. | 0.592 |
| NM\_011650 | Mus musculus translin (Tsn), mRNA | 0.592 |
| AK038651 | Mus musculus adult male hypothalamus cDNA, RIKEN full-length enriched library, clone:A230052H18 product:hypothetical protein, full insert sequence. | 0.592 |
| NM\_018760 | Mus musculus solute carrier family 4 (anion exchanger), member 4 (Slc4a4), mRNA | 0.592 |
| NM\_016881 | Mus musculus phosphomannomutase 2 (Pmm2), mRNA | 0.592 |
| NM\_023671 | Mus musculus chloride channel, nucleotide-sensitive, 1A (Clns1a), mRNA | 0.591 |
| AK005395 | Mus musculus adult female placenta cDNA, RIKEN full-length enriched library, clone:1600002H07 product:hypothetical protein, full insert sequence. | 0.591 |
| AK089965 | Mus musculus embryo RCB-0549 Cle-H3 cDNA, RIKEN full-length enriched library, clone:G430031O03 product:coated vesicle membrane protein, full insert sequence. | 0.591 |
| NM\_026377 | Mus musculus RIKEN cDNA 6330577E15 gene (6330577E15Rik), mRNA | 0.591 |
| NM\_177715 | Mus musculus potassium channel tetramerisation domain containing 12 (Kctd12), mRNA | 0.591 |
| NM\_025520 | Mus musculus LSM5 homolog, U6 small nuclear RNA associated (S. cerevisiae) (Lsm5), mRNA | 0.591 |
| NM\_013664 | Mus musculus SH3-domain GRB2-like 1 (Sh3gl1), mRNA | 0.590 |
| AK014663 | Mus musculus 0 day neonate head cDNA, RIKEN full-length enriched library, clone:4833408C14 product:unknown EST, full insert sequence. | 0.590 |
| NM\_028844 | Mus musculus apoptosis, caspase activation inhibitor (Aven), mRNA | 0.590 |
| NM\_018870 | Mus musculus phosphoglycerate mutase 2 (Pgam2), mRNA | 0.590 |
| NM\_025927 | Mus musculus mitochondrial ribosomal protein L45 (Mrpl45), mRNA | 0.590 |
| NM\_011247 | Mus musculus retinoblastoma binding protein 6 (Rbbp6), mRNA | 0.590 |
| BC051474 | Mus musculus mRNA similar to RAS, guanyl releasing protein 2 (cDNA clone MGC:62910 IMAGE:1179744), complete cds. | 0.590 |
| NM\_172507 | Mus musculus RIKEN cDNA A930014C21 gene (A930014C21Rik), mRNA | 0.589 |
| NAP027441-1 | Unknown | 0.589 |
| NM\_008714 | Mus musculus Notch gene homolog 1 (Drosophila) (Notch1), mRNA | 0.589 |
| NM\_180588 | Mus musculus RIKEN cDNA 2700029E10 gene (2700029E10Rik), mRNA | 0.589 |
| NM\_026842 | Mus musculus ubiquilin 1 (Ubqln1), transcript variant 1, mRNA | 0.589 |
| NM\_021525 | Mus musculus RNA terminal phosphate cyclase-like 1 (Rcl1), mRNA | 0.589 |
| AK005177 | Mus musculus adult male cerebellum cDNA, RIKEN full-length enriched library, clone:1500008C05 product:unknown EST, full insert sequence. | 0.589 |
| BC064013 | Mus musculus cDNA clone MGC:70135 IMAGE:6515698, complete cds. | 0.589 |
| NM\_018889 | Mus musculus phosphatidylinositol glycan, class B (Pigb), mRNA | 0.589 |
| NM\_198299 | Mus musculus RIKEN cDNA E130303B06 gene (E130303B06Rik), mRNA | 0.588 |
| BC068133 | Mus musculus cDNA clone MGC:92946 IMAGE:6813785, complete cds. | 0.588 |
| BY715341 | BY715341 RIKEN full-length enriched, adult male testis Mus musculus cDNA clone 4930515G16 5'. | 0.588 |
| NM\_011121 | Mus musculus polo-like kinase 1 (Drosophila) (Plk1), mRNA | 0.588 |
| NM\_013727 | Mus musculus 5-azacytidine induced gene 2 (Azi2), mRNA | 0.588 |
| NM\_145972 | Mus musculus cDNA sequence BC027231 (BC027231), mRNA | 0.588 |
| AK036084 | Mus musculus 16 days neonate cerebellum cDNA, RIKEN full-length enriched library, clone:9630033H11 product:INOSITOL POLYPHOSPHATE MULTIKINASE homolog [Rattus norvegicus], full insert sequence. | 0.587 |
| BC049240 | Mus musculus ligase III, DNA, ATP-dependent, mRNA (cDNA clone MGC:54652 IMAGE:4504384), complete cds. | 0.587 |
| AK006658 | Mus musculus adult male testis cDNA, RIKEN full-length enriched library, clone:1700040I03 product:hypothetical Protease associated (PA) domain containing protein, full insert sequence. | 0.587 |
| AF193344 | Mus musculus GCN2gamma mRNA, complete cds. | 0.587 |
| NM\_146014 | Mus musculus cerebral cavernous malformation 2 homolog (human) (Ccm2), mRNA | 0.587 |
| AK077383 | Mus musculus 6 days neonate head cDNA, RIKEN full-length enriched library, clone:5430409E09 product:unknown EST, full insert sequence | 0.587 |
| NM\_177266 | Mus musculus RIKEN cDNA A930009M04 gene (A930009M04Rik), mRNA | 0.587 |
| NM\_199322 | Mus musculus DOT1-like, histone H3 methyltransferase (S. cerevisiae) (Dot1l), mRNA | 0.587 |
| AK006869 | Mus musculus adult male testis cDNA, RIKEN full-length enriched library, clone:1700063H04 product:hypothetical protein, full insert sequence. | 0.587 |
| NAP029656-1 | Unknown | 0.587 |
| NM\_026041 | Mus musculus RIKEN cDNA 2810430M08 gene (2810430M08Rik), mRNA | 0.587 |
| AF273673 | Mus musculus Importin9 isoform 2 (Importin9) mRNA, complete cds. | 0.586 |
| NM\_019761 | Mus musculus NTF2-related export protein 1 (Nxt1), mRNA | 0.586 |
| NM\_031179 | Mus musculus splicing factor 3b, subunit 1 (Sf3b1), mRNA | 0.586 |
| AK010002 | Mus musculus adult male tongue cDNA, RIKEN full-length enriched library, clone:2310061I09 product:hypothetical protein, full insert sequence. | 0.586 |
| AK082752 | Mus musculus ES cells cDNA, RIKEN full-length enriched library, clone:C330003F08 product:similar to HYPOTHETICAL 35.1 KDA PROTEIN [Homo sapiens], full insert sequence. | 0.586 |
| NM\_011585 | Mus musculus cytotoxic granule-associated RNA binding protein 1 (Tia1), mRNA | 0.586 |
| NM\_026541 | Mus musculus RIKEN cDNA 4930553M18 gene (4930553M18Rik), mRNA | 0.586 |
| AK172938 | Unknown | 0.586 |
| NM\_019830 | Mus musculus heterogeneous nuclear ribonucleoproteins methyltransferase-like 2 (S. cerevisiae) (Hrmt1l2), mRNA | 0.586 |
| AK032668 | Mus musculus 10 days neonate cerebellum cDNA, RIKEN full-length enriched library, clone:6530410M19 product:unknown EST, full insert sequence. | 0.586 |
| NM\_138657 | Mus musculus suppressor of cytokine signaling 7 (Socs7), mRNA | 0.586 |
| NM\_025541 | Mus musculus ASF1 anti-silencing function 1 homolog A (S. cerevisiae) (Asf1a), mRNA | 0.585 |
| NM\_008740 | Mus musculus N-ethylmaleimide sensitive fusion protein (Nsf), mRNA | 0.585 |
| NM\_016714 | Mus musculus nucleoporin 50 (Nup50), mRNA | 0.585 |
| AB093302 | Mus musculus mRNA for mKIAA1842 protein. | 0.585 |
| NAP096050-001 | Unknown | 0.585 |
| NM\_011072 | Mus musculus profilin 1 (Pfn1), mRNA | 0.584 |
| NM\_146116 | Mus musculus RIKEN cDNA 4930542G03 gene (4930542G03Rik), mRNA | 0.584 |
| NM\_138677 | Mus musculus ER degradation enhancer, mannosidase alpha-like 1 (Edem1), mRNA | 0.584 |
| NM\_009423 | Mus musculus Tnf receptor associated factor 4 (Traf4), mRNA | 0.584 |
| AB093258 | Mus musculus mRNA for mKIAA0678 protein. | 0.584 |
| AK008437 | Mus musculus adult male small intestine cDNA, RIKEN full-length enriched library, clone:2010204K13 product:CIP7 (cip7) mRNA, full insert sequence. | 0.584 |
| NM\_146036 | Mus musculus AHA1, activator of heat shock 90kDa protein ATPase homolog 1 (yeast) (Ahsa1), mRNA | 0.584 |
| U27838 | Mus musculus glycosyl-phosphatidyl-inositol-anchored protein homolog mRNA, complete cds. | 0.584 |
| NM\_011014 | Mus musculus opioid receptor, sigma 1 (Oprs1), mRNA | 0.584 |
| NM\_028151 | Mus musculus RIKEN cDNA 2610528A15 gene (2610528A15Rik), mRNA | 0.583 |
| NM\_028023 | Mus musculus cell division cycle associated 4 (Cdca4), mRNA | 0.583 |
| NM\_133934 | Mus musculus RIKEN cDNA 5830446M03 gene (5830446M03Rik), mRNA | 0.583 |
| NAP021083-001 | Unknown | 0.583 |
| NAP111074-1 | Unknown | 0.583 |
| AK122471 | Mus musculus mRNA for mKIAA1221 protein. | 0.583 |
| NM\_175552 | Mus musculus WD repeat domain 3 (Wdr3), mRNA | 0.583 |
| NM\_009831 | Mus musculus cyclin G1 (Ccng1), mRNA | 0.582 |
| NM\_146141 | Mus musculus RIKEN cDNA 1110013G13 gene (1110013G13Rik), mRNA | 0.582 |
| NM\_030685 | Mus musculus DNA segment, Chr 3, University of California at Los Angeles 1 (D3Ucla1), mRNA | 0.582 |
| NM\_144945 | Mus musculus leucine-rich repeat LGI family, member 2 (Lgi2), mRNA | 0.582 |
| NM\_027148 | Mus musculus exosome component 8 (Exosc8), mRNA | 0.582 |
| NM\_144545 | Mus musculus eukaryotic translation initiation factor 3, subunit 1 alpha (Eif3s1), mRNA | 0.582 |
| NM\_181542 | Mus musculus schlafen 10 (Slfn10), mRNA | 0.582 |
| NM\_133834 | Mus musculus RIKEN cDNA 4833420I20 gene (4833420I20Rik), mRNA | 0.581 |
| NM\_026048 | Mus musculus RIKEN cDNA 2810452K22 gene (2810452K22Rik), mRNA | 0.581 |
| NM\_053124 | Mus musculus SWI/SNF related, matrix associated, actin dependent regulator of chromatin, subfamily a, member 5 (Smarca5), mRNA | 0.581 |
| AK010745 | Mus musculus ES cells cDNA, RIKEN full-length enriched library, clone:2410089B13 product:hypothetical protein, full insert sequence. | 0.581 |
| NAP070973-1 | Unknown | 0.581 |
| AK049441 | Mus musculus 7 days embryo whole body cDNA, RIKEN full-length enriched library, clone:C430011O11 product:succinate dehydrogenase complex, subunit A, flavoprotein (Fp), full insert sequence. | 0.581 |
| BM936630 | UI-M-BH3-arq-a-02-0-UI.r1 NIH\_BMAP\_M\_S4 Mus musculus cDNA clone UI-M-BH3-arq-a-02-0-UI 5', mRNA sequence | 0.580 |
| AK129287 | Mus musculus mRNA for mKIAA1090 protein. | 0.580 |
| NM\_025875 | Mus musculus RNA binding motif protein 8 (Rbm8), mRNA | 0.580 |
| XM\_358238 | Mus musculus similar to High mobility group protein 1 (HMG-1) (Amphoterin) (Heparin-binding protein p30) (LOC385454), mRNA | 0.580 |
| Z49204 | M.musculus mRNA for NADP transhydrogenase. | 0.580 |
| BC052434 | Mus musculus cell division cycle 6 homolog (S. cerevisiae), mRNA (cDNA clone MGC:63392 IMAGE:6837113), complete cds. | 0.580 |
| XM\_284500 | Mus musculus RIKEN cDNA 9930116O05 gene (9930116O05Rik), mRNA | 0.580 |
| A\_52\_P190357 | Unknown | 0.579 |
| BC053524 | Mus musculus importin 7, mRNA (cDNA clone IMAGE:5342173), partial cds. | 0.579 |
| NM\_010368 | Mus musculus beta-glucuronidase (Gus), mRNA | 0.579 |
| AK082094 | Mus musculus 0 day neonate cerebellum cDNA, RIKEN full-length enriched library, clone:C230006B22 product:HYPOTHETICAL 39.4 KDA PROTEIN homolog [Homo sapiens], full insert sequence. | 0.579 |
| NM\_025656 | Mus musculus survivor of motor neuron protein interacting protein 1 (Sip1), mRNA | 0.579 |
| NM\_023536 | Mus musculus RIKEN cDNA 2610012O22 gene (2610012O22Rik), mRNA | 0.579 |
| AK129110 | Mus musculus mRNA for mKIAA0286 protein. | 0.578 |
| NM\_025302 | Mus musculus mitochondrial ribosomal protein L2 (Mrpl2), mRNA | 0.578 |
| NM\_020570 | Mus musculus X-ray repair complementing defective repair in Chinese hamster cells 2 (Xrcc2), mRNA | 0.578 |
| AK051733 | Mus musculus 12 days embryo spinal ganglion cDNA, RIKEN full-length enriched library, clone:D130068H22 product:Erbb2 interacting protein, full insert sequence. | 0.578 |
| BC037217 | Mus musculus RIKEN cDNA A230103N10 gene, mRNA (cDNA clone IMAGE:5355497), with apparent retained intron | 0.577 |
| NM\_025436 | Mus musculus sterol-C4-methyl oxidase-like (Sc4mol), mRNA | 0.577 |
| NM\_011640 | Mus musculus transformation related protein 53 (Trp53), mRNA | 0.577 |
| NM\_009863 | Mus musculus cell division cycle 7 (S. cerevisiae) (Cdc7), mRNA | 0.576 |
| NM\_007573 | Mus musculus complement component 1, q subcomponent binding protein (C1qbp), mRNA | 0.576 |
| NM\_027349 | Mus musculus RIKEN cDNA 2610015J01 gene (2610015J01Rik), mRNA | 0.576 |
| NM\_172967 | Mus musculus RIKEN cDNA 4930503L19 gene (4930503L19Rik), mRNA | 0.576 |
| NM\_024240 | Mus musculus RIKEN cDNA 2810037C03 gene (2810037C03Rik), mRNA | 0.576 |
| NM\_011156 | Mus musculus prolyl endopeptidase (Prep), mRNA | 0.575 |
| NM\_030241 | Mus musculus RIKEN cDNA 2410195B05 gene (2410195B05Rik), mRNA | 0.575 |
| NM\_026640 | Mus musculus expressed sequence AW413625 (AW413625), mRNA | 0.575 |
| ENSMUST00000059950 | AGENCOURT\_10722970 NIH\_MGC\_169 Mus musculus cDNA clone IMAGE:6771789 5', mRNA sequence [CA464118] | 0.574 |
| NM\_009839 | Mus musculus chaperonin subunit 6b (zeta) (Cct6b), mRNA | 0.574 |
| AK045060 | Mus musculus 9.5 days embryo parthenogenote cDNA, RIKEN full-length enriched library, clone:B130023L16 product:unknown EST, full insert sequence. | 0.574 |
| ENSMUST00000058706 | Unknown | 0.574 |
| AK042338 | Mus musculus 3 days neonate thymus cDNA, RIKEN full-length enriched library, clone:A630083K06 product:unclassifiable, full insert sequence. | 0.573 |
| NM\_010404 | Mus musculus huntingtin-associated protein 1 (Hap1), transcript variant 1, mRNA | 0.573 |
| AK079278 | Mus musculus adult male urinary bladder cDNA, RIKEN full-length enriched library, clone:9530080G04 product:unknown EST, full insert sequence. | 0.573 |
| AK051624 | Mus musculus 12 days embryo spinal ganglion cDNA, RIKEN full-length enriched library, clone:D130060M14 product:protein kinase, cGMP-dependent, type I, full insert sequence. | 0.573 |
| AK012399 | Mus musculus 11 days embryo whole body cDNA, RIKEN full-length enriched library, clone:2700049A03 product:hypothetical protein, full insert sequence. | 0.573 |
| AK010523 | Mus musculus ES cells cDNA, RIKEN full-length enriched library, clone:2410017C19 product:INOSITOL POLYPHOSPHATE MULTIKINASE homolog [Rattus norvegicus], full insert sequence. | 0.573 |
| NM\_025638 | Mus musculus RIKEN cDNA 2610020H15 gene (2610020H15Rik), mRNA | 0.573 |
| XM\_136018 | Mus musculus RIKEN cDNA 4933401B06 gene (4933401B06Rik), mRNA | 0.573 |
| NM\_177246 | Mus musculus RIKEN cDNA B230220B15 gene (B230220B15Rik), mRNA | 0.573 |
| AK011507 | Mus musculus 10 days embryo whole body cDNA, RIKEN full-length enriched library, clone:2610021K21 product:hypothetical EF-hand/Aminoacyl-transfer RNA synthetases class-II containing protein, full insert sequence. | 0.573 |
| AK012978 | Mus musculus 10, 11 days embryo whole body cDNA, RIKEN full-length enriched library, clone:2810403P18 product:similar to CDNA FLJ10539 FIS, CLONE NT2RP2001218 [Homo sapiens], full insert sequence | 0.572 |
| NM\_027869 | Mus musculus polyribonucleotide nucleotidyltransferase 1 (Pnpt1), mRNA | 0.572 |
| XM\_144609 | Mus musculus similar to High mobility group protein 1 (HMG-1) (LOC243311), mRNA | 0.572 |
| NM\_027328 | Mus musculus RIKEN cDNA 1500019O16 gene (1500019O16Rik), mRNA | 0.572 |
| NM\_172647 | Mus musculus F11 receptor (F11r), mRNA | 0.572 |
| AK012141 | Mus musculus 10 days embryo whole body cDNA, RIKEN full-length enriched library, clone:2610524F24 product:hypothetical protein, full insert sequence. | 0.572 |
| XM\_356350 | Mus musculus similar to WD repeat domain 9 isoform A; cAMP response element binding and beta-tranducin family-like; transcriptional unit N143 (LOC382236), mRNA | 0.572 |
| NM\_011410 | Mus musculus schlafen 4 (Slfn4), mRNA | 0.571 |
| NM\_023773 | Mus musculus RIKEN cDNA 4930548G07 gene (4930548G07Rik), mRNA | 0.571 |
| NM\_030215 | Mus musculus Werner helicasae interacting protein 1 (Wrnip1), mRNA | 0.571 |
| AK129215 | Mus musculus mRNA for mKIAA0786 protein. | 0.571 |
| NM\_134011 | Mus musculus transforming growth factor beta regulated gene 4 (Tbrg4), mRNA | 0.570 |
| TC1059737 | AF490391 sperm-associated WD repeat protein {Mus musculus}, partial (3%) | 0.570 |
| BC018281 | Mus musculus RIKEN cDNA 6030411K04 gene, mRNA (cDNA clone MGC:7843 IMAGE:3500852), complete cds. | 0.570 |
| NM\_175332 | Mus musculus RIKEN cDNA E130012A19 gene (E130012A19Rik), mRNA | 0.570 |
| XM\_207781 | Mus musculus similar to RNA polymerase II termination factor (LOC280458), mRNA | 0.570 |
| NM\_016681 | Mus musculus CHK2 checkpoint homolog (S. pombe) (Chek2), mRNA | 0.570 |
| BC030947 | Mus musculus succinate-Coenzyme A ligase, GDP-forming, beta subunit, mRNA (cDNA clone IMAGE:5003997), partial cds. | 0.569 |
| NM\_145627 | Mus musculus RNA binding motif protein 10 (Rbm10), mRNA | 0.569 |
| NM\_025531 | Mus musculus RIKEN cDNA 2310042G06 gene (2310042G06Rik), mRNA | 0.569 |
| NM\_018886 | Mus musculus lectin, galactose binding, soluble 8 (Lgals8), mRNA | 0.569 |
| NM\_134040 | Mus musculus DEAD (Asp-Glu-Ala-Asp) box polypeptide 1 (Ddx1), mRNA | 0.569 |
| NM\_010123 | Mus musculus eukaryotic translation initiation factor 3, subunit 10 (theta) (Eif3s10), mRNA | 0.568 |
| NM\_008021 | Mus musculus forkhead box M1 (Foxm1), mRNA | 0.568 |
| NM\_172527 | Mus musculus nudix (nucleoside diphosphate linked moiety X)-type motif 15 (Nudt15), mRNA | 0.568 |
| NM\_011951 | Mus musculus mitogen activated protein kinase 14 (Mapk14), mRNA | 0.568 |
| XM\_134917 | Mus musculus similar to senescence downregulated leo1-like (LOC235497), mRNA | 0.568 |
| NM\_011677 | Mus musculus uracil-DNA glycosylase (Ung), mRNA | 0.568 |
| AK080282 | Mus musculus 3 days neonate thymus cDNA, RIKEN full-length enriched library, clone:A630024B09 product:DNA segment, Chr 10, ERATO Doi 755, expressed, full insert sequence | 0.568 |
| AK032585 | Mus musculus adult male olfactory brain cDNA, RIKEN full-length enriched library, clone:6430604M11 product:unknown EST, full insert sequence | 0.568 |
| NM\_022654 | Mus musculus leucine-rich and death domain containing (Lrdd), mRNA | 0.567 |
| A\_51\_P383950 | Unknown | 0.567 |
| AK010662 | Mus musculus ES cells cDNA, RIKEN full-length enriched library, clone:2410043H24 product:tRNA adenylyltransferase, mitochondrial, full insert sequence. | 0.567 |
| NM\_031161 | Mus musculus cholecystokinin (Cck), mRNA | 0.567 |
| BC058577 | Mus musculus cDNA clone IMAGE:6489870, partial cds | 0.567 |
| NM\_018825 | Mus musculus adaptor protein with pleckstrin homology and src (Aps), mRNA | 0.567 |
| NM\_007669 | Mus musculus cyclin-dependent kinase inhibitor 1A (P21) (Cdkn1a), mRNA | 0.567 |
| NM\_019816 | Mus musculus apoptosis antagonizing transcription factor (Aatf), mRNA | 0.567 |
| NM\_013595 | Mus musculus methyl-CpG binding domain protein 3 (Mbd3), mRNA | 0.567 |
| NM\_022018 | Mus musculus niban protein (Niban), mRNA | 0.566 |
| NM\_134024 | Mus musculus tubulin, gamma 1 (Tubg1), mRNA | 0.566 |
| BC002288 | Mus musculus, clone IMAGE:3590001, mRNA, partial cds. | 0.566 |
| NM\_007624 | Mus musculus chromobox homolog 3 (Drosophila HP1 gamma) (Cbx3), mRNA | 0.566 |
| NAP112302-1 | Unknown | 0.566 |
| NM\_017377 | Mus musculus UDP-Gal:betaGlcNAc beta 1,4- galactosyltransferase, polypeptide 2 (B4galt2), mRNA | 0.566 |
| NM\_013832 | Mus musculus RAS protein activator like 1 (GAP1 like) (Rasal1), mRNA | 0.566 |
| NM\_024177 | Mus musculus mitochondrial ribosomal protein L38 (Mrpl38), mRNA | 0.566 |
| NM\_026453 | Mus musculus RIKEN cDNA 2600016B03 gene (2600016B03Rik), mRNA | 0.565 |
| NM\_025300 | Mus musculus mitochondrial ribosomal protein L15 (Mrpl15), mRNA | 0.565 |
| NM\_011568 | Mus musculus THO complex 4 (Thoc4), mRNA | 0.564 |
| NM\_029344 | Mus musculus acylphosphatase 2, muscle type (Acyp2), mRNA | 0.564 |
| BC066048 | Mus musculus peroxisome proliferative activated receptor, gamma, coactivator-related 1, mRNA (cDNA clone MGC:90133 IMAGE:6825158), complete cds. | 0.564 |
| AK122269 | Mus musculus mRNA for mKIAA0386 protein. | 0.564 |
| NM\_009196 | Mus musculus solute carrier family 16 (monocarboxylic acid transporters), member 1 (Slc16a1), mRNA | 0.564 |
| BC052756 | Mus musculus transportin 3, mRNA (cDNA clone IMAGE:6334654), partial cds. | 0.564 |
| NM\_025878 | Mus musculus mitochondrial ribosomal protein S18B (Mrps18b), mRNA | 0.564 |
| NM\_011200 | Mus musculus protein tyrosine phosphatase 4a1 (Ptp4a1), mRNA | 0.563 |
| NAP028542-1 | Unknown | 0.563 |
| BF160261 | 601771184F1 NCI\_CGAP\_Lu29 Mus musculus cDNA clone IMAGE:3990212 5'. | 0.563 |
| NM\_172406 | Mus musculus amyotrophic lateral sclerosis 2 (juvenile) chromosome region, candidate 3 homolog(human) (Als2cr3), mRNA | 0.563 |
| AK085987 | Mus musculus 16 days neonate heart cDNA, RIKEN full-length enriched library, clone:D830038K18 product:hypothetical Vitamin B12 dependent methionine synthase activation domain containing protein, full insert sequence. | 0.563 |
| AK078821 | Mus musculus 16 days embryo lung cDNA, RIKEN full-length enriched library, clone:8430430N13 product:hypothetical protein, full insert sequence. | 0.563 |
| NM\_176976 | Mus musculus RIKEN cDNA 5830418K08 gene (5830418K08Rik), mRNA | 0.563 |
| NM\_001001882 | Mus musculus expressed sequence AW540478 (AW540478), mRNA | 0.563 |
| AK079255 | Mus musculus adult male urinary bladder cDNA, RIKEN full-length enriched library, clone:9530059O14 product:unknown EST, full insert sequence. | 0.562 |
| BC063078 | Mus musculus RIKEN cDNA 3110040D16 gene, mRNA (cDNA clone IMAGE:6827516), partial cds | 0.562 |
| NM\_173189 | Mus musculus RIKEN cDNA D030046N04 gene (D030046N04Rik), mRNA | 0.562 |
| AK010827 | Mus musculus ES cells cDNA, RIKEN full-length enriched library, clone:2410170E07 product:hypothetical P-loop containing nucleotide triphosphate hydrolases structure containing protein, full insert sequence. | 0.562 |
| NM\_027332 | Mus musculus RIKEN cDNA 2310066N05 gene (2310066N05Rik), mRNA | 0.562 |
| NM\_025902 | Mus musculus RIKEN cDNA 1500009M05 gene (1500009M05Rik), mRNA | 0.562 |
| NM\_053179 | Mus musculus N-acetylneuraminic acid synthase (sialic acid synthase) (Nans), mRNA | 0.562 |
| NM\_134092 | Mus musculus Mdm2, transformed 3T3 cell double minute p53 binding protein (Mtbp), mRNA | 0.562 |
| AY036116 | Mus musculus membrane-bound factor MBF1 (Mbf) mRNA, complete cds. | 0.562 |
| NM\_146043 | Mus musculus spindlin (Spin), mRNA | 0.561 |
| NM\_019869 | Mus musculus RNA binding motif protein 14 (Rbm14), mRNA | 0.561 |
| NM\_009774 | Mus musculus budding uninhibited by benzimidazoles 3 homolog (S. cerevisiae) (Bub3), mRNA | 0.561 |
| NM\_152807 | Mus musculus RIKEN cDNA 3110023B02 gene (3110023B02Rik), mRNA | 0.561 |
| AK078008 | Mus musculus 11 days embryo head cDNA, RIKEN full-length enriched library, clone:6230408M08 product:unknown EST, full insert sequence | 0.560 |
| NM\_153082 | Mus musculus RIKEN cDNA C330021A05 gene (C330021A05Rik), mRNA | 0.560 |
| NM\_134066 | Mus musculus aldo-keto reductase family 1, member C18 (Akr1c18), mRNA | 0.560 |
| D10727 | Mus musculus mRNA for NDPP-1 protein, complete cds. | 0.560 |
| NM\_033526 | Mus musculus expressed sequence AI663987 (AI663987), mRNA | 0.560 |
| NM\_182939 | Mus musculus protein phosphatase 4, regulatory subunit 2 (Ppp4r2), mRNA | 0.560 |
| AK085944 | Mus musculus 16 days neonate heart cDNA, RIKEN full-length enriched library, clone:D830032E20 product:similar to ENVELOPE PROTEIN (FRAGMENT) [Friend spleen focus-forming virus], full insert sequence. | 0.559 |
| NM\_198246 | Mus musculus RIKEN cDNA 2210023C10 gene (2210023C10Rik), mRNA | 0.559 |
| AK088462 | Mus musculus 2 days neonate thymus thymic cells cDNA, RIKEN full-length enriched library, clone:E430018E05 product:heterogeneous nuclear ribonucleoprotein K, full insert sequence. | 0.559 |
| NM\_009383 | Mus musculus Tial1 cytotoxic granule-associated RNA binding protein-like 1 (Tial1), mRNA | 0.559 |
| XM\_141694 | Mus musculus similar to transcription factor NRF; ITBA4 gene (9430034D17Rik), mRNA | 0.559 |
| NM\_178054 | Mus musculus muted (Muted), mRNA | 0.559 |
| TC1025241 | U96416 cytochrome b {Dennyus distinctus timjonesi}, partial (11%) | 0.558 |
| NM\_144829 | Mus musculus RIKEN cDNA 2310044P18 gene (2310044P18Rik), mRNA | 0.558 |
| NM\_001001152 | Mus musculus zinc finger protein 458 (Zfp458), mRNA | 0.558 |
| NM\_027432 | Mus musculus RIKEN cDNA 2610312E17 gene (2610312E17Rik), mRNA | 0.558 |
| NM\_021897 | Mus musculus transformation related protein 53 inducible nuclear protein 1 (Trp53inp1), mRNA | 0.558 |
| NM\_031376 | Mus musculus phosphoinositide-3-kinase adaptor protein 1 (Pik3ap1), mRNA | 0.558 |
| NM\_007630 | Mus musculus cyclin B2 (Ccnb2), mRNA | 0.558 |
| NM\_028358 | Mus musculus single-stranded DNA binding protein 1 (Ssbp1), transcript variant 2, mRNA | 0.558 |
| BC054123 | Mus musculus kinesin family member 14, mRNA (cDNA clone IMAGE:6509974), partial cds. | 0.558 |
| AK089259 | Mus musculus NOD-derived CD11c +ve dendritic cells cDNA, RIKEN full-length enriched library, clone:F630107H02 product:sideroflexin 2, full insert sequence | 0.557 |
| NM\_010237 | Mus musculus fyn-related kinase (Frk), mRNA | 0.557 |
| NM\_145150 | Mus musculus protein regulator of cytokinesis 1 (Prc1), mRNA | 0.557 |
| BC028900 | Mus musculus pleckstrin homology domain containing, family H (with MyTH4 domain) member 1, mRNA (cDNA clone IMAGE:3495084), partial cds. | 0.557 |
| NM\_134071 | Mus musculus ankyrin repeat domain 32 (Ankrd32), mRNA | 0.557 |
| NM\_011982 | Mus musculus homer homolog 1 (Drosophila) (Homer1), transcript variant S, mRNA | 0.557 |
| TC1002006 | BC026797 Gle1l protein {Mus musculus}, complete | 0.557 |
| AK082484 | Mus musculus 0 day neonate cerebellum cDNA, RIKEN full-length enriched library, clone:C230055J01 product:PHOSPHORIBOSYLPYROPHOSPHATE SYNTHETASE-ASSOCIATED PROTEIN (39 KDA) (PHOSPHORIBOSYLPYROPHOSPHATE SYNTHETASE-ASSOCIATED PROTEIN 39) homolog [Rattus no | 0.556 |
| AK032738 | Mus musculus 12 days embryo male wolffian duct includes surrounding region cDNA, RIKEN full-length enriched library, clone:6720422M22 product:unknown EST, full insert sequence | 0.556 |
| NM\_025930 | Mus musculus RIKEN cDNA 2600011C06 gene (2600011C06Rik), mRNA | 0.556 |
| NM\_028279 | Mus musculus N-acetylated alpha-linked acidic dipeptidase 2 (Naalad2), mRNA | 0.556 |
| NM\_026472 | Mus musculus Mki67 (FHA domain) interacting nucleolar phosphoprotein (Mki67ip), mRNA | 0.556 |
| NM\_138757 | Mus musculus RIKEN cDNA 4933424B01 gene (4933424B01Rik), mRNA | 0.556 |
| NM\_009359 | Mus musculus testis expressed gene 9 (Tex9), mRNA | 0.556 |
| NM\_024438 | Mus musculus dual specificity phosphatase 19 (Dusp19), mRNA | 0.555 |
| NM\_007951 | Mus musculus enhancer of rudimentary homolog (Drosophila) (Erh), mRNA | 0.555 |
| AK084405 | Mus musculus 12 days embryo eyeball cDNA, RIKEN full-length enriched library, clone:D230038P13 product:unclassifiable, full insert sequence. | 0.555 |
| NM\_019402 | Mus musculus poly(A) binding protein, nuclear 1 (Pabpn1), mRNA | 0.555 |
| AK028031 | Mus musculus 18-day embryo whole body cDNA, RIKEN full-length enriched library, clone:1190025P04 product:similar to DC11 [Homo sapiens], full insert sequence. | 0.555 |
| NM\_026921 | Mus musculus HESB like domain containing 2 (Hbld2), mRNA | 0.555 |
| NM\_207218 | Mus musculus expressed sequence AI461933 (AI461933), mRNA | 0.555 |
| NM\_016856 | Mus musculus cleavage and polyadenylation specific factor 2 (Cpsf2), mRNA | 0.555 |
| AI465905 | vw18e05.y1 Soares\_mammary\_gland\_NbMMG Mus musculus cDNA clone IMAGE:1244192 5' similar to gb:X52009 GLYCINE RECEPTOR ALPHA-1 CHAIN PRECURSOR (HUMAN);. | 0.555 |
| NM\_025525 | Mus musculus RIKEN cDNA 2310020H19 gene (2310020H19Rik), mRNA | 0.554 |
| AK046853 | Mus musculus 10 days neonate medulla oblongata cDNA, RIKEN full-length enriched library, clone:B830029I03 product:hypothetical G-protein beta WD-40 repeats containing protein, full insert sequence. | 0.554 |
| AK052318 | Mus musculus 13 days embryo heart cDNA, RIKEN full-length enriched library, clone:D330027G24 product:unclassifiable, full insert sequence | 0.554 |
| NM\_010178 | Mus musculus FUS interacting protein (serine-arginine rich) 1 (Fusip1), mRNA | 0.554 |
| NM\_011171 | Mus musculus protein C receptor, endothelial (Procr), mRNA | 0.553 |
| AK086180 | Mus musculus 15 days embryo head cDNA, RIKEN full-length enriched library, clone:D930010O07 product:hypothetical protein, full insert sequence. | 0.553 |
| NM\_026149 | Mus musculus RIKEN cDNA 4921532K09 gene (4921532K09Rik), mRNA | 0.553 |
| NM\_030143 | Mus musculus DNA-damage-inducible transcript 4-like (Ddit4l), mRNA | 0.553 |
| XM\_354775 | Mus musculus similar to NADH-ubiquinone oxidoreductase 13 kDa-A subunit, mitochondrial precursor (Complex I-13KD-A) (CI-13KD-A) (LOC380871), mRNA | 0.552 |
| AK087204 | Mus musculus 0 day neonate lung cDNA, RIKEN full-length enriched library, clone:E030034B13 product:unclassifiable, full insert sequence. | 0.552 |
| NM\_018859 | Mus musculus aldo-keto reductase family 1, member E1 (Akr1e1), mRNA | 0.552 |
| NM\_013822 | Mus musculus jagged 1 (Jag1), mRNA | 0.552 |
| NM\_016861 | Mus musculus PDZ and LIM domain 1 (elfin) (Pdlim1), mRNA | 0.552 |
| CF750939 | UI-M-HK0-cmr-e-21-0-UI.r1 NIH\_BMAP\_HK0 Mus musculus cDNA clone IMAGE:30623252 5', mRNA sequence | 0.552 |
| NM\_018771 | Mus musculus regulator of G-protein signaling 19 interacting protein 1 (Rgs19ip1), mRNA | 0.552 |
| AK030819 | Mus musculus adult male thymus cDNA, RIKEN full-length enriched library, clone:5830412K01 product:weakly similar to MUCOLIPIDIN [Homo sapiens], full insert sequence. | 0.552 |
| NM\_009689 | Mus musculus baculoviral IAP repeat-containing 5 (Birc5), mRNA | 0.552 |
| NM\_176933 | Mus musculus dual specificity phosphatase 4 (Dusp4), mRNA | 0.551 |
| NM\_025616 | Mus musculus translocase of inner mitochondrial membrane 50 homolog (yeast) (Timm50), mRNA | 0.551 |
| TC982705 | ERF1\_HUMAN Eukaryotic peptide chain release factor subunit 1 (eRF1) (Eukaryotic release factor 1) (TB3-1) (C11 protein). [Rabbit] {Oryctolagus cuniculus}, complete | 0.551 |
| AK083569 | Mus musculus 9 days embryo whole body cDNA, RIKEN full-length enriched library, clone:D030046J24 product:unknown EST, full insert sequence | 0.551 |
| NM\_011808 | Mus musculus E26 avian leukemia oncogene 1, 5' domain (Ets1), mRNA | 0.551 |
| NM\_023645 | Mus musculus KDEL (Lys-Asp-Glu-Leu) containing 1 (Kdelc1), mRNA | 0.551 |
| NM\_015747 | Mus musculus solute carrier family 20, member 1 (Slc20a1), mRNA | 0.551 |
| NM\_199199 | Mus musculus expressed sequence AI316787 (AI316787), mRNA | 0.550 |
| NM\_175016 | Mus musculus expressed sequence AU016977 (AU016977), mRNA | 0.550 |
| NM\_007891 | Mus musculus E2F transcription factor 1 (E2f1), mRNA | 0.550 |
| NM\_007525 | Mus musculus BRCA1 associated RING domain 1 (Bard1), mRNA | 0.550 |
| NM\_201364 | Mus musculus similar to hypothetical protein FLJ10706 (MGC65590), mRNA | 0.549 |
| NM\_022328 | Mus musculus myeloid/lymphoid or mixed lineage-leukemia translocation to 1 homolog (Drosophila) (Mllt1), mRNA | 0.549 |
| XM\_136985 | Mus musculus similar to triosephosphate isomerase 1 (LOC215969), mRNA | 0.549 |
| NM\_013559 | Mus musculus heat shock protein 105 (Hsp105), mRNA | 0.549 |
| NM\_175498 | Mus musculus paraneoplastic antigen MA2 (Pnma2), mRNA | 0.549 |
| NM\_028717 | Mus musculus amyotrophic lateral sclerosis 2 (juvenile) homolog (human) (Als2), mRNA | 0.549 |
| NM\_008697 | Mus musculus ninein (Nin), mRNA | 0.549 |
| NM\_023449 | Mus musculus solute carrier family 9 (sodium/hydrogen exchanger), isoform 3 regulator 2 (Slc9a3r2), mRNA | 0.548 |
| NM\_026004 | Mus musculus 5'-nucleotidase, cytosolic III (Nt5c3), mRNA | 0.548 |
| BC049236 | Mus musculus, clone IMAGE:4235872, mRNA. | 0.548 |
| AK013315 | Mus musculus 10, 11 days embryo whole body cDNA, RIKEN full-length enriched library, clone:2810449M14 product:inferred: RIKEN cDNA 5730434B08 gene, full insert sequence. | 0.548 |
| NM\_138599 | Mus musculus DNA segment, Chr 16, Wayne State University 109, expressed (D16Wsu109e), mRNA | 0.548 |
| NM\_026737 | Mus musculus PHD finger protein 5A (Phf5a), mRNA | 0.548 |
| NM\_026602 | Mus musculus breast carcinoma amplified sequence 2 (Bcas2), mRNA | 0.548 |
| NM\_010191 | Mus musculus farnesyl diphosphate farnesyl transferase 1 (Fdft1), mRNA | 0.547 |
| NM\_027930 | Mus musculus RIKEN cDNA 2610016C23 gene (2610016C23Rik), mRNA | 0.547 |
| NM\_145409 | Mus musculus CTF18, chromosome transmission fidelity factor 18 homolog (S. cerevisiae) (Chtf18), mRNA | 0.547 |
| NM\_007968 | Mus musculus Ewing sarcoma homolog (Ewsh), mRNA | 0.547 |
| NM\_029573 | Mus musculus isocitrate dehydrogenase 3 (NAD+) alpha (Idh3a), mRNA | 0.547 |
| BE981145 | UI-M-CG0-bcu-h-04-0-UI.s1 NIH\_BMAP\_Ret4\_S1 Mus musculus cDNA clone UI-M-CG0-bcu-h-04-0-UI 3'. | 0.547 |
| AK078146 | Mus musculus adult male medulla oblongata cDNA, RIKEN full-length enriched library, clone:6330576N08 product:hypothetical TPR repeat containing protein, full insert sequence. | 0.547 |
| AK034817 | Mus musculus 12 days embryo embryonic body between diaphragm region and neck cDNA, RIKEN full-length enriched library, clone:9430042G06 product:nucleolar and coiled-body phosphoprotein 1, full insert sequence | 0.546 |
| NM\_024174 | Mus musculus mitochondrial ribosomal protein S23 (Mrps23), mRNA | 0.546 |
| XM\_142470 | Mus musculus similar to variable region of immunoglobulin kappa light chain (LOC235908), mRNA | 0.546 |
| NM\_022309 | Mus musculus core binding factor beta (Cbfb), mRNA | 0.546 |
| NM\_172796 | Mus musculus RIKEN cDNA 9830137M10 gene (9830137M10Rik), mRNA | 0.546 |
| AK077686 | Mus musculus 8 days embryo whole body cDNA, RIKEN full-length enriched library, clone:5730521N16 product:weakly similar to ZINC FINGER PROTEIN 4 (FRAGMENT) [Rattus norvegicus], full insert sequence. | 0.546 |
| NM\_138306 | Mus musculus diacylglycerol kinase zeta (Dgkz), mRNA | 0.546 |
| NM\_145955 | Mus musculus RIKEN cDNA 1110007A13 gene (1110007A13Rik), mRNA | 0.545 |
| NM\_013554 | Mus musculus homeo box D10 (Hoxd10), mRNA | 0.545 |
| AF058956 | Mus musculus GTP-specific succinyl-CoA synthetase beta subunit (Scs) mRNA, partial cds. | 0.545 |
| NM\_010852 | Mus musculus myelin basic protein expression factor 2, repressor (Myef2), mRNA | 0.545 |
| NM\_025281 | Mus musculus Ly1 antibody reactive clone (Lyar), mRNA | 0.545 |
| NM\_025860 | Mus musculus DEAD (Asp-Glu-Ala-Asp) box polypeptide 18 (Ddx18), mRNA | 0.545 |
| AK019500 | Mus musculus 0 day neonate skin cDNA, RIKEN full-length enriched library, clone:4632417O19 product:NS1-associated protein 1, full insert sequence. | 0.545 |
| NM\_134160 | Mus musculus mucolipin 3 (Mcoln3), mRNA | 0.545 |
| NM\_009015 | Mus musculus RAD54 like (S. cerevisiae) (Rad54l), mRNA | 0.545 |
| NM\_133780 | Mus musculus translocated promoter region (Tpr), mRNA | 0.544 |
| AK052358 | Mus musculus 13 days embryo heart cDNA, RIKEN full-length enriched library, clone:D330037H05 product:unknown EST, full insert sequence. | 0.544 |
| NM\_009408 | Mus musculus topoisomerase (DNA) I (Top1), mRNA | 0.544 |
| NM\_172733 | Mus musculus RIKEN cDNA 2500002K03 gene (2500002K03Rik), mRNA | 0.544 |
| NM\_030066 | Mus musculus armadillo repeat containing, X-linked 1 (Armcx1), mRNA | 0.544 |
| NM\_177014 | Mus musculus RIKEN cDNA A530020G20 gene (A530020G20Rik), mRNA | 0.544 |
| NM\_025310 | Mus musculus FtsJ homolog 3 (E. coli) (Ftsj3), mRNA | 0.544 |
| BC054110 | Mus musculus cDNA clone MGC:60992 IMAGE:30017181, complete cds. | 0.544 |
| A\_51\_P402908 | Unknown | 0.543 |
| NM\_008193 | Mus musculus guanylate kinase 1 (Guk1), mRNA | 0.543 |
| NAP026555-1 | Unknown | 0.543 |
| BC023730 | Mus musculus expressed sequence AI452358, mRNA (cDNA clone IMAGE:5352301), partial cds | 0.543 |
| NM\_018757 | Mus musculus expressed in non-metastatic cells 6, protein (Nme6), mRNA | 0.543 |
| AK029572 | Mus musculus adult male testis cDNA, RIKEN full-length enriched library, clone:4930402C13 product:hypothetical SCAN domain containing protein, full insert sequence. | 0.543 |
| AK038129 | Mus musculus 16 days neonate thymus cDNA, RIKEN full-length enriched library, clone:A130080M17 product:inner membrane protein, mitochondrial, full insert sequence. | 0.543 |
| BC018353 | Mus musculus heterogeneous nuclear ribonucleoprotein U, mRNA (cDNA clone MGC:5768 IMAGE:3584692), complete cds. | 0.543 |
| XM\_143595 | Mus musculus similar to Nucleoside diphosphate kinase B (NDK B) (NDP kinase B) (P18) (LOC229879), mRNA | 0.543 |
| NM\_009860 | Mus musculus cell division cycle 25 homolog C (S. cerevisiae) (Cdc25c), mRNA | 0.542 |
| BC057604 | Mus musculus cDNA clone MGC:67261 IMAGE:6416213, complete cds. | 0.542 |
| NM\_008302 | Mus musculus heat shock protein 1, beta (Hspcb), mRNA | 0.541 |
| AK044509 | Mus musculus adult retina cDNA, RIKEN full-length enriched library, clone:A930017M02 product:unknown EST, full insert sequence | 0.541 |
| AF114437 | Mus musculus phosphatidylcholine transfer protein (Pctp) mRNA, partial cds. | 0.541 |
| NM\_181545 | Mus musculus schlafen 8 (Slfn8-pending), mRNA | 0.541 |
| NAP059333-1 | Unknown | 0.541 |
| NM\_145705 | Mus musculus Terf1 (TRF1)-interacting nuclear factor 2 (Tinf2), mRNA | 0.541 |
| NM\_133774 | Mus musculus StAR-related lipid transfer (START) domain containing 4 (Stard4), mRNA | 0.541 |
| NM\_181590 | Mus musculus RIKEN cDNA 2810403P18 gene (2810403P18Rik), mRNA | 0.541 |
| NM\_145956 | Mus musculus c6.1a protein (C6.1A), mRNA | 0.541 |
| NM\_025644 | Mus musculus exosome component 1 (Exosc1), mRNA | 0.541 |
| BC032932 | Mus musculus RIKEN cDNA 2610101J03 gene, mRNA (cDNA clone MGC:41415 IMAGE:1529010), complete cds. | 0.540 |
| NM\_139063 | Mus musculus muted (Muted), mRNA | 0.540 |
| AF024519 | Mus musculus glucocorticoid-induced leucine zipper GILZ protein mRNA, complete cds. | 0.540 |
| NM\_133933 | Mus musculus ribophorin I (Rpn1), mRNA | 0.540 |
| NM\_178391 | Mus musculus SET domain and mariner transposase fusion gene (Setmar), mRNA | 0.540 |
| AK051339 | Mus musculus 12 days embryo spinal ganglion cDNA, RIKEN full-length enriched library, clone:D130036J04 product:EPSILON-TRIMETHYLLYSINE 2-OXOGLUTARATE DIOXYGENASE TMLH (EC 1.14.11.8) homolog [Mus musculus], full insert sequence. | 0.540 |
| AK046429 | Mus musculus adult male corpora quadrigemina cDNA, RIKEN full-length enriched library, clone:B230384C22 product:hypothetical P-loop containing nucleotide triphosphate hydrolases structure containing protein, full insert sequence. | 0.539 |
| NM\_175628 | Mus musculus alpha-2-macroglobulin (A2m), mRNA | 0.539 |
| BC023187 | Mus musculus cell division cycle 27 homolog (S. cerevisiae), mRNA (cDNA clone MGC:36343 IMAGE:4954387), complete cds. | 0.539 |
| NM\_026623 | Mus musculus cleavage and polyadenylation specific factor 5 (Cpsf5), mRNA | 0.539 |
| NM\_009013 | Mus musculus RAD51 associated protein 1 (Rad51ap1), mRNA | 0.539 |
| NM\_025840 | Mus musculus basic leucine zipper and W2 domains 2 (Bzw2), mRNA | 0.539 |
| AK086572 | Mus musculus 15 days embryo head cDNA, RIKEN full-length enriched library, clone:D930037P12 product:214K23.2.1 (NOVEL PROTEIN, ISOFORM 1) (FRAGMENT) homolog [Homo sapiens], full insert sequence. | 0.539 |
| NM\_026728 | Mus musculus DNA segment, Chr 4, ERATO Doi 765, expressed (D4Ertd765e), mRNA | 0.539 |
| NM\_023556 | Mus musculus mevalonate kinase (Mvk), mRNA | 0.539 |
| NM\_016806 | Mus musculus heterogeneous nuclear ribonucleoprotein A2/B1 (Hnrpa2b1), transcript variant 1, mRNA | 0.538 |
| NM\_028109 | Mus musculus RIKEN cDNA 2610005B21 gene (2610005B21Rik), mRNA | 0.537 |
| TC1010843 | ROA1\_HUMAN Heterogeneous nuclear ribonucleoprotein A1 (Helix-destabilizing protein) (Single-strand binding protein) (hnRNP core protein A1). [Human] {Homo sapiens}, partial (98%) | 0.537 |
| NM\_016865 | Mus musculus HIV-1 tat interactive protein 2, homolog (human) (Htatip2), mRNA | 0.537 |
| NM\_011655 | Mus musculus tubulin, beta 5 (Tubb5), mRNA | 0.537 |
| AK038694 | Mus musculus adult male hypothalamus cDNA, RIKEN full-length enriched library, clone:A230055N17 product:unknown EST, full insert sequence | 0.536 |
| AK007939 | Mus musculus 10 day old male pancreas cDNA, RIKEN full-length enriched library, clone:1810063B05 product:unknown EST, full insert sequence. | 0.536 |
| NM\_011710 | Mus musculus tryptophanyl-tRNA synthetase (Wars), mRNA | 0.536 |
| NM\_028762 | Mus musculus RIKEN cDNA 1200009A02 gene (1200009A02Rik), mRNA | 0.536 |
| NM\_026201 | Mus musculus cell division cycle and apoptosis regulator 1 (Ccar1), mRNA | 0.536 |
| AK019760 | Mus musculus adult male testis cDNA, RIKEN full-length enriched library, clone:4930552N12 product:hypothetical protein, full insert sequence. | 0.535 |
| NM\_134471 | Mus musculus kinesin family member 2C (Kif2c), mRNA | 0.535 |
| XM\_356099 | Mus musculus RIKEN cDNA 2400006P09 gene (2400006P09Rik), mRNA | 0.535 |
| XM\_282971 | Mus musculus whn-dependent transcript 3 (Wdt3-pending), mRNA | 0.535 |
| NM\_011233 | Mus musculus RAD17 homolog (S. pombe) (Rad17), mRNA | 0.535 |
| AK040867 | Mus musculus adult male aorta and vein cDNA, RIKEN full-length enriched library, clone:A530031P09 product:phosphatidylinositol 3-kinase catalytic delta polypeptide, full insert sequence. | 0.535 |
| XM\_133622 | Mus musculus hypothetical protein Hbxap (Hbxap), mRNA | 0.535 |
| NM\_053089 | Mus musculus NMDA receptor-regulated gene 1 (Narg1), mRNA | 0.535 |
| NM\_025935 | Mus musculus TBC1 domain family, member 7 (Tbc1d7), mRNA | 0.535 |
| AK076286 | Mus musculus 0 day neonate skin cDNA, RIKEN full-length enriched library, clone:4632413C04 product:proliferation-associated 2G4, 38kD, full insert sequence | 0.535 |
| BC062179 | Mus musculus cDNA clone MGC:70141 IMAGE:6333817, complete cds. | 0.534 |
| NM\_008229 | Mus musculus histone deacetylase 2 (Hdac2), mRNA | 0.534 |
| NM\_133992 | Mus musculus ubiquitin specific protease 52 (Usp52), mRNA | 0.534 |
| AK013338 | Mus musculus 10, 11 days embryo whole body cDNA, RIKEN full-length enriched library, clone:2810453L12 product:hypothetical protein, full insert sequence. | 0.534 |
| NM\_133928 | Mus musculus coiled-coil-helix-coiled-coil-helix domain containing 4 (Chchd4), mRNA | 0.533 |
| NM\_011794 | Mus musculus bisphosphate 3'-nucleotidase 1 (Bpnt1), mRNA | 0.533 |
| NM\_026035 | Mus musculus mitochondrial ribosomal protein L55 (Mrpl55), mRNA | 0.533 |
| NM\_177337 | Mus musculus RIKEN cDNA C730007L20 gene (C730007L20Rik), mRNA | 0.533 |
| NM\_178084 | Mus musculus RIKEN cDNA B230120H23 gene (B230120H23Rik), mRNA | 0.533 |
| NM\_023275 | Mus musculus ras homolog gene family, member J (Rhoj), mRNA | 0.533 |
| NM\_013493 | Mus musculus cellular nucleic acid binding protein 1 (Cnbp1), mRNA | 0.533 |
| NM\_031881 | Mus musculus neural precursor cell expressed, developmentally down-regulated gene 4-like (Nedd4l), mRNA | 0.532 |
| NM\_021384 | Mus musculus RIKEN cDNA 2510004L01 gene (2510004L01Rik), mRNA | 0.532 |
| NM\_008085 | Mus musculus glyceraldehyde-3-phosphate dehydrogenase, spermatogenic (Gapds), mRNA | 0.532 |
| NM\_016723 | Mus musculus ubiquitin carboxyl-terminal esterase L3 (ubiquitin thiolesterase) (Uchl3), mRNA | 0.532 |
| NM\_001002008 | Mus musculus cDNA sequence BC049807 (BC049807), mRNA | 0.532 |
| NAP000004-017 | Unknown | 0.532 |
| NM\_010492 | Mus musculus islet cell autoantigen 1 (Ica1), mRNA | 0.532 |
| NM\_022987 | Mus musculus zinc finger protein of the cerebellum 5 (Zic5), mRNA | 0.532 |
| NM\_013898 | Mus musculus translocase of inner mitochondrial membrane 8 homolog a (yeast) (Timm8a), mRNA | 0.532 |
| NM\_145381 | Mus musculus lactamase, beta 2 (Lactb2), mRNA | 0.532 |
| NM\_010499 | Mus musculus immediate early response 2 (Ier2), mRNA | 0.532 |
| AK080746 | Mus musculus adult retina cDNA, RIKEN full-length enriched library, clone:A930035J23 product:unknown EST, full insert sequence. | 0.532 |
| NM\_146613 | Mus musculus olfactory receptor 973 (Olfr973), mRNA | 0.531 |
| NM\_020282 | Mus musculus NAD(P)H dehydrogenase, quinone 2 (Nqo2), mRNA | 0.531 |
| BC050854 | Mus musculus asparagine-linked glycosylation 6 homolog (yeast, alpha-1,3,-glucosyltransferase), mRNA (cDNA clone IMAGE:6332345), partial cds. | 0.531 |
| NM\_009193 | Mus musculus stem-loop binding protein (Slbp), mRNA | 0.531 |
| NM\_177331 | Mus musculus RIKEN cDNA 5830483C08 gene (5830483C08Rik), mRNA | 0.531 |
| NM\_026393 | Mus musculus RIKEN cDNA 1110025F24 gene (1110025F24Rik), mRNA | 0.531 |
| NM\_015774 | Mus musculus ERO1-like (S. cerevisiae) (Ero1l), mRNA | 0.530 |
| NM\_024214 | Mus musculus translocase of outer mitochondrial membrane 20 homolog (yeast) (Tomm20), mRNA | 0.530 |
| NM\_134059 | Mus musculus DEAD (Asp-Glu-Ala-Asp) box polypeptide 41 (Ddx41), mRNA | 0.530 |
| NM\_026858 | Mus musculus RIKEN cDNA 1110068E08 gene (1110068E08Rik), mRNA | 0.529 |
| NM\_175121 | Mus musculus solute carrier family 38, member 2 (Slc38a2), mRNA | 0.529 |
| NAP019608-001 | Unknown | 0.529 |
| AK046811 | Mus musculus 10 days neonate medulla oblongata cDNA, RIKEN full-length enriched library, clone:B830013D20 product:unknown EST, full insert sequence | 0.529 |
| NM\_010193 | Mus musculus feminization 1 homolog b (C. elegans) (Fem1b), mRNA | 0.529 |
| NM\_016661 | Mus musculus S-adenosylhomocysteine hydrolase (Ahcy), mRNA | 0.527 |
| NM\_027950 | Mus musculus RIKEN cDNA 1700012B18 gene (1700012B18Rik), mRNA | 0.527 |
| BC039801 | Mus musculus RIKEN cDNA 3110003A17 gene, mRNA (cDNA clone IMAGE:1448067), partial cds. | 0.527 |
| XM\_126172 | Mus musculus la related protein (Larp-pending), mRNA | 0.527 |
| NM\_009698 | Mus musculus adenine phosphoribosyl transferase (Aprt), mRNA | 0.527 |
| AK011836 | Mus musculus 10 days embryo whole body cDNA, RIKEN full-length enriched library, clone:2610110A13 product:hypothetical EF-hand/Aminoacyl-transfer RNA synthetases class-II containing protein, full insert sequence. | 0.527 |
| NM\_028091 | Mus musculus O-sialoglycoprotein endopeptidase-like 1 (Osgepl1), mRNA | 0.527 |
| NM\_199026 | Mus musculus phosphatidylinositol glycan, class L (Pigl), mRNA | 0.527 |
| XM\_193319 | Mus musculus similar to Methionine adenosyltransferase II, alpha (LOC269628), mRNA | 0.527 |
| NM\_015731 | Mus musculus ATPase, class II, type 9A (Atp9a), mRNA | 0.527 |
| NM\_030715 | Mus musculus polymerase (DNA directed), eta (RAD 30 related) (Polh), mRNA | 0.526 |
| NM\_172502 | Mus musculus cDNA sequence BC057552 (BC057552), mRNA | 0.526 |
| BC024125 | Mus musculus E2F transcription factor 3, mRNA (cDNA clone IMAGE:5099645), partial cds. | 0.526 |
| AF095938 | Mus musculus succinate dehydrogenase Fp subunit mRNA, partial cds. | 0.526 |
| U59758 | Mus musculus p53-variant (p53) mRNA, partial cds. | 0.526 |
| AK016638 | Mus musculus adult male testis cDNA, RIKEN full-length enriched library, clone:4933403K24 product:hypothetical protein, full insert sequence. | 0.526 |
| NM\_172410 | Mus musculus nucleoporin 93 (Nup93), mRNA | 0.526 |
| NM\_213733 | Mus musculus aminopeptidase-like 1 (Npepl1), mRNA | 0.525 |
| NM\_010598 | Mus musculus potassium voltage-gated channel, shaker-related subfamily, beta member 2 (Kcnab2), mRNA | 0.525 |
| NM\_008710 | Mus musculus nicotinamide nucleotide transhydrogenase (Nnt), mRNA | 0.525 |
| AK083178 | Mus musculus adult male hippocampus cDNA, RIKEN full-length enriched library, clone:C630023P15 product:meiosis-specific nuclear structural protein 1, full insert sequence. | 0.525 |
| NM\_007634 | Mus musculus cyclin F (Ccnf), mRNA | 0.525 |
| AK011403 | Mus musculus 10 days embryo whole body cDNA, RIKEN full-length enriched library, clone:2610015J01 product:SIMILAR TO S164 PROTEIN homolog [Mus musculus], full insert sequence. | 0.525 |
| NM\_028339 | Mus musculus thioredoxin domain containing 1 (Txndc1), mRNA | 0.525 |
| XM\_148854 | Mus musculus RIKEN cDNA 1110006O17 gene (1110006O17Rik), mRNA | 0.525 |
| XM\_129647 | Mus musculus glutamyl-prolyl-tRNA synthetase (Eprs), mRNA | 0.525 |
| AK129202 | Mus musculus mRNA for mKIAA0731 protein. | 0.525 |
| NM\_025853 | Mus musculus RIKEN cDNA 1700022L09 gene (1700022L09Rik), mRNA | 0.524 |
| NM\_013742 | Mus musculus cysteinyl-tRNA synthetase (Cars), mRNA | 0.524 |
| BC056964 | Mus musculus RIKEN cDNA 4930553M18 gene, mRNA (cDNA clone IMAGE:6827717), partial cds | 0.524 |
| AK029300 | Mus musculus 0 day neonate head cDNA, RIKEN full-length enriched library, clone:4832416J22 product:unknown EST, full insert sequence | 0.524 |
| AK049476 | Mus musculus 7 days embryo whole body cDNA, RIKEN full-length enriched library, clone:C430015M08 product:hypothetical FKBP-type peptidyl-prolyl cis-trans isomerase domain profile/FKBP-type peptidyl-prolyl cis-trans isomerase (PPIase) containing protein, | 0.524 |
| NM\_008258 | Mus musculus hematological and neurological expressed sequence 1 (Hn1), mRNA | 0.523 |
| NM\_178716 | Mus musculus transportin 1 (Tnpo1), mRNA | 0.523 |
| NM\_133807 | Mus musculus expressed sequence AA959742 (AA959742), mRNA | 0.523 |
| NM\_009837 | Mus musculus chaperonin subunit 4 (delta) (Cct4), mRNA | 0.523 |
| NM\_010830 | Mus musculus mutS homolog 6 (E. coli) (Msh6), mRNA | 0.523 |
| XM\_140993 | Mus musculus hypothetical protein A630086N24 (A630086N24), mRNA | 0.522 |
| NM\_008442 | Mus musculus kinesin family member 2A (Kif2a), mRNA | 0.522 |
| NM\_017397 | Mus musculus DEAD (Asp-Glu-Ala-Asp) box polypeptide 20 (Ddx20), mRNA | 0.522 |
| NM\_146011 | Mus musculus Rho GTPase activating protein 9 (Arhgap9), mRNA | 0.522 |
| AK010559 | Mus musculus ES cells cDNA, RIKEN full-length enriched library, clone:2410019P08 product:hypothetical Proline-rich region/Zinc finger, C2H2 type containing protein, full insert sequence. | 0.522 |
| NM\_172947 | Mus musculus RIKEN cDNA 2600001B17 gene (2600001B17Rik), mRNA | 0.521 |
| NM\_015762 | Mus musculus thioredoxin reductase 1 (Txnrd1), mRNA | 0.521 |
| NM\_019774 | Mus musculus A kinase (PRKA) anchor protein 8 (Akap8), mRNA | 0.521 |
| AK010772 | Mus musculus ES cells cDNA, RIKEN full-length enriched library, clone:2410118I19 product:CDNA FLJ12545 FIS, CLONE NT2RM4000611, WEAKLY SIMILAR TO VEGETATIBLE INCOMPATIBILITY PROTEIN HET-E-1 (FRAGMENT) homolog [Homo sapiens], full insert sequence. [AK010 | 0.521 |
| NM\_170760 | Mus musculus U2 small nuclear RNA auxiliary factor 1-like 4 (U2af1l4), mRNA | 0.521 |
| NM\_138656 | Mus musculus mevalonate (diphospho) decarboxylase (Mvd), mRNA | 0.520 |
| NM\_013489 | Mus musculus CD84 antigen (Cd84), mRNA | 0.520 |
| NAP101637-1 | Unknown | 0.520 |
| NM\_026653 | Mus musculus replication protein A1 (Rpa1), mRNA | 0.520 |
| NM\_009684 | Mus musculus apoptotic protease activating factor 1 (Apaf1), mRNA | 0.520 |
| NM\_016918 | Mus musculus nudix (nucleoside diphosphate linked moiety X)-type motif 5 (Nudt5), mRNA | 0.520 |
| NM\_013812 | Mus musculus CDK2 (cyclin-dependent kinase 2)-associated protein 1 (Cdk2ap1), mRNA | 0.519 |
| NM\_016804 | Mus musculus metaxin 2 (Mtx2), mRNA | 0.519 |
| NM\_178798 | Mus musculus expressed sequence AI643885 (AI643885), mRNA | 0.519 |
| AK011422 | Mus musculus 10 days embryo whole body cDNA, RIKEN full-length enriched library, clone:2610016K01 product:OVARC1001161 PROTEIN homolog [Homo sapiens], full insert sequence. | 0.519 |
| NAP025648-001 | Unknown | 0.519 |
| NM\_026382 | Mus musculus RIKEN cDNA 6530403A03 gene (6530403A03Rik), mRNA | 0.519 |
| NM\_021539 | Mus musculus WD repeat and SOCS box-containing 2 (Wsb2), mRNA | 0.519 |
| NM\_028597 | Mus musculus THO complex 3 (Thoc3), mRNA | 0.518 |
| NM\_029705 | Mus musculus Machado-Joseph disease (spinocerebellar ataxia 3, olivopontocerebellar ataxia 3, autosomal dominant, ataxin 3) homolog (human) (Mjd), mRNA | 0.518 |
| NM\_025710 | Mus musculus ubiquinol-cytochrome c reductase, Rieske iron-sulfur polypeptide 1 (Uqcrfs1), mRNA | 0.517 |
| BC034507 | Mus musculus cDNA sequence BC034507, mRNA (cDNA clone MGC:30495 IMAGE:4222393), complete cds. | 0.517 |
| NM\_028177 | Mus musculus NADH dehydrogenase (ubiquinone) 1, alpha/beta subcomplex, 1 (Ndufab1), mRNA | 0.517 |
| NM\_138592 | Mus musculus ubiquitin specific protease 39 (Usp39), mRNA | 0.517 |
| NM\_025495 | Mus musculus RIKEN cDNA 1700022C02 gene (1700022C02Rik), mRNA | 0.517 |
| NM\_007404 | Mus musculus a disintegrin and metalloproteinase domain 9 (meltrin gamma) (Adam9), mRNA | 0.516 |
| NM\_025591 | Mus musculus RIKEN cDNA 2010309E21 gene (2010309E21Rik), mRNA | 0.516 |
| NM\_026008 | Mus musculus RIKEN cDNA 2610207F23 gene (2610207F23Rik), mRNA | 0.516 |
| NM\_175187 | Mus musculus RIKEN cDNA 2810446P07 gene (2810446P07Rik), mRNA | 0.516 |
| NM\_008014 | Mus musculus protein phosphatase 1G (formerly 2C), magnesium-dependent, gamma isoform (Ppm1g), mRNA | 0.516 |
| NM\_021415 | Mus musculus calcium channel, voltage-dependent, T type, alpha 1H subunit (Cacna1h), mRNA | 0.516 |
| NM\_145523 | Mus musculus grancalcin (Gca), mRNA | 0.516 |
| NM\_026310 | Mus musculus mitochondrial ribosomal protein L18 (Mrpl18), mRNA | 0.516 |
| NAP001627-002 | Unknown | 0.515 |
| NM\_016692 | Mus musculus inner centromere protein (Incenp), mRNA | 0.515 |
| BC048905 | Mus musculus RIKEN cDNA B230312B02 gene, mRNA (cDNA clone IMAGE:3489827), partial cds. | 0.515 |
| NM\_022653 | Mus musculus thimet oligopeptidase 1 (Thop1), mRNA | 0.515 |
| NM\_009004 | Mus musculus kinesin family member 20A (Kif20a), mRNA | 0.515 |
| NM\_008482 | Mus musculus laminin B1 subunit 1 (Lamb1-1), mRNA | 0.515 |
| NM\_007790 | Mus musculus chondroitin sulfate proteoglycan 6 (Cspg6), mRNA | 0.514 |
| XM\_110743 | Mus musculus similar to mitochondrial solute carrier protein (LOC194744), mRNA | 0.514 |
| NM\_007808 | Mus musculus cytochrome c, somatic (Cycs), mRNA | 0.514 |
| AK021027 | Mus musculus 4 days neonate thymus cDNA, RIKEN full-length enriched library, clone:B630009I04 product:similar to ASC-1 COMPLEX SUBUNIT P200 (FRAGMENT) [Homo sapiens], full insert sequence. | 0.514 |
| NM\_030014 | Mus musculus hook homolog 1 (Drosophila) (Hook1), mRNA | 0.514 |
| BM934075 | UI-M-CG0p-bmo-c-09-0-UI.r1 NIH\_BMAP\_Ret4\_S2 Mus musculus cDNA clone UI-M-CG0p-bmo-c-09-0-UI 5', mRNA sequence | 0.514 |
| NM\_028232 | Mus musculus shugoshin-like 1 (S. pombe) (Sgol1), mRNA | 0.514 |
| NM\_134063 | Mus musculus cDNA sequence BC016423 (BC016423), mRNA | 0.514 |
| NM\_181595 | Mus musculus protein phosphatase 1, regulatory (inhibitor) subunit 9A (Ppp1r9a), mRNA | 0.514 |
| NM\_013853 | Mus musculus ATP-binding cassette, sub-family F (GCN20), member 2 (Abcf2), mRNA | 0.513 |
| NM\_018785 | Mus musculus formin binding protein 3 (Fnbp3), mRNA | 0.513 |
| AK129075 | Mus musculus mRNA for mKIAA0173 protein. | 0.513 |
| NM\_010497 | Mus musculus isocitrate dehydrogenase 1 (NADP+), soluble (Idh1), mRNA | 0.513 |
| NM\_008667 | Mus musculus Ngfi-A binding protein 1 (Nab1), mRNA | 0.513 |
| AK042393 | Mus musculus 3 days neonate thymus cDNA, RIKEN full-length enriched library, clone:A630087P08 product:replication factor C (activator 1) 3 (38 kDa), full insert sequence. | 0.512 |
| NM\_015816 | Mus musculus LSM4 homolog, U6 small nuclear RNA associated (S. cerevisiae) (Lsm4), mRNA | 0.512 |
| NM\_178914 | Mus musculus expressed sequence AI661438 (AI661438), mRNA | 0.512 |
| NM\_008379 | Mus musculus karyopherin (importin) beta 1 (Kpnb1), mRNA | 0.512 |
| NM\_009088 | Mus musculus RNA polymerase 1-4 (Rpo1-4), mRNA | 0.512 |
| NM\_172594 | Mus musculus DEAH (Asp-Glu-Ala-His) box polypeptide 29 (Dhx29), mRNA | 0.512 |
| AF197159 | Mus musculus cubilin mRNA, partial cds. | 0.511 |
| NM\_175554 | Mus musculus RIKEN cDNA E130314M08 gene (E130314M08Rik), mRNA | 0.511 |
| NM\_178918 | Mus musculus expressed sequence AW544865 (AW544865), mRNA | 0.511 |
| NM\_020007 | Mus musculus muscleblind-like 1 (Drosophila) (Mbnl1), mRNA | 0.511 |
| NAP060490-1 | Unknown | 0.511 |
| NM\_010250 | Mus musculus gamma-aminobutyric acid (GABA-A) receptor, subunit alpha 1 (Gabra1), mRNA | 0.511 |
| NM\_011270 | Mus musculus Rhesus blood group CE and D (Rhced), mRNA | 0.511 |
| NM\_010792 | Mus musculus methyltransferase-like 1 (Mettl1), mRNA | 0.510 |
| BC004827 | Mus musculus phosphoserine aminotransferase 1, mRNA (cDNA clone MGC:6462 IMAGE:2616298), complete cds. | 0.510 |
| AK079035 | Mus musculus adult male epididymis cDNA, RIKEN full-length enriched library, clone:9230115A19 product:ATP-DEPENDENT CHROMATIN REMODELING PROTEIN SNF2H homolog [Mus musculus], full insert sequence. | 0.509 |
| BC029086 | Mus musculus mRNA similar to DKFZP566O1646 protein (cDNA clone MGC:27788 IMAGE:3156934), complete cds. | 0.509 |
| NM\_013722 | Mus musculus synapsin III (Syn3), mRNA | 0.509 |
| NM\_153546 | Mus musculus O-acyltransferase (membrane bound) domain containing 1 (Oact1), mRNA | 0.509 |
| AK049446 | Mus musculus 7 days embryo whole body cDNA, RIKEN full-length enriched library, clone:C430013M08 product:NUCLEAR TRANSPORT RECEPTOR homolog [Homo sapiens], full insert sequence. | 0.509 |
| NM\_008111 | Mus musculus guanosine diphosphate (GDP) dissociation inhibitor 2 (Gdi2), mRNA | 0.509 |
| BC068168 | Mus musculus neuropilin (NRP) and tolloid (TLL)-like 2, mRNA (cDNA clone IMAGE:30544936), with apparent retained intron | 0.509 |
| NM\_009128 | Mus musculus stearoyl-Coenzyme A desaturase 2 (Scd2), mRNA | 0.508 |
| NM\_025377 | Mus musculus RIKEN cDNA 1110001A07 gene (1110001A07Rik), mRNA | 0.508 |
| ENSMUST00000069200 | Unknown | 0.508 |
| NM\_009388 | Mus musculus transketolase (Tkt), mRNA | 0.508 |
| NM\_153419 | Mus musculus glutamate-rich WD repeat containing 1 (Grwd1), mRNA | 0.507 |
| NM\_144826 | Mus musculus RIKEN cDNA 4732497O03 gene (4732497O03Rik), mRNA | 0.507 |
| AF487281 | Mus musculus prokineticin 1 (Pk1) mRNA, partial cds. | 0.507 |
| BC054362 | Mus musculus cDNA sequence BC010304, mRNA (cDNA clone IMAGE:5027838), partial cds. | 0.507 |
| AF194970 | Mus musculus LEK1 mRNA, partial cds. | 0.507 |
| NM\_025546 | Mus musculus RIKEN cDNA 2410005K20 gene (2410005K20Rik), mRNA | 0.506 |
| NM\_027296 | Mus musculus tRNA nucleotidyl transferase, CCA-adding, 1 (Trnt1), mRNA | 0.506 |
| NM\_019414 | Mus musculus selenium binding protein 2 (Selenbp2), mRNA | 0.506 |
| XM\_358223 | Mus musculus similar to High mobility group protein 1 (HMG-1) (Amphoterin) (Heparin-binding protein p30) (LOC385395), mRNA | 0.505 |
| TC1066374 | UNRI\_MOUSE UNR-interacting protein (Serine-threonine kinase receptor-associated protein). [Mouse] {Mus musculus}, complete | 0.505 |
| AK122485 | Mus musculus mRNA for mKIAA1284 protein. | 0.504 |
| NM\_009836 | Mus musculus chaperonin subunit 3 (gamma) (Cct3), mRNA | 0.504 |
| TC1010869 | SFR3\_HUMAN Splicing factor, arginine/serine-rich 3 (Pre-mRNA splicing factor SRP20) (X16 protein). [Mouse] {Mus musculus}, partial (55%) | 0.504 |
| NM\_145556 | Mus musculus TAR DNA binding protein (Tardbp), mRNA | 0.504 |
| ENSMUST00000038707 | Unknown | 0.503 |
| AK010126 | Mus musculus adult male tongue cDNA, RIKEN full-length enriched library, clone:2310069F09 product:similar to RNA POLYMERASE III SUBUNIT [Homo sapiens], full insert sequence. | 0.503 |
| NM\_027275 | Mus musculus RIKEN cDNA 2810422B04 gene (2810422B04Rik), mRNA | 0.503 |
| BC047049 | Mus musculus UPF3 regulator of nonsense transcripts homolog B (yeast), mRNA (cDNA clone IMAGE:5358431), partial cds. | 0.503 |
| NM\_028315 | Mus musculus RIKEN cDNA 2810028N01 gene (2810028N01Rik), mRNA | 0.502 |
| BC040800 | Mus musculus kinesin family member 5B, mRNA (cDNA clone IMAGE:3985895), partial cds. | 0.502 |
| NM\_011991 | Mus musculus COP9 (constitutive photomorphogenic) homolog, subunit 3 (Arabidopsis thaliana) (Cops3), mRNA | 0.502 |
| NM\_008613 | Mus musculus meiosis-specific nuclear structural protein 1 (Mns1), mRNA | 0.501 |
| NM\_009838 | Mus musculus chaperonin subunit 6a (zeta) (Cct6a), mRNA | 0.501 |
| NM\_029673 | Mus musculus inner membrane protein, mitochondrial (Immt), mRNA | 0.501 |
| NM\_023210 | Mus musculus acidic (leucine-rich) nuclear phosphoprotein 32 family, member E (Anp32e), mRNA | 0.501 |
| NM\_011155 | Mus musculus protein phosphatase 5, catalytic subunit (Ppp5c), mRNA | 0.501 |
| NM\_146217 | Mus musculus alanyl-tRNA synthetase (Aars), mRNA | 0.501 |
| NM\_009227 | Mus musculus small nuclear ribonucleoprotein E (Snrpe), mRNA | 0.501 |
| NM\_013691 | Mus musculus thrombospondin 3 (Thbs3), mRNA | 0.500 |
| NM\_026499 | Mus musculus splicing factor, arginine/serine-rich 6 (Sfrs6), mRNA | 0.500 |
| NM\_009108 | Mus musculus nuclear receptor subfamily 1, group H, member 4 (Nr1h4), mRNA | 0.500 |
| Z11886 | M.musculus notch-1 mRNA. | 0.500 |
| TC1020264 | AB091123 PEM-3 {Halocynthia roretzi}, partial (29%) | 0.499 |
| BC012871 | Mus musculus phosphatase and actin regulator 2, mRNA (cDNA clone IMAGE:3482264), partial cds. | 0.499 |
| NM\_144958 | Mus musculus eukaryotic translation initiation factor 4A1 (Eif4a1), mRNA | 0.499 |
| AK129034 | Mus musculus mRNA for mKIAA0013 protein. | 0.499 |
| BC038050 | Mus musculus RIKEN cDNA G630013P12 gene, mRNA (cDNA clone IMAGE:3497828), with apparent retained intron. | 0.498 |
| NM\_146159 | Mus musculus cDNA sequence BC023882 (BC023882), mRNA | 0.498 |
| NM\_175294 | Mus musculus RIKEN cDNA 8430423A01 gene (8430423A01Rik), mRNA | 0.498 |
| NM\_010634 | Mus musculus fatty acid binding protein 5, epidermal (Fabp5), mRNA | 0.497 |
| TC1057697 | CI10\_HUMAN Protein C9orf10. [Human] {Homo sapiens}, partial (26%) | 0.497 |
| M18373 | Mouse amyloid beta protein precursor, complete cds. | 0.497 |
| NM\_011431 | Mus musculus U5 small nuclear ribonucleoprotein (Snrp116), mRNA | 0.497 |
| TC1031811 | AY063750 WD repeat protein Gemin5 {Homo sapiens}, partial (92%) | 0.497 |
| NM\_012055 | Mus musculus asparagine synthetase (Asns), mRNA | 0.497 |
| NM\_178605 | Mus musculus DNA segment, Chr 13, Wayne State University 177, expressed (D13Wsu177e), mRNA | 0.497 |
| NM\_007633 | Mus musculus cyclin E1 (Ccne1), mRNA | 0.497 |
| NM\_008549 | Mus musculus mannosidase 2, alpha 1 (Man2a1), mRNA | 0.497 |
| AK017664 | Mus musculus 8 days embryo whole body cDNA, RIKEN full-length enriched library, clone:5730454C12 product:inferred: amidophosphoribosyltransferase precursor {Rattus norvegicus}, full insert sequence. | 0.496 |
| NM\_016739 | Mus musculus GPI-anchored membrane protein 1 (Gpiap1), mRNA | 0.496 |
| NM\_172558 | Mus musculus gem (nuclear organelle) associated protein 5 (Gemin5), mRNA | 0.496 |
| NM\_008112 | Mus musculus guanosine diphosphate (GDP) dissociation inhibitor 3 (Gdi3), mRNA | 0.496 |
| NM\_009530 | Mus musculus alpha thalassemia/mental retardation syndrome X-linked homolog (human) (Atrx), mRNA | 0.496 |
| NM\_022988 | Mus musculus Ngg1 interacting factor 3-like 1 (S. pombe) (Nif3l1), mRNA | 0.496 |
| AK010783 | Mus musculus ES cells cDNA, RIKEN full-length enriched library, clone:2410127E18 product:hypothetical Kelch repeat containing protein, full insert sequence. | 0.495 |
| NM\_019441 | Mus musculus palmitoyl-protein thioesterase 2 (Ppt2), mRNA | 0.495 |
| NM\_199145 | Mus musculus RIKEN cDNA 3110062M04 gene (3110062M04Rik), mRNA | 0.494 |
| BC055464 | Mus musculus RIKEN cDNA 2610101J03 gene, mRNA (cDNA clone IMAGE:3984120), partial cds. | 0.494 |
| NM\_133216 | Mus musculus X-prolyl aminopeptidase (aminopeptidase P) 1, soluble (Xpnpep1), mRNA | 0.494 |
| NM\_019468 | Mus musculus glucose-6-phosphate dehydrogenase 2 (G6pd2), mRNA | 0.494 |
| NM\_009870 | Mus musculus cyclin-dependent kinase 4 (Cdk4), mRNA | 0.494 |
| NAP027859-1 | Unknown | 0.494 |
| XM\_149793 | Mus musculus RIKEN cDNA 1700013D24 gene (1700013D24Rik), mRNA | 0.493 |
| BC037150 | Mus musculus cDNA sequence BC018347, mRNA (cDNA clone IMAGE:5148558), partial cds. | 0.493 |
| AK011942 | Mus musculus 10 days embryo whole body cDNA, RIKEN full-length enriched library, clone:2610300B10 product:hypothetical protein, full insert sequence. | 0.492 |
| AK032739 | Mus musculus 12 days embryo male wolffian duct includes surrounding region cDNA, RIKEN full-length enriched library, clone:6720422P08 product:unknown EST, full insert sequence | 0.492 |
| A\_52\_P549754 | Unknown | 0.492 |
| AK077578 | Mus musculus 8 days embryo whole body cDNA, RIKEN full-length enriched library, clone:5730455C01 product:hypothetical RNA-binding domain, RBD structure containing protein, full insert sequence. | 0.492 |
| NM\_025537 | Mus musculus RIKEN cDNA 2310050B20 gene (2310050B20Rik), mRNA | 0.492 |
| NM\_010700 | Mus musculus low density lipoprotein receptor (Ldlr), mRNA | 0.492 |
| AK032842 | Mus musculus 12 days embryo male wolffian duct includes surrounding region cDNA, RIKEN full-length enriched library, clone:6720461H18 product:unknown EST, full insert sequence. | 0.492 |
| NM\_172518 | Mus musculus RIKEN cDNA 6720460I06 gene (6720460I06Rik), mRNA | 0.492 |
| NM\_145452 | Mus musculus RAS p21 protein activator 1 (Rasa1), mRNA | 0.492 |
| NM\_019748 | Mus musculus ubiquitin-like 1 (sentrin) activating enzyme E1A (Uble1a), mRNA | 0.491 |
| NM\_029797 | Mus musculus RIKEN cDNA 2610034E18 gene (2610034E18Rik), mRNA | 0.491 |
| NM\_008298 | Mus musculus DnaJ (Hsp40) homolog, subfamily A, member 1 (Dnaja1), mRNA | 0.491 |
| NM\_144554 | Mus musculus tribbles homolog 3 (Drosophila) (Trib3), mRNA | 0.491 |
| AK044090 | Mus musculus 10 days neonate cortex cDNA, RIKEN full-length enriched library, clone:A830089D24 product:hypothetical protein, full insert sequence. | 0.491 |
| NM\_013562 | Mus musculus interferon-related developmental regulator 1 (Ifrd1), mRNA | 0.491 |
| NM\_133747 | Mus musculus RIKEN cDNA 2810405J04 gene (2810405J04Rik), mRNA | 0.491 |
| AK031771 | Mus musculus 11 days embryo head cDNA, RIKEN full-length enriched library, clone:6230417E10 product:weakly similar to SHC TRANSFORMING PROTEIN [Homo sapiens], full insert sequence. | 0.490 |
| NM\_026309 | Mus musculus LSM3 homolog, U6 small nuclear RNA associated (S. cerevisiae) (Lsm3), mRNA | 0.490 |
| NM\_007763 | Mus musculus cysteine-rich protein 1 (intestinal) (Crip1), mRNA | 0.490 |
| AK053594 | Mus musculus 0 day neonate eyeball cDNA, RIKEN full-length enriched library, clone:E130112M23 product:dachshund 1 (Drosophila), full insert sequence. | 0.490 |
| NM\_016905 | Mus musculus galactokinase 1 (Galk1), mRNA | 0.490 |
| AK078542 | Mus musculus 12 days embryo female mullerian duct includes surrounding region cDNA, RIKEN full-length enriched library, clone:6820443O06 product:NUANCE (FRAGMENT) homolog [Mus musculus], full insert sequence | 0.490 |
| NM\_013536 | Mus musculus gene rich cluster, C2f gene (Grcc2f), mRNA | 0.490 |
| NM\_172253 | Mus musculus TWIST neighbor (Twistnb), mRNA | 0.490 |
| NM\_008541 | Mus musculus MAD homolog 5 (Drosophila) (Smad5), mRNA | 0.489 |
| NM\_009716 | Mus musculus activating transcription factor 4 (Atf4), mRNA | 0.489 |
| NM\_030717 | Mus musculus lactamase, beta (Lactb), mRNA | 0.489 |
| NM\_178869 | Mus musculus tubulin tyrosine ligase-like 1 (Ttll1), mRNA | 0.489 |
| NM\_008949 | Mus musculus proteasome (prosome, macropain) 26S subunit, ATPase 3, interacting protein (Psmc3ip), mRNA | 0.489 |
| AY035702 | Mus musculus Ets-1 mRNA, partial cds, alternatively spliced. | 0.489 |
| NM\_025564 | Mus musculus RIKEN cDNA 2010012C16 gene (2010012C16Rik), mRNA | 0.489 |
| NM\_020619 | Mus musculus glucosidase 1 (Gcs1), mRNA | 0.489 |
| NM\_177445 | Mus musculus aspartyl-tRNA synthetase (Dars), mRNA | 0.489 |
| NM\_027592 | Mus musculus RIKEN cDNA 4921516M08 gene (4921516M08Rik), mRNA | 0.489 |
| NM\_027945 | Mus musculus RIKEN cDNA 1700007H16 gene (1700007H16Rik), mRNA | 0.489 |
| NAP026936-1 | Unknown | 0.489 |
| NM\_009786 | Mus musculus calcyclin binding protein (Cacybp), mRNA | 0.488 |
| NM\_178113 | Mus musculus RIKEN cDNA B130055D15 gene (B130055D15Rik), mRNA | 0.488 |
| NM\_011692 | Mus musculus von Hippel-Lindau binding protein 1 (Vbp1), mRNA | 0.488 |
| NM\_019796 | Mus musculus synaptotagmin binding, cytoplasmic RNA interacting protein (Syncrip), mRNA | 0.488 |
| ENSMUST00000061648 | Unknown | 0.488 |
| AK013434 | Mus musculus 10, 11 days embryo whole body cDNA, RIKEN full-length enriched library, clone:2810482I07 product:unclassifiable, full insert sequence. | 0.488 |
| A\_51\_P262202 | Unknown | 0.487 |
| AK090184 | Mus musculus 11 days embryo spinal cord cDNA, RIKEN full-length enriched library, clone:G630013P12 product:weakly similar to KIAA1689 PROTEIN (FRAGMENT) [Homo sapiens], full insert sequence. | 0.487 |
| AJ250191 | Mus musculus partial mRNA for stretch responsive protein 278 (sr278 gene). | 0.487 |
| NM\_013907 | Mus musculus F-box and WD-40 domain protein 4 (Fbxw4), mRNA | 0.487 |
| NM\_054102 | Mus musculus influenza virus NS1A binding protein (Ivns1abp), mRNA | 0.487 |
| AK019989 | Mus musculus 8 days embryo whole body cDNA, RIKEN full-length enriched library, clone:5730594O13 product:HUPF3B homolog [Homo sapiens], full insert sequence. | 0.487 |
| NM\_009014 | Mus musculus RAD51-like 1 (S. cerevisiae) (Rad51l1), mRNA | 0.487 |
| NM\_133362 | Mus musculus erythroid differentiation regulator 1 (Erdr1), mRNA | 0.487 |
| NM\_011241 | Mus musculus RAN GTPase activating protein 1 (Rangap1), mRNA | 0.486 |
| AK014084 | Mus musculus 13 days embryo head cDNA, RIKEN full-length enriched library, clone:3110025H23 product:hypothetical protein, full insert sequence. | 0.486 |
| NM\_009226 | Mus musculus small nuclear ribonucleoprotein D1 (Snrpd1), mRNA | 0.485 |
| ENSMUST00000053085 | 603311654F1 NCI\_CGAP\_Mam6 Mus musculus cDNA clone IMAGE:5351940 5', mRNA sequence [BI692008] | 0.485 |
| NM\_008821 | Mus musculus plasmacytoma expressed transcript 2 (Pet2), mRNA | 0.485 |
| NM\_173400 | Mus musculus RIKEN cDNA 6230416J20 gene (6230416J20Rik), mRNA | 0.485 |
| NM\_013636 | Mus musculus protein phosphatase 1, catalytic subunit, gamma isoform (Ppp1cc), mRNA | 0.485 |
| NM\_177235 | Mus musculus RIKEN cDNA B230209C24 gene (B230209C24Rik), mRNA | 0.485 |
| AK030651 | Mus musculus 6 days neonate head cDNA, RIKEN full-length enriched library, clone:5430403J05 product:unclassifiable, full insert sequence | 0.484 |
| NM\_146081 | Mus musculus protein phosphatase 4, regulatory subunit 1 (Ppp4r1), mRNA | 0.484 |
| NM\_010330 | Mus musculus embigin (Emb), mRNA | 0.484 |
| NM\_016871 | Mus musculus translocase of outer mitochondrial membrane 40 homolog (yeast) (Tomm40), mRNA | 0.484 |
| AK051772 | Mus musculus 12 days embryo spinal ganglion cDNA, RIKEN full-length enriched library, clone:D130077A04 product:unclassifiable, full insert sequence | 0.483 |
| AK090370 | Mus musculus 11 days embryo spinal cord cDNA, RIKEN full-length enriched library, clone:G630067B08 product:hypothetical RFX DNA-binding domain containing protein, full insert sequence. | 0.483 |
| NM\_023716 | Mus musculus RIKEN cDNA 2410129E14 gene (2410129E14Rik), mRNA | 0.483 |
| NM\_028904 | Mus musculus RIKEN cDNA 4932432N11 gene (4932432N11Rik), mRNA | 0.482 |
| NM\_028582 | Mus musculus influenza virus NS1A binding protein (Ivns1abp), mRNA | 0.482 |
| NM\_021511 | Mus musculus RRS1 ribosome biogenesis regulator homolog (S. cerevisiae) (Rrs1), mRNA | 0.482 |
| NM\_025965 | Mus musculus signal sequence receptor, alpha (Ssr1), mRNA | 0.482 |
| NM\_024428 | Mus musculus RIKEN cDNA 2810410M20 gene (2810410M20Rik), mRNA | 0.481 |
| NM\_010256 | Mus musculus phosphoribosylglycinamide formyltransferase (Gart), mRNA | 0.481 |
| NM\_026934 | Mus musculus RIKEN cDNA 2610312B22 gene (2610312B22Rik), mRNA | 0.481 |
| NM\_053110 | Mus musculus glycoprotein (transmembrane) nmb (Gpnmb), mRNA | 0.481 |
| U43892 | Mus musculus ABC transporter-7 mRNA, partial cds. | 0.480 |
| AK014608 | Mus musculus 0 day neonate skin cDNA, RIKEN full-length enriched library, clone:4632434I11 product:hypothetical Cytochrome c family heme-binding site containing protein, full insert sequence. | 0.480 |
| NM\_025882 | Mus musculus polymerase (DNA-directed), epsilon 4 (p12 subunit) (Pole4), mRNA | 0.480 |
| NM\_019666 | Mus musculus synaptotagmin binding, cytoplasmic RNA interacting protein (Syncrip), mRNA | 0.480 |
| NM\_009189 | Mus musculus sine oculis-related homeobox 1 homolog (Drosophila) (Six1), mRNA | 0.480 |
| NAP028432-1 | Unknown | 0.480 |
| NM\_022314 | Mus musculus tropomyosin 3, gamma (Tpm3), mRNA | 0.480 |
| NM\_199035 | Mus musculus asparagine-linked glycosylation 8 homolog (yeast, alpha-1,3-glucosyltransferase) (Alg8), mRNA | 0.479 |
| AK020090 | Mus musculus 11 days embryo head cDNA, RIKEN full-length enriched library, clone:6230427J02 product:hypothetical protein, full insert sequence. | 0.479 |
| NAP021334-001 | Unknown | 0.479 |
| NM\_016670 | Mus musculus Pbx/knotted 1 homeobox (Pknox1), mRNA | 0.479 |
| M62766 | Mouse HMG-CoA reductase mRNA, 3' end. | 0.479 |
| NM\_144866 | Mus musculus eukaryotic translation termination factor 1 (Etf1), mRNA | 0.479 |
| NM\_026033 | Mus musculus ocular development associated gene (Odag), mRNA | 0.478 |
| NM\_145926 | Mus musculus mannoside acetylglucosaminyltransferase 4, isoenzyme B (Mgat4b), mRNA | 0.478 |
| AK047398 | Mus musculus 10 days neonate cerebellum cDNA, RIKEN full-length enriched library, clone:B930055M19 product:unknown EST, full insert sequence. | 0.477 |
| AK008240 | Mus musculus adult male small intestine cDNA, RIKEN full-length enriched library, clone:2010013O18 product:SMALL NUCLEAR RIBONUCLEOPROTEIN F (SNRNP-F) (SM PROTEIN F) (SM-F) (SMF) homolog [Homo sapiens], full insert sequence. | 0.477 |
| NM\_172308 | Mus musculus formyltetrahydrofolate synthetase domain containing 1 (Fthfsdc1), mRNA | 0.476 |
| NM\_145462 | Mus musculus DNA segment, Chr 14, ERATO Doi 500, expressed (D14Ertd500e), mRNA | 0.476 |
| BC049166 | Mus musculus nucleolar protein 8, mRNA (cDNA clone IMAGE:6486468), partial cds. | 0.475 |
| NM\_133939 | Mus musculus LSM8 homolog, U6 small nuclear RNA associated (S. cerevisiae) (Lsm8), mRNA | 0.475 |
| NM\_007616 | Mus musculus caveolin, caveolae protein (Cav), mRNA | 0.475 |
| NM\_009840 | Mus musculus chaperonin subunit 8 (theta) (Cct8), mRNA | 0.474 |
| NM\_013896 | Mus musculus translocase of inner mitochondrial membrane 10 homolog (yeast) (Timm10), mRNA | 0.474 |
| BC059873 | Mus musculus 3-hydroxy-3-methylglutaryl-Coenzyme A reductase, mRNA (cDNA clone IMAGE:6810312), partial cds. | 0.474 |
| NM\_031257 | Mus musculus pleckstrin homology domain-containing, family A (phosphoinositide binding specific) member 2 (Plekha2), mRNA | 0.474 |
| AK087556 | Mus musculus 0 day neonate eyeball cDNA, RIKEN full-length enriched library, clone:E130319M10 product:ubiquitin-like 1 (sentrin) activating enzyme E1A, full insert sequence. | 0.474 |
| NM\_011284 | Mus musculus replication protein A2 (Rpa2), mRNA | 0.473 |
| NM\_015751 | Mus musculus ATP-binding cassette, sub-family E (OABP), member 1 (Abce1), mRNA | 0.473 |
| BU609786 | UI-M-DJ2-bvz-j-24-0-UI.r1 NIH\_BMAP\_DJ2 Mus musculus cDNA clone UI-M-DJ2-bvz-j-24-0-UI 5', mRNA sequence | 0.473 |
| AK017715 | Mus musculus 8 days embryo whole body cDNA, RIKEN full-length enriched library, clone:5730490E06 product:hypothetical RNA-binding domain, RBD structure containing protein, full insert sequence. | 0.473 |
| NM\_025695 | Mus musculus SMC6 structural maintenance of chromosomes 6-like 1 (yeast) (Smc6l1), mRNA | 0.473 |
| NM\_021336 | Mus musculus small nuclear ribonucleoprotein polypeptide A' (Snrpa1), mRNA | 0.473 |
| NAP046391-1 | Unknown | 0.473 |
| NM\_016710 | Mus musculus nucleosome binding protein 1 (Nsbp1), mRNA | 0.472 |
| BI100969 | BI100969 602886295F1 NCI\_CGAP\_Kid14 Mus musculus cDNA clone IMAGE:5041727 5', mRNA sequence | 0.472 |
| ENSMUST00000030569 | Unknown | 0.472 |
| BC055031 | Mus musculus synaptic nuclear envelope 2, mRNA (cDNA clone IMAGE:5042878), partial cds. | 0.472 |
| XM\_194845 | Mus musculus similar to Gle1l protein (LOC271022), mRNA | 0.472 |
| NM\_010066 | Mus musculus DNA methyltransferase (cytosine-5) 1 (Dnmt1), mRNA | 0.472 |
| NAP028759-1 | Unknown | 0.472 |
| NM\_013686 | Mus musculus t-complex protein 1 (Tcp1), mRNA | 0.471 |
| AK015913 | Mus musculus adult male testis cDNA, RIKEN full-length enriched library, clone:4930527E24 product:weakly similar to XMR PROTEIN [Mus musculus], full insert sequence. | 0.471 |
| TC1084768 | BC031435 Chpt1 protein {Mus musculus}, partial (18%) | 0.471 |
| NM\_178846 | Mus musculus EST C77032 (C77032), transcript variant 2, mRNA | 0.471 |
| AK013180 | Mus musculus 10, 11 days embryo whole body cDNA, RIKEN full-length enriched library, clone:2810428H03 product:transcription factor Dp 1, full insert sequence. | 0.471 |
| BC049929 | Mus musculus RIKEN cDNA 4732462I11 gene, mRNA (cDNA clone IMAGE:5309846), partial cds. | 0.471 |
| NM\_177669 | Mus musculus RIKEN cDNA A630098G03 gene (A630098G03Rik), mRNA | 0.471 |
| NM\_008300 | Mus musculus heat shock protein 4 (Hspa4), mRNA | 0.470 |
| NAP027343-1 | Unknown | 0.470 |
| NM\_182768 | Mus musculus expressed sequence AA407526 (AA407526), transcript variant 2, mRNA | 0.470 |
| NM\_178636 | Mus musculus RIKEN cDNA 4833446K15 gene (4833446K15Rik), mRNA | 0.470 |
| NM\_010474 | Mus musculus heparan sulfate (glucosamine) 3-O-sulfotransferase 1 (Hs3st1), mRNA | 0.469 |
| AK076403 | Mus musculus 10 days neonate skin cDNA, RIKEN full-length enriched library, clone:4732496K05 product:EUKARYOTIC TRANSLATION INITIATION FACTOR 5 (EIF-5) homolog [Rattus norvegicus], full insert sequence. | 0.469 |
| NM\_011614 | Mus musculus tumor necrosis factor (ligand) superfamily, member 12 (Tnfsf12), mRNA | 0.469 |
| AK084087 | Mus musculus 12 days embryo spinal ganglion cDNA, RIKEN full-length enriched library, clone:D130085P08 product:unknown EST, full insert sequence. | 0.469 |
| XM\_127501 | Mus musculus RIKEN cDNA G630013P12 gene (G630013P12Rik), mRNA | 0.469 |
| NM\_008799 | Mus musculus programmed cell death 2 (Pdcd2), mRNA | 0.468 |
| ENSMUST00000059869 | BM168F16.1 (NOVEL PROTEIN SIMILAR TO HIGH-MOBILITY GROUP BOX 1 (HMGB1)). [Source:SPTREMBL;Acc:Q80YZ1] | 0.468 |
| NM\_007971 | Mus musculus enhancer of zeste homolog 2 (Drosophila) (Ezh2), mRNA | 0.468 |
| NAP061435-1 | Unknown | 0.467 |
| AK017690 | Mus musculus 8 days embryo whole body cDNA, RIKEN full-length enriched library, clone:5730470C14 product:unknown EST, full insert sequence. | 0.467 |
| AK012772 | Mus musculus 10, 11 days embryo whole body cDNA, RIKEN full-length enriched library, clone:2810021C03 product:hypothetical RNA-binding region RNP-1 (RNA recognition motif) containing protein, full insert sequence. | 0.467 |
| AK078033 | Mus musculus 11 days embryo head cDNA, RIKEN full-length enriched library, clone:6230425C22 product:weakly similar to HYPOTHETICAL WD-REPEAT PROTEIN CGI-48 [Homo sapiens], full insert sequence. | 0.467 |
| TC1084189 | Unknown | 0.467 |
| NM\_010829 | Mus musculus mutS homolog 3 (E. coli) (Msh3), mRNA | 0.467 |
| NM\_029775 | Mus musculus RIKEN cDNA 4833420K19 gene (4833420K19Rik), mRNA | 0.467 |
| XM\_130877 | Mus musculus guanine monphosphate synthetase (Gmps), mRNA | 0.467 |
| NM\_009538 | Mus musculus pleiomorphic adenoma gene-like 1 (Plagl1), mRNA | 0.466 |
| NM\_013830 | Mus musculus PRP4 pre-mRNA processing factor 4 homolog B (yeast) (Prpf4b), mRNA | 0.466 |
| NM\_027642 | Mus musculus PHD finger protein 6 (Phf6), mRNA | 0.466 |
| NM\_144835 | Mus musculus cDNA sequence BC019693 (BC019693), mRNA | 0.466 |
| 4432406C08 | hypothetical Winged helix DNA-binding domain structure containing protein | 0.466 |
| NM\_007426 | Mus musculus angiopoietin 2 (Agpt2), mRNA | 0.465 |
| NM\_134081 | Mus musculus DnaJ (Hsp40) homolog, subfamily C, member 9 (Dnajc9), mRNA | 0.465 |
| NM\_145367 | Mus musculus thioredoxin domain containing 5 (Txndc5), mRNA | 0.465 |
| NM\_010941 | Mus musculus NAD(P) dependent steroid dehydrogenase-like (Nsdhl), mRNA | 0.465 |
| NM\_013787 | Mus musculus S-phase kinase-associated protein 2 (p45) (Skp2), mRNA | 0.465 |
| BC051230 | Mus musculus, clone IMAGE:3472087, mRNA. | 0.464 |
| NM\_026195 | Mus musculus 5-aminoimidazole-4-carboxamide ribonucleotide formyltransferase/IMP cyclohydrolase (Atic), mRNA | 0.464 |
| BC038663 | Mus musculus RIKEN cDNA 1110018J23 gene, mRNA (cDNA clone IMAGE:5366157), partial cds. | 0.464 |
| NM\_010325 | Mus musculus glutamate oxaloacetate transaminase 2, mitochondrial (Got2), mRNA | 0.463 |
| NM\_029804 | Mus musculus heterogeneous nuclear ribonucleoprotein M (Hnrpm), mRNA | 0.463 |
| NM\_025801 | Mus musculus phosphogluconate dehydrogenase (Pgd), mRNA | 0.463 |
| NM\_019673 | Mus musculus actin-like 6A (Actl6a), mRNA | 0.463 |
| NM\_134084 | Mus musculus peptidylprolyl isomerase F (cyclophilin F) (Ppif), mRNA | 0.462 |
| NM\_026053 | Mus musculus gem (nuclear organelle) associated protein 6 (Gemin6), mRNA | 0.462 |
| NM\_009367 | Mus musculus transforming growth factor, beta 2 (Tgfb2), mRNA | 0.462 |
| NM\_010247 | Mus musculus thyroid autoantigen (G22p1), mRNA | 0.462 |
| AK129078 | Mus musculus mRNA for mKIAA0179 protein. | 0.462 |
| NM\_134010 | Mus musculus nucleoporin 107 (Nup107), mRNA | 0.461 |
| NM\_181412 | Mus musculus hypothetical protein LOC223773 (LOC223773), mRNA | 0.461 |
| AK089989 | Mus musculus bladder RCB-0544 MBT-2 cDNA, RIKEN full-length enriched library, clone:G430046G23 product:hypothetical protein, full insert sequence | 0.461 |
| NM\_029879 | Mus musculus DNA segment, Chr 13, Brigham & Women's Genetics 1146 expressed (D13Bwg1146e), mRNA | 0.460 |
| NM\_025547 | Mus musculus RIKEN cDNA 2410017I18 gene (2410017I18Rik), mRNA | 0.460 |
| NM\_009150 | Mus musculus selenium binding protein 1 (Selenbp1), mRNA | 0.460 |
| NM\_008893 | Mus musculus polymerase (DNA directed), alpha 2 (Pola2), mRNA | 0.460 |
| NM\_026282 | Mus musculus RIKEN cDNA 2410030K01 gene (2410030K01Rik), mRNA | 0.460 |
| NM\_008567 | Mus musculus minichromosome maintenance deficient 6 (MIS5 homolog, S. pombe) (S. cerevisiae) (Mcm6), mRNA | 0.459 |
| AK004831 | Mus musculus adult male lung cDNA, RIKEN full-length enriched library, clone:1210001E11 product:SPLICING FACTOR, ARGININE/SERINE-RICH 6 homolog [Homo sapiens], full insert sequence. | 0.459 |
| NM\_019698 | Mus musculus pyrroline-5-carboxylate synthetase (glutamate gamma-semialdehyde synthetase) (Pycs), mRNA | 0.459 |
| NM\_021524 | Mus musculus pre-B-cell colony-enhancing factor 1 (Pbef1), mRNA | 0.459 |
| AK017490 | Mus musculus 8 days embryo whole body cDNA, RIKEN full-length enriched library, clone:5730405D16 product:similar to U3 SMALL NUCLEOLAR RIBONUCLEOPROTEIN PROTEIN MPP10 (M PHASE PHOSPHOPROTEIN 10) [Homo sapiens], full insert sequence. | 0.458 |
| TC1059929 | AF162707 protein tyrosine phosphatase-like protein PTPLA {Ovis aries}, partial (25%) | 0.458 |
| NM\_021505 | Mus musculus anaphase-promoting complex subunit 5 (Anapc5), mRNA | 0.458 |
| AK049629 | Mus musculus 12 days embryo spinal cord cDNA, RIKEN full-length enriched library, clone:C530008M17 product:hypothetical protein, full insert sequence. | 0.458 |
| NM\_175265 | Mus musculus RIKEN cDNA 6720463M24 gene (6720463M24Rik), mRNA | 0.458 |
| NM\_145131 | Mus musculus pitrilysin metalloprotease 1 (Pitrm1), mRNA | 0.458 |
| ENSMUST00000039549 | mi69c05.y1 Soares mouse embryo NbME13.5 14.5 Mus musculus cDNA clone IMAGE:468776 5' similar to gb:M99167 Mouse rearranged RNA binding protein (MOUSE);. [AI893526] | 0.458 |
| NM\_172746 | Mus musculus expressed sequence C86302 (C86302), mRNA | 0.458 |
| XM\_133186 | Mus musculus similar to hypothetical protein FLJ14486 (LOC232879), mRNA | 0.458 |
| BC053106 | Mus musculus actin-related protein 3-beta, mRNA (cDNA clone IMAGE:5685374), partial cds. | 0.457 |
| NM\_012012 | Mus musculus exonuclease 1 (Exo1), mRNA | 0.457 |
| NM\_026067 | Mus musculus RIKEN cDNA 3110010F15 gene (3110010F15Rik), mRNA | 0.457 |
| BC057602 | Mus musculus cDNA clone MGC:67258 IMAGE:6413648, complete cds. | 0.457 |
| NM\_019814 | Mus musculus hypoxia induced gene 1 (Hig1), mRNA | 0.456 |
| BB755555 | BB755555 RIKEN full-length enriched, melanocyte Mus musculus cDNA clone G270062I20 3', mRNA sequence | 0.456 |
| NM\_011605 | Mus musculus thymopoietin (Tmpo), mRNA | 0.456 |
| NM\_177325 | Mus musculus expressed sequence AW550801 (AW550801), mRNA | 0.456 |
| NM\_175665 | Mus musculus histone 1, H2bk (Hist1h2bk), mRNA | 0.456 |
| NM\_133930 | Mus musculus cysteine-rich with EGF-like domains 1 (Creld1), mRNA | 0.456 |
| NM\_177356 | Mus musculus lysosomal-associated membrane protein 3 (Lamp3), mRNA | 0.456 |
| AK129097 | Mus musculus mRNA for mKIAA0239 protein. | 0.456 |
| AK010725 | Mus musculus ES cells cDNA, RIKEN full-length enriched library, clone:2410076I21 product:hypothetical protein, full insert sequence. | 0.456 |
| NAP071064-1 | Unknown | 0.455 |
| NM\_153098 | Mus musculus CD109 antigen (Cd109), mRNA | 0.455 |
| NM\_026550 | Mus musculus PAK1 interacting protein 1 (Pak1ip1), mRNA | 0.455 |
| NM\_172146 | Mus musculus phosphoribosyl pyrophosphate amidotransferase (Ppat), mRNA | 0.455 |
| AK014396 | Mus musculus adult male brain cDNA, RIKEN full-length enriched library, clone:3632411M23 product:weakly similar to COPINE VI (NEURONAL-COPINE) (N-COPINE) [Homo sapiens], full insert sequence. | 0.455 |
| AK011489 | Mus musculus 10 days embryo whole body cDNA, RIKEN full-length enriched library, clone:2610020K06 product:replication factor C (activator 1) 5 (36.5 kDa), full insert sequence. | 0.455 |
| AK083643 | Mus musculus 9 days embryo whole body cDNA, RIKEN full-length enriched library, clone:D030058M09 product:hypothetical P-loop containing nucleotide triphosphate hydrolases structure containing protein, full insert sequence. | 0.454 |
| NM\_025369 | Mus musculus mitochondrial ribosomal protein S36 (Mrps36), mRNA | 0.454 |
| AK034156 | Mus musculus adult male diencephalon cDNA, RIKEN full-length enriched library, clone:9330160C06 product:similar to SMALL GTP-BINDING PROTEIN ASSOCIATED PROTEIN (FRAGMENT) [Mus musculus], full insert sequence | 0.454 |
| NM\_028274 | Mus musculus exosome component 6 (Exosc6), mRNA | 0.453 |
| NM\_199056 | Mus musculus RIKEN cDNA 1810043M15 gene (1810043M15Rik), mRNA | 0.453 |
| NM\_173047 | Mus musculus carbonyl reductase 3 (Cbr3), mRNA | 0.453 |
| NM\_028712 | Mus musculus RAP2B, member of RAS oncogene family (Rap2b), mRNA | 0.453 |
| NM\_010722 | Mus musculus lamin B2 (Lmnb2), mRNA | 0.452 |
| NM\_016965 | Mus musculus NCK-associated protein 1 (Nckap1), mRNA | 0.452 |
| NAP029945-1 | Unknown | 0.452 |
| ENSMUST00000045570 | Unknown | 0.452 |
| NM\_011499 | Mus musculus serine/threonine kinase receptor associated protein (Strap), mRNA | 0.452 |
| NM\_011160 | Mus musculus protein kinase, cGMP-dependent, type I (Prkg1), mRNA | 0.451 |
| AK017170 | Mus musculus 11 days pregnant adult female ovary and uterus cDNA, RIKEN full-length enriched library, clone:5033414A21 product:inferred: human CLASP-4 {Homo sapiens}, full insert sequence | 0.451 |
| NM\_026613 | Mus musculus RIKEN cDNA 2810027O19 gene (2810027O19Rik), mRNA | 0.451 |
| NM\_146165 | Mus musculus JTV1 gene (Jtv1), mRNA | 0.451 |
| NM\_008651 | Mus musculus myeloblastosis oncogene-like 1 (Mybl1), mRNA | 0.451 |
| AB093279 | Mus musculus mRNA for mKIAA1011 protein. | 0.450 |
| XM\_141679 | Mus musculus similar to Testis derived transcript (LOC236749), mRNA | 0.450 |
| NM\_021385 | Mus musculus RAD18 homolog (S. cerevisiae) (Rad18), mRNA | 0.450 |
| NM\_028185 | Mus musculus U7 snRNP-specific Sm-like protein LSM11 (Lsm11), mRNA | 0.450 |
| NM\_007839 | Mus musculus DEAH (Asp-Glu-Ala-His) box polypeptide 15 (Dhx15), mRNA | 0.450 |
| NM\_144731 | Mus musculus UDP-N-acetyl-alpha-D-galactosamine: polypeptide N-acetylgalactosaminyltransferase 7 (Galnt7), mRNA | 0.450 |
| BC057001 | Mus musculus outer dense fiber of sperm tails 2, mRNA (cDNA clone MGC:66991 IMAGE:6827260), complete cds. | 0.449 |
| AF223568 | Mus musculus frataxin pseudogene, complete sequence. | 0.449 |
| NM\_025824 | Mus musculus basic leucine zipper and W2 domains 1 (Bzw1), mRNA | 0.449 |
| NM\_020004 | Mus musculus GCN5 general control of amino acid synthesis-like 2 (yeast) (Gcn5l2), mRNA | 0.449 |
| NM\_019487 | Mus musculus heme binding protein 2 (Hebp2), mRNA | 0.449 |
| XM\_129213 | Mus musculus similar to eukaryotic translation initiation factor 4A1; initiation factor eIF-4A long form (LOC208110), mRNA | 0.449 |
| BC032167 | Mus musculus phosphoglycerate kinase 1, mRNA (cDNA clone IMAGE:5354908) | 0.448 |
| ENSMUST00000048525 | Unknown | 0.448 |
| AK012292 | Mus musculus 11 days embryo whole body cDNA, RIKEN full-length enriched library, clone:2700026H11 product:ECTONUCLEOSIDE TRIPHOSPHATE DIPHOSPHOHYDROLASE 6 (EC 3.6.1.6) (NTPDASE6) (CD39 ANTIGEN-LIKE 2) homolog [Rattus norvegicus], full insert sequence. [ | 0.448 |
| NM\_028245 | Mus musculus zinc finger protein 131 (Zfp131), mRNA | 0.448 |
| NM\_181582 | Mus musculus eukaryotic translation initiation factor 5A (Eif5a), mRNA | 0.448 |
| NM\_175507 | Mus musculus transmembrane protein 20 (Tmem20), mRNA | 0.448 |
| NAP052136-1 | Unknown | 0.448 |
| AI323028 | AI323028 mj26c04.y1 Soares mouse embryo NbME13.5 14.5 Mus musculus cDNA clone IMAGE:477222 5' similar to gb:X02308 THYMIDYLATE SYNTHASE (HUMAN);, mRNA sequence | 0.447 |
| NM\_173757 | Mus musculus mitochondrial ribosomal protein S27 (Mrps27), mRNA | 0.447 |
| AK028815 | Mus musculus 10 days neonate skin cDNA, RIKEN full-length enriched library, clone:4732458H05 product:cytochrome P450, 51, full insert sequence. | 0.447 |
| NM\_146106 | Mus musculus lysophospholipase-like 1 (Lyplal1), mRNA | 0.447 |
| AK050381 | Mus musculus adult male liver tumor cDNA, RIKEN full-length enriched library, clone:C730042B03 product:weakly similar to TROPHININ-ASSOCIATED PROTEIN (TASTIN) (TROPHININ-ASSISTING PROTEIN) [Homo sapiens], full insert sequence. | 0.447 |
| NM\_027139 | Mus musculus TAF9 RNA polymerase II, TATA box binding protein (TBP)-associated factor (Taf9), mRNA | 0.446 |
| BC010304 | Mus musculus cDNA sequence BC010304, mRNA (cDNA clone IMAGE:3597827), partial cds. | 0.446 |
| NM\_144553 | Mus musculus discs, large homolog 7 (Drosophila) (Dlg7), mRNA | 0.446 |
| NM\_028230 | Mus musculus serine hydroxymethyl transferase 2 (mitochondrial) (Shmt2), mRNA | 0.446 |
| NM\_175692 | Mus musculus RIKEN cDNA A930034L06 gene (A930034L06Rik), mRNA | 0.446 |
| NM\_134058 | Mus musculus pelota homolog (Drosophila) (Pelo), mRNA | 0.446 |
| NM\_207255 | Mus musculus RIKEN cDNA C530030I18 gene (C530030I18Rik), mRNA | 0.445 |
| NM\_019941 | Mus musculus zinc finger protein 235 (Zfp235), mRNA | 0.445 |
| NM\_146006 | Mus musculus lanosterol synthase (Lss), mRNA | 0.445 |
| TC966009 | A8A1\_MOUSE Potential phospholipid-transporting ATPase IA(Chromaffin granule ATPase II). [Mouse] {Mus musculus}, complete | 0.445 |
| NM\_153199 | Mus musculus ATPase family, AAA domain containing 2 (Atad2), mRNA | 0.445 |
| NM\_173867 | Mus musculus RIKEN cDNA 2610510H01 gene (2610510H01Rik), mRNA | 0.444 |
| NM\_033607 | Mus musculus ubiquitin carboxyl-terminal esterase L4 (Uchl4), mRNA | 0.444 |
| NM\_023119 | Mus musculus enolase 1, alpha non-neuron (Eno1), mRNA | 0.444 |
| NM\_010298 | Mus musculus glycine receptor, beta subunit (Glrb), mRNA | 0.444 |
| NM\_025928 | Mus musculus polyamine-modulated factor 1 (Pmf1), mRNA | 0.443 |
| NM\_011830 | Mus musculus inosine 5'-phosphate dehydrogenase 2 (Impdh2), mRNA | 0.443 |
| NM\_172552 | Mus musculus thymine DNA glycosylase (Tdg), mRNA | 0.443 |
| NM\_181517 | Mus musculus importin 7 (Ipo7), mRNA | 0.443 |
| NM\_053163 | Mus musculus mitochondrial ribosomal protein L36 (Mrpl36), mRNA | 0.443 |
| NM\_008031 | Mus musculus fragile X mental retardation syndrome 1 homolog (Fmr1), mRNA | 0.443 |
| NM\_011252 | Mus musculus RNA binding motif protein, X chromosome (Rbmx), mRNA | 0.442 |
| NM\_199032 | Mus musculus cDNA sequence BC062951 (BC062951), mRNA | 0.442 |
| NM\_180678 | Mus musculus glycyl-tRNA synthetase (Gars), mRNA | 0.441 |
| NM\_028487 | Mus musculus RIKEN cDNA 1700034P14 gene (1700034P14Rik), mRNA | 0.441 |
| NAP104001-1 | Unknown | 0.441 |
| NM\_028106 | Mus musculus RIKEN cDNA 2610005H11 gene (2610005H11Rik), mRNA | 0.441 |
| AK129465 | Mus musculus mRNA for mKIAA1924 protein. | 0.441 |
| NM\_007999 | Mus musculus flap structure specific endonuclease 1 (Fen1), mRNA | 0.440 |
| NM\_021303 | Mus musculus cDNA sequence AF155546 (AF155546), mRNA | 0.440 |
| NM\_134163 | Mus musculus muscleblind-like 3 (Drosophila) (Mbnl3), mRNA | 0.440 |
| NM\_021383 | Mus musculus rcd1 (required for cell differentiation) homolog 1 (S. pombe) (Rqcd1), mRNA | 0.439 |
| BC040784 | Mus musculus cDNA clone MGC:49148 IMAGE:4976926, complete cds. | 0.439 |
| NM\_146041 | Mus musculus GDP-mannose 4, 6-dehydratase (Gmds), mRNA | 0.439 |
| NM\_024448 | Mus musculus RAB12, member RAS oncogene family (Rab12), mRNA | 0.439 |
| AK010453 | Mus musculus ES cells cDNA, RIKEN full-length enriched library, clone:2410008J01 product:pyrimidine synthesis protein CAD (fragment) homolog [Homo sapiens], full insert sequence. | 0.439 |
| AK013308 | Mus musculus 10, 11 days embryo whole body cDNA, RIKEN full-length enriched library, clone:2810449C10 product:unknown EST, full insert sequence. | 0.438 |
| NM\_026785 | Mus musculus ubiquitin-conjugating enzyme E2C (Ube2c), mRNA | 0.438 |
| NM\_007988 | Mus musculus fatty acid synthase (Fasn), mRNA | 0.438 |
| NM\_145455 | Mus musculus basic transcription factor 3 (Btf3), mRNA | 0.438 |
| NM\_016795 | Mus musculus serine/arginine-rich protein specific kinase 1 (Srpk1), mRNA | 0.438 |
| NM\_025279 | Mus musculus heterogeneous nuclear ribonucleoprotein K (Hnrpk), mRNA | 0.437 |
| NM\_025903 | Mus musculus interferon-related developmental regulator 2 (Ifrd2), mRNA | 0.437 |
| NM\_021510 | Mus musculus heterogeneous nuclear ribonucleoprotein H1 (Hnrph1), mRNA | 0.437 |
| NM\_009737 | Mus musculus branched chain aminotransferase 2, mitochondrial (Bcat2), mRNA | 0.436 |
| NM\_022011 | Mus musculus general transcription factor II H, polypeptide 2 (Gtf2h2), mRNA | 0.436 |
| NM\_010576 | Mus musculus integrin alpha 4 (Itga4), mRNA | 0.436 |
| NM\_026483 | Mus musculus M-phase phosphoprotein 10 (U3 small nucleolar ribonucleoprotein) (Mphosph10), mRNA | 0.436 |
| NM\_009707 | Mus musculus Rho GTPase activating protein 6 (Arhgap6), mRNA | 0.435 |
| NM\_011690 | Mus musculus valyl-tRNA synthetase 2 (Vars2), mRNA | 0.435 |
| NM\_008828 | Mus musculus phosphoglycerate kinase 1 (Pgk1), mRNA | 0.434 |
| NM\_019551 | Mus musculus Traf and Tnf receptor associated protein (Ttrap), mRNA | 0.434 |
| L06234 | Mus musculus dihydropyridine sensitive skeletal muscle calcium channel mRNA, complete cds. | 0.434 |
| NM\_025936 | Mus musculus arginyl-tRNA synthetase (Rars), mRNA | 0.433 |
| AK050154 | Mus musculus adult male liver tumor cDNA, RIKEN full-length enriched library, clone:C730023J07 product:Leucine-rich repeat/Leucine-rich repeat, containing protein, full insert sequence. | 0.433 |
| NM\_008157 | Mus musculus G protein-coupled receptor 19 (Gpr19), mRNA | 0.433 |
| AK018076 | Mus musculus 11 days embryo head cDNA, RIKEN full-length enriched library, clone:6230400I06 product:unclassifiable, full insert sequence. | 0.433 |
| NM\_198090 | Mus musculus RIKEN cDNA 2610510D13 gene (2610510D13Rik), transcript variant a, mRNA | 0.432 |
| AK083676 | Mus musculus 9 days embryo whole body cDNA, RIKEN full-length enriched library, clone:D030064D06 product:unknown EST, full insert sequence | 0.432 |
| NM\_146144 | Mus musculus ubiquitin specific protease 1 (Usp1), mRNA | 0.432 |
| NM\_025952 | Mus musculus RIKEN cDNA 2610529C04 gene (2610529C04Rik), mRNA | 0.431 |
| NM\_010344 | Mus musculus glutathione reductase 1 (Gsr), mRNA | 0.431 |
| AK034845 | Mus musculus 12 days embryo embryonic body between diaphragm region and neck cDNA, RIKEN full-length enriched library, clone:9430046J23 product:NS1-associated protein 1, full insert sequence. | 0.430 |
| NM\_021272 | Mus musculus fatty acid binding protein 7, brain (Fabp7), mRNA | 0.430 |
| NM\_025558 | Mus musculus RIKEN cDNA 1810044O22 gene (1810044O22Rik), mRNA | 0.430 |
| XM\_136621 | Mus musculus similar to Transcription factor BTF3 (RNA polymerase B transcription factor 3) (LOC227114), mRNA | 0.430 |
| NM\_028744 | Mus musculus phosphatidylinositol 4-kinase type 2 beta (Pi4k2b), mRNA | 0.430 |
| NM\_028244 | Mus musculus RIKEN cDNA 2600005C20 gene (2600005C20Rik), mRNA | 0.429 |
| TC984858 | AF288289 RPT protein {Rattus norvegicus}, complete | 0.429 |
| NM\_172616 | Mus musculus RIKEN cDNA C330027C09 gene (C330027C09Rik), mRNA | 0.428 |
| NM\_019562 | Mus musculus ubiquitin carboxyl-terminal esterase L5 (Uchl5), mRNA | 0.428 |
| BC076625 | Unknown | 0.428 |
| ENSMUST00000042100 | Unknown | 0.428 |
| NM\_177372 | Mus musculus RIKEN cDNA E130315B21 gene (E130315B21Rik), mRNA | 0.427 |
| NM\_026352 | Mus musculus peptidylprolyl isomerase D (cyclophilin D) (Ppid), mRNA | 0.427 |
| U14648 | Mus musculus putative myelin regulatory factor 1 mRNA, partial cds. | 0.427 |
| NM\_013932 | Mus musculus DEAD (Asp-Glu-Ala-Asp) box polypeptide 25 (Ddx25), mRNA | 0.426 |
| NM\_153581 | Mus musculus glycoprotein m6a (Gpm6a), mRNA | 0.426 |
| NM\_013871 | Mus musculus mitogen-activated protein kinase 12 (Mapk12), mRNA | 0.426 |
| U72681 | Mus musculus EF-6 mRNA, partial cds. | 0.426 |
| NM\_009054 | Mus musculus tripartite motif protein 27 (Trim27), mRNA | 0.425 |
| NM\_009361 | Mus musculus transcription factor Dp 1 (Tfdp1), mRNA | 0.425 |
| NM\_026517 | Mus musculus RIKEN cDNA 3110001N18 gene (3110001N18Rik), mRNA | 0.425 |
| NM\_026656 | Mus musculus mucolipin 2 (Mcoln2), mRNA | 0.425 |
| AK076238 | Mus musculus 14 days embryo liver cDNA, RIKEN full-length enriched library, clone:4432409B16 product:weakly similar to PHOSPHORIBOSYLFORMYLGLYCINAMIDINE SYNTHASE (EC 6.3.5.3) (FGAM SYNTHASE) (FGAMS) (FORMYLGLYCINAMIDE RIBOTIDE AMIDOTRANSFERASE) (FGARAT) | 0.425 |
| NM\_053092 | Mus musculus lysyl-tRNA synthetase (Kars), mRNA | 0.424 |
| NM\_011029 | Mus musculus laminin receptor 1 (ribosomal protein SA) (Lamr1), mRNA | 0.424 |
| NM\_028677 | Mus musculus peptidyl prolyl isomerase H (Ppih), mRNA | 0.424 |
| AK011719 | Mus musculus 10 days embryo whole body cDNA, RIKEN full-length enriched library, clone:2610039C10 product:hypothetical protein, full insert sequence. | 0.424 |
| NM\_023603 | Mus musculus splicing factor proline/glutamine rich (polypyrimidine tract binding protein associated) (Sfpq), mRNA | 0.424 |
| NM\_021312 | Mus musculus WD repeat domain 12 (Wdr12), mRNA | 0.424 |
| NM\_009687 | Mus musculus apurinic/apyrimidinic endonuclease 1 (Apex1), mRNA | 0.422 |
| AK048051 | Mus musculus 16 days embryo head cDNA, RIKEN full-length enriched library, clone:C130032B15 product:MICROSOMAL SIGNAL PEPTIDASE 23 KDA SUBUNIT (EC 3.4.-.-) (SPASE 22 KDA SUBUNIT) (SPC22/23) homolog [Canis familiaris], full insert sequence. | 0.422 |
| AK014994 | Mus musculus adult male testis cDNA, RIKEN full-length enriched library, clone:4921532D18 product:hypothetical RNA-binding region RNP-1 (RNA recognition motif) containing protein, full insert sequence. | 0.422 |
| NAP002592-003 | Unknown | 0.422 |
| NM\_145529 | Mus musculus cleavage stimulation factor, 3' pre-RNA, subunit 3 (Cstf3), mRNA | 0.421 |
| XM\_131720 | Mus musculus RIKEN cDNA 2610002D18 gene (2610002D18Rik), mRNA | 0.421 |
| NM\_009282 | Mus musculus stromal antigen 1 (Stag1), mRNA | 0.421 |
| NM\_009278 | Mus musculus Sjogren syndrome antigen B (Ssb), mRNA | 0.421 |
| NM\_026561 | Mus musculus RIKEN cDNA C330027I04 gene (C330027I04Rik), mRNA | 0.420 |
| NM\_021720 | Mus musculus downstream neighbor of SON (Donson), mRNA | 0.420 |
| NM\_008956 | Mus musculus polypyrimidine tract binding protein 1 (Ptbp1), mRNA | 0.420 |
| AK050869 | Mus musculus 9 days embryo whole body cDNA, RIKEN full-length enriched library, clone:D030027O06 product:unknown EST, full insert sequence. | 0.420 |
| NM\_176945 | Mus musculus RIKEN cDNA 6820443O06 gene (6820443O06Rik), mRNA | 0.419 |
| NM\_028355 | Mus musculus RIKEN cDNA 2810475A17 gene (2810475A17Rik), mRNA | 0.419 |
| BC021422 | Mus musculus mRNA similar to hypothetical protein FLJ21657 (cDNA clone MGC:29320 IMAGE:5011629), complete cds | 0.419 |
| NM\_007415 | Mus musculus ADP-ribosyltransferase (NAD+, poly (ADP-ribose) polymerase) 1 (Adprt1), mRNA | 0.419 |
| BC026492 | Mus musculus DEAD (Asp-Glu-Ala-Asp) box polypeptide 46, mRNA (cDNA clone MGC:31579 IMAGE:4505095), complete cds. | 0.419 |
| NM\_011733 | Mus musculus cold shock domain protein A (Csda), mRNA | 0.418 |
| NM\_007658 | Mus musculus cell division cycle 25 homolog A (S. cerevisiae) (Cdc25a), mRNA | 0.418 |
| BC058670 | Mus musculus cDNA clone IMAGE:6410591, partial cds. | 0.417 |
| NM\_013902 | Mus musculus FK506 binding protein 3 (Fkbp3), mRNA | 0.417 |
| NM\_027123 | Mus musculus RIKEN cDNA 2310010B21 gene (2310010B21Rik), mRNA | 0.417 |
| AK008736 | Mus musculus adult male stomach cDNA, RIKEN full-length enriched library, clone:2210016F16 product:hypothetical protein, full insert sequence. | 0.417 |
| NM\_010447 | Mus musculus heterogeneous nuclear ribonucleoprotein A1 (Hnrpa1), mRNA | 0.417 |
| AK030970 | Mus musculus adult male thymus cDNA, RIKEN full-length enriched library, clone:5830472I03 product:ANTISENSE RNA OVERLAPPING MCH PROTEIN [Rattus norvegicus], full insert sequence. | 0.417 |
| NM\_008590 | Mus musculus mesoderm specific transcript (Mest), mRNA | 0.417 |
| NM\_023136 | Mus musculus deoxythymidylate kinase (Dtymk), mRNA | 0.416 |
| NM\_144918 | Mus musculus SET and MYND domain containing 5 (Smyd5), mRNA | 0.416 |
| NM\_029766 | Mus musculus RIKEN cDNA 2810047L02 gene (2810047L02Rik), mRNA | 0.415 |
| 9430025N12 | unknown EST | 0.415 |
| NM\_145938 | Mus musculus ribonuclease P 40kDa subunit (human) (Rpp40), mRNA | 0.414 |
| NM\_010353 | Mus musculus germ cell-specific gene 2 (Gsg2), mRNA | 0.414 |
| AK012596 | Mus musculus 11 days embryo whole body cDNA, RIKEN full-length enriched library, clone:2700091C21 product:similar to PUTATIVE RNA BINDING PROTEIN [Homo sapiens], full insert sequence | 0.414 |
| NM\_027973 | Mus musculus myeloid leukemia factor 1 interacting protein (Mlf1ip), mRNA | 0.414 |
| NM\_176979 | Mus musculus topoisomerase (DNA) II beta binding protein (Topbp1), mRNA | 0.414 |
| NM\_146283 | Mus musculus olfactory receptor 1366 (Olfr1366), mRNA | 0.413 |
| ENSMUST00000024054 | Unknown | 0.413 |
| XM\_196313 | Mus musculus hypothetical LOC269515 (LOC269515), mRNA | 0.413 |
| NM\_010160 | Mus musculus CUG triplet repeat,RNA binding protein 2 (Cugbp2), mRNA | 0.413 |
| NM\_145373 | Mus musculus cDNA sequence BC010462 (BC010462), mRNA | 0.413 |
| NM\_013552 | Mus musculus hyaluronan mediated motility receptor (RHAMM) (Hmmr), mRNA | 0.412 |
| AK047427 | Mus musculus 10 days neonate cerebellum cDNA, RIKEN full-length enriched library, clone:B930060C03 product:hypothetical Bromodomain/ PHD-finger containing protein, full insert sequence. | 0.412 |
| AK047584 | Mus musculus 10 days neonate cerebellum cDNA, RIKEN full-length enriched library, clone:B930094H20 product:unknown EST, full insert sequence. | 0.412 |
| NM\_025359 | Mus musculus transmembrane 4 superfamily member 13 (Tm4sf13), mRNA | 0.412 |
| NM\_007832 | Mus musculus deoxycytidine kinase (Dck), mRNA | 0.410 |
| NM\_024223 | Mus musculus cysteine rich protein 2 (Crip2), mRNA | 0.410 |
| NM\_010439 | Mus musculus high mobility group box 1 (Hmgb1), mRNA | 0.409 |
| NM\_007550 | Mus musculus Bloom syndrome homolog (human) (Blm), mRNA | 0.409 |
| NM\_175390 | Mus musculus RIKEN cDNA 1700049L16 gene (1700049L16Rik), mRNA | 0.409 |
| NM\_138745 | Mus musculus methylenetetrahydrofolate dehydrogenase (NADP+ dependent), methenyltetrahydrofolate cyclohydrolase, formyltetrahydrofolate synthase (Mthfd1), mRNA | 0.409 |
| NM\_022989 | Mus musculus ADP-ribosylation factor-like 6 interacting protein 6 (Arl6ip6), mRNA | 0.408 |
| NM\_176860 | Mus musculus RIKEN cDNA 2810457I06 gene (2810457I06Rik), mRNA | 0.408 |
| NM\_025814 | Mus musculus RIKEN cDNA 1200009K13 gene (1200009K13Rik), mRNA | 0.408 |
| NAP113430-1 | Unknown | 0.408 |
| AK018282 | Mus musculus adult male olfactory brain cDNA, RIKEN full-length enriched library, clone:6430514L14 product:hypothetical protein, full insert sequence. | 0.408 |
| NM\_011119 | Mus musculus proliferation-associated 2G4 (Pa2g4), mRNA | 0.408 |
| NM\_019685 | Mus musculus RuvB-like protein 1 (Ruvbl1), mRNA | 0.408 |
| ENSMUST00000017522 | Unknown | 0.407 |
| NM\_010450 | Mus musculus homeo box A11 (Hoxa11), mRNA | 0.407 |
| NM\_197959 | Mus musculus RIKEN cDNA 3000004C01 gene (3000004C01Rik), mRNA | 0.407 |
| BC004768 | Mus musculus cDNA clone MGC:7054 IMAGE:3156506, complete cds. | 0.407 |
| NM\_009384 | Mus musculus T-cell lymphoma invasion and metastasis 1 (Tiam1), mRNA | 0.407 |
| NM\_031863 | Mus musculus RIKEN cDNA 2610528M18 gene (2610528M18Rik), mRNA | 0.407 |
| BC066834 | Mus musculus microtubule associated serine/threonine kinase-like, mRNA (cDNA clone IMAGE:30110680), partial cds. | 0.407 |
| AK076333 | Mus musculus 10 days neonate skin cDNA, RIKEN full-length enriched library, clone:4732440O11 product:weakly similar to GLUCOSIDASE II [Sus scrofa], full insert sequence. | 0.406 |
| NM\_026929 | Mus musculus RIKEN cDNA 1810008K03 gene (1810008K03Rik), mRNA | 0.406 |
| NM\_028108 | Mus musculus Mak3 homolog (S. cerevisiae) (Mak3), mRNA | 0.406 |
| AK032413 | Mus musculus adult male olfactory brain cDNA, RIKEN full-length enriched library, clone:6430540A02 product:unknown EST, full insert sequence | 0.406 |
| NM\_023117 | Mus musculus cell division cycle 25 homolog B (S. cerevisiae) (Cdc25b), mRNA | 0.405 |
| NM\_181416 | Mus musculus RIKEN cDNA 6530401L14 gene (6530401L14Rik), mRNA | 0.405 |
| TC983367 | Unknown | 0.405 |
| NM\_033075 | Mus musculus DNA segment, Chr 17, human D6S56E 5 (D17H6S56E-5), mRNA | 0.405 |
| NM\_178765 | Mus musculus RIKEN cDNA 5730410E15 gene (5730410E15Rik), mRNA | 0.405 |
| NM\_009612 | Mus musculus activin A receptor, type II-like 1 (Acvrl1), mRNA | 0.403 |
| NAP029963-1 | Unknown | 0.403 |
| NAP030172-1 | Unknown | 0.403 |
| NM\_023565 | Mus musculus chromosome segregation 1-like (S. cerevisiae) (Cse1l), mRNA | 0.403 |
| NM\_011524 | Mus musculus transforming, acidic coiled-coil containing protein 3 (Tacc3), mRNA | 0.402 |
| NM\_023243 | Mus musculus cyclin H (Ccnh), mRNA | 0.402 |
| NM\_134014 | Mus musculus exportin 1, CRM1 homolog (yeast) (Xpo1), mRNA | 0.400 |
| NM\_027406 | Mus musculus formyltetrahydrofolate dehydrogenase (Fthfd), mRNA | 0.400 |
| NM\_174868 | Mus musculus RIKEN cDNA C030011O14 gene (C030011O14Rik), mRNA | 0.400 |
| NM\_024169 | Mus musculus FK506 binding protein 11 (Fkbp11), mRNA | 0.400 |
| NM\_026845 | Mus musculus peptidylprolyl isomerase (cyclophilin)-like 1 (Ppil1), mRNA | 0.400 |
| NM\_010849 | Mus musculus myelocytomatosis oncogene (Myc), mRNA | 0.400 |
| AK002846 | Mus musculus adult male kidney cDNA, RIKEN full-length enriched library, clone:0610039M07 product:PX19 (SBBI12) (PX19-LIKE PROTEIN) homolog [Homo sapiens], full insert sequence. | 0.400 |
| NM\_010329 | Mus musculus glycoprotein 38 (Gp38), mRNA | 0.399 |
| BC016563 | Mus musculus pentatricopeptide repeat domain 2, mRNA (cDNA clone IMAGE:2654214), partial cds. | 0.399 |
| BC042619 | Mus musculus Fanconi anemia, complementation group D2, mRNA (cDNA clone IMAGE:4948653), partial cds. | 0.398 |
| AK028505 | Mus musculus 0 day neonate skin cDNA, RIKEN full-length enriched library, clone:4632409J05 product:RAD18 homolog (S.cerevisiae), full insert sequence. | 0.398 |
| BC019782 | Mus musculus 3-hydroxy-3-methylglutaryl-Coenzyme A reductase, mRNA (cDNA clone IMAGE:4166540), with apparent retained intron. | 0.397 |
| AK005633 | Mus musculus adult male testis cDNA, RIKEN full-length enriched library, clone:1700003F12 product:hypothetical Apoptosis regulator protein, Bcl-2 family BH domain containing protein, full insert sequence. | 0.397 |
| NM\_053221 | Mus musculus vomeronasal 1 receptor, A6 (V1ra6), mRNA | 0.397 |
| XM\_122812 | Mus musculus similar to Heterogeneous nuclear ribonucleoprotein A1 (Helix-destabilizing protein) (Single-strand binding protein) (hnRNP core protein A1) (HDP-1) (Topoisomerase-inhibitor suppressed) (LOC223404), mRNA | 0.397 |
| NM\_007856 | Mus musculus 7-dehydrocholesterol reductase (Dhcr7), mRNA | 0.397 |
| D00812 | Mus musculus mRNA for 30-kDa subunit of replication protein A, complete cds. | 0.396 |
| AK088353 | Mus musculus 2 days neonate thymus thymic cells cDNA, RIKEN full-length enriched library, clone:E430013M20 product:hypothetical Neuronal Sec1, NSec1 structure containing protein, full insert sequence. | 0.396 |
| NM\_025447 | Mus musculus RIKEN cDNA 1500031M22 gene (1500031M22Rik), mRNA | 0.396 |
| NM\_007930 | Mus musculus ectodermal-neural cortex 1 (Enc1), mRNA | 0.396 |
| AK122559 | Mus musculus mRNA for mKIAA1821 protein. | 0.395 |
| NM\_020588 | Mus musculus RIKEN cDNA 1300007B12 gene (1300007B12Rik), mRNA | 0.395 |
| NM\_146083 | Mus musculus splicing factor, arginine/serine-rich 7 (Sfrs7), mRNA | 0.395 |
| NM\_025772 | Mus musculus dystrobrevin binding protein 1 (Dtnbp1), mRNA | 0.395 |
| NM\_013550 | Mus musculus histone 1, H3a (Hist1h3a), mRNA | 0.394 |
| NM\_010058 | Mus musculus dystrophia myotonica-containing WD repeat motif (Dmwd), mRNA | 0.394 |
| NM\_021288 | Mus musculus thymidylate synthase (Tyms), mRNA | 0.393 |
| NM\_009234 | Mus musculus SRY-box containing gene 11 (Sox11), mRNA | 0.393 |
| NM\_009908 | Mus musculus cytidine monophospho-N-acetylneuraminic acid synthetase (Cmas), mRNA | 0.393 |
| AK087493 | Mus musculus 0 day neonate eyeball cDNA, RIKEN full-length enriched library, clone:E130306D19 product:hypothetical Dbl domain (dbl | 0.392 |
| NM\_009584 | Mus musculus zuotin related factor 2 (Zrf2), mRNA | 0.392 |
| AK019528 | Mus musculus 0 day neonate head cDNA, RIKEN full-length enriched library, clone:4833446K15 product:hypothetical protein, full insert sequence. | 0.391 |
| NAP115124-1 | Unknown | 0.391 |
| AK037833 | Mus musculus 16 days neonate thymus cDNA, RIKEN full-length enriched library, clone:A130053G07 product:unknown EST, full insert sequence | 0.390 |
| XM\_111995 | Mus musculus similar to Nol5a protein (LOC195806), mRNA | 0.390 |
| NM\_025797 | Mus musculus cytochrome b-5 (Cyb5), mRNA | 0.389 |
| NM\_008704 | Mus musculus expressed in non-metastatic cells 1, protein (Nme1), mRNA | 0.388 |
| NM\_175749 | Mus musculus nucleoporin 153 (Nup153), mRNA | 0.387 |
| NM\_022724 | Mus musculus suppressor of variegation 3-9 homolog 2 (Drosophila) (Suv39h2), mRNA | 0.386 |
| NM\_178609 | Mus musculus E2F transcription factor 7 (E2f7), mRNA | 0.385 |
| AY262012 | Mus musculus retinal pigment mRNA, partial cds. | 0.384 |
| NM\_178269 | Mus musculus RIKEN cDNA 2610019I03 gene (2610019I03Rik), transcript variant 2, mRNA | 0.384 |
| NM\_008047 | Mus musculus follistatin-like 1 (Fstl1), mRNA | 0.384 |
| NM\_022563 | Mus musculus discoidin domain receptor family, member 2 (Ddr2), mRNA | 0.383 |
| NM\_008652 | Mus musculus myeloblastosis oncogene-like 2 (Mybl2), mRNA | 0.383 |
| NM\_021556 | Mus musculus mitochondrial ribosomal protein S30 (Mrps30), mRNA | 0.382 |
| NM\_009445 | Mus musculus Ttk protein kinase (Ttk), mRNA | 0.382 |
| NM\_008706 | Mus musculus NAD(P)H dehydrogenase, quinone 1 (Nqo1), mRNA | 0.382 |
| NM\_027000 | Mus musculus GTP binding protein 4 (Gtpbp4), mRNA | 0.381 |
| NM\_153151 | Mus musculus acetyl-Coenzyme A acetyltransferase 3 (Acat3), mRNA | 0.381 |
| NM\_009770 | Mus musculus B-cell translocation gene 3 (Btg3), mRNA | 0.381 |
| AK086756 | Mus musculus 15 days embryo head cDNA, RIKEN full-length enriched library, clone:D930049I16 product:unknown EST, full insert sequence | 0.381 |
| NM\_019976 | Mus musculus RIKEN cDNA 5430413I02 gene (5430413I02Rik), mRNA | 0.380 |
| ENSMUST00000022903 | Unknown | 0.380 |
| AK017992 | Mus musculus adult male thymus cDNA, RIKEN full-length enriched library, clone:5830445G22 product:IL-5 PROMOTER REII-REGION-BINDING PROTEIN homolog [Homo sapiens], full insert sequence. | 0.380 |
| XM\_137113 | Mus musculus similar to Heterogeneous nuclear ribonucleoproteins A2/B1 (hnRNP A2 / hnRNP B1) (LOC211870), mRNA | 0.379 |
| AK052877 | Mus musculus 16 days neonate heart cDNA, RIKEN full-length enriched library, clone:D830017E01 product:protein kinase, cGMP-dependent, type I, full insert sequence | 0.379 |
| XM\_356348 | Mus musculus ATP-binding cassette, sub-family B (MDR | 0.378 |
| NM\_026030 | Mus musculus eukaryotic translation initiation factor 2, subunit 2 (beta) (Eif2s2), mRNA | 0.378 |
| NM\_011561 | Mus musculus thymine DNA glycosylase (Tdg), mRNA | 0.376 |
| NM\_009185 | Mus musculus Tal1 interrupting locus (Sil), mRNA | 0.376 |
| NM\_177733 | Mus musculus E2F transcription factor 2 (E2f2), mRNA | 0.376 |
| NM\_009028 | Mus musculus RAS-like, family 2, locus 9 (Rasl2-9), mRNA | 0.376 |
| NM\_008894 | Mus musculus polymerase (DNA directed), delta 2, regulatory subunit (Pold2), mRNA | 0.375 |
| NM\_173363 | Mus musculus eukaryotic translation initiation factor 5 (Eif5), mRNA | 0.375 |
| NM\_029665 | Mus musculus importin 11 (Ipo11), mRNA | 0.374 |
| NM\_023871 | Mus musculus SET translocation (Set), mRNA | 0.373 |
| NM\_025951 | Mus musculus phosphatidylinositol 4-kinase type 2 beta (Pi4k2b), mRNA | 0.373 |
| AK083909 | Mus musculus 12 days embryo spinal ganglion cDNA, RIKEN full-length enriched library, clone:D130058J16 product:hypothetical protein, full insert sequence | 0.372 |
| NAP054818-1 | Unknown | 0.372 |
| NM\_027435 | Mus musculus ATPase family, AAA domain containing 2 (Atad2), mRNA | 0.371 |
| AK032696 | Mus musculus 12 days embryo male wolffian duct includes surrounding region cDNA, RIKEN full-length enriched library, clone:6720402K17 product:hypothetical protein, full insert sequence. | 0.371 |
| Z37110 | M.musculus mRNA for cyclin G. | 0.370 |
| NM\_011514 | Mus musculus suppressor of variegation 3-9 homolog 1 (Drosophila) (Suv39h1), mRNA | 0.369 |
| NM\_011448 | Mus musculus SRY-box containing gene 9 (Sox9), mRNA | 0.369 |
| NM\_145480 | Mus musculus replication factor C (activator 1) 4 (Rfc4), mRNA | 0.369 |
| NM\_013935 | Mus musculus protein tyrosine phosphatase-like (proline instead of catalytic arginine), member a (Ptpla), mRNA | 0.368 |
| AK076798 | Mus musculus adult male testis cDNA, RIKEN full-length enriched library, clone:4930470B19 product:unclassifiable, full insert sequence. | 0.368 |
| NM\_175238 | Mus musculus DNA segment, Chr 2, ERATO Doi 145, expressed (D2Ertd145e), mRNA | 0.368 |
| NM\_010892 | Mus musculus NIMA (never in mitosis gene a)-related expressed kinase 2 (Nek2), mRNA | 0.368 |
| NM\_134122 | Mus musculus nurim (nuclear envelope membrane protein) (Nrm), mRNA | 0.368 |
| NM\_024187 | Mus musculus U2 small nuclear ribonucleoprotein auxiliary factor (U2AF) 1 (U2af1), mRNA | 0.368 |
| NM\_146130 | Mus musculus RIKEN cDNA 2610510D13 gene (2610510D13Rik), transcript variant b, mRNA | 0.368 |
| NM\_023284 | Mus musculus cell division cycle associated 1 (Cdca1), mRNA | 0.368 |
| NM\_011536 | Mus musculus T-box 4 (Tbx4), mRNA | 0.367 |
| NM\_133815 | Mus musculus lamin B receptor (Lbr), mRNA | 0.367 |
| NM\_013724 | Mus musculus Nik related kinase (Nrk), mRNA | 0.367 |
| NM\_009471 | Mus musculus uridine monophosphate synthetase (Umps), mRNA | 0.366 |
| AK129332 | Mus musculus mRNA for mKIAA1333 protein. | 0.366 |
| NM\_007684 | Mus musculus centrin 3 (Cetn3), mRNA | 0.366 |
| NM\_139303 | Mus musculus kinesin family member 18A (Kif18a), mRNA | 0.365 |
| NM\_011570 | Mus musculus testis derived transcript (Tes), mRNA | 0.365 |
| BC064129 | Mus musculus RIKEN cDNA 2310007D09 gene, mRNA (cDNA clone MGC:73899 IMAGE:3975722), complete cds. | 0.364 |
| AK012088 | Mus musculus 10 days embryo whole body cDNA, RIKEN full-length enriched library, clone:2610510B12 product:fatty acid Coenzyme A ligase, long chain 3, full insert sequence. | 0.364 |
| NM\_011132 | Mus musculus polymerase (DNA directed), epsilon (Pole), mRNA | 0.364 |
| NM\_198937 | Mus musculus DNA segment, Chr 17, ERATO Doi 441, expressed (D17Ertd441e), mRNA | 0.363 |
| NM\_026515 | Mus musculus RIKEN cDNA 2810417H13 gene (2810417H13Rik), mRNA | 0.362 |
| NM\_011705 | Mus musculus vaccinia related kinase 1 (Vrk1), mRNA | 0.362 |
| NM\_182650 | Mus musculus heterogeneous nuclear ribonucleoprotein A2/B1 (Hnrpa2b1), transcript variant 2, mRNA | 0.360 |
| NM\_009391 | Mus musculus RAN, member RAS oncogene family (Ran), mRNA | 0.360 |
| NM\_133740 | Mus musculus heterogeneous nuclear ribonucleoprotein methyltransferase-like 3 (S. cerevisiae) (Hrmt1l3), mRNA | 0.360 |
| NM\_010219 | Mus musculus FK506 binding protein 4 (Fkbp4), mRNA | 0.359 |
| NM\_175563 | Mus musculus RIKEN cDNA B930067F20 gene (B930067F20Rik), mRNA | 0.359 |
| AK004158 | Mus musculus 18-day embryo whole body cDNA, RIKEN full-length enriched library, clone:1110038H03 product:unknown EST, full insert sequence. | 0.359 |
| NM\_178788 | Mus musculus dCMP deaminase (Dctd), mRNA | 0.358 |
| NM\_026632 | Mus musculus replication protein A3 (Rpa3), mRNA | 0.358 |
| BC036146 | Mus musculus tissue factor pathway inhibitor, mRNA (cDNA clone MGC:37332 IMAGE:4975683), complete cds. | 0.358 |
| NM\_010917 | Mus musculus nidogen 1 (Nid1), mRNA | 0.358 |
| XM\_140742 | Mus musculus RIKEN cDNA 1110018J23 gene (1110018J23Rik), mRNA | 0.357 |
| NM\_145706 | Mus musculus RIKEN cDNA 2610529I12 gene (2610529I12Rik), mRNA | 0.357 |
| NM\_011015 | Mus musculus origin recognition complex, subunit 1-like (S.cereviaiae) (Orc1l), mRNA | 0.356 |
| AK122429 | Mus musculus mRNA for mKIAA1046 protein. | 0.356 |
| NM\_016904 | Mus musculus CDC28 protein kinase 1 (Cks1), mRNA | 0.356 |
| NM\_023554 | Mus musculus nucleolar protein 7 (Nol7), mRNA | 0.356 |
| NM\_172505 | Mus musculus hypothetical protein A730008H23 (A730008H23), mRNA | 0.355 |
| NM\_011990 | Mus musculus solute carrier family 7 (cationic amino acid transporter, y+ system), member 11 (Slc7a11), mRNA | 0.355 |
| NM\_015829 | Mus musculus solute carrier family 25 (mitochondrial carrier, adenine nucleotide translocator), member 13 (Slc25a13), mRNA | 0.355 |
| NM\_023058 | Mus musculus expressed sequence AW209059 (AW209059), mRNA | 0.354 |
| NM\_019455 | Mus musculus prostaglandin D2 synthase 2, hematopoietic (Ptgds2), mRNA | 0.354 |
| BC052742 | Mus musculus shugoshin-like 2 (S. pombe), mRNA (cDNA clone MGC:63378 IMAGE:6833875), complete cds. | 0.354 |
| NM\_017407 | Mus musculus sperm associated antigen 5 (Spag5), mRNA | 0.354 |
| NM\_009104 | Mus musculus ribonucleotide reductase M2 (Rrm2), mRNA | 0.354 |
| TC1061078 | Unknown | 0.354 |
| X95315 | MMUSF1EX1 M.musculus USF1 (non-coding exon 1) | 0.353 |
| NM\_019939 | Mus musculus membrane protein, palmitoylated 6 (MAGUK p55 subfamily member 6) (Mpp6), mRNA | 0.353 |
| NM\_010620 | Mus musculus kinesin-like 7 (Knsl7), mRNA | 0.353 |
| NM\_027178 | Mus musculus peptidylprolyl isomerase (cyclophilin) like 5 (Ppil5), mRNA | 0.353 |
| NM\_172598 | Mus musculus RIKEN cDNA D630024B06 gene (D630024B06Rik), mRNA | 0.352 |
| NM\_025387 | Mus musculus transmembrane protein 14C (Tmem14c), mRNA | 0.351 |
| NM\_133762 | Mus musculus RIKEN cDNA 5830426I05 gene (5830426I05Rik), mRNA | 0.351 |
| AK047775 | Mus musculus adult male corpus striatum cDNA, RIKEN full-length enriched library, clone:C030034L01 product:hypothetical BRCT domain containing protein, full insert sequence. | 0.351 |
| BC038279 | Mus musculus RIKEN cDNA C130052G03 gene, mRNA (cDNA clone IMAGE:4484010), partial cds. | 0.350 |
| AK122475 | Mus musculus mRNA for mKIAA1244 protein. | 0.350 |
| AF016313 | Mus musculus TFPIbeta (TFPIbeta) mRNA, partial cds. | 0.350 |
| NM\_011356 | Mus musculus frizzled-related protein (Frzb), mRNA | 0.350 |
| NM\_145946 | Mus musculus cDNA sequence BC025462 (BC025462), mRNA | 0.350 |
| NM\_009765 | Mus musculus breast cancer 2 (Brca2), mRNA | 0.349 |
| NM\_178309 | Mus musculus BRCA1 interacting protein C-terminal helicase 1 (Brip1), mRNA | 0.348 |
| ENSMUST00000046911 | Unknown | 0.348 |
| NM\_173374 | Mus musculus splicing factor, arginine/serine-rich 1 (ASF/SF2) (Sfrs1), mRNA | 0.348 |
| NM\_011249 | Mus musculus retinoblastoma-like 1 (p107) (Rbl1), mRNA | 0.347 |
| NAP048817-1 | Unknown | 0.347 |
| AK082861 | Mus musculus ES cells cDNA, RIKEN full-length enriched library, clone:C330045B02 product:unknown EST, full insert sequence. | 0.346 |
| NM\_019482 | Mus musculus pannexin 1 (Panx1), mRNA | 0.346 |
| NM\_172015 | Mus musculus isoleucine-tRNA synthetase (Iars), mRNA | 0.345 |
| NM\_010158 | Mus musculus KH domain containing, RNA binding, signal transduction associated 3 (Khdrbs3), mRNA | 0.344 |
| NM\_011276 | Mus musculus ring finger protein 12 (Rnf12), mRNA | 0.342 |
| NM\_198161 | Mus musculus RIKEN cDNA 2700087I09 gene (2700087I09Rik), mRNA | 0.342 |
| NM\_198652 | Mus musculus RIKEN cDNA 6430706D22 gene (6430706D22Rik), mRNA | 0.341 |
| NM\_025508 | Mus musculus guanosine monophosphate reductase (Gmpr), mRNA | 0.341 |
| NM\_009621 | Mus musculus a disintegrin-like and metalloprotease (reprolysin type) with thrombospondin type 1 motif, 1 (Adamts1), mRNA | 0.341 |
| NAP027398-1 | Unknown | 0.341 |
| NM\_013716 | Mus musculus Ras-GTPase-activating protein SH3-domain binding protein (G3bp), mRNA | 0.339 |
| NM\_146208 | Mus musculus cDNA sequence BC034753 (BC034753), mRNA | 0.339 |
| BC022774 | Mus musculus cDNA clone MGC:32192 IMAGE:5006129, complete cds. | 0.338 |
| AB093228 | Mus musculus mRNA for mKIAA0313 protein. | 0.338 |
| AK129093 | Mus musculus mRNA for mKIAA0225 protein. | 0.337 |
| NAP030651-1 | Unknown | 0.337 |
| NM\_012039 | Mus musculus ZW10 homolog (Drosophila), centromere/kinetochore protein (Zw10), mRNA | 0.337 |
| AK050924 | Mus musculus 9 days embryo whole body cDNA, RIKEN full-length enriched library, clone:D030036L15 product:unknown EST, full insert sequence. | 0.337 |
| NM\_175445 | Mus musculus Ras association (RalGDS/AF-6) domain family 2 (Rassf2), mRNA | 0.337 |
| AK012079 | Mus musculus 10 days embryo whole body cDNA, RIKEN full-length enriched library, clone:2610509G12 product:hypothetical AAA-protein (ATPases associated with various cellular activities) containing protein, full insert sequence. | 0.337 |
| BC011230 | Mus musculus RIKEN cDNA 2510015F01 gene, mRNA (cDNA clone MGC:19108 IMAGE:4207917), complete cds. | 0.337 |
| NM\_145354 | Mus musculus DNA segment, Chr 13, Wayne State University 123, expressed (D13Wsu123e), mRNA | 0.337 |
| AF237702 | Mus musculus serine hydroxymethyltransferase mRNA, complete cds. | 0.336 |
| NM\_028131 | Mus musculus RIKEN cDNA 2610510J17 gene (2610510J17Rik), mRNA | 0.336 |
| XM\_136701 | Mus musculus similar to ALY (LOC226955), mRNA | 0.336 |
| BC013089 | Mus musculus E26 avian leukemia oncogene 1, 5' domain, mRNA (cDNA clone IMAGE:3499941), partial cds. | 0.336 |
| NM\_012015 | Mus musculus H2A histone family, member Y (H2afy), mRNA | 0.335 |
| NM\_010790 | Mus musculus maternal embryonic leucine zipper kinase (Melk), mRNA | 0.335 |
| NM\_026312 | Mus musculus RIKEN cDNA 2610029G23 gene (2610029G23Rik), mRNA | 0.334 |
| NM\_011589 | Mus musculus timeless homolog (Drosophila) (Timeless), mRNA | 0.334 |
| NM\_028039 | Mus musculus RIKEN cDNA 2410004I17 gene (2410004I17Rik), mRNA | 0.334 |
| NM\_026023 | Mus musculus DNA segment, Chr 11, ERATO Doi 603, expressed (D11Ertd603e), mRNA | 0.334 |
| NAP102462-1 | Unknown | 0.332 |
| NM\_175660 | Mus musculus histone 1, H2ab (Hist1h2ab), mRNA | 0.332 |
| NM\_145953 | Mus musculus cystathionase (cystathionine gamma-lyase) (Cth), mRNA | 0.332 |
| XM\_144252 | Mus musculus similar to Kinesin-like 6 (LOC242914), mRNA | 0.332 |
| NM\_199196 | Mus musculus DNA segment, Chr 11, ERATO Doi 530, expressed (D11Ertd530e), mRNA | 0.331 |
| AK076422 | Mus musculus 0 day neonate head cDNA, RIKEN full-length enriched library, clone:4831429J16 product:similar to HYPOTHETICAL 31.3 KDA PROTEIN [Homo sapiens], full insert sequence. | 0.331 |
| AF033664 | Mus musculus gene-trap line CT 146 cbp146 (cbp146) mRNA, partial cds. | 0.331 |
| BC005508 | Mus musculus cofactor required for Sp1 transcriptional activation, subunit 3, mRNA (cDNA clone MGC:7510 IMAGE:3491622), complete cds. | 0.327 |
| AK035414 | Mus musculus adult male urinary bladder cDNA, RIKEN full-length enriched library, clone:9530036D08 product:unknown EST, full insert sequence | 0.327 |
| NM\_173391 | Mus musculus tryptophan hydroxylase 2 (Tph2), mRNA | 0.327 |
| NM\_183089 | Mus musculus RIKEN cDNA 2600005O03 gene (2600005O03Rik), mRNA | 0.326 |
| AK034427 | Mus musculus adult male diencephalon cDNA, RIKEN full-length enriched library, clone:9330189P03 product:cysteine-rich hydrophobic domain 1, full insert sequence. | 0.326 |
| NM\_133878 | Mus musculus chromosome condensation 1 (Chc1), mRNA | 0.324 |
| NM\_018868 | Mus musculus nucleolar protein 5 (Nol5), mRNA | 0.324 |
| NM\_025372 | Mus musculus timeless interacting protein (Tipin), mRNA | 0.322 |
| AK046514 | Mus musculus adult male adrenal gland cDNA, RIKEN full-length enriched library, clone:B330002M01 product:hypothetical protein, full insert sequence. | 0.322 |
| NM\_010880 | Mus musculus nucleolin (Ncl), mRNA | 0.322 |
| NM\_019967 | Mus musculus deleted in bladder cancer chromosome region candidate 1 (human) (Dbccr1), mRNA | 0.321 |
| AK033068 | Mus musculus adult male corpus striatum cDNA, RIKEN full-length enriched library, clone:7630402G21 product:NEURONAL NICOTINIC ACETYLCHOLINE RECEPTOR SUBUNIT homolog [Rattus norvegicus], full insert sequence | 0.321 |
| NM\_133706 | Mus musculus RIKEN cDNA 1810014L12 gene (1810014L12Rik), mRNA | 0.321 |
| BC025645 | Mus musculus RIKEN cDNA E030034P13 gene, mRNA (cDNA clone IMAGE:5323568), partial cds. | 0.321 |
| NM\_011131 | Mus musculus polymerase (DNA directed), delta 1, catalytic subunit (Pold1), mRNA | 0.321 |
| NM\_021274 | Mus musculus chemokine (C-X-C motif) ligand 10 (Cxcl10), mRNA | 0.320 |
| ENSMUST00000058900 | Unknown | 0.320 |
| NM\_027263 | Mus musculus RIKEN cDNA 2610040C18 gene (2610040C18Rik), mRNA | 0.320 |
| ENSMUST00000037336 | Unknown | 0.319 |
| NM\_053173 | Mus musculus kinesin family member C5A (Kifc5a), mRNA | 0.318 |
| NM\_009103 | Mus musculus ribonucleotide reductase M1 (Rrm1), mRNA | 0.318 |
| M30774 | Mouse thymidylate synthase pseudogene mRNA, complete cds. | 0.318 |
| NM\_016777 | Mus musculus nuclear autoantigenic sperm protein (histone-binding) (Nasp), mRNA | 0.318 |
| NM\_029523 | Mus musculus DEP domain containing 1 (Depdc1), mRNA | 0.317 |
| NM\_011022 | Mus musculus ovary testis transcribed (Ott), mRNA | 0.316 |
| AK084256 | Mus musculus 12 days embryo eyeball cDNA, RIKEN full-length enriched library, clone:D230014L02 product:hypothetical HECT domain (Ubiquitin-protein ligase) containing protein, full insert sequence. | 0.316 |
| BC065150 | Mus musculus cDNA clone IMAGE:6856210, partial cds | 0.316 |
| AK011883 | Mus musculus 10 days embryo whole body cDNA, RIKEN full-length enriched library, clone:2610204L24 product:cyclin E2, full insert sequence. | 0.315 |
| ENSMUST00000062426 | Unknown | 0.315 |
| BC062095 | Mus musculus SRY-box containing gene 11, mRNA (cDNA clone MGC:69636 IMAGE:5716171), complete cds. | 0.314 |
| XM\_134948 | Mus musculus RIKEN cDNA 2410076I21 gene (2410076I21Rik), mRNA | 0.314 |
| NM\_027324 | Mus musculus sideroflexin 1 (Sfxn1), mRNA | 0.313 |
| NM\_009308 | Mus musculus synaptotagmin 4 (Syt4), mRNA | 0.313 |
| NM\_020567 | Mus musculus geminin (Gmnn), mRNA | 0.312 |
| NM\_027411 | Mus musculus RIKEN cDNA 2600001J17 gene (2600001J17Rik), mRNA | 0.312 |
| NM\_008446 | Mus musculus kinesin family member 4 (Kif4), mRNA | 0.312 |
| NM\_024184 | Mus musculus ASF1 anti-silencing function 1 homolog B (S. cerevisiae) (Asf1b), mRNA | 0.311 |
| NM\_013733 | Mus musculus chromatin assembly factor 1, subunit A (p150) (Chaf1a), mRNA | 0.311 |
| AK016444 | Mus musculus adult male testis cDNA, RIKEN full-length enriched library, clone:4931408D14 product:weakly similar to 2900054E04RIK PROTEIN (FRAGMENT) [Mus musculus], full insert sequence | 0.310 |
| XM\_139388 | Mus musculus similar to ras-GTPase-activating protein SH3-domain binding protein (LOC239378), mRNA | 0.308 |
| CF751326 | UI-M-HK0-cmp-o-18-0-UI.r1 NIH\_BMAP\_HK0 Mus musculus cDNA clone IMAGE:30625025 5', mRNA sequence | 0.308 |
| NM\_007765 | Mus musculus collapsin response mediator protein 1 (Crmp1), mRNA | 0.308 |
| NM\_011565 | Mus musculus TEA domain family member 2 (Tead2), mRNA | 0.308 |
| NM\_010716 | Mus musculus ligase III, DNA, ATP-dependent (Lig3), mRNA | 0.307 |
| AY344585 | Mus musculus hypothetical protein (Arzc) mRNA, complete cds. | 0.307 |
| NM\_175494 | Mus musculus zinc finger protein 367 (Zfp367), mRNA | 0.307 |
| XM\_204426 | Mus musculus similar to DYSKERIN (LOC245474), mRNA | 0.306 |
| NM\_023258 | Mus musculus PYD and CARD domain containing (Pycard), mRNA | 0.305 |
| NM\_011638 | Mus musculus transferrin receptor (Tfrc), mRNA | 0.305 |
| AK079302 | Mus musculus adult male urinary bladder cDNA, RIKEN full-length enriched library, clone:9530095P22 product:3-HYDROXY-3-METHYLGLUTARYL-COENZYME A REDUCTASE (EC 1.1.1.34) (HMG-COA REDUCTASE) homolog [Cricetulus griseus], full insert sequence. | 0.304 |
| NM\_144818 | Mus musculus RIKEN cDNA A730011O11 gene (A730011O11Rik), mRNA | 0.303 |
| NM\_016925 | Mus musculus Fanconi anemia, complementation group A (Fanca), mRNA | 0.302 |
| NM\_133994 | Mus musculus glutathione S-transferase, theta 3 (Gstt3), mRNA | 0.302 |
| NM\_025565 | Mus musculus RIKEN cDNA 2600017H08 gene (2600017H08Rik), mRNA | 0.302 |
| NM\_027504 | Mus musculus PR domain containing 16 (Prdm16), mRNA | 0.301 |
| NM\_008921 | Mus musculus DNA primase, p49 subunit (Prim1), mRNA | 0.300 |
| NM\_011495 | Mus musculus polo-like kinase 4 (Drosophila) (Plk4), mRNA | 0.299 |
| NM\_008722 | Mus musculus nucleophosmin 1 (Npm1), mRNA | 0.298 |
| NM\_025979 | Mus musculus microtubule associated serine/threonine kinase-like (Mastl), mRNA | 0.298 |
| NM\_175384 | Mus musculus RIKEN cDNA 2610311M19 gene (2610311M19Rik), mRNA | 0.298 |
| NM\_173006 | Mus musculus paraoxonase 3 (Pon3), mRNA | 0.298 |
| NM\_172578 | Mus musculus expressed sequence C79407 (C79407), mRNA | 0.297 |
| NM\_026024 | Mus musculus RIKEN cDNA 2700084L22 gene (2700084L22Rik), mRNA | 0.296 |
| NM\_026114 | Mus musculus eukaryotic translation initiation factor 2, subunit 1 alpha (Eif2s1), mRNA | 0.296 |
| NM\_027407 | Mus musculus RIKEN cDNA 1700030B17 gene (1700030B17Rik), mRNA | 0.296 |
| AK006487 | Mus musculus adult male testis cDNA, RIKEN full-length enriched library, clone:1700029F09 product:hypothetical Esterase/lipase/thioesterase family active site containing protein, full insert sequence. | 0.296 |
| NM\_029976 | Mus musculus DNA segment, Chr 11, ERATO Doi 497, expressed (D11Ertd497e), mRNA | 0.295 |
| NM\_025422 | Mus musculus RIKEN cDNA 1110055L24 gene (1110055L24Rik), mRNA | 0.295 |
| AK034534 | Mus musculus 12 days embryo embryonic body between diaphragm region and neck cDNA, RIKEN full-length enriched library, clone:9430001O05 product:cytoskeleton associated protein 2, full insert sequence. | 0.293 |
| NM\_019731 | Mus musculus expressed in non-metastatic cells 4, protein (Nme4), mRNA | 0.293 |
| NM\_010517 | Mus musculus insulin-like growth factor binding protein 4 (Igfbp4), mRNA | 0.293 |
| BC049694 | Mus musculus cDNA clone MGC:58593 IMAGE:6705916, complete cds. | 0.292 |
| NM\_009862 | Mus musculus cell division cycle 45 homolog (S. cerevisiae)-like (Cdc45l), mRNA | 0.292 |
| ENSMUST00000053041 | Unknown | 0.291 |
| NM\_025380 | Mus musculus eukaryotic translation elongation factor 1 epsilon 1 (Eef1e1), mRNA | 0.291 |
| NM\_025596 | Mus musculus RIKEN cDNA 2610524G07 gene (2610524G07Rik), mRNA | 0.291 |
| NM\_020010 | Mus musculus cytochrome P450, 51 (Cyp51), mRNA | 0.291 |
| NM\_010448 | Mus musculus heterogeneous nuclear ribonucleoprotein A/B (Hnrpab), mRNA | 0.290 |
| NM\_212484 | Mus musculus RIKEN cDNA A230103N10 gene (A230103N10Rik), mRNA | 0.290 |
| NM\_008638 | Mus musculus methylenetetrahydrofolate dehydrogenase (NAD+ dependent), methenyltetrahydrofolate cyclohydrolase (Mthfd2), mRNA | 0.290 |
| NM\_025866 | Mus musculus cell division cycle associated 7 (Cdca7), mRNA | 0.290 |
| NM\_007486 | Mus musculus Rho, GDP dissociation inhibitor (GDI) beta (Arhgdib), mRNA | 0.289 |
| NM\_008889 | Mus musculus protein phosphatase 1, regulatory (inhibitor) subunit 14B (Ppp1r14b), mRNA | 0.289 |
| NAP107172-1 | Unknown | 0.288 |
| NM\_011727 | Mus musculus X-linked lymphocyte-regulated 3b (Xlr3b), mRNA | 0.288 |
| AK036786 | Mus musculus adult female vagina cDNA, RIKEN full-length enriched library, clone:9930009M05 product:unknown EST, full insert sequence | 0.288 |
| NM\_011797 | Mus musculus carbonic anhydrase 14 (Car14), mRNA | 0.287 |
| NM\_008566 | Mus musculus minichromosome maintenance deficient 5, cell division cycle 46 (S. cerevisiae) (Mcm5), mRNA | 0.287 |
| AK034079 | Mus musculus adult male diencephalon cDNA, RIKEN full-length enriched library, clone:9330154M02 product:Rho interacting protein 2, full insert sequence. | 0.286 |
| NM\_011358 | Mus musculus splicing factor, arginine/serine-rich 2 (SC-35) (Sfrs2), mRNA | 0.286 |
| NM\_010338 | Mus musculus G protein-coupled receptor 37 (Gpr37), mRNA | 0.285 |
| NM\_021891 | Mus musculus fidgetin-like 1 (Fignl1), mRNA | 0.284 |
| NM\_025900 | Mus musculus DEK oncogene (DNA binding) (Dek), mRNA | 0.284 |
| AK084529 | Mus musculus 13 days embryo heart cDNA, RIKEN full-length enriched library, clone:D330012D13 product:hypothetical protein, full insert sequence. | 0.284 |
| NM\_026115 | Mus musculus histone aminotransferase 1 (Hat1), mRNA | 0.283 |
| ENSMUST00000049171 | Unknown | 0.283 |
| AK013116 | Mus musculus 10, 11 days embryo whole body cDNA, RIKEN full-length enriched library, clone:2810418N01 product:HYPOTHETICAL PROTEIN KIAA0186 homolog [Homo sapiens], full insert sequence. | 0.283 |
| AK028407 | Mus musculus 13 days embryo whole body cDNA, RIKEN full-length enriched library, clone:3930401C23 product:T-box 4, full insert sequence. | 0.282 |
| XM\_355303 | Mus musculus RIKEN cDNA 1700029F09 gene (1700029F09Rik), mRNA | 0.281 |
| NM\_007984 | Mus musculus fascin homolog 1, actin bundling protein (Strongylocentrotus) purpuratus) (Fscn1), mRNA | 0.280 |
| ENSMUST00000060297 | Unknown | 0.279 |
| NM\_007691 | Mus musculus checkpoint kinase 1 homolog (S. pombe) (Chek1), mRNA | 0.279 |
| AK012568 | Mus musculus 11 days embryo whole body cDNA, RIKEN full-length enriched library, clone:2700085B09 product:high mobility group box 2, full insert sequence. | 0.279 |
| NM\_138305 | Mus musculus adenylate cyclase 3 (Adcy3), mRNA | 0.278 |
| BC050071 | Mus musculus RIKEN cDNA 2610036L11 gene, mRNA (cDNA clone IMAGE:30021627), partial cds | 0.278 |
| NM\_027290 | Mus musculus minichromosome maintenance deficient 10 (S. cerevisiae) (Mcm10), mRNA | 0.277 |
| NM\_146403 | Mus musculus olfactory receptor 1295 (Olfr1295), mRNA | 0.277 |
| AK017750 | Mus musculus 8 days embryo whole body cDNA, RIKEN full-length enriched library, clone:5730505K17 product:hypothetical protein, full insert sequence. | 0.276 |
| NM\_008563 | Mus musculus minichromosome maintenance deficient 3 (S. cerevisiae) (Mcm3), mRNA | 0.276 |
| AK088548 | Mus musculus 2 days neonate thymus thymic cells cDNA, RIKEN full-length enriched library, clone:E430020A07 product:hypothetical protein, full insert sequence. | 0.275 |
| NM\_019741 | Mus musculus solute carrier family 2 (facilitated glucose transporter), member 5 (Slc2a5), mRNA | 0.274 |
| NM\_010477 | Mus musculus heat shock protein 1 (chaperonin) (Hspd1), mRNA | 0.274 |
| NM\_013726 | Mus musculus expressed sequence AA545217 (AA545217), mRNA | 0.271 |
| BC003261 | Mus musculus aurora kinase B, mRNA (cDNA clone MGC:5803 IMAGE:3501444), complete cds. | 0.271 |
| XM\_134100 | Mus musculus cytoskeleton associated protein 2 (Ckap2), mRNA | 0.270 |
| NM\_011497 | Mus musculus serine/threonine kinase 6 (Stk6), mRNA | 0.270 |
| NM\_133977 | Mus musculus transferrin (Trf), mRNA | 0.268 |
| NM\_009387 | Mus musculus thymidine kinase 1 (Tk1), mRNA | 0.266 |
| NM\_145942 | Mus musculus 3-hydroxy-3-methylglutaryl-Coenzyme A synthase 1 (Hmgcs1), mRNA | 0.266 |
| BC027537 | Mus musculus RIKEN cDNA 2810418N01 gene, mRNA (cDNA clone MGC:41228 IMAGE:3466154), complete cds. | 0.266 |
| NM\_007792 | Mus musculus cysteine and glycine-rich protein 2 (Csrp2), mRNA | 0.265 |
| NM\_177912 | Mus musculus expressed sequence AI987692 (AI987692), mRNA | 0.263 |
| NM\_021886 | Mus musculus centromere autoantigen H (Cenph), mRNA | 0.263 |
| NM\_016750 | Mus musculus H2A histone family, member Z (H2afz), mRNA | 0.262 |
| AK048650 | Mus musculus 16 days embryo head cDNA, RIKEN full-length enriched library, clone:C130093K11 product:SMC4 PROTEIN homolog [Microtus arvalis], full insert sequence. | 0.262 |
| NM\_175654 | Mus musculus histone 1, H4d (Hist1h4d), mRNA | 0.260 |
| NM\_009773 | Mus musculus budding uninhibited by benzimidazoles 1 homolog, beta (S. cerevisiae) (Bub1b), mRNA | 0.260 |
| NM\_026631 | Mus musculus nucleolar protein family A, member 2 (Nola2), mRNA | 0.259 |
| NM\_016662 | Mus musculus Max dimerization protein 3 (Mxd3), mRNA | 0.259 |
| NAP070876-1 | Unknown | 0.258 |
| NM\_024245 | Mus musculus kinesin family member 23 (Kif23), mRNA | 0.258 |
| NM\_026014 | Mus musculus retroviral integration site 2 (Ris2), mRNA | 0.258 |
| AK010905 | Mus musculus 13 days embryo liver cDNA, RIKEN full-length enriched library, clone:2510004J16 product:similar to HYPOTHETICAL 31.3 KDA PROTEIN [Homo sapiens], full insert sequence. | 0.258 |
| BC060208 | Mus musculus RIKEN cDNA 2810047L02 gene, mRNA (cDNA clone MGC:64712 IMAGE:6837782), complete cds. | 0.258 |
| NM\_007681 | Mus musculus centromere autoantigen A (Cenpa), mRNA | 0.256 |
| NM\_026374 | Mus musculus interleukin enhancer binding factor 2 (Ilf2), mRNA | 0.256 |
| AK011467 | Mus musculus 10 days embryo whole body cDNA, RIKEN full-length enriched library, clone:2610019J24 product:high mobility group box 2, full insert sequence. | 0.255 |
| NM\_001001176 | Mus musculus cDNA sequence BC066223 (BC066223), mRNA | 0.255 |
| NM\_010049 | Mus musculus dihydrofolate reductase (Dhfr), mRNA | 0.252 |
| NM\_134469 | Mus musculus farnesyl diphosphate synthetase (Fdps), mRNA | 0.252 |
| NM\_018815 | Mus musculus nucleoporin 210 (Nup210), mRNA | 0.252 |
| ENSMUST00000035502 | Unknown | 0.252 |
| NM\_011670 | Mus musculus ubiquitin carboxy-terminal hydrolase L1 (Uchl1), mRNA | 0.251 |
| NM\_010480 | Mus musculus heat shock protein 1, alpha (Hspca), mRNA | 0.250 |
| U42385 | Mus musculus fibroblast growth factor inducible gene 16 (FIN16) mRNA, complete cds. | 0.247 |
| NM\_146040 | Mus musculus cDNA sequence BC006933 (BC006933), mRNA | 0.247 |
| NM\_146235 | Mus musculus cDNA sequence BC004701 (BC004701), mRNA | 0.245 |
| NM\_173169 | Mus musculus polo-like kinase 4 (Drosophila) (Plk4), mRNA | 0.243 |
| AF420436 | Mus musculus strain FVB Abca12 mRNA, partial cds. | 0.242 |
| NM\_009828 | Mus musculus cyclin A2 (Ccna2), mRNA | 0.238 |
| NM\_146902 | Mus musculus olfactory receptor 1221 (Olfr1221), mRNA | 0.238 |
| AK083530 | Mus musculus 9 days embryo whole body cDNA, RIKEN full-length enriched library, clone:D030041G16 product:hypothetical protein, full insert sequence. | 0.236 |
| NAP029013-1 | Unknown | 0.236 |
| AK041640 | Mus musculus 3 days neonate thymus cDNA, RIKEN full-length enriched library, clone:A630026H08 product:zinc finger protein, subfamily 1A, 4, full insert sequence. | 0.236 |
| NM\_133786 | Mus musculus SMC4 structural maintenance of chromosomes 4-like 1 (yeast) (Smc4l1), mRNA | 0.235 |
| NM\_010097 | Mus musculus SPARC-like 1 (mast9, hevin) (Sparcl1), mRNA | 0.235 |
| NM\_146171 | Mus musculus RIKEN cDNA 2810406C15 gene (2810406C15Rik), mRNA | 0.234 |
| NM\_181589 | Mus musculus RIKEN cDNA 2610318C08 gene (2610318C08Rik), mRNA | 0.234 |
| NAP052665-1 | Unknown | 0.234 |
| NM\_139269 | Mus musculus HRAS like suppressor 3 (Hrasls3), mRNA | 0.233 |
| NM\_008892 | Mus musculus polymerase (DNA directed), alpha 1 (Pola1), mRNA | 0.231 |
| NM\_009892 | Mus musculus chitinase 3-like 3 (Chi3l3), mRNA | 0.230 |
| NM\_177960 | Mus musculus isopentenyl-diphosphate delta isomerase (Idi1), mRNA | 0.230 |
| NM\_010615 | Mus musculus kinesin family member 11 (Kif11), mRNA | 0.230 |
| NM\_175329 | Mus musculus RIKEN cDNA 1620401E04 gene (1620401E04Rik), mRNA | 0.228 |
| BC052738 | Mus musculus synaptotagmin 4, mRNA (cDNA clone MGC:64624 IMAGE:6832499), complete cds. | 0.226 |
| NM\_023595 | Mus musculus deoxyuridine triphosphatase (Dutp), mRNA | 0.225 |
| NM\_013882 | Mus musculus G two S phase expressed protein 1 (Gtse1), mRNA | 0.225 |
| NM\_027495 | Mus musculus RIKEN cDNA 5730537D05 gene (5730537D05Rik), mRNA | 0.224 |
| NM\_028390 | Mus musculus anillin, actin binding protein (scraps homolog, Drosophila) (Anln), mRNA | 0.224 |
| X82786 | M.musculus mRNA for Ki-67. | 0.223 |
| NM\_010655 | Mus musculus karyopherin (importin) alpha 2 (Kpna2), mRNA | 0.222 |
| NM\_025581 | Mus musculus RIKEN cDNA 2810433K01 gene (2810433K01Rik), mRNA | 0.222 |
| NM\_011133 | Mus musculus polymerase (DNA directed), epsilon 2 (p59 subunit) (Pole2), mRNA | 0.221 |
| NM\_008568 | Mus musculus minichromosome maintenance deficient 7 (S. cerevisiae) (Mcm7), mRNA | 0.221 |
| NM\_011799 | Mus musculus cell division cycle 6 homolog (S. cerevisiae) (Cdc6), mRNA | 0.219 |
| AK017868 | Mus musculus 8 days embryo whole body cDNA, RIKEN full-length enriched library, clone:5730576N04 product:hypothetical protein, full insert sequence. | 0.219 |
| NM\_009766 | Mus musculus bombesin-like receptor 3 (Brs3), mRNA | 0.214 |
| NM\_012025 | Mus musculus Rac GTPase-activating protein 1 (Racgap1), mRNA | 0.214 |
| NM\_026412 | Mus musculus DNA segment, Chr 2, ERATO Doi 750, expressed (D2Ertd750e), mRNA | 0.212 |
| NAP030643-1 | Unknown | 0.211 |
| NM\_175119 | Mus musculus RIKEN cDNA 1300007C21 gene (1300007C21Rik), mRNA | 0.209 |
| NM\_007659 | Mus musculus cell division cycle 2 homolog A (S. pombe) (Cdc2a), mRNA | 0.209 |
| NAP072161-1 | Unknown | 0.209 |
| NM\_026038 | Mus musculus RIKEN cDNA 2810055F11 gene (2810055F11Rik), mRNA | 0.207 |
| NM\_011045 | Mus musculus proliferating cell nuclear antigen (Pcna), mRNA | 0.207 |
| NM\_175177 | Mus musculus 3-hydroxybutyrate dehydrogenase (heart, mitochondrial) (Bdh), mRNA | 0.206 |
| NM\_011234 | Mus musculus RAD51 homolog (S. cerevisiae) (Rad51), mRNA | 0.204 |
| NM\_026139 | Mus musculus armadillo repeat containing, X-linked 2 (Armcx2), mRNA | 0.202 |
| NM\_011700 | Mus musculus villin-like protein (Villp), mRNA | 0.200 |
| NM\_021790 | Mus musculus SoxLZ/Sox6 leucine zipper binding protein in testis (Solt), mRNA | 0.200 |
| NM\_008017 | Mus musculus SMC2 structural maintenance of chromosomes 2-like 1 (yeast) (Smc2l1), mRNA | 0.198 |
| NM\_009791 | Mus musculus calmodulin binding protein 1 (Calmbp1), mRNA | 0.198 |
| NM\_019641 | Mus musculus stathmin 1 (Stmn1), mRNA | 0.196 |
| NM\_015767 | Mus musculus tocopherol (alpha) transfer protein (Ttpa), mRNA | 0.195 |
| AK015074 | Mus musculus adult male testis cDNA, RIKEN full-length enriched library, clone:4930404C15 product:3-phosphoglycerate dehydrogenase, full insert sequence. | 0.191 |
| NM\_013538 | Mus musculus cell division cycle associated 3 (Cdca3), mRNA | 0.190 |
| NM\_011576 | Mus musculus tissue factor pathway inhibitor (Tfpi), mRNA | 0.190 |
| NM\_007900 | Mus musculus ect2 oncogene (Ect2), mRNA | 0.190 |
| NM\_011369 | Mus musculus Shc SH2-domain binding protein 1 (Shcbp1), mRNA | 0.186 |
| NM\_019499 | Mus musculus MAD2 (mitotic arrest deficient, homolog)-like 1 (yeast) (Mad2l1), mRNA | 0.186 |
| NM\_173762 | Mus musculus centromere protein E (Cenpe), mRNA | 0.184 |
| AK009772 | Mus musculus adult male tongue cDNA, RIKEN full-length enriched library, clone:2310043D08 product:hypothetical protein, full insert sequence. | 0.183 |
| NM\_010777 | Mus musculus myelin basic protein (Mbp), mRNA | 0.182 |
| NM\_026167 | Mus musculus kelch-like 13 (Drosophila) (Klhl13), mRNA | 0.180 |
| NM\_057173 | Mus musculus LIM domain only 1 (Lmo1), mRNA | 0.176 |
| AY259532 | Mus musculus M-phase phosphoprotein mRNA, complete cds. | 0.176 |
| NM\_172296 | Mus musculus doublesex and mab-3 related transcription factor like family A2 (Dmrta2), mRNA | 0.175 |
| NM\_033571 | Mus musculus FK506 binding protein 6 (Fkbp6), mRNA | 0.174 |
| NM\_144526 | Mus musculus RIKEN cDNA 6720460F02 gene (6720460F02Rik), mRNA | 0.173 |
| NM\_023209 | Mus musculus PDZ binding kinase (Pbk), mRNA | 0.171 |
| AK045067 | Mus musculus 9.5 days embryo parthenogenote cDNA, RIKEN full-length enriched library, clone:B130024C23 product:KINESIN SUPERFAMILY PROTEIN 20B (FRAGMENT) homolog [Mus musculus], full insert sequence. | 0.169 |
| NM\_009772 | Mus musculus budding uninhibited by benzimidazoles 1 homolog (S. cerevisiae) (Bub1), mRNA | 0.165 |
| NM\_008234 | Mus musculus helicase, lymphoid specific (Hells), mRNA | 0.165 |
| U83902 | Mus musculus mitotic checkpoint component Mad2 mRNA, complete cds. | 0.164 |
| NM\_008564 | Mus musculus minichromosome maintenance deficient 2 mitotin (S. cerevisiae) (Mcm2), mRNA | 0.164 |
| NM\_010931 | Mus musculus ubiquitin-like, containing PHD and RING finger domains, 1 (Uhrf1), mRNA | 0.161 |
| NM\_025975 | Mus musculus t-complex-associated-testis-expressed 1-like (Tcte1l), mRNA | 0.157 |
| AK028715 | Mus musculus 10 days neonate skin cDNA, RIKEN full-length enriched library, clone:4732442J12 product:unknown EST, full insert sequence. | 0.157 |
| NM\_008125 | Mus musculus gap junction membrane channel protein beta 2 (Gjb2), mRNA | 0.152 |
| AJ237585 | Mus musculus mRNA for hypothetical protein expressed in thymocytes (clone MFT.M05.13/MTA.B10.066), partial. | 0.148 |
| NM\_025415 | Mus musculus CDC28 protein kinase regulatory subunit 2 (Cks2), mRNA | 0.146 |
| NM\_010807 | Mus musculus MARCKS-like protein (Mlp), mRNA | 0.138 |
| NM\_021365 | Mus musculus X-linked lymphocyte-regulated 4 (Xlr4), mRNA | 0.131 |
| XM\_132481 | Mus musculus similar to G2/mitotic-specific cyclin B1 (LOC231869), mRNA | 0.125 |
| NM\_172301 | Mus musculus cyclin B1 (Ccnb1), mRNA | 0.121 |
| NM\_016966 | Mus musculus 3-phosphoglycerate dehydrogenase (Phgdh), mRNA | 0.108 |
| NM\_011623 | Mus musculus topoisomerase (DNA) II alpha (Top2a), mRNA | 0.105 |
| NM\_010140 | Mus musculus Eph receptor A3 (Epha3), mRNA | 0.084 |
| NM\_008581 | Mus musculus melanoma antigen (Mela), mRNA | 0.083 |
