# Supplementary material for: Global analysis of gene expression changes during retinoic acid-induced growth arrest and differentiation of melanoma: comparison to differentially expressed genes in melanocytes vs melanoma
Source: BMC Genomics. 2008 Oct 11;9:478. doi: 10.1186/1471-2164-9-478 (PMC2572629; doi:10.1186/1471-2164-9-478)
Supplement: Additional file 3 — Members of the 203 gene set, fold change at 48 h of RA treatment and relative expression in melan-a vs. B16 cells. [file 1471-2164-9-478-S3.pdf]

Additional file 3.

Members of the 203 gene set, fold change at 48 h of RA treatment and relative expression in melan-a vs. B16 cells.

Accession#	Name	B16 + RA 48hr	melan-a/ B16
NM_011623	topoisomerase (dna) ii alpha; top2a	0.51	0.11
NM_025415	CDC28 protein kinase regulatory subunit 2	0.57	0.15
AJ237585	chromosome condensation protein G	0.49	0.15
NM_008564	mini chromosome maintenance deficient 2; mcmd2	0.60	0.16
NM_023209	t-cell-originated protein kinase; 2810434b10rik	0.46	0.17
NM_057173	lim only 1; lmo1	0.33	0.18
NM_019499	mitotic arrest deficient-like 1; mad2l1	0.53	0.19
NM_011369	shc sh2-domain binding protein 1; shcbp1	0.45	0.19
NM_007900	ect2 oncogene; ect2	0.46	0.19
NM_013538	cell division cycle associated 3	0.49	0.19
NM_009791	calmodulin binding protein 1; calmbp1	0.46	0.20
NM_026139	armadillo repeat containing, X-linked 2	0.66	0.20
NM_011234	rad51 homolog (s. cerevisiae); rad51	0.58	0.20
NM_007659	cell division cycle 2 homolog a (s. pombe); cdc2a	0.48	0.21
NM_011799	cell division cycle 6 homolog (s. cerevisiae); cdc6	0.52	0.22
NM_025581	riken cdna 2810433k01; 2810433k01rik	0.57	0.22
NM_013882	g two s phase expressed protein 1; gtse1	0.53	0.23
NM_023595	dUTP pyrophosphatase	0.63	0.23
NM_008892	dna polymerase alpha 1; 180 kda; pola1	0.44	0.23
AK010905	cell division cycle associated 8	0.45	0.26
NM_016662	max dimerization protein 3; mad3	0.45	0.26
NM_026631	nucleolar protein family A, member 2	0.58	0.26
NM_016750	h2a histone family; member z; h2afz	0.56	0.26
NM_007691	checkpoint kinase 1 homolog (s. pombe); chek1	0.48	0.28
AK013116	homolog to hypothetical protein kiaa0186	0.35	0.28
NM_025380	eukaryotic translation elongation factor 1 epsilon 1	0.51	0.29
NM_009862	cell division cycle 45 homolog-like; cdc45l	0.58	0.29
AK006487	RIKEN cDNA 1700029F09 gene	0.49	0.30
NM_026024	HSPC150 protein	0.55	0.30
NM_025979	microtubule associated serine/threonine kinase-like	0.44	0.30
NM_008722	nucleophosmin 1; npm1	0.55	0.30
NM_011638	transferrin receptor; trfr	0.60	0.30
NM_020567	geminin; geminin-pending	0.58	0.31
NM_009308	synaptotagmin 4; syt4	0.68	0.31
AK011883	cdna clone cyclin e2	0.24	0.32
NM_019482	pannexin 1; panx1	0.59	0.35
NM_009765	breast cancer 2; brca2	0.54	0.35
NM_019939	membrane protein (maguk p55 subfamily member 6)	0.34	0.35
NM_023058	membrane-associated cdc2-inhibitory kinase	0.55	0.35
NM_016904	cdc28 protein kinase 1; cks1	0.41	0.36
NM_009391	ran; member ras oncogene family; ran	0.51	0.36
NM_011132	dna polymerase epsilon; pole	0.45	0.36

NM_007684	centrin 3; cetn3	0.54	0.37
NM_011514	suppressor of variegation 3-9 homolog 1; suv39h1	0.49	0.37
NM_026030	eukaryotic translation initiation factor 2; subunit 2	0.53	0.38
NM_009770	b-cell translocation gene 3; btg3	0.55	0.38
NM_022724	suppressor of variegation 3-9 homologue 2; suv39h2	0.53	0.39
NM_009584	zuotin related factor 2; zrf2	0.44	0.39
NM_010849	myelocytomatosis oncogene; myc	0.50	0.40
NM_024169	FK506 binding protein 11, 19 kDa	0.37	0.40
NM_023117	cell division cycle 25 homolog b; cdc25b	0.62	0.41
NM_024223	cysteine-rich protein 2	0.54	0.41
NM_026656	mucolipin 2	0.18	0.42
NM_009361	transcription factor dp 1; tfdp1	0.56	0.43
NM_026352	peptidylprolyl isomerase d (cyclophilin d); ppid	0.58	0.43
NM_010344	glutathione reductase 1; gsr	0.65	0.43
NM_025928	polyamine-modulated factor 1	0.48	0.44
NM_011499	serine/threonine kinase receptor associated protein	0.52	0.45
NM_028712	rap2b; member of ras oncogene family; rap2b	0.37	0.45
AK014396	copine IV	0.48	0.45
NM_011605	thymopoietin; tmpo	0.64	0.46
NM_019814	hypoxia induced gene 1; hig1-pending	0.55	0.46
NM_019698	aldehyde dehydrogenase 18 family, member A1	0.49	0.46
NM_008567	mini chromosome maintenance deficient 6; mcmd6	0.60	0.46
NM_010247	thyroid autoantigen 70 kda; g22p1	0.61	0.46
NM34084	expressed sequence aw457192; aw457192	0.66	0.46
NM_008799	programmed cell death 2; pdcd2	0.51	0.47
NM_016710	nucleosome binding protein 1; nsbp1	0.57	0.47
NM_025695	SMC6 structural maintenance of chromosomes 6	0.62	0.47
NM_011284	replication protein a2; rpa2	0.47	0.47
AK014608	homolog to cdna flj13936 fis; clone y79aa1000802	0.26	0.48
NM_021511	regulator for ribosome resistance homolog	0.54	0.48
NM_009226	small nuclear ribonucleoprotein d1; snrpd1	0.54	0.49
NM_020619	glucosidase 1; gcs1	0.59	0.49
NM_025564	riken cdna 2010012c16; 2010012c16rik	0.60	0.49
NM_009716	activating transcription factor 4; atf4	0.47	0.49
NM_013536	gene rich cluster; c2f gene; grcc2f	0.62	0.49
NM_016905	galactokinase; glk	0.51	0.49
NM_013562	interferon-related developmental regulator 1; ifrd1	0.53	0.49
NM_008298	dnaj (hsp40) homolog; subfamily a; member 1	0.49	0.49
AK011942	cdna clone homolog to dj383j4.3	0.25	0.49
NM_019468	glucose-6-phosphate dehydrogenase 2; g6pd2	0.48	0.49
NM_010634	fatty acid binding protein 5 (psoriasis-associated)	0.31	0.50
AF194970	centromere protein F, 350/400ka (mitosin)	0.55	0.51
NM_010792	methyltransferase-like 1 (s. cerevisiae); mettl1	0.52	0.51
NM_028232	shugoshin-like 1 (S. pombe)	0.50	0.51
NM_009004	rab6; kinesin-like; rab6kifl	0.57	0.51
NM_015762	thioredoxin reductase 1; txnrd1	0.86	0.52
NM33807	expressed sequence aa959742; aa959742	0.49	0.52
NM_007634	cyclin f; ccnf	0.57	0.53
NM_016661	s-adenosylhomocysteine hydrolase; ahcy	0.50	0.53

NM_015774	ero1-like (s. cerevisiae); ero1l	0.41	0.53
NM_009193	stem-loop binding protein; slbp	0.60	0.53
NM_013898	translocase of inner mitochondrial membrane 8	0.29	0.53
NM_053089	riken cdna 5730450d16; narg1-pending	0.61	0.53
NM_009013	rad51 associated protein 1; rad51ap1	0.61	0.54
NM33933	expressed sequence au018702; au018702	0.51	0.54
NM_018757	expressed in non-metastatic cells 6; protein; nme6	0.45	0.54
NM_025310	FtsJ homolog 3 (E. coli)	0.52	0.54
NM_025281	ly1 antibody reactive clone; lyar	0.60	0.55
NM_009689	baculoviral iap repeat-containing 5; birc5	0.42	0.55
NM_010178	FUS interacting protein (serine-arginine rich) 1	0.58	0.55
NM34092	expressed sequence ai429604; ai429604	0.34	0.56
NM_007669	cyclin-dependent kinase inhibitor 1a (p21); cdkn1a	0.60	0.57
NM_007573	complement component 1; c1qbp	0.49	0.58
NM_011640	transformation related protein 53; trp53	0.55	0.58
NM_026041	CGI-115 protein	0.40	0.59
AK010292	homolog to ribonuclease hi large subunit (ec 3.1.26.)	0.49	0.59
NM_025904	riken cdna 1600012f09; 1600012f09rik	0.56	0.59
NM_019836	hypothetical brain protein similar to x96994 br-1	0.57	0.59
NM_016897	translocase of inner mitochondrial membrane 23	0.65	0.60
NM_008186	general transcription factor ii h; polypeptide 1	0.47	0.60
NM_010442	heme oxygenase (decycling) 1; hmox1	0.40	0.60
BC006867	unknown (protein for mgc:11792)	0.62	0.61
AK012959	cdna clone zinc finger; c2h2 type containing protein	0.61	0.61
NM_023323	brix domain containing 1	0.41	0.61
BC027063	3-hydroxybutyrate dehydrogenase	0.34	0.62
NM_021512	nucleoporin 160kDa	0.57	0.62
NM33678	riken cdna 2410004c24; 2410004c24rik	0.60	0.62
NM_012058	signal recognition particle 9 kda; srp9	0.62	0.63
NM_009472	unc-5 homolog (c. elegans) 3; unc5h3	0.32	0.63
BC022907	unknown (protein for mgc:25558)	0.48	0.64
NM34151	expressed sequence al024047; al024047	0.64	0.64
X92590	hira protein; hira	0.59	0.64
NM_010481	heat shock protein; 74 kda; a; hspa9a	0.34	0.64
AK021408	trophinin associated protein (tastin)	0.45	0.65
NM_013699	upstream binding protein 1; ubp1	0.56	0.65
NM_018776	cytokine receptor-like factor 3; crlf3	0.61	0.65
NM_018861	neutral amino acid transporter; slc1a4	0.44	0.65
NM_011304	ruvb-like protein 2; ruvbl2	0.51	0.66
NM33227	nucleoporin 155; nup155	0.55	0.66
NM_008565	mini chromosome maintenance deficient 4	0.49	0.67

NM_009465	axl receptor tyrosine kinase; axl	1.70	10.38
NM_009263	secreted phosphoprotein 1; spp1	3.46	9.44
NM_021278	thymosin; beta 4; x chromosome; tmsb4x	3.96	9.17
NM_011340	serine (or cysteine) proteinase inhibitor; clade f	1.71	4.51
NM_009155	selenoprotein p; plasma; 1; sepp1	2.72	4.23
NM_010501	interferon-induced protein; ifit3	2.02	4.11
NM_023476	lipocalin 7; lcn7	1.30	3.99
AK007352	cdna clone hypothetical protein	3.31	3.77

NM_008655	growth arrest and dna-damage-inducible 45 beta	2.29	3.76
NM_054077	proline arginine-rich end leucine-rich repeat; prelp	2.15	3.51
NM_008515	leucine rich repeat (in flii) interacting protein 1; lrrfip1	1.70	3.36
NM_025626	riken cdna 3110001a13; 3110001a13rik	1.04	3.32
NM_009373	transglutaminase 2; c polypeptide; tgm2	2.14	3.22
NM_008872	plasminogen activator; tissue; plat	1.96	3.21
NM_009136	scrapie responsive gene 1; scrg1	1.90	3.00
NM_008862	protein kinase inhibitor; alpha; pkia	1.86	2.91
NM_011303	retinal short-chain dehydrogenase/reductase 1	4.64	2.69
NM_010260	guanylate nucleotide binding protein 2; gbp2	2.56	2.66
NM_018827	cytokine receptor-like factor 1; crlf1	1.83	2.51
NM_008410	integral membrane protein 2b; itm2b	1.79	2.45
NM_008492	lactate dehydrogenase 2; b chain; ldh2	2.17	2.43
NM_008609	matrix metalloproteinase 15; mmp15	1.83	2.42
NM_053146	protocadherin beta 21; pcdhb21	3.32	2.35
NM_010197	fibroblast growth factor 1; fgf1	2.20	2.28
NM_009636	ae binding protein 1; aebp1	1.58	2.20
NM_030261	sestrin 3	1.58	2.19
NM_007899	extracellular matrix protein 1; ecm1	2.51	2.19
NM_008010	fibroblast growth factor receptor 3; fgfr3	1.75	2.18
NM_007399	a disintegrin and metalloprotease domain 10	1.59	2.11
NM_028375	riken cdna 2900027g03; 2900027g03rik	1.89	2.10
NM_053078	neuronal protein 3.1; d0h4s114	2.03	2.10
NM_018804	synaptotagmin 11; syt11	2.03	2.05
NM_009242	secreted acidic cysteine rich glycoprotein; sparc	2.26	2.05
AK009137	homolog to kiaa1434 protein (fragment)	1.97	2.03
NM_020573	oxysterol binding protein-like 1a; osbpl1a	1.96	2.02
BC011344	unknown (protein for mgc:5739)	2.60	2.01
AK018640	homolog to dj462o23.2 (novel protein)	1.65	1.99
NM_016765	dimethylarginine dimethylaminohydrolase 2; ddah2	2.20	1.94
NM_010764	mannosidase 2; alpha b1; man2b1	1.74	1.94
NM_008905	protein tyrosine phosphatase; receptor-type; ppfibp2	1.83	1.94
NM_030706	tripartite motif protein trim2; trim2	2.10	1.90
NM_009115	s100 protein; beta polypeptide; neural; s100b	3.23	1.86
NM_009923	cyclic nucleotide phosphodiesterase 1; cnp1	3.62	1.83
NM_007709	cbp/p300-interacting transactivator; cited1	3.24	1.82
NM_010753	max dimerization protein 4; mad4	1.96	1.80
U34361	laf-4	2.12	1.79
NM_011050	programmed cell death 4; pdcd4	1.73	1.78
NM33716	riken cdna 1810031k02; 1810031k02rik	1.13	1.77
S60315	myotonic dystrophy kinase; dmr-b15	1.84	1.76
X15052	neural cell adhesion molecule ncam-180	2.37	1.74
NM34030	expressed sequence ai849362; ai849362	2.20	1.74
NM_019963	signal transducer and activator of transcription 2	1.63	1.74
NM_053141	protocadherin beta 16; pcdhb16	2.89	1.74
NM_007428	angiotensinogen; agt	1.62	1.73
NM_010952	ornithine decarboxylase antizyme 2; oaz2	2.14	1.71
NM_009366	transforming growth factor β 1 induced transcript 4	2.67	1.70
NM_030259	hypothetical protein; mgc:7036; bc003324	1.99	1.70
NM_019709	membrane-bound transcription factor protease	1.43	1.68

AK008529	cdna clone homolog to phospholipase	1.69	1.68
NM_021292	ellis van creveld gene homolog (human); evc	2.08	1.65
NM_008580	mitogen activated protein kinase kinase kinase 5	1.88	1.64
NM_007760	carnitine acetyltransferase; crat	1.64	1.64
NM_024477	hypothetical protein; mgc:7623; bc002262	1.92	1.64
NM_030714	deltex3; dtx3	2.31	1.62
AK009454	homolog to cdna flj13680 fis; clone place2000007	1.35	1.61
	zinc finger protein 261; zfp261	1.74	1.60
NM_008090	gata binding protein 2; gata2	1.61	1.59
BC006717	cell cycle progression 1	1.48	1.56
NM_019919	latent transforming growth factor beta binding protein 1	7.52	1.54
NM_012032	tumor differentially expressed 1; tde1	2.13	1.52
NM_011101	protein kinase c; alpha; prkca	4.87	1.51
