# Supplementary material for: Global analysis of gene expression changes during retinoic acid-induced growth arrest and differentiation of melanoma: comparison to differentially expressed genes in melanocytes vs melanoma
Source: BMC Genomics. 2008 Oct 11;9:478. doi: 10.1186/1471-2164-9-478 (PMC2572629; doi:10.1186/1471-2164-9-478)
Supplement: Additional file 4 — Classification of 203 Gene Set Members According to Gene Ontology Biological Processes. Processes were ranked by p-value which is the Holm-Bonferroni adjusted probability of a random set of 203 genes containing at least the stated representation in the Pathway Studio database. The number of members within each process that are expressed lower in melan-a, higher in melan-a and the total number of members are provided in columns 2, 3 and 4 respectively. [file 1471-2164-9-478-S4.pdf]

Additional file 4

Classification of 203 Gene Set Members According to Gene Ontology Biological Processes.

**Biological Process**	**Expressed lower in melan-A than B16**	**Expressed higher in melan-A than B16**	**Total**	***p*-value**
cell division	18	0	18	1.82×10^{-24}
cell cycle	22	0	22	3.53×10^{-24}
regulation of cell cycle progression	19	0	19	1.06×10^{-21}
DNA replication	13	0	13	1.36×10^{-17}
Mitosis	13	0	13	4.65×10^{-17}
Transcription	16	6	22	4.02×10^{-12}
Transport	14	7	21	1.51×10^{-11}
regulation of cyclin-dependent protein kinase activity	7	0	7	2.11×10^{-10}
response to DNA damage stimulus	9	0	9	1.21×10^{-09}
DNA repair	10	0	10	1.42×10^{-09}
regulation of transcription, DNA-dependent	15	7	22	3.35×10^{-09}
cell proliferation	7	4	11	4.09×10^{-09}
Apoptosis	6	5	11	2.20×10^{-08}
negative regulation of cell proliferation	6	3	9	4.86×10^{-08}
DNA unwinding during replication	5	0	5	5.67×10^{-08}
protein amino acid phosphorylation	5	7	12	1.12×10^{-07}
DNA replication initiation	5	0	5	1.54×10^{-07}
regulation of transcription	8	2	10	1.86×10^{-06}
anti-apoptosis	5	2	7	4.61×10^{-06}
regulation of transcription from RNA polymerase II promoter	5	3	8	4.84×10^{-06}
cyclin-dependent protein kinase regulator activity	4	0	4	1.93×10^{-05}
G2 phase of mitotic cell cycle	3	0	3	6.83×10^{-05}
negative regulation of DNA replication	3	0	3	0.000109
protein folding	7	0	7	0.000117
double-strand break repair via homologous recombination	3	0	3	0.000163
Proteolysis	2	7	9	0.000299
transcription regulator activity	4	2	6	0.000869
DNA recombination	4	0	4	0.00105

cell aging	2	1	3	0.00106
astrocyte activation	0	2	2	0.00140
induction of apoptosis by intracellular signals	2	1	3	0.00214
cell differentiation	4	3	7	0.00346
response to oxidative stress	3	1	4	0.00359
activation of NF-kappaB transcription factor	1	2	3	0.00376
negative regulation of protein kinase activity	0	3	3	0.00425
induction of apoptosis	1	4	5	0.00426
cell cycle arrest	3	1	4	0.00547
cell-cell signaling	1	5	6	0.00623
positive regulation of I-kappaB kinase-NF-kappaB cascade	2	2	4	0.00623
positive regulation of transcription, DNA-dependent	4	0	4	0.00737
nucleocytoplasmic transport	2	1	3	0.00817
DNA replication checkpoint	2	0	2	0.0136
response to stress	2	2	4	0.0189
35S primary transcript processing	2	0	2	0.0283
skeletal development	0	4	4	0.0340
microtubule cytoskeleton organization and biogenesis	2	1	3	0.0342
cell adhesion	1	6	7	0.0357
cholesterol metabolism	0	3	3	0.0454
traversing start control point of mitotic cell cycle	2	0	2	0.0476

Processes were ranked by p-value which is the Holm-Bonferroni adjusted probability of a random set of 203 genes containing at least the stated representation in the Pathway Studio database. The number of members within each process that are expressed lower in melan-a, higher in melan-a and the total number of members are provided in columns 2, 3 and 4 respectively.
