# Supplementary material for: Global analysis of gene expression changes during retinoic acid-induced growth arrest and differentiation of melanoma: comparison to differentially expressed genes in melanocytes vs melanoma
Source: BMC Genomics. 2008 Oct 11;9:478. doi: 10.1186/1471-2164-9-478 (PMC2572629; doi:10.1186/1471-2164-9-478)
Supplement: Additional file 5 — Members of 203 gene set which are components of the major biological processes. Using Pathway Studio, we identified biological processes which were enriched for members of the 203 gene set. Members of the five processes that are most significantly enriched according to Fisher's Exact Test are given. [file 1471-2164-9-478-S5.pdf]

Additional file 5. Members of 203 gene set which are components of the major biological processes. Using Pathway Studio software, we identified biological processes which were enriched for members of the 203 gene set. Members of the five processes that are most significantly enriched according to Fisher's Exact Test are given.

**Cell Division**	
BIRC5	baculoviral IAP repeat-containing 5
CCNE2	cyclin E2 (predicted)
CCNF	cyclin F
CDC2	cell division cycle 2 homolog A (S. pombe)
CDC25B	cell division cycle 25 homolog B (S. cerevisiae)
CDC45L	similar to cell division cycle 45 homolog (S. cerevisiae)-like
CDC6	cell division cycle 6 homolog (S. cerevisiae) (predicted)
CENPF	centromere autoantigen F
CETN3	centrin 3
CKS1B	RGD1561797 (predicted)
CKS2	similar to Cyclin-dependent kinases regulatory subunit 2 (CKS-2) (predicted)
FLJ10468	cell division cycle associated 8
FTSJ3	FtsJ homolog 3 (E. coli)
HCAP-G	similar to chromosome condensation protein G (predicted)
KIF20A	kinesin family member 20A (predicted)
MAD2L1	MAD2 (mitotic arrest deficient, homolog)-like 1 (yeast) (predicted)
SGOL1	similar to shugoshin-like 1
TOME-1	cell division cycle associated 3

**Cell Cycle**	
BIRC5	baculoviral IAP repeat-containing 5
CCNE2	cyclin E2 (predicted)
CCNF	cyclin F
CDC2	cell division cycle 2 homolog A (S. pombe)
CDC25B	cell division cycle 25 homolog B (S. cerevisiae)
CDC45L	similar to cell division cycle 45 homolog (S. cerevisiae)-like
CDC6	cell division cycle 6 homolog (S. cerevisiae) (predicted)
CDKN1A	cyclin-dependent kinase inhibitor 1A
CETN3	centrin 3
CHEK1	checkpoint kinase 1 homolog (S. pombe)
CKS1B	RGD1561797 (predicted)
CKS2	similar to Cyclin-dependent kinases regulatory subunit 2 (CKS-2) (predicted)
FLJ10468	cell division cycle associated 8
GMNN	geminin (predicted)
HCAP-G	similar to chromosome condensation protein G (predicted)
MAD2L1	MAD2 (mitotic arrest deficient, homolog)-like 1 (yeast) (predicted)
MCM2	minichromosome maintenance deficient 2 mitotin (S. cerevisiae) (predicted)
MCM6	minichromosome maintenance deficient 6 (MIS5 homolog, S. pombe)
PKMYT1	protein kinase, membrane associated tyrosine/threonine 1 (predicted)
SGOL1	similar to shugoshin-like 1
TFDP1	transcription factor Dp 1
TP53	tumor protein p53

Regulation of Cell Cycle Progression	
AXL	AXL receptor tyrosine kinase
BTG3	B-cell translocation gene 3
CCNE2	cyclin E2 (predicted)
CCNF	cyclin F
CDC2	cell division cycle 2 homolog A (S. pombe)
CDC25B	cell division cycle 25 homolog B (S. cerevisiae)
CDC45L	similar to cell division cycle 45 homolog (S. cerevisiae)-like
CDKN1A	cyclin-dependent kinase inhibitor 1A
ECT2	ect2 oncogene (predicted)
EEF1E1	eukaryotic translation elongation factor 1 epsilon 1 (predicted)
FAD1	breast cancer 2
FGF1	fibroblast growth factor 1
GADD45B	growth arrest and DNA-damage-inducible 45 beta
GMNN	geminin (predicted)
MYC	myelocytomatosis viral oncogene homolog (avian)
PRKCA	protein kinase C, alpha
RAN	RAN, member RAS oncogene family
TFDP1	transcription factor Dp 1
TP53	tumor protein p53

DNA Replication	
CDC45L	similar to cell division cycle 45 homolog (S. cerevisiae)-like
CDC6	cell division cycle 6 homolog (S. cerevisiae) (predicted)
dut	deoxyuridine triphosphatase
GINS1	similar to Hypothetical UPF0080 protein KIAA0186 (predicted)
MCM2	minichromosome maintenance deficient 2 mitotin (S. cerevisiae) (predicted)
Mcm4	minichromosome maintenance deficient 4 homolog (S. cerevisiae)
MCM6	minichromosome maintenance deficient 6 (MIS5 homolog, S. pombe)
POLA	polymerase (DNA directed), alpha 1
POLE	polymerase (DNA directed), epsilon (mapped)
RNASEH2A	ribonuclease H2, large subunit
RPA2	replication protein A2
TOP2A	topoisomerase (DNA) 2 alpha
ZRF1	DnaJ (Hsp40) homolog, subfamily C, member 2

Mitosis	
BIRC5	baculoviral IAP repeat-containing 5
CCNF	cyclin F
CDC2	cell division cycle 2 homolog A (S. pombe)
CDC25B	cell division cycle 25 homolog B (S. cerevisiae)
CDC6	cell division cycle 6 homolog (S. cerevisiae) (predicted)
CENPF	centromere autoantigen F
CETN3	centrin 3
FLJ10468	cell division cycle associated 8
HCAP-G	similar to chromosome condensation protein G (predicted)
MAD2L1	MAD2 (mitotic arrest deficient, homolog)-like 1 (yeast) (predicted)
RAN	RAN, member RAS oncogene family
SGOL1	similar to shugoshin-like 1
TOPK	PDZ binding kinase (predicted)
